# Supplementary material for: ABEL: an active-learning behavior estimation and labeling platform
Source: bioRxiv. 2026 Sep 9:2026.08.30.748115. Preprint. [Version 2] doi: 10.64898/2026.08.30.748115 (PMC13622494; doi:10.64898/2026.08.30.748115)
Supplement: Supplementary file 2 [file media-2.pdf]

feature	modality	3CS	EPM	FC
back_left_paw_acceleration_energy	kinematics	0	0	0
back_left_paw_acceleration_max	kinematics	0	0	0
back_left_paw_acceleration_mean	kinematics	0	0	0
back_left_paw_acceleration_median	kinematics	0	0	0
back_left_paw_acceleration_p10	kinematics	0	0	0
back_left_paw_acceleration_p90	kinematics	0	0	0
back_left_paw_acceleration_periodicity	kinematics	0	0	0
back_left_paw_acceleration_std	kinematics	0	0	0
back_left_paw_forward_velocity_energy	kinematics	0	0	0
back_left_paw_forward_velocity_max	kinematics	0	0	0
back_left_paw_forward_velocity_mean	kinematics	0	0	0
back_left_paw_forward_velocity_median	kinematics	0	0	0
back_left_paw_forward_velocity_p10	kinematics	0	0	0
back_left_paw_forward_velocity_p90	kinematics	0	0	0
back_left_paw_forward_velocity_periodicity	kinematics	0	0	0
back_left_paw_forward_velocity_std	kinematics	0	0	0
back_left_paw_jerk_energy	kinematics	0	0	0
back_left_paw_jerk_max	kinematics	0	0	0
back_left_paw_jerk_mean	kinematics	0	0	0
back_left_paw_jerk_median	kinematics	0	0	0
back_left_paw_jerk_p10	kinematics	0	0	0
back_left_paw_jerk_p90	kinematics	0	0	0
back_left_paw_jerk_periodicity	kinematics	0	0	0
back_left_paw_jerk_std	kinematics	0	0	0
back_left_paw_lateral_velocity_energy	kinematics	0	0	0
back_left_paw_lateral_velocity_max	kinematics	0	0	0
back_left_paw_lateral_velocity_mean	kinematics	0	0	0
back_left_paw_lateral_velocity_median	kinematics	0	0	0
back_left_paw_lateral_velocity_p10	kinematics	0	0	0
back_left_paw_lateral_velocity_p90	kinematics	0	0	0
back_left_paw_lateral_velocity_periodicity	kinematics	0	0	0
back_left_paw_lateral_velocity_std	kinematics	0	0	0
back_left_paw_speed_energy	kinematics	0	0	0
back_left_paw_speed_max	kinematics	0	0	0
back_left_paw_speed_mean	kinematics	0	0	0
back_left_paw_speed_median	kinematics	0	0	0
back_left_paw_speed_p10	kinematics	0	0	0
back_left_paw_speed_p90	kinematics	0	0	0
back_left_paw_speed_periodicity	kinematics	0	0	0
back_left_paw_speed_std	kinematics	0	0	0
back_left_paw_velocity_x_energy	kinematics	0	0	0
back_left_paw_velocity_x_max	kinematics	0	0	0
back_left_paw_velocity_x_mean	kinematics	0	0	0

back_left_paw_velocity_x_median	kinematics	0	0	0
back_left_paw_velocity_x_p10	kinematics	0	0	0
back_left_paw_velocity_x_p90	kinematics	0	0	0
back_left_paw_velocity_x_periodicity	kinematics	0	0	0
back_left_paw_velocity_x_std	kinematics	0	0	0
back_left_paw_velocity_y_energy	kinematics	0	0	0
back_left_paw_velocity_y_max	kinematics	0	0	0
back_left_paw_velocity_y_mean	kinematics	0	0	0
back_left_paw_velocity_y_median	kinematics	0	0	0
back_left_paw_velocity_y_p10	kinematics	0	0	0
back_left_paw_velocity_y_p90	kinematics	0	0	0
back_left_paw_velocity_y_periodicity	kinematics	0	0	0
back_left_paw_velocity_y_std	kinematics	0	0	0
back_right_paw_acceleration_energy	kinematics	0	0	0
back_right_paw_acceleration_max	kinematics	0	0	0
back_right_paw_acceleration_mean	kinematics	0	0	0
back_right_paw_acceleration_median	kinematics	0	0	0
back_right_paw_acceleration_p10	kinematics	0	0	0
back_right_paw_acceleration_p90	kinematics	0	0	0
back_right_paw_acceleration_periodicity	kinematics	0	0	0
back_right_paw_acceleration_std	kinematics	0	0	0
back_right_paw_forward_velocity_energy	kinematics	0	0	0
back_right_paw_forward_velocity_max	kinematics	0	0	0
back_right_paw_forward_velocity_mean	kinematics	0	0	0
back_right_paw_forward_velocity_median	kinematics	0	0	0
back_right_paw_forward_velocity_p10	kinematics	0	0	0
back_right_paw_forward_velocity_p90	kinematics	0	0	0
back_right_paw_forward_velocity_periodicity	kinematics	0	0	0
back_right_paw_forward_velocity_std	kinematics	0	0	0
back_right_paw_jerk_energy	kinematics	0	0	0
back_right_paw_jerk_max	kinematics	0	0	0
back_right_paw_jerk_mean	kinematics	0	0	0
back_right_paw_jerk_median	kinematics	0	0	0
back_right_paw_jerk_p10	kinematics	0	0	0
back_right_paw_jerk_p90	kinematics	0	0	0
back_right_paw_jerk_periodicity	kinematics	0	0	0
back_right_paw_jerk_std	kinematics	0	0	0
back_right_paw_lateral_velocity_energy	kinematics	0	0	0
back_right_paw_lateral_velocity_max	kinematics	0	0	0
back_right_paw_lateral_velocity_mean	kinematics	0	0	0
back_right_paw_lateral_velocity_median	kinematics	0	0	0
back_right_paw_lateral_velocity_p10	kinematics	0	0	0
back_right_paw_lateral_velocity_p90	kinematics	0	0	0
back_right_paw_lateral_velocity_periodicity	kinematics	0	0	0

back_right_paw_lateral_velocity_std	kinematics	0	0	0
back_right_paw_speed_energy	kinematics	0	0	0
back_right_paw_speed_max	kinematics	0	0	0
back_right_paw_speed_mean	kinematics	0	0	0
back_right_paw_speed_median	kinematics	0	0	0
back_right_paw_speed_p10	kinematics	0	0	0
back_right_paw_speed_p90	kinematics	0	0	0
back_right_paw_speed_periodicity	kinematics	0	0	0
back_right_paw_speed_std	kinematics	0	0	0
back_right_paw_velocity_x_energy	kinematics	0	0	0
back_right_paw_velocity_x_max	kinematics	0	0	0
back_right_paw_velocity_x_mean	kinematics	0	0	0
back_right_paw_velocity_x_median	kinematics	0	0	0
back_right_paw_velocity_x_p10	kinematics	0	0	0
back_right_paw_velocity_x_p90	kinematics	0	0	0
back_right_paw_velocity_x_periodicity	kinematics	0	0	0
back_right_paw_velocity_x_std	kinematics	0	0	0
back_right_paw_velocity_y_energy	kinematics	0	0	0
back_right_paw_velocity_y_max	kinematics	0	0	0
back_right_paw_velocity_y_mean	kinematics	0	0	0
back_right_paw_velocity_y_median	kinematics	0	0	0
back_right_paw_velocity_y_p10	kinematics	0	0	0
back_right_paw_velocity_y_p90	kinematics	0	0	0
back_right_paw_velocity_y_periodicity	kinematics	0	0	0
back_right_paw_velocity_y_std	kinematics	0	0	0
body_angle_to_roi_1_delta	context (ROI/target)	1	1	1
body_angle_to_roi_1_energy	context (ROI/target)	1	1	1
body_angle_to_roi_1_max	context (ROI/target)	1	1	1
body_angle_to_roi_1_mean	context (ROI/target)	1	1	1
body_angle_to_roi_1_median	context (ROI/target)	1	1	1
body_angle_to_roi_1_p10	context (ROI/target)	1	1	1
body_angle_to_roi_1_p90	context (ROI/target)	1	1	1
body_angle_to_roi_1_periodicity	context (ROI/target)	1	1	1
body_angle_to_roi_1_std	context (ROI/target)	1	1	1
body_angle_to_roi_1_trend	context (ROI/target)	1	1	1
body_angle_to_roi_2_delta	context (ROI/target)	1	0	0
body_angle_to_roi_2_energy	context (ROI/target)	1	0	0
body_angle_to_roi_2_max	context (ROI/target)	1	0	0
body_angle_to_roi_2_mean	context (ROI/target)	1	0	0
body_angle_to_roi_2_median	context (ROI/target)	1	0	0
body_angle_to_roi_2_p10	context (ROI/target)	1	0	0
body_angle_to_roi_2_p90	context (ROI/target)	1	0	0
body_angle_to_roi_2_periodicity	context (ROI/target)	1	0	0
body_angle_to_roi_2_std	context (ROI/target)	1	0	0

body_angle_to_roi_2_trend	context (ROI/target)	1	0	0
body_angle_to_target_delta	context (ROI/target)	1	1	1
body_angle_to_target_energy	context (ROI/target)	1	1	1
body_angle_to_target_max	context (ROI/target)	1	1	1
body_angle_to_target_mean	context (ROI/target)	1	1	1
body_angle_to_target_median	context (ROI/target)	1	1	1
body_angle_to_target_p10	context (ROI/target)	1	1	1
body_angle_to_target_p90	context (ROI/target)	1	1	1
body_angle_to_target_periodicity	context (ROI/target)	1	1	1
body_angle_to_target_std	context (ROI/target)	1	1	1
body_angle_to_target_trend	context (ROI/target)	1	1	1
body_centroid_roi_1_axial_abs_energy	context (ROI/target)	1	1	1
body_centroid_roi_1_axial_abs_max	context (ROI/target)	1	1	1
body_centroid_roi_1_axial_abs_mean	context (ROI/target)	1	1	1
body_centroid_roi_1_axial_abs_median	context (ROI/target)	1	1	1
body_centroid_roi_1_axial_abs_p10	context (ROI/target)	1	1	1
body_centroid_roi_1_axial_abs_p90	context (ROI/target)	1	1	1
body_centroid_roi_1_axial_abs_periodicity	context (ROI/target)	1	1	1
body_centroid_roi_1_axial_abs_std	context (ROI/target)	1	1	1
body_centroid_roi_1_axial_energy	context (ROI/target)	1	1	1
body_centroid_roi_1_axial_max	context (ROI/target)	1	1	1
body_centroid_roi_1_axial_mean	context (ROI/target)	1	1	1
body_centroid_roi_1_axial_median	context (ROI/target)	1	1	1
body_centroid_roi_1_axial_p10	context (ROI/target)	1	1	1
body_centroid_roi_1_axial_p90	context (ROI/target)	1	1	1
body_centroid_roi_1_axial_periodicity	context (ROI/target)	1	1	1
body_centroid_roi_1_axial_std	context (ROI/target)	1	1	1
body_centroid_roi_1_lateral_abs_energy	context (ROI/target)	1	1	1
body_centroid_roi_1_lateral_abs_max	context (ROI/target)	1	1	1
body_centroid_roi_1_lateral_abs_mean	context (ROI/target)	1	1	1
body_centroid_roi_1_lateral_abs_median	context (ROI/target)	1	1	1
body_centroid_roi_1_lateral_abs_p10	context (ROI/target)	1	1	1
body_centroid_roi_1_lateral_abs_p90	context (ROI/target)	1	1	1
body_centroid_roi_1_lateral_abs_periodicity	context (ROI/target)	1	1	1
body_centroid_roi_1_lateral_abs_std	context (ROI/target)	1	1	1
body_centroid_roi_1_lateral_energy	context (ROI/target)	1	1	1
body_centroid_roi_1_lateral_max	context (ROI/target)	1	1	1
body_centroid_roi_1_lateral_mean	context (ROI/target)	1	1	1
body_centroid_roi_1_lateral_median	context (ROI/target)	1	1	1
body_centroid_roi_1_lateral_p10	context (ROI/target)	1	1	1
body_centroid_roi_1_lateral_p90	context (ROI/target)	1	1	1
body_centroid_roi_1_lateral_periodicity	context (ROI/target)	1	1	1
body_centroid_roi_1_lateral_std	context (ROI/target)	1	1	1
body_centroid_roi_2_axial_abs_energy	context (ROI/target)	1	0	0

body_centroid_roi_2_axial_abs_max	context (ROI/target)	1	0	0
body_centroid_roi_2_axial_abs_mean	context (ROI/target)	1	0	0
body_centroid_roi_2_axial_abs_median	context (ROI/target)	1	0	0
body_centroid_roi_2_axial_abs_p10	context (ROI/target)	1	0	0
body_centroid_roi_2_axial_abs_p90	context (ROI/target)	1	0	0
body_centroid_roi_2_axial_abs_periodicity	context (ROI/target)	1	0	0
body_centroid_roi_2_axial_abs_std	context (ROI/target)	1	0	0
body_centroid_roi_2_axial_energy	context (ROI/target)	1	0	0
body_centroid_roi_2_axial_max	context (ROI/target)	1	0	0
body_centroid_roi_2_axial_mean	context (ROI/target)	1	0	0
body_centroid_roi_2_axial_median	context (ROI/target)	1	0	0
body_centroid_roi_2_axial_p10	context (ROI/target)	1	0	0
body_centroid_roi_2_axial_p90	context (ROI/target)	1	0	0
body_centroid_roi_2_axial_periodicity	context (ROI/target)	1	0	0
body_centroid_roi_2_axial_std	context (ROI/target)	1	0	0
body_centroid_roi_2_lateral_abs_energy	context (ROI/target)	1	0	0
body_centroid_roi_2_lateral_abs_max	context (ROI/target)	1	0	0
body_centroid_roi_2_lateral_abs_mean	context (ROI/target)	1	0	0
body_centroid_roi_2_lateral_abs_median	context (ROI/target)	1	0	0
body_centroid_roi_2_lateral_abs_p10	context (ROI/target)	1	0	0
body_centroid_roi_2_lateral_abs_p90	context (ROI/target)	1	0	0
body_centroid_roi_2_lateral_abs_periodicity	context (ROI/target)	1	0	0
body_centroid_roi_2_lateral_abs_std	context (ROI/target)	1	0	0
body_centroid_roi_2_lateral_energy	context (ROI/target)	1	0	0
body_centroid_roi_2_lateral_max	context (ROI/target)	1	0	0
body_centroid_roi_2_lateral_mean	context (ROI/target)	1	0	0
body_centroid_roi_2_lateral_median	context (ROI/target)	1	0	0
body_centroid_roi_2_lateral_p10	context (ROI/target)	1	0	0
body_centroid_roi_2_lateral_p90	context (ROI/target)	1	0	0
body_centroid_roi_2_lateral_periodicity	context (ROI/target)	1	0	0
body_centroid_roi_2_lateral_std	context (ROI/target)	1	0	0
body_centroid_to_roi_1_corner_dist_delta	context (ROI/target)	1	1	1
body_centroid_to_roi_1_corner_dist_energy	context (ROI/target)	1	1	1
body_centroid_to_roi_1_corner_dist_max	context (ROI/target)	1	1	1
body_centroid_to_roi_1_corner_dist_mean	context (ROI/target)	1	1	1
body_centroid_to_roi_1_corner_dist_median	context (ROI/target)	1	1	1
body_centroid_to_roi_1_corner_dist_p10	context (ROI/target)	1	1	1
body_centroid_to_roi_1_corner_dist_p90	context (ROI/target)	1	1	1
body_centroid_to_roi_1_corner_dist_periodicity	context (ROI/target)	1	1	1
body_centroid_to_roi_1_corner_dist_std	context (ROI/target)	1	1	1
body_centroid_to_roi_1_corner_dist_trend	context (ROI/target)	1	1	1
body_centroid_to_roi_1_dist_delta	context (ROI/target)	1	1	1
body_centroid_to_roi_1_dist_energy	context (ROI/target)	1	1	1
body_centroid_to_roi_1_dist_max	context (ROI/target)	1	1	1

body_centroid_to_roi_1_dist_mean	context (ROI/target)	1	1	1
body_centroid_to_roi_1_dist_median	context (ROI/target)	1	1	1
body_centroid_to_roi_1_dist_p10	context (ROI/target)	1	1	1
body_centroid_to_roi_1_dist_p90	context (ROI/target)	1	1	1
body_centroid_to_roi_1_dist_periodicity	context (ROI/target)	1	1	1
body_centroid_to_roi_1_dist_std	context (ROI/target)	1	1	1
body_centroid_to_roi_1_dist_trend	context (ROI/target)	1	1	1
body_centroid_to_roi_1_edge_dist_delta	context (ROI/target)	1	1	1
body_centroid_to_roi_1_edge_dist_energy	context (ROI/target)	1	1	1
body_centroid_to_roi_1_edge_dist_max	context (ROI/target)	1	1	1
body_centroid_to_roi_1_edge_dist_mean	context (ROI/target)	1	1	1
body_centroid_to_roi_1_edge_dist_median	context (ROI/target)	1	1	1
body_centroid_to_roi_1_edge_dist_p10	context (ROI/target)	1	1	1
body_centroid_to_roi_1_edge_dist_p90	context (ROI/target)	1	1	1
body_centroid_to_roi_1_edge_dist_periodicity	context (ROI/target)	1	1	1
body_centroid_to_roi_1_edge_dist_std	context (ROI/target)	1	1	1
body_centroid_to_roi_1_edge_dist_trend	context (ROI/target)	1	1	1
body_centroid_to_roi_1_signed_dist_delta	context (ROI/target)	1	1	1
body_centroid_to_roi_1_signed_dist_energy	context (ROI/target)	1	1	1
body_centroid_to_roi_1_signed_dist_max	context (ROI/target)	1	1	1
body_centroid_to_roi_1_signed_dist_mean	context (ROI/target)	1	1	1
body_centroid_to_roi_1_signed_dist_median	context (ROI/target)	1	1	1
body_centroid_to_roi_1_signed_dist_p10	context (ROI/target)	1	1	1
body_centroid_to_roi_1_signed_dist_p90	context (ROI/target)	1	1	1
body_centroid_to_roi_1_signed_dist_periodicity	context (ROI/target)	1	1	1
body_centroid_to_roi_1_signed_dist_std	context (ROI/target)	1	1	1
body_centroid_to_roi_1_signed_dist_trend	context (ROI/target)	1	1	1
body_centroid_to_roi_2_corner_dist_delta	context (ROI/target)	1	0	0
body_centroid_to_roi_2_corner_dist_energy	context (ROI/target)	1	0	0
body_centroid_to_roi_2_corner_dist_max	context (ROI/target)	1	0	0
body_centroid_to_roi_2_corner_dist_mean	context (ROI/target)	1	0	0
body_centroid_to_roi_2_corner_dist_median	context (ROI/target)	1	0	0
body_centroid_to_roi_2_corner_dist_p10	context (ROI/target)	1	0	0
body_centroid_to_roi_2_corner_dist_p90	context (ROI/target)	1	0	0
body_centroid_to_roi_2_corner_dist_periodicity	context (ROI/target)	1	0	0
body_centroid_to_roi_2_corner_dist_std	context (ROI/target)	1	0	0
body_centroid_to_roi_2_corner_dist_trend	context (ROI/target)	1	0	0
body_centroid_to_roi_2_dist_delta	context (ROI/target)	1	0	0
body_centroid_to_roi_2_dist_energy	context (ROI/target)	1	0	0
body_centroid_to_roi_2_dist_max	context (ROI/target)	1	0	0
body_centroid_to_roi_2_dist_mean	context (ROI/target)	1	0	0
body_centroid_to_roi_2_dist_median	context (ROI/target)	1	0	0
body_centroid_to_roi_2_dist_p10	context (ROI/target)	1	0	0
body_centroid_to_roi_2_dist_p90	context (ROI/target)	1	0	0

body_centroid_to_roi_2_dist_periodicity	context (ROI/target)	1	0	0
body_centroid_to_roi_2_dist_std	context (ROI/target)	1	0	0
body_centroid_to_roi_2_dist_trend	context (ROI/target)	1	0	0
body_centroid_to_roi_2_edge_dist_delta	context (ROI/target)	1	0	0
body_centroid_to_roi_2_edge_dist_energy	context (ROI/target)	1	0	0
body_centroid_to_roi_2_edge_dist_max	context (ROI/target)	1	0	0
body_centroid_to_roi_2_edge_dist_mean	context (ROI/target)	1	0	0
body_centroid_to_roi_2_edge_dist_median	context (ROI/target)	1	0	0
body_centroid_to_roi_2_edge_dist_p10	context (ROI/target)	1	0	0
body_centroid_to_roi_2_edge_dist_p90	context (ROI/target)	1	0	0
body_centroid_to_roi_2_edge_dist_periodicity	context (ROI/target)	1	0	0
body_centroid_to_roi_2_edge_dist_std	context (ROI/target)	1	0	0
body_centroid_to_roi_2_edge_dist_trend	context (ROI/target)	1	0	0
body_centroid_to_roi_2_signed_dist_delta	context (ROI/target)	1	0	0
body_centroid_to_roi_2_signed_dist_energy	context (ROI/target)	1	0	0
body_centroid_to_roi_2_signed_dist_max	context (ROI/target)	1	0	0
body_centroid_to_roi_2_signed_dist_mean	context (ROI/target)	1	0	0
body_centroid_to_roi_2_signed_dist_median	context (ROI/target)	1	0	0
body_centroid_to_roi_2_signed_dist_p10	context (ROI/target)	1	0	0
body_centroid_to_roi_2_signed_dist_p90	context (ROI/target)	1	0	0
body_centroid_to_roi_2_signed_dist_periodicity	context (ROI/target)	1	0	0
body_centroid_to_roi_2_signed_dist_std	context (ROI/target)	1	0	0
body_centroid_to_roi_2_signed_dist_trend	context (ROI/target)	1	0	0
body_centroid_to_target_dist_delta	context (ROI/target)	1	1	1
body_centroid_to_target_dist_energy	context (ROI/target)	1	1	1
body_centroid_to_target_dist_max	context (ROI/target)	1	1	1
body_centroid_to_target_dist_mean	context (ROI/target)	1	1	1
body_centroid_to_target_dist_median	context (ROI/target)	1	1	1
body_centroid_to_target_dist_p10	context (ROI/target)	1	1	1
body_centroid_to_target_dist_p90	context (ROI/target)	1	1	1
body_centroid_to_target_dist_periodicity	context (ROI/target)	1	1	1
body_centroid_to_target_dist_std	context (ROI/target)	1	1	1
body_centroid_to_target_dist_trend	context (ROI/target)	1	1	1
body_left_acceleration_energy	kinematics	0	1	0
body_left_acceleration_max	kinematics	0	1	0
body_left_acceleration_mean	kinematics	0	1	0
body_left_acceleration_median	kinematics	0	1	0
body_left_acceleration_p10	kinematics	0	1	0
body_left_acceleration_p90	kinematics	0	1	0
body_left_acceleration_periodicity	kinematics	0	1	0
body_left_acceleration_std	kinematics	0	1	0
body_left_forward_velocity_energy	kinematics	0	1	0
body_left_forward_velocity_max	kinematics	0	1	0
body_left_forward_velocity_mean	kinematics	0	1	0

body_left_forward_velocity_median	kinematics	0	1	0
body_left_forward_velocity_p10	kinematics	0	1	0
body_left_forward_velocity_p90	kinematics	0	1	0
body_left_forward_velocity_periodicity	kinematics	0	1	0
body_left_forward_velocity_std	kinematics	0	1	0
body_left_jerk_energy	kinematics	0	1	0
body_left_jerk_max	kinematics	0	1	0
body_left_jerk_mean	kinematics	0	1	0
body_left_jerk_median	kinematics	0	1	0
body_left_jerk_p10	kinematics	0	1	0
body_left_jerk_p90	kinematics	0	1	0
body_left_jerk_periodicity	kinematics	0	1	0
body_left_jerk_std	kinematics	0	1	0
body_left_lateral_velocity_energy	kinematics	0	1	0
body_left_lateral_velocity_max	kinematics	0	1	0
body_left_lateral_velocity_mean	kinematics	0	1	0
body_left_lateral_velocity_median	kinematics	0	1	0
body_left_lateral_velocity_p10	kinematics	0	1	0
body_left_lateral_velocity_p90	kinematics	0	1	0
body_left_lateral_velocity_periodicity	kinematics	0	1	0
body_left_lateral_velocity_std	kinematics	0	1	0
body_left_speed_energy	kinematics	0	1	0
body_left_speed_max	kinematics	0	1	0
body_left_speed_mean	kinematics	0	1	0
body_left_speed_median	kinematics	0	1	0
body_left_speed_p10	kinematics	0	1	0
body_left_speed_p90	kinematics	0	1	0
body_left_speed_periodicity	kinematics	0	1	0
body_left_speed_std	kinematics	0	1	0
body_left_velocity_x_energy	kinematics	0	1	0
body_left_velocity_x_max	kinematics	0	1	0
body_left_velocity_x_mean	kinematics	0	1	0
body_left_velocity_x_median	kinematics	0	1	0
body_left_velocity_x_p10	kinematics	0	1	0
body_left_velocity_x_p90	kinematics	0	1	0
body_left_velocity_x_periodicity	kinematics	0	1	0
body_left_velocity_x_std	kinematics	0	1	0
body_left_velocity_y_energy	kinematics	0	1	0
body_left_velocity_y_max	kinematics	0	1	0
body_left_velocity_y_mean	kinematics	0	1	0
body_left_velocity_y_median	kinematics	0	1	0
body_left_velocity_y_p10	kinematics	0	1	0
body_left_velocity_y_p90	kinematics	0	1	0
body_left_velocity_y_periodicity	kinematics	0	1	0

body_left_velocity_y_std	kinematics	0	1	0
body_length_px_energy	kinematics	1	1	1
body_length_px_max	pose geometry	1	1	1
body_length_px_mean	pose geometry	1	1	1
body_length_px_median	pose geometry	1	1	1
body_length_px_p10	pose geometry	1	1	1
body_length_px_p90	pose geometry	1	1	1
body_length_px_periodicity	pose geometry	1	1	1
body_length_px_std	kinematics	1	1	1
body_mid_acceleration_energy	kinematics	0	1	0
body_mid_acceleration_max	kinematics	0	1	0
body_mid_acceleration_mean	kinematics	0	1	0
body_mid_acceleration_median	kinematics	0	1	0
body_mid_acceleration_p10	kinematics	0	1	0
body_mid_acceleration_p90	kinematics	0	1	0
body_mid_acceleration_periodicity	kinematics	0	1	0
body_mid_acceleration_std	kinematics	0	1	0
body_mid_forward_velocity_energy	kinematics	0	1	0
body_mid_forward_velocity_max	kinematics	0	1	0
body_mid_forward_velocity_mean	kinematics	0	1	0
body_mid_forward_velocity_median	kinematics	0	1	0
body_mid_forward_velocity_p10	kinematics	0	1	0
body_mid_forward_velocity_p90	kinematics	0	1	0
body_mid_forward_velocity_periodicity	kinematics	0	1	0
body_mid_forward_velocity_std	kinematics	0	1	0
body_mid_jerk_energy	kinematics	0	1	0
body_mid_jerk_max	kinematics	0	1	0
body_mid_jerk_mean	kinematics	0	1	0
body_mid_jerk_median	kinematics	0	1	0
body_mid_jerk_p10	kinematics	0	1	0
body_mid_jerk_p90	kinematics	0	1	0
body_mid_jerk_periodicity	kinematics	0	1	0
body_mid_jerk_std	kinematics	0	1	0
body_mid_lateral_velocity_energy	kinematics	0	1	0
body_mid_lateral_velocity_max	kinematics	0	1	0
body_mid_lateral_velocity_mean	kinematics	0	1	0
body_mid_lateral_velocity_median	kinematics	0	1	0
body_mid_lateral_velocity_p10	kinematics	0	1	0
body_mid_lateral_velocity_p90	kinematics	0	1	0
body_mid_lateral_velocity_periodicity	kinematics	0	1	0
body_mid_lateral_velocity_std	kinematics	0	1	0
body_mid_speed_energy	kinematics	0	1	0
body_mid_speed_max	kinematics	0	1	0
body_mid_speed_mean	kinematics	0	1	0

body_mid_speed_median	kinematics	0	1	0
body_mid_speed_p10	kinematics	0	1	0
body_mid_speed_p90	kinematics	0	1	0
body_mid_speed_periodicity	kinematics	0	1	0
body_mid_speed_std	kinematics	0	1	0
body_mid_velocity_x_energy	kinematics	0	1	0
body_mid_velocity_x_max	kinematics	0	1	0
body_mid_velocity_x_mean	kinematics	0	1	0
body_mid_velocity_x_median	kinematics	0	1	0
body_mid_velocity_x_p10	kinematics	0	1	0
body_mid_velocity_x_p90	kinematics	0	1	0
body_mid_velocity_x_periodicity	kinematics	0	1	0
body_mid_velocity_x_std	kinematics	0	1	0
body_mid_velocity_y_energy	kinematics	0	1	0
body_mid_velocity_y_max	kinematics	0	1	0
body_mid_velocity_y_mean	kinematics	0	1	0
body_mid_velocity_y_median	kinematics	0	1	0
body_mid_velocity_y_p10	kinematics	0	1	0
body_mid_velocity_y_p90	kinematics	0	1	0
body_mid_velocity_y_periodicity	kinematics	0	1	0
body_mid_velocity_y_std	kinematics	0	1	0
body_orientation_delta	kinematics	1	1	1
body_orientation_energy	kinematics	1	1	1
body_orientation_max	pose geometry	1	1	1
body_orientation_mean	pose geometry	1	1	1
body_orientation_median	pose geometry	1	1	1
body_orientation_p10	pose geometry	1	1	1
body_orientation_p90	pose geometry	1	1	1
body_orientation_periodicity	pose geometry	1	1	1
body_orientation_std	kinematics	1	1	1
body_orientation_trend	pose geometry	1	1	1
body_right_acceleration_energy	kinematics	0	1	0
body_right_acceleration_max	kinematics	0	1	0
body_right_acceleration_mean	kinematics	0	1	0
body_right_acceleration_median	kinematics	0	1	0
body_right_acceleration_p10	kinematics	0	1	0
body_right_acceleration_p90	kinematics	0	1	0
body_right_acceleration_periodicity	kinematics	0	1	0
body_right_acceleration_std	kinematics	0	1	0
body_right_forward_velocity_energy	kinematics	0	1	0
body_right_forward_velocity_max	kinematics	0	1	0
body_right_forward_velocity_mean	kinematics	0	1	0
body_right_forward_velocity_median	kinematics	0	1	0
body_right_forward_velocity_p10	kinematics	0	1	0

body_right_forward_velocity_p90	kinematics	0	1	0
body_right_forward_velocity_periodicity	kinematics	0	1	0
body_right_forward_velocity_std	kinematics	0	1	0
body_right_jerk_energy	kinematics	0	1	0
body_right_jerk_max	kinematics	0	1	0
body_right_jerk_mean	kinematics	0	1	0
body_right_jerk_median	kinematics	0	1	0
body_right_jerk_p10	kinematics	0	1	0
body_right_jerk_p90	kinematics	0	1	0
body_right_jerk_periodicity	kinematics	0	1	0
body_right_jerk_std	kinematics	0	1	0
body_right_lateral_velocity_energy	kinematics	0	1	0
body_right_lateral_velocity_max	kinematics	0	1	0
body_right_lateral_velocity_mean	kinematics	0	1	0
body_right_lateral_velocity_median	kinematics	0	1	0
body_right_lateral_velocity_p10	kinematics	0	1	0
body_right_lateral_velocity_p90	kinematics	0	1	0
body_right_lateral_velocity_periodicity	kinematics	0	1	0
body_right_lateral_velocity_std	kinematics	0	1	0
body_right_speed_energy	kinematics	0	1	0
body_right_speed_max	kinematics	0	1	0
body_right_speed_mean	kinematics	0	1	0
body_right_speed_median	kinematics	0	1	0
body_right_speed_p10	kinematics	0	1	0
body_right_speed_p90	kinematics	0	1	0
body_right_speed_periodicity	kinematics	0	1	0
body_right_speed_std	kinematics	0	1	0
body_right_velocity_x_energy	kinematics	0	1	0
body_right_velocity_x_max	kinematics	0	1	0
body_right_velocity_x_mean	kinematics	0	1	0
body_right_velocity_x_median	kinematics	0	1	0
body_right_velocity_x_p10	kinematics	0	1	0
body_right_velocity_x_p90	kinematics	0	1	0
body_right_velocity_x_periodicity	kinematics	0	1	0
body_right_velocity_x_std	kinematics	0	1	0
body_right_velocity_y_energy	kinematics	0	1	0
body_right_velocity_y_max	kinematics	0	1	0
body_right_velocity_y_mean	kinematics	0	1	0
body_right_velocity_y_median	kinematics	0	1	0
body_right_velocity_y_p10	kinematics	0	1	0
body_right_velocity_y_p90	kinematics	0	1	0
body_right_velocity_y_periodicity	kinematics	0	1	0
body_right_velocity_y_std	kinematics	0	1	0
center_acceleration_energy	context (ROI/target)	0	0	1

center_acceleration_max	context (ROI/target)	0	0	1
center_acceleration_mean	context (ROI/target)	0	0	1
center_acceleration_median	context (ROI/target)	0	0	1
center_acceleration_p10	context (ROI/target)	0	0	1
center_acceleration_p90	context (ROI/target)	0	0	1
center_acceleration_periodicity	context (ROI/target)	0	0	1
center_acceleration_std	context (ROI/target)	0	0	1
center_body_acceleration_energy	context (ROI/target)	1	0	0
center_body_acceleration_max	context (ROI/target)	1	0	0
center_body_acceleration_mean	context (ROI/target)	1	0	0
center_body_acceleration_median	context (ROI/target)	1	0	0
center_body_acceleration_p10	context (ROI/target)	1	0	0
center_body_acceleration_p90	context (ROI/target)	1	0	0
center_body_acceleration_periodicity	context (ROI/target)	1	0	0
center_body_acceleration_std	context (ROI/target)	1	0	0
center_body_forward_velocity_energy	context (ROI/target)	1	0	0
center_body_forward_velocity_max	context (ROI/target)	1	0	0
center_body_forward_velocity_mean	context (ROI/target)	1	0	0
center_body_forward_velocity_median	context (ROI/target)	1	0	0
center_body_forward_velocity_p10	context (ROI/target)	1	0	0
center_body_forward_velocity_p90	context (ROI/target)	1	0	0
center_body_forward_velocity_periodicity	context (ROI/target)	1	0	0
center_body_forward_velocity_std	context (ROI/target)	1	0	0
center_body_jerk_energy	context (ROI/target)	1	0	0
center_body_jerk_max	context (ROI/target)	1	0	0
center_body_jerk_mean	context (ROI/target)	1	0	0
center_body_jerk_median	context (ROI/target)	1	0	0
center_body_jerk_p10	context (ROI/target)	1	0	0
center_body_jerk_p90	context (ROI/target)	1	0	0
center_body_jerk_periodicity	context (ROI/target)	1	0	0
center_body_jerk_std	context (ROI/target)	1	0	0
center_body_lateral_velocity_energy	context (ROI/target)	1	0	0
center_body_lateral_velocity_max	context (ROI/target)	1	0	0
center_body_lateral_velocity_mean	context (ROI/target)	1	0	0
center_body_lateral_velocity_median	context (ROI/target)	1	0	0
center_body_lateral_velocity_p10	context (ROI/target)	1	0	0
center_body_lateral_velocity_p90	context (ROI/target)	1	0	0
center_body_lateral_velocity_periodicity	context (ROI/target)	1	0	0
center_body_lateral_velocity_std	context (ROI/target)	1	0	0
center_body_speed_energy	context (ROI/target)	1	0	0
center_body_speed_max	context (ROI/target)	1	0	0
center_body_speed_mean	context (ROI/target)	1	0	0
center_body_speed_median	context (ROI/target)	1	0	0
center_body_speed_p10	context (ROI/target)	1	0	0

center_body_speed_p90	context (ROI/target)	1	0	0
center_body_speed_periodicity	context (ROI/target)	1	0	0
center_body_speed_std	context (ROI/target)	1	0	0
center_body_velocity_x_energy	context (ROI/target)	1	0	0
center_body_velocity_x_max	context (ROI/target)	1	0	0
center_body_velocity_x_mean	context (ROI/target)	1	0	0
center_body_velocity_x_median	context (ROI/target)	1	0	0
center_body_velocity_x_p10	context (ROI/target)	1	0	0
center_body_velocity_x_p90	context (ROI/target)	1	0	0
center_body_velocity_x_periodicity	context (ROI/target)	1	0	0
center_body_velocity_x_std	context (ROI/target)	1	0	0
center_body_velocity_y_energy	context (ROI/target)	1	0	0
center_body_velocity_y_max	context (ROI/target)	1	0	0
center_body_velocity_y_mean	context (ROI/target)	1	0	0
center_body_velocity_y_median	context (ROI/target)	1	0	0
center_body_velocity_y_p10	context (ROI/target)	1	0	0
center_body_velocity_y_p90	context (ROI/target)	1	0	0
center_body_velocity_y_periodicity	context (ROI/target)	1	0	0
center_body_velocity_y_std	context (ROI/target)	1	0	0
center_forward_velocity_energy	context (ROI/target)	0	0	1
center_forward_velocity_max	context (ROI/target)	0	0	1
center_forward_velocity_mean	context (ROI/target)	0	0	1
center_forward_velocity_median	context (ROI/target)	0	0	1
center_forward_velocity_p10	context (ROI/target)	0	0	1
center_forward_velocity_p90	context (ROI/target)	0	0	1
center_forward_velocity_periodicity	context (ROI/target)	0	0	1
center_forward_velocity_std	context (ROI/target)	0	0	1
center_jerk_energy	context (ROI/target)	0	0	1
center_jerk_max	context (ROI/target)	0	0	1
center_jerk_mean	context (ROI/target)	0	0	1
center_jerk_median	context (ROI/target)	0	0	1
center_jerk_p10	context (ROI/target)	0	0	1
center_jerk_p90	context (ROI/target)	0	0	1
center_jerk_periodicity	context (ROI/target)	0	0	1
center_jerk_std	context (ROI/target)	0	0	1
center_lateral_velocity_energy	context (ROI/target)	0	0	1
center_lateral_velocity_max	context (ROI/target)	0	0	1
center_lateral_velocity_mean	context (ROI/target)	0	0	1
center_lateral_velocity_median	context (ROI/target)	0	0	1
center_lateral_velocity_p10	context (ROI/target)	0	0	1
center_lateral_velocity_p90	context (ROI/target)	0	0	1
center_lateral_velocity_periodicity	context (ROI/target)	0	0	1
center_lateral_velocity_std	context (ROI/target)	0	0	1
center_speed_energy	context (ROI/target)	0	0	1

center_speed_max	context (ROI/target)	0	0	1
center_speed_mean	context (ROI/target)	0	0	1
center_speed_median	context (ROI/target)	0	0	1
center_speed_p10	context (ROI/target)	0	0	1
center_speed_p90	context (ROI/target)	0	0	1
center_speed_periodicity	context (ROI/target)	0	0	1
center_speed_std	context (ROI/target)	0	0	1
center_velocity_x_energy	context (ROI/target)	0	0	1
center_velocity_x_max	context (ROI/target)	0	0	1
center_velocity_x_mean	context (ROI/target)	0	0	1
center_velocity_x_median	context (ROI/target)	0	0	1
center_velocity_x_p10	context (ROI/target)	0	0	1
center_velocity_x_p90	context (ROI/target)	0	0	1
center_velocity_x_periodicity	context (ROI/target)	0	0	1
center_velocity_x_std	context (ROI/target)	0	0	1
center_velocity_y_energy	context (ROI/target)	0	0	1
center_velocity_y_max	context (ROI/target)	0	0	1
center_velocity_y_mean	context (ROI/target)	0	0	1
center_velocity_y_median	context (ROI/target)	0	0	1
center_velocity_y_p10	context (ROI/target)	0	0	1
center_velocity_y_p90	context (ROI/target)	0	0	1
center_velocity_y_periodicity	context (ROI/target)	0	0	1
center_velocity_y_std	context (ROI/target)	0	0	1
centroid_velocity_energy	context (ROI/target)	1	1	1
centroid_velocity_max	context (ROI/target)	1	1	1
centroid_velocity_mean	context (ROI/target)	1	1	1
centroid_velocity_median	context (ROI/target)	1	1	1
centroid_velocity_p10	context (ROI/target)	1	1	1
centroid_velocity_p90	context (ROI/target)	1	1	1
centroid_velocity_periodicity	context (ROI/target)	1	1	1
centroid_velocity_std	context (ROI/target)	1	1	1
density_outlier_score	pose geometry	1	1	1
dist_back_left_paw_to_back_right_paw_delta	kinematics	0	0	0
dist_back_left_paw_to_back_right_paw_energy	kinematics	0	0	0
dist_back_left_paw_to_back_right_paw_max	pose geometry	0	0	0
dist_back_left_paw_to_back_right_paw_mean	pose geometry	0	0	0
dist_back_left_paw_to_back_right_paw_median	pose geometry	0	0	0
dist_back_left_paw_to_back_right_paw_norm_delta	kinematics	0	0	0
dist_back_left_paw_to_back_right_paw_norm_energy	kinematics	0	0	0
dist_back_left_paw_to_back_right_paw_norm_max	pose geometry	0	0	0
dist_back_left_paw_to_back_right_paw_norm_mean	pose geometry	0	0	0
dist_back_left_paw_to_back_right_paw_norm_median	pose geometry	0	0	0
dist_back_left_paw_to_back_right_paw_norm_p10	pose geometry	0	0	0
dist_back_left_paw_to_back_right_paw_norm_p90	pose geometry	0	0	0

dist_back_left_paw_to_back_right_paw_norm_periodic	pose geometry	0	0	0
dist_back_left_paw_to_back_right_paw_norm_std	kinematics	0	0	0
dist_back_left_paw_to_back_right_paw_norm_trend	pose geometry	0	0	0
dist_back_left_paw_to_back_right_paw_p10	pose geometry	0	0	0
dist_back_left_paw_to_back_right_paw_p90	pose geometry	0	0	0
dist_back_left_paw_to_back_right_paw_periodicity	pose geometry	0	0	0
dist_back_left_paw_to_back_right_paw_std	kinematics	0	0	0
dist_back_left_paw_to_back_right_paw_trend	pose geometry	0	0	0
dist_back_left_paw_to_front_left_paw_delta	kinematics	0	0	0
dist_back_left_paw_to_front_left_paw_energy	kinematics	0	0	0
dist_back_left_paw_to_front_left_paw_max	pose geometry	0	0	0
dist_back_left_paw_to_front_left_paw_mean	pose geometry	0	0	0
dist_back_left_paw_to_front_left_paw_median	pose geometry	0	0	0
dist_back_left_paw_to_front_left_paw_norm_delta	kinematics	0	0	0
dist_back_left_paw_to_front_left_paw_norm_energy	kinematics	0	0	0
dist_back_left_paw_to_front_left_paw_norm_max	pose geometry	0	0	0
dist_back_left_paw_to_front_left_paw_norm_mean	pose geometry	0	0	0
dist_back_left_paw_to_front_left_paw_norm_median	pose geometry	0	0	0
dist_back_left_paw_to_front_left_paw_norm_p10	pose geometry	0	0	0
dist_back_left_paw_to_front_left_paw_norm_p90	pose geometry	0	0	0
dist_back_left_paw_to_front_left_paw_norm_periodicit	pose geometry	0	0	0
dist_back_left_paw_to_front_left_paw_norm_std	kinematics	0	0	0
dist_back_left_paw_to_front_left_paw_norm_trend	pose geometry	0	0	0
dist_back_left_paw_to_front_left_paw_p10	pose geometry	0	0	0
dist_back_left_paw_to_front_left_paw_p90	pose geometry	0	0	0
dist_back_left_paw_to_front_left_paw_periodicity	pose geometry	0	0	0
dist_back_left_paw_to_front_left_paw_std	kinematics	0	0	0
dist_back_left_paw_to_front_left_paw_trend	pose geometry	0	0	0
dist_back_left_paw_to_front_right_paw_delta	kinematics	0	0	0
dist_back_left_paw_to_front_right_paw_energy	kinematics	0	0	0
dist_back_left_paw_to_front_right_paw_max	pose geometry	0	0	0
dist_back_left_paw_to_front_right_paw_mean	pose geometry	0	0	0
dist_back_left_paw_to_front_right_paw_median	pose geometry	0	0	0
dist_back_left_paw_to_front_right_paw_norm_delta	kinematics	0	0	0
dist_back_left_paw_to_front_right_paw_norm_energy	kinematics	0	0	0
dist_back_left_paw_to_front_right_paw_norm_max	pose geometry	0	0	0
dist_back_left_paw_to_front_right_paw_norm_mean	pose geometry	0	0	0
dist_back_left_paw_to_front_right_paw_norm_median	pose geometry	0	0	0
dist_back_left_paw_to_front_right_paw_norm_p10	pose geometry	0	0	0
dist_back_left_paw_to_front_right_paw_norm_p90	pose geometry	0	0	0
dist_back_left_paw_to_front_right_paw_norm_periodic	pose geometry	0	0	0
dist_back_left_paw_to_front_right_paw_norm_std	kinematics	0	0	0
dist_back_left_paw_to_front_right_paw_norm_trend	pose geometry	0	0	0
dist_back_left_paw_to_front_right_paw_p10	pose geometry	0	0	0

dist_back_left_paw_to_front_right_paw_p90	pose geometry	0	0	0
dist_back_left_paw_to_front_right_paw_periodicity	pose geometry	0	0	0
dist_back_left_paw_to_front_right_paw_std	kinematics	0	0	0
dist_back_left_paw_to_front_right_paw_trend	pose geometry	0	0	0
dist_back_left_paw_to_left_ear_delta	kinematics	0	0	0
dist_back_left_paw_to_left_ear_energy	kinematics	0	0	0
dist_back_left_paw_to_left_ear_max	pose geometry	0	0	0
dist_back_left_paw_to_left_ear_mean	pose geometry	0	0	0
dist_back_left_paw_to_left_ear_median	pose geometry	0	0	0
dist_back_left_paw_to_left_ear_norm_delta	kinematics	0	0	0
dist_back_left_paw_to_left_ear_norm_energy	kinematics	0	0	0
dist_back_left_paw_to_left_ear_norm_max	pose geometry	0	0	0
dist_back_left_paw_to_left_ear_norm_mean	pose geometry	0	0	0
dist_back_left_paw_to_left_ear_norm_median	pose geometry	0	0	0
dist_back_left_paw_to_left_ear_norm_p10	pose geometry	0	0	0
dist_back_left_paw_to_left_ear_norm_p90	pose geometry	0	0	0
dist_back_left_paw_to_left_ear_norm_periodicity	pose geometry	0	0	0
dist_back_left_paw_to_left_ear_norm_std	kinematics	0	0	0
dist_back_left_paw_to_left_ear_norm_trend	pose geometry	0	0	0
dist_back_left_paw_to_left_ear_p10	pose geometry	0	0	0
dist_back_left_paw_to_left_ear_p90	pose geometry	0	0	0
dist_back_left_paw_to_left_ear_periodicity	pose geometry	0	0	0
dist_back_left_paw_to_left_ear_std	kinematics	0	0	0
dist_back_left_paw_to_left_ear_trend	pose geometry	0	0	0
dist_back_left_paw_to_mid_back_delta	kinematics	0	0	0
dist_back_left_paw_to_mid_back_energy	kinematics	0	0	0
dist_back_left_paw_to_mid_back_max	pose geometry	0	0	0
dist_back_left_paw_to_mid_back_mean	pose geometry	0	0	0
dist_back_left_paw_to_mid_back_median	pose geometry	0	0	0
dist_back_left_paw_to_mid_back_norm_delta	kinematics	0	0	0
dist_back_left_paw_to_mid_back_norm_energy	kinematics	0	0	0
dist_back_left_paw_to_mid_back_norm_max	pose geometry	0	0	0
dist_back_left_paw_to_mid_back_norm_mean	pose geometry	0	0	0
dist_back_left_paw_to_mid_back_norm_median	pose geometry	0	0	0
dist_back_left_paw_to_mid_back_norm_p10	pose geometry	0	0	0
dist_back_left_paw_to_mid_back_norm_p90	pose geometry	0	0	0
dist_back_left_paw_to_mid_back_norm_periodicity	pose geometry	0	0	0
dist_back_left_paw_to_mid_back_norm_std	kinematics	0	0	0
dist_back_left_paw_to_mid_back_norm_trend	pose geometry	0	0	0
dist_back_left_paw_to_mid_back_p10	pose geometry	0	0	0
dist_back_left_paw_to_mid_back_p90	pose geometry	0	0	0
dist_back_left_paw_to_mid_back_periodicity	pose geometry	0	0	0
dist_back_left_paw_to_mid_back_std	kinematics	0	0	0
dist_back_left_paw_to_mid_back_trend	pose geometry	0	0	0

dist_back_left_paw_to_nose_delta	kinematics	0	0	0
dist_back_left_paw_to_nose_energy	kinematics	0	0	0
dist_back_left_paw_to_nose_max	pose geometry	0	0	0
dist_back_left_paw_to_nose_mean	pose geometry	0	0	0
dist_back_left_paw_to_nose_median	pose geometry	0	0	0
dist_back_left_paw_to_nose_norm_delta	kinematics	0	0	0
dist_back_left_paw_to_nose_norm_energy	kinematics	0	0	0
dist_back_left_paw_to_nose_norm_max	pose geometry	0	0	0
dist_back_left_paw_to_nose_norm_mean	pose geometry	0	0	0
dist_back_left_paw_to_nose_norm_median	pose geometry	0	0	0
dist_back_left_paw_to_nose_norm_p10	pose geometry	0	0	0
dist_back_left_paw_to_nose_norm_p90	pose geometry	0	0	0
dist_back_left_paw_to_nose_norm_periodicity	pose geometry	0	0	0
dist_back_left_paw_to_nose_norm_std	kinematics	0	0	0
dist_back_left_paw_to_nose_norm_trend	pose geometry	0	0	0
dist_back_left_paw_to_nose_p10	pose geometry	0	0	0
dist_back_left_paw_to_nose_p90	pose geometry	0	0	0
dist_back_left_paw_to_nose_periodicity	pose geometry	0	0	0
dist_back_left_paw_to_nose_std	kinematics	0	0	0
dist_back_left_paw_to_nose_trend	pose geometry	0	0	0
dist_back_left_paw_to_right_ear_delta	kinematics	0	0	0
dist_back_left_paw_to_right_ear_energy	kinematics	0	0	0
dist_back_left_paw_to_right_ear_max	pose geometry	0	0	0
dist_back_left_paw_to_right_ear_mean	pose geometry	0	0	0
dist_back_left_paw_to_right_ear_median	pose geometry	0	0	0
dist_back_left_paw_to_right_ear_norm_delta	kinematics	0	0	0
dist_back_left_paw_to_right_ear_norm_energy	kinematics	0	0	0
dist_back_left_paw_to_right_ear_norm_max	pose geometry	0	0	0
dist_back_left_paw_to_right_ear_norm_mean	pose geometry	0	0	0
dist_back_left_paw_to_right_ear_norm_median	pose geometry	0	0	0
dist_back_left_paw_to_right_ear_norm_p10	pose geometry	0	0	0
dist_back_left_paw_to_right_ear_norm_p90	pose geometry	0	0	0
dist_back_left_paw_to_right_ear_norm_periodicity	pose geometry	0	0	0
dist_back_left_paw_to_right_ear_norm_std	kinematics	0	0	0
dist_back_left_paw_to_right_ear_norm_trend	pose geometry	0	0	0
dist_back_left_paw_to_right_ear_p10	pose geometry	0	0	0
dist_back_left_paw_to_right_ear_p90	pose geometry	0	0	0
dist_back_left_paw_to_right_ear_periodicity	pose geometry	0	0	0
dist_back_left_paw_to_right_ear_std	kinematics	0	0	0
dist_back_left_paw_to_right_ear_trend	pose geometry	0	0	0
dist_back_left_paw_to_tail_base_delta	kinematics	0	0	0
dist_back_left_paw_to_tail_base_energy	kinematics	0	0	0
dist_back_left_paw_to_tail_base_max	pose geometry	0	0	0
dist_back_left_paw_to_tail_base_mean	pose geometry	0	0	0

dist_back_left_paw_to_tail_base_median	pose geometry	0	0	0
dist_back_left_paw_to_tail_base_norm_delta	kinematics	0	0	0
dist_back_left_paw_to_tail_base_norm_energy	kinematics	0	0	0
dist_back_left_paw_to_tail_base_norm_max	pose geometry	0	0	0
dist_back_left_paw_to_tail_base_norm_mean	pose geometry	0	0	0
dist_back_left_paw_to_tail_base_norm_median	pose geometry	0	0	0
dist_back_left_paw_to_tail_base_norm_p10	pose geometry	0	0	0
dist_back_left_paw_to_tail_base_norm_p90	pose geometry	0	0	0
dist_back_left_paw_to_tail_base_norm_periodicity	pose geometry	0	0	0
dist_back_left_paw_to_tail_base_norm_std	kinematics	0	0	0
dist_back_left_paw_to_tail_base_norm_trend	pose geometry	0	0	0
dist_back_left_paw_to_tail_base_p10	pose geometry	0	0	0
dist_back_left_paw_to_tail_base_p90	pose geometry	0	0	0
dist_back_left_paw_to_tail_base_periodicity	pose geometry	0	0	0
dist_back_left_paw_to_tail_base_std	kinematics	0	0	0
dist_back_left_paw_to_tail_base_trend	pose geometry	0	0	0
dist_back_right_paw_to_front_left_paw_delta	kinematics	0	0	0
dist_back_right_paw_to_front_left_paw_energy	kinematics	0	0	0
dist_back_right_paw_to_front_left_paw_max	pose geometry	0	0	0
dist_back_right_paw_to_front_left_paw_mean	pose geometry	0	0	0
dist_back_right_paw_to_front_left_paw_median	pose geometry	0	0	0
dist_back_right_paw_to_front_left_paw_norm_delta	kinematics	0	0	0
dist_back_right_paw_to_front_left_paw_norm_energy	kinematics	0	0	0
dist_back_right_paw_to_front_left_paw_norm_max	pose geometry	0	0	0
dist_back_right_paw_to_front_left_paw_norm_mean	pose geometry	0	0	0
dist_back_right_paw_to_front_left_paw_norm_median	pose geometry	0	0	0
dist_back_right_paw_to_front_left_paw_norm_p10	pose geometry	0	0	0
dist_back_right_paw_to_front_left_paw_norm_p90	pose geometry	0	0	0
dist_back_right_paw_to_front_left_paw_norm_periodic	pose geometry	0	0	0
dist_back_right_paw_to_front_left_paw_norm_std	kinematics	0	0	0
dist_back_right_paw_to_front_left_paw_norm_trend	pose geometry	0	0	0
dist_back_right_paw_to_front_left_paw_p10	pose geometry	0	0	0
dist_back_right_paw_to_front_left_paw_p90	pose geometry	0	0	0
dist_back_right_paw_to_front_left_paw_periodicity	pose geometry	0	0	0
dist_back_right_paw_to_front_left_paw_std	kinematics	0	0	0
dist_back_right_paw_to_front_left_paw_trend	pose geometry	0	0	0
dist_back_right_paw_to_front_right_paw_delta	kinematics	0	0	0
dist_back_right_paw_to_front_right_paw_energy	kinematics	0	0	0
dist_back_right_paw_to_front_right_paw_max	pose geometry	0	0	0
dist_back_right_paw_to_front_right_paw_mean	pose geometry	0	0	0
dist_back_right_paw_to_front_right_paw_median	pose geometry	0	0	0
dist_back_right_paw_to_front_right_paw_norm_delta	kinematics	0	0	0
dist_back_right_paw_to_front_right_paw_norm_energy	kinematics	0	0	0
dist_back_right_paw_to_front_right_paw_norm_max	pose geometry	0	0	0

dist_back_right_paw_to_front_right_paw_norm_mean	pose geometry	0	0	0
dist_back_right_paw_to_front_right_paw_norm_mediar	pose geometry	0	0	0
dist_back_right_paw_to_front_right_paw_norm_p10	pose geometry	0	0	0
dist_back_right_paw_to_front_right_paw_norm_p90	pose geometry	0	0	0
dist_back_right_paw_to_front_right_paw_norm_periodi	pose geometry	0	0	0
dist_back_right_paw_to_front_right_paw_norm_std	kinematics	0	0	0
dist_back_right_paw_to_front_right_paw_norm_trend	pose geometry	0	0	0
dist_back_right_paw_to_front_right_paw_p10	pose geometry	0	0	0
dist_back_right_paw_to_front_right_paw_p90	pose geometry	0	0	0
dist_back_right_paw_to_front_right_paw_periodicity	pose geometry	0	0	0
dist_back_right_paw_to_front_right_paw_std	kinematics	0	0	0
dist_back_right_paw_to_front_right_paw_trend	pose geometry	0	0	0
dist_back_right_paw_to_left_ear_delta	kinematics	0	0	0
dist_back_right_paw_to_left_ear_energy	kinematics	0	0	0
dist_back_right_paw_to_left_ear_max	pose geometry	0	0	0
dist_back_right_paw_to_left_ear_mean	pose geometry	0	0	0
dist_back_right_paw_to_left_ear_median	pose geometry	0	0	0
dist_back_right_paw_to_left_ear_norm_delta	kinematics	0	0	0
dist_back_right_paw_to_left_ear_norm_energy	kinematics	0	0	0
dist_back_right_paw_to_left_ear_norm_max	pose geometry	0	0	0
dist_back_right_paw_to_left_ear_norm_mean	pose geometry	0	0	0
dist_back_right_paw_to_left_ear_norm_median	pose geometry	0	0	0
dist_back_right_paw_to_left_ear_norm_p10	pose geometry	0	0	0
dist_back_right_paw_to_left_ear_norm_p90	pose geometry	0	0	0
dist_back_right_paw_to_left_ear_norm_periodicity	pose geometry	0	0	0
dist_back_right_paw_to_left_ear_norm_std	kinematics	0	0	0
dist_back_right_paw_to_left_ear_norm_trend	pose geometry	0	0	0
dist_back_right_paw_to_left_ear_p10	pose geometry	0	0	0
dist_back_right_paw_to_left_ear_p90	pose geometry	0	0	0
dist_back_right_paw_to_left_ear_periodicity	pose geometry	0	0	0
dist_back_right_paw_to_left_ear_std	kinematics	0	0	0
dist_back_right_paw_to_left_ear_trend	pose geometry	0	0	0
dist_back_right_paw_to_mid_back_delta	kinematics	0	0	0
dist_back_right_paw_to_mid_back_energy	kinematics	0	0	0
dist_back_right_paw_to_mid_back_max	pose geometry	0	0	0
dist_back_right_paw_to_mid_back_mean	pose geometry	0	0	0
dist_back_right_paw_to_mid_back_median	pose geometry	0	0	0
dist_back_right_paw_to_mid_back_norm_delta	kinematics	0	0	0
dist_back_right_paw_to_mid_back_norm_energy	kinematics	0	0	0
dist_back_right_paw_to_mid_back_norm_max	pose geometry	0	0	0
dist_back_right_paw_to_mid_back_norm_mean	pose geometry	0	0	0
dist_back_right_paw_to_mid_back_norm_median	pose geometry	0	0	0
dist_back_right_paw_to_mid_back_norm_p10	pose geometry	0	0	0
dist_back_right_paw_to_mid_back_norm_p90	pose geometry	0	0	0

dist_back_right_paw_to_mid_back_norm_periodicity	pose geometry	0	0	0
dist_back_right_paw_to_mid_back_norm_std	kinematics	0	0	0
dist_back_right_paw_to_mid_back_norm_trend	pose geometry	0	0	0
dist_back_right_paw_to_mid_back_p10	pose geometry	0	0	0
dist_back_right_paw_to_mid_back_p90	pose geometry	0	0	0
dist_back_right_paw_to_mid_back_periodicity	pose geometry	0	0	0
dist_back_right_paw_to_mid_back_std	kinematics	0	0	0
dist_back_right_paw_to_mid_back_trend	pose geometry	0	0	0
dist_back_right_paw_to_nose_delta	kinematics	0	0	0
dist_back_right_paw_to_nose_energy	kinematics	0	0	0
dist_back_right_paw_to_nose_max	pose geometry	0	0	0
dist_back_right_paw_to_nose_mean	pose geometry	0	0	0
dist_back_right_paw_to_nose_median	pose geometry	0	0	0
dist_back_right_paw_to_nose_norm_delta	kinematics	0	0	0
dist_back_right_paw_to_nose_norm_energy	kinematics	0	0	0
dist_back_right_paw_to_nose_norm_max	pose geometry	0	0	0
dist_back_right_paw_to_nose_norm_mean	pose geometry	0	0	0
dist_back_right_paw_to_nose_norm_median	pose geometry	0	0	0
dist_back_right_paw_to_nose_norm_p10	pose geometry	0	0	0
dist_back_right_paw_to_nose_norm_p90	pose geometry	0	0	0
dist_back_right_paw_to_nose_norm_periodicity	pose geometry	0	0	0
dist_back_right_paw_to_nose_norm_std	kinematics	0	0	0
dist_back_right_paw_to_nose_norm_trend	pose geometry	0	0	0
dist_back_right_paw_to_nose_p10	pose geometry	0	0	0
dist_back_right_paw_to_nose_p90	pose geometry	0	0	0
dist_back_right_paw_to_nose_periodicity	pose geometry	0	0	0
dist_back_right_paw_to_nose_std	kinematics	0	0	0
dist_back_right_paw_to_nose_trend	pose geometry	0	0	0
dist_back_right_paw_to_right_ear_delta	kinematics	0	0	0
dist_back_right_paw_to_right_ear_energy	kinematics	0	0	0
dist_back_right_paw_to_right_ear_max	pose geometry	0	0	0
dist_back_right_paw_to_right_ear_mean	pose geometry	0	0	0
dist_back_right_paw_to_right_ear_median	pose geometry	0	0	0
dist_back_right_paw_to_right_ear_norm_delta	kinematics	0	0	0
dist_back_right_paw_to_right_ear_norm_energy	kinematics	0	0	0
dist_back_right_paw_to_right_ear_norm_max	pose geometry	0	0	0
dist_back_right_paw_to_right_ear_norm_mean	pose geometry	0	0	0
dist_back_right_paw_to_right_ear_norm_median	pose geometry	0	0	0
dist_back_right_paw_to_right_ear_norm_p10	pose geometry	0	0	0
dist_back_right_paw_to_right_ear_norm_p90	pose geometry	0	0	0
dist_back_right_paw_to_right_ear_norm_periodicity	pose geometry	0	0	0
dist_back_right_paw_to_right_ear_norm_std	kinematics	0	0	0
dist_back_right_paw_to_right_ear_norm_trend	pose geometry	0	0	0
dist_back_right_paw_to_right_ear_p10	pose geometry	0	0	0

dist_back_right_paw_to_right_ear_p90	pose geometry	0	0	0
dist_back_right_paw_to_right_ear_periodicity	pose geometry	0	0	0
dist_back_right_paw_to_right_ear_std	kinematics	0	0	0
dist_back_right_paw_to_right_ear_trend	pose geometry	0	0	0
dist_back_right_paw_to_tail_base_delta	kinematics	0	0	0
dist_back_right_paw_to_tail_base_energy	kinematics	0	0	0
dist_back_right_paw_to_tail_base_max	pose geometry	0	0	0
dist_back_right_paw_to_tail_base_mean	pose geometry	0	0	0
dist_back_right_paw_to_tail_base_median	pose geometry	0	0	0
dist_back_right_paw_to_tail_base_norm_delta	kinematics	0	0	0
dist_back_right_paw_to_tail_base_norm_energy	kinematics	0	0	0
dist_back_right_paw_to_tail_base_norm_max	pose geometry	0	0	0
dist_back_right_paw_to_tail_base_norm_mean	pose geometry	0	0	0
dist_back_right_paw_to_tail_base_norm_median	pose geometry	0	0	0
dist_back_right_paw_to_tail_base_norm_p10	pose geometry	0	0	0
dist_back_right_paw_to_tail_base_norm_p90	pose geometry	0	0	0
dist_back_right_paw_to_tail_base_norm_periodicity	pose geometry	0	0	0
dist_back_right_paw_to_tail_base_norm_std	kinematics	0	0	0
dist_back_right_paw_to_tail_base_norm_trend	pose geometry	0	0	0
dist_back_right_paw_to_tail_base_p10	pose geometry	0	0	0
dist_back_right_paw_to_tail_base_p90	pose geometry	0	0	0
dist_back_right_paw_to_tail_base_periodicity	pose geometry	0	0	0
dist_back_right_paw_to_tail_base_std	kinematics	0	0	0
dist_back_right_paw_to_tail_base_trend	pose geometry	0	0	0
dist_body_left_to_body_mid_delta	kinematics	0	1	0
dist_body_left_to_body_mid_energy	kinematics	0	1	0
dist_body_left_to_body_mid_max	pose geometry	0	1	0
dist_body_left_to_body_mid_mean	pose geometry	0	1	0
dist_body_left_to_body_mid_median	pose geometry	0	1	0
dist_body_left_to_body_mid_norm_delta	kinematics	0	1	0
dist_body_left_to_body_mid_norm_energy	kinematics	0	1	0
dist_body_left_to_body_mid_norm_max	pose geometry	0	1	0
dist_body_left_to_body_mid_norm_mean	pose geometry	0	1	0
dist_body_left_to_body_mid_norm_median	pose geometry	0	1	0
dist_body_left_to_body_mid_norm_p10	pose geometry	0	1	0
dist_body_left_to_body_mid_norm_p90	pose geometry	0	1	0
dist_body_left_to_body_mid_norm_periodicity	pose geometry	0	1	0
dist_body_left_to_body_mid_norm_std	kinematics	0	1	0
dist_body_left_to_body_mid_norm_trend	pose geometry	0	1	0
dist_body_left_to_body_mid_p10	pose geometry	0	1	0
dist_body_left_to_body_mid_p90	pose geometry	0	1	0
dist_body_left_to_body_mid_periodicity	pose geometry	0	1	0
dist_body_left_to_body_mid_std	kinematics	0	1	0
dist_body_left_to_body_mid_trend	pose geometry	0	1	0

dist_body_left_to_body_right_delta	kinematics	0	1	0
dist_body_left_to_body_right_energy	kinematics	0	1	0
dist_body_left_to_body_right_max	pose geometry	0	1	0
dist_body_left_to_body_right_mean	pose geometry	0	1	0
dist_body_left_to_body_right_median	pose geometry	0	1	0
dist_body_left_to_body_right_norm_delta	kinematics	0	1	0
dist_body_left_to_body_right_norm_energy	kinematics	0	1	0
dist_body_left_to_body_right_norm_max	pose geometry	0	1	0
dist_body_left_to_body_right_norm_mean	pose geometry	0	1	0
dist_body_left_to_body_right_norm_median	pose geometry	0	1	0
dist_body_left_to_body_right_norm_p10	pose geometry	0	1	0
dist_body_left_to_body_right_norm_p90	pose geometry	0	1	0
dist_body_left_to_body_right_norm_periodicity	pose geometry	0	1	0
dist_body_left_to_body_right_norm_std	kinematics	0	1	0
dist_body_left_to_body_right_norm_trend	pose geometry	0	1	0
dist_body_left_to_body_right_p10	pose geometry	0	1	0
dist_body_left_to_body_right_p90	pose geometry	0	1	0
dist_body_left_to_body_right_periodicity	pose geometry	0	1	0
dist_body_left_to_body_right_std	kinematics	0	1	0
dist_body_left_to_body_right_trend	pose geometry	0	1	0
dist_body_left_to_ear_left_delta	kinematics	0	1	0
dist_body_left_to_ear_left_energy	kinematics	0	1	0
dist_body_left_to_ear_left_max	pose geometry	0	1	0
dist_body_left_to_ear_left_mean	pose geometry	0	1	0
dist_body_left_to_ear_left_median	pose geometry	0	1	0
dist_body_left_to_ear_left_norm_delta	kinematics	0	1	0
dist_body_left_to_ear_left_norm_energy	kinematics	0	1	0
dist_body_left_to_ear_left_norm_max	pose geometry	0	1	0
dist_body_left_to_ear_left_norm_mean	pose geometry	0	1	0
dist_body_left_to_ear_left_norm_median	pose geometry	0	1	0
dist_body_left_to_ear_left_norm_p10	pose geometry	0	1	0
dist_body_left_to_ear_left_norm_p90	pose geometry	0	1	0
dist_body_left_to_ear_left_norm_periodicity	pose geometry	0	1	0
dist_body_left_to_ear_left_norm_std	kinematics	0	1	0
dist_body_left_to_ear_left_norm_trend	pose geometry	0	1	0
dist_body_left_to_ear_left_p10	pose geometry	0	1	0
dist_body_left_to_ear_left_p90	pose geometry	0	1	0
dist_body_left_to_ear_left_periodicity	pose geometry	0	1	0
dist_body_left_to_ear_left_std	kinematics	0	1	0
dist_body_left_to_ear_left_trend	pose geometry	0	1	0
dist_body_left_to_ear_right_delta	kinematics	0	1	0
dist_body_left_to_ear_right_energy	kinematics	0	1	0
dist_body_left_to_ear_right_max	pose geometry	0	1	0
dist_body_left_to_ear_right_mean	pose geometry	0	1	0

dist_body_left_to_ear_right_median	pose geometry	0	1	0
dist_body_left_to_ear_right_norm_delta	kinematics	0	1	0
dist_body_left_to_ear_right_norm_energy	kinematics	0	1	0
dist_body_left_to_ear_right_norm_max	pose geometry	0	1	0
dist_body_left_to_ear_right_norm_mean	pose geometry	0	1	0
dist_body_left_to_ear_right_norm_median	pose geometry	0	1	0
dist_body_left_to_ear_right_norm_p10	pose geometry	0	1	0
dist_body_left_to_ear_right_norm_p90	pose geometry	0	1	0
dist_body_left_to_ear_right_norm_periodicity	pose geometry	0	1	0
dist_body_left_to_ear_right_norm_std	kinematics	0	1	0
dist_body_left_to_ear_right_norm_trend	pose geometry	0	1	0
dist_body_left_to_ear_right_p10	pose geometry	0	1	0
dist_body_left_to_ear_right_p90	pose geometry	0	1	0
dist_body_left_to_ear_right_periodicity	pose geometry	0	1	0
dist_body_left_to_ear_right_std	kinematics	0	1	0
dist_body_left_to_ear_right_trend	pose geometry	0	1	0
dist_body_left_to_nose_delta	kinematics	0	1	0
dist_body_left_to_nose_energy	kinematics	0	1	0
dist_body_left_to_nose_max	pose geometry	0	1	0
dist_body_left_to_nose_mean	pose geometry	0	1	0
dist_body_left_to_nose_median	pose geometry	0	1	0
dist_body_left_to_nose_norm_delta	kinematics	0	1	0
dist_body_left_to_nose_norm_energy	kinematics	0	1	0
dist_body_left_to_nose_norm_max	pose geometry	0	1	0
dist_body_left_to_nose_norm_mean	pose geometry	0	1	0
dist_body_left_to_nose_norm_median	pose geometry	0	1	0
dist_body_left_to_nose_norm_p10	pose geometry	0	1	0
dist_body_left_to_nose_norm_p90	pose geometry	0	1	0
dist_body_left_to_nose_norm_periodicity	pose geometry	0	1	0
dist_body_left_to_nose_norm_std	kinematics	0	1	0
dist_body_left_to_nose_norm_trend	pose geometry	0	1	0
dist_body_left_to_nose_p10	pose geometry	0	1	0
dist_body_left_to_nose_p90	pose geometry	0	1	0
dist_body_left_to_nose_periodicity	pose geometry	0	1	0
dist_body_left_to_nose_std	kinematics	0	1	0
dist_body_left_to_nose_trend	pose geometry	0	1	0
dist_body_left_to_tail_base_delta	kinematics	0	1	0
dist_body_left_to_tail_base_energy	kinematics	0	1	0
dist_body_left_to_tail_base_max	pose geometry	0	1	0
dist_body_left_to_tail_base_mean	pose geometry	0	1	0
dist_body_left_to_tail_base_median	pose geometry	0	1	0
dist_body_left_to_tail_base_norm_delta	kinematics	0	1	0
dist_body_left_to_tail_base_norm_energy	kinematics	0	1	0
dist_body_left_to_tail_base_norm_max	pose geometry	0	1	0

dist_body_left_to_tail_base_norm_mean	pose geometry	0	1	0
dist_body_left_to_tail_base_norm_median	pose geometry	0	1	0
dist_body_left_to_tail_base_norm_p10	pose geometry	0	1	0
dist_body_left_to_tail_base_norm_p90	pose geometry	0	1	0
dist_body_left_to_tail_base_norm_periodicity	pose geometry	0	1	0
dist_body_left_to_tail_base_norm_std	kinematics	0	1	0
dist_body_left_to_tail_base_norm_trend	pose geometry	0	1	0
dist_body_left_to_tail_base_p10	pose geometry	0	1	0
dist_body_left_to_tail_base_p90	pose geometry	0	1	0
dist_body_left_to_tail_base_periodicity	pose geometry	0	1	0
dist_body_left_to_tail_base_std	kinematics	0	1	0
dist_body_left_to_tail_base_trend	pose geometry	0	1	0
dist_body_mid_to_body_right_delta	kinematics	0	1	0
dist_body_mid_to_body_right_energy	kinematics	0	1	0
dist_body_mid_to_body_right_max	pose geometry	0	1	0
dist_body_mid_to_body_right_mean	pose geometry	0	1	0
dist_body_mid_to_body_right_median	pose geometry	0	1	0
dist_body_mid_to_body_right_norm_delta	kinematics	0	1	0
dist_body_mid_to_body_right_norm_energy	kinematics	0	1	0
dist_body_mid_to_body_right_norm_max	pose geometry	0	1	0
dist_body_mid_to_body_right_norm_mean	pose geometry	0	1	0
dist_body_mid_to_body_right_norm_median	pose geometry	0	1	0
dist_body_mid_to_body_right_norm_p10	pose geometry	0	1	0
dist_body_mid_to_body_right_norm_p90	pose geometry	0	1	0
dist_body_mid_to_body_right_norm_periodicity	pose geometry	0	1	0
dist_body_mid_to_body_right_norm_std	kinematics	0	1	0
dist_body_mid_to_body_right_norm_trend	pose geometry	0	1	0
dist_body_mid_to_body_right_p10	pose geometry	0	1	0
dist_body_mid_to_body_right_p90	pose geometry	0	1	0
dist_body_mid_to_body_right_periodicity	pose geometry	0	1	0
dist_body_mid_to_body_right_std	kinematics	0	1	0
dist_body_mid_to_body_right_trend	pose geometry	0	1	0
dist_body_mid_to_ear_left_delta	kinematics	0	1	0
dist_body_mid_to_ear_left_energy	kinematics	0	1	0
dist_body_mid_to_ear_left_max	pose geometry	0	1	0
dist_body_mid_to_ear_left_mean	pose geometry	0	1	0
dist_body_mid_to_ear_left_median	pose geometry	0	1	0
dist_body_mid_to_ear_left_norm_delta	kinematics	0	1	0
dist_body_mid_to_ear_left_norm_energy	kinematics	0	1	0
dist_body_mid_to_ear_left_norm_max	pose geometry	0	1	0
dist_body_mid_to_ear_left_norm_mean	pose geometry	0	1	0
dist_body_mid_to_ear_left_norm_median	pose geometry	0	1	0
dist_body_mid_to_ear_left_norm_p10	pose geometry	0	1	0
dist_body_mid_to_ear_left_norm_p90	pose geometry	0	1	0

dist_body_mid_to_ear_left_norm_periodicity	pose geometry	0	1	0
dist_body_mid_to_ear_left_norm_std	kinematics	0	1	0
dist_body_mid_to_ear_left_norm_trend	pose geometry	0	1	0
dist_body_mid_to_ear_left_p10	pose geometry	0	1	0
dist_body_mid_to_ear_left_p90	pose geometry	0	1	0
dist_body_mid_to_ear_left_periodicity	pose geometry	0	1	0
dist_body_mid_to_ear_left_std	kinematics	0	1	0
dist_body_mid_to_ear_left_trend	pose geometry	0	1	0
dist_body_mid_to_ear_right_delta	kinematics	0	1	0
dist_body_mid_to_ear_right_energy	kinematics	0	1	0
dist_body_mid_to_ear_right_max	pose geometry	0	1	0
dist_body_mid_to_ear_right_mean	pose geometry	0	1	0
dist_body_mid_to_ear_right_median	pose geometry	0	1	0
dist_body_mid_to_ear_right_norm_delta	kinematics	0	1	0
dist_body_mid_to_ear_right_norm_energy	kinematics	0	1	0
dist_body_mid_to_ear_right_norm_max	pose geometry	0	1	0
dist_body_mid_to_ear_right_norm_mean	pose geometry	0	1	0
dist_body_mid_to_ear_right_norm_median	pose geometry	0	1	0
dist_body_mid_to_ear_right_norm_p10	pose geometry	0	1	0
dist_body_mid_to_ear_right_norm_p90	pose geometry	0	1	0
dist_body_mid_to_ear_right_norm_periodicity	pose geometry	0	1	0
dist_body_mid_to_ear_right_norm_std	kinematics	0	1	0
dist_body_mid_to_ear_right_norm_trend	pose geometry	0	1	0
dist_body_mid_to_ear_right_p10	pose geometry	0	1	0
dist_body_mid_to_ear_right_p90	pose geometry	0	1	0
dist_body_mid_to_ear_right_periodicity	pose geometry	0	1	0
dist_body_mid_to_ear_right_std	kinematics	0	1	0
dist_body_mid_to_ear_right_trend	pose geometry	0	1	0
dist_body_mid_to_nose_delta	kinematics	0	1	0
dist_body_mid_to_nose_energy	kinematics	0	1	0
dist_body_mid_to_nose_max	pose geometry	0	1	0
dist_body_mid_to_nose_mean	pose geometry	0	1	0
dist_body_mid_to_nose_median	pose geometry	0	1	0
dist_body_mid_to_nose_norm_delta	kinematics	0	1	0
dist_body_mid_to_nose_norm_energy	kinematics	0	1	0
dist_body_mid_to_nose_norm_max	pose geometry	0	1	0
dist_body_mid_to_nose_norm_mean	pose geometry	0	1	0
dist_body_mid_to_nose_norm_median	pose geometry	0	1	0
dist_body_mid_to_nose_norm_p10	pose geometry	0	1	0
dist_body_mid_to_nose_norm_p90	pose geometry	0	1	0
dist_body_mid_to_nose_norm_periodicity	pose geometry	0	1	0
dist_body_mid_to_nose_norm_std	kinematics	0	1	0
dist_body_mid_to_nose_norm_trend	pose geometry	0	1	0
dist_body_mid_to_nose_p10	pose geometry	0	1	0

dist_body_mid_to_nose_p90	pose geometry	0	1	0
dist_body_mid_to_nose_periodicity	pose geometry	0	1	0
dist_body_mid_to_nose_std	kinematics	0	1	0
dist_body_mid_to_nose_trend	pose geometry	0	1	0
dist_body_mid_to_tail_base_delta	kinematics	0	1	0
dist_body_mid_to_tail_base_energy	kinematics	0	1	0
dist_body_mid_to_tail_base_max	pose geometry	0	1	0
dist_body_mid_to_tail_base_mean	pose geometry	0	1	0
dist_body_mid_to_tail_base_median	pose geometry	0	1	0
dist_body_mid_to_tail_base_norm_delta	kinematics	0	1	0
dist_body_mid_to_tail_base_norm_energy	kinematics	0	1	0
dist_body_mid_to_tail_base_norm_max	pose geometry	0	1	0
dist_body_mid_to_tail_base_norm_mean	pose geometry	0	1	0
dist_body_mid_to_tail_base_norm_median	pose geometry	0	1	0
dist_body_mid_to_tail_base_norm_p10	pose geometry	0	1	0
dist_body_mid_to_tail_base_norm_p90	pose geometry	0	1	0
dist_body_mid_to_tail_base_norm_periodicity	pose geometry	0	1	0
dist_body_mid_to_tail_base_norm_std	kinematics	0	1	0
dist_body_mid_to_tail_base_norm_trend	pose geometry	0	1	0
dist_body_mid_to_tail_base_p10	pose geometry	0	1	0
dist_body_mid_to_tail_base_p90	pose geometry	0	1	0
dist_body_mid_to_tail_base_periodicity	pose geometry	0	1	0
dist_body_mid_to_tail_base_std	kinematics	0	1	0
dist_body_mid_to_tail_base_trend	pose geometry	0	1	0
dist_body_right_to_ear_left_delta	kinematics	0	1	0
dist_body_right_to_ear_left_energy	kinematics	0	1	0
dist_body_right_to_ear_left_max	pose geometry	0	1	0
dist_body_right_to_ear_left_mean	pose geometry	0	1	0
dist_body_right_to_ear_left_median	pose geometry	0	1	0
dist_body_right_to_ear_left_norm_delta	kinematics	0	1	0
dist_body_right_to_ear_left_norm_energy	kinematics	0	1	0
dist_body_right_to_ear_left_norm_max	pose geometry	0	1	0
dist_body_right_to_ear_left_norm_mean	pose geometry	0	1	0
dist_body_right_to_ear_left_norm_median	pose geometry	0	1	0
dist_body_right_to_ear_left_norm_p10	pose geometry	0	1	0
dist_body_right_to_ear_left_norm_p90	pose geometry	0	1	0
dist_body_right_to_ear_left_norm_periodicity	pose geometry	0	1	0
dist_body_right_to_ear_left_norm_std	kinematics	0	1	0
dist_body_right_to_ear_left_norm_trend	pose geometry	0	1	0
dist_body_right_to_ear_left_p10	pose geometry	0	1	0
dist_body_right_to_ear_left_p90	pose geometry	0	1	0
dist_body_right_to_ear_left_periodicity	pose geometry	0	1	0
dist_body_right_to_ear_left_std	kinematics	0	1	0
dist_body_right_to_ear_left_trend	pose geometry	0	1	0

dist_body_right_to_ear_right_delta	kinematics	0	1	0
dist_body_right_to_ear_right_energy	kinematics	0	1	0
dist_body_right_to_ear_right_max	pose geometry	0	1	0
dist_body_right_to_ear_right_mean	pose geometry	0	1	0
dist_body_right_to_ear_right_median	pose geometry	0	1	0
dist_body_right_to_ear_right_norm_delta	kinematics	0	1	0
dist_body_right_to_ear_right_norm_energy	kinematics	0	1	0
dist_body_right_to_ear_right_norm_max	pose geometry	0	1	0
dist_body_right_to_ear_right_norm_mean	pose geometry	0	1	0
dist_body_right_to_ear_right_norm_median	pose geometry	0	1	0
dist_body_right_to_ear_right_norm_p10	pose geometry	0	1	0
dist_body_right_to_ear_right_norm_p90	pose geometry	0	1	0
dist_body_right_to_ear_right_norm_periodicity	pose geometry	0	1	0
dist_body_right_to_ear_right_norm_std	kinematics	0	1	0
dist_body_right_to_ear_right_norm_trend	pose geometry	0	1	0
dist_body_right_to_ear_right_p10	pose geometry	0	1	0
dist_body_right_to_ear_right_p90	pose geometry	0	1	0
dist_body_right_to_ear_right_periodicity	pose geometry	0	1	0
dist_body_right_to_ear_right_std	kinematics	0	1	0
dist_body_right_to_ear_right_trend	pose geometry	0	1	0
dist_body_right_to_nose_delta	kinematics	0	1	0
dist_body_right_to_nose_energy	kinematics	0	1	0
dist_body_right_to_nose_max	pose geometry	0	1	0
dist_body_right_to_nose_mean	pose geometry	0	1	0
dist_body_right_to_nose_median	pose geometry	0	1	0
dist_body_right_to_nose_norm_delta	kinematics	0	1	0
dist_body_right_to_nose_norm_energy	kinematics	0	1	0
dist_body_right_to_nose_norm_max	pose geometry	0	1	0
dist_body_right_to_nose_norm_mean	pose geometry	0	1	0
dist_body_right_to_nose_norm_median	pose geometry	0	1	0
dist_body_right_to_nose_norm_p10	pose geometry	0	1	0
dist_body_right_to_nose_norm_p90	pose geometry	0	1	0
dist_body_right_to_nose_norm_periodicity	pose geometry	0	1	0
dist_body_right_to_nose_norm_std	kinematics	0	1	0
dist_body_right_to_nose_norm_trend	pose geometry	0	1	0
dist_body_right_to_nose_p10	pose geometry	0	1	0
dist_body_right_to_nose_p90	pose geometry	0	1	0
dist_body_right_to_nose_periodicity	pose geometry	0	1	0
dist_body_right_to_nose_std	kinematics	0	1	0
dist_body_right_to_nose_trend	pose geometry	0	1	0
dist_body_right_to_tail_base_delta	kinematics	0	1	0
dist_body_right_to_tail_base_energy	kinematics	0	1	0
dist_body_right_to_tail_base_max	pose geometry	0	1	0
dist_body_right_to_tail_base_mean	pose geometry	0	1	0

dist_body_right_to_tail_base_median	pose geometry	0	1	0
dist_body_right_to_tail_base_norm_delta	kinematics	0	1	0
dist_body_right_to_tail_base_norm_energy	kinematics	0	1	0
dist_body_right_to_tail_base_norm_max	pose geometry	0	1	0
dist_body_right_to_tail_base_norm_mean	pose geometry	0	1	0
dist_body_right_to_tail_base_norm_median	pose geometry	0	1	0
dist_body_right_to_tail_base_norm_p10	pose geometry	0	1	0
dist_body_right_to_tail_base_norm_p90	pose geometry	0	1	0
dist_body_right_to_tail_base_norm_periodicity	pose geometry	0	1	0
dist_body_right_to_tail_base_norm_std	kinematics	0	1	0
dist_body_right_to_tail_base_norm_trend	pose geometry	0	1	0
dist_body_right_to_tail_base_p10	pose geometry	0	1	0
dist_body_right_to_tail_base_p90	pose geometry	0	1	0
dist_body_right_to_tail_base_periodicity	pose geometry	0	1	0
dist_body_right_to_tail_base_std	kinematics	0	1	0
dist_body_right_to_tail_base_trend	pose geometry	0	1	0
dist_center_body_to_left_body_delta	context (ROI/target)	1	0	0
dist_center_body_to_left_body_energy	context (ROI/target)	1	0	0
dist_center_body_to_left_body_max	context (ROI/target)	1	0	0
dist_center_body_to_left_body_mean	context (ROI/target)	1	0	0
dist_center_body_to_left_body_median	context (ROI/target)	1	0	0
dist_center_body_to_left_body_norm_delta	context (ROI/target)	1	0	0
dist_center_body_to_left_body_norm_energy	context (ROI/target)	1	0	0
dist_center_body_to_left_body_norm_max	context (ROI/target)	1	0	0
dist_center_body_to_left_body_norm_mean	context (ROI/target)	1	0	0
dist_center_body_to_left_body_norm_median	context (ROI/target)	1	0	0
dist_center_body_to_left_body_norm_p10	context (ROI/target)	1	0	0
dist_center_body_to_left_body_norm_p90	context (ROI/target)	1	0	0
dist_center_body_to_left_body_norm_periodicity	context (ROI/target)	1	0	0
dist_center_body_to_left_body_norm_std	context (ROI/target)	1	0	0
dist_center_body_to_left_body_norm_trend	context (ROI/target)	1	0	0
dist_center_body_to_left_body_p10	context (ROI/target)	1	0	0
dist_center_body_to_left_body_p90	context (ROI/target)	1	0	0
dist_center_body_to_left_body_periodicity	context (ROI/target)	1	0	0
dist_center_body_to_left_body_std	context (ROI/target)	1	0	0
dist_center_body_to_left_body_trend	context (ROI/target)	1	0	0
dist_center_body_to_left_ear_delta	context (ROI/target)	1	0	0
dist_center_body_to_left_ear_energy	context (ROI/target)	1	0	0
dist_center_body_to_left_ear_max	context (ROI/target)	1	0	0
dist_center_body_to_left_ear_mean	context (ROI/target)	1	0	0
dist_center_body_to_left_ear_median	context (ROI/target)	1	0	0
dist_center_body_to_left_ear_norm_delta	context (ROI/target)	1	0	0
dist_center_body_to_left_ear_norm_energy	context (ROI/target)	1	0	0
dist_center_body_to_left_ear_norm_max	context (ROI/target)	1	0	0

dist_center_body_to_left_ear_norm_mean	context (ROI/target)	1	0	0
dist_center_body_to_left_ear_norm_median	context (ROI/target)	1	0	0
dist_center_body_to_left_ear_norm_p10	context (ROI/target)	1	0	0
dist_center_body_to_left_ear_norm_p90	context (ROI/target)	1	0	0
dist_center_body_to_left_ear_norm_periodicity	context (ROI/target)	1	0	0
dist_center_body_to_left_ear_norm_std	context (ROI/target)	1	0	0
dist_center_body_to_left_ear_norm_trend	context (ROI/target)	1	0	0
dist_center_body_to_left_ear_p10	context (ROI/target)	1	0	0
dist_center_body_to_left_ear_p90	context (ROI/target)	1	0	0
dist_center_body_to_left_ear_periodicity	context (ROI/target)	1	0	0
dist_center_body_to_left_ear_std	context (ROI/target)	1	0	0
dist_center_body_to_left_ear_trend	context (ROI/target)	1	0	0
dist_center_body_to_nose_delta	context (ROI/target)	1	0	0
dist_center_body_to_nose_energy	context (ROI/target)	1	0	0
dist_center_body_to_nose_max	context (ROI/target)	1	0	0
dist_center_body_to_nose_mean	context (ROI/target)	1	0	0
dist_center_body_to_nose_median	context (ROI/target)	1	0	0
dist_center_body_to_nose_norm_delta	context (ROI/target)	1	0	0
dist_center_body_to_nose_norm_energy	context (ROI/target)	1	0	0
dist_center_body_to_nose_norm_max	context (ROI/target)	1	0	0
dist_center_body_to_nose_norm_mean	context (ROI/target)	1	0	0
dist_center_body_to_nose_norm_median	context (ROI/target)	1	0	0
dist_center_body_to_nose_norm_p10	context (ROI/target)	1	0	0
dist_center_body_to_nose_norm_p90	context (ROI/target)	1	0	0
dist_center_body_to_nose_norm_periodicity	context (ROI/target)	1	0	0
dist_center_body_to_nose_norm_std	context (ROI/target)	1	0	0
dist_center_body_to_nose_norm_trend	context (ROI/target)	1	0	0
dist_center_body_to_nose_p10	context (ROI/target)	1	0	0
dist_center_body_to_nose_p90	context (ROI/target)	1	0	0
dist_center_body_to_nose_periodicity	context (ROI/target)	1	0	0
dist_center_body_to_nose_std	context (ROI/target)	1	0	0
dist_center_body_to_nose_trend	context (ROI/target)	1	0	0
dist_center_body_to_right_body_delta	context (ROI/target)	1	0	0
dist_center_body_to_right_body_energy	context (ROI/target)	1	0	0
dist_center_body_to_right_body_max	context (ROI/target)	1	0	0
dist_center_body_to_right_body_mean	context (ROI/target)	1	0	0
dist_center_body_to_right_body_median	context (ROI/target)	1	0	0
dist_center_body_to_right_body_norm_delta	context (ROI/target)	1	0	0
dist_center_body_to_right_body_norm_energy	context (ROI/target)	1	0	0
dist_center_body_to_right_body_norm_max	context (ROI/target)	1	0	0
dist_center_body_to_right_body_norm_mean	context (ROI/target)	1	0	0
dist_center_body_to_right_body_norm_median	context (ROI/target)	1	0	0
dist_center_body_to_right_body_norm_p10	context (ROI/target)	1	0	0
dist_center_body_to_right_body_norm_p90	context (ROI/target)	1	0	0

dist_center_body_to_right_body_norm_periodicity	context (ROI/target)	1	0	0
dist_center_body_to_right_body_norm_std	context (ROI/target)	1	0	0
dist_center_body_to_right_body_norm_trend	context (ROI/target)	1	0	0
dist_center_body_to_right_body_p10	context (ROI/target)	1	0	0
dist_center_body_to_right_body_p90	context (ROI/target)	1	0	0
dist_center_body_to_right_body_periodicity	context (ROI/target)	1	0	0
dist_center_body_to_right_body_std	context (ROI/target)	1	0	0
dist_center_body_to_right_body_trend	context (ROI/target)	1	0	0
dist_center_body_to_right_ear_delta	context (ROI/target)	1	0	0
dist_center_body_to_right_ear_energy	context (ROI/target)	1	0	0
dist_center_body_to_right_ear_max	context (ROI/target)	1	0	0
dist_center_body_to_right_ear_mean	context (ROI/target)	1	0	0
dist_center_body_to_right_ear_median	context (ROI/target)	1	0	0
dist_center_body_to_right_ear_norm_delta	context (ROI/target)	1	0	0
dist_center_body_to_right_ear_norm_energy	context (ROI/target)	1	0	0
dist_center_body_to_right_ear_norm_max	context (ROI/target)	1	0	0
dist_center_body_to_right_ear_norm_mean	context (ROI/target)	1	0	0
dist_center_body_to_right_ear_norm_median	context (ROI/target)	1	0	0
dist_center_body_to_right_ear_norm_p10	context (ROI/target)	1	0	0
dist_center_body_to_right_ear_norm_p90	context (ROI/target)	1	0	0
dist_center_body_to_right_ear_norm_periodicity	context (ROI/target)	1	0	0
dist_center_body_to_right_ear_norm_std	context (ROI/target)	1	0	0
dist_center_body_to_right_ear_norm_trend	context (ROI/target)	1	0	0
dist_center_body_to_right_ear_p10	context (ROI/target)	1	0	0
dist_center_body_to_right_ear_p90	context (ROI/target)	1	0	0
dist_center_body_to_right_ear_periodicity	context (ROI/target)	1	0	0
dist_center_body_to_right_ear_std	context (ROI/target)	1	0	0
dist_center_body_to_right_ear_trend	context (ROI/target)	1	0	0
dist_center_body_to_tail_base_delta	context (ROI/target)	1	0	0
dist_center_body_to_tail_base_energy	context (ROI/target)	1	0	0
dist_center_body_to_tail_base_max	context (ROI/target)	1	0	0
dist_center_body_to_tail_base_mean	context (ROI/target)	1	0	0
dist_center_body_to_tail_base_median	context (ROI/target)	1	0	0
dist_center_body_to_tail_base_norm_delta	context (ROI/target)	1	0	0
dist_center_body_to_tail_base_norm_energy	context (ROI/target)	1	0	0
dist_center_body_to_tail_base_norm_max	context (ROI/target)	1	0	0
dist_center_body_to_tail_base_norm_mean	context (ROI/target)	1	0	0
dist_center_body_to_tail_base_norm_median	context (ROI/target)	1	0	0
dist_center_body_to_tail_base_norm_p10	context (ROI/target)	1	0	0
dist_center_body_to_tail_base_norm_p90	context (ROI/target)	1	0	0
dist_center_body_to_tail_base_norm_periodicity	context (ROI/target)	1	0	0
dist_center_body_to_tail_base_norm_std	context (ROI/target)	1	0	0
dist_center_body_to_tail_base_norm_trend	context (ROI/target)	1	0	0
dist_center_body_to_tail_base_p10	context (ROI/target)	1	0	0

dist_center_body_to_tail_base_p90	context (ROI/target)	1	0	0
dist_center_body_to_tail_base_periodicity	context (ROI/target)	1	0	0
dist_center_body_to_tail_base_std	context (ROI/target)	1	0	0
dist_center_body_to_tail_base_trend	context (ROI/target)	1	0	0
dist_center_to_ear_left_delta	context (ROI/target)	0	0	1
dist_center_to_ear_left_energy	context (ROI/target)	0	0	1
dist_center_to_ear_left_max	context (ROI/target)	0	0	1
dist_center_to_ear_left_mean	context (ROI/target)	0	0	1
dist_center_to_ear_left_median	context (ROI/target)	0	0	1
dist_center_to_ear_left_norm_delta	context (ROI/target)	0	0	1
dist_center_to_ear_left_norm_energy	context (ROI/target)	0	0	1
dist_center_to_ear_left_norm_max	context (ROI/target)	0	0	1
dist_center_to_ear_left_norm_mean	context (ROI/target)	0	0	1
dist_center_to_ear_left_norm_median	context (ROI/target)	0	0	1
dist_center_to_ear_left_norm_p10	context (ROI/target)	0	0	1
dist_center_to_ear_left_norm_p90	context (ROI/target)	0	0	1
dist_center_to_ear_left_norm_periodicity	context (ROI/target)	0	0	1
dist_center_to_ear_left_norm_std	context (ROI/target)	0	0	1
dist_center_to_ear_left_norm_trend	context (ROI/target)	0	0	1
dist_center_to_ear_left_p10	context (ROI/target)	0	0	1
dist_center_to_ear_left_p90	context (ROI/target)	0	0	1
dist_center_to_ear_left_periodicity	context (ROI/target)	0	0	1
dist_center_to_ear_left_std	context (ROI/target)	0	0	1
dist_center_to_ear_left_trend	context (ROI/target)	0	0	1
dist_center_to_ear_right_delta	context (ROI/target)	0	0	1
dist_center_to_ear_right_energy	context (ROI/target)	0	0	1
dist_center_to_ear_right_max	context (ROI/target)	0	0	1
dist_center_to_ear_right_mean	context (ROI/target)	0	0	1
dist_center_to_ear_right_median	context (ROI/target)	0	0	1
dist_center_to_ear_right_norm_delta	context (ROI/target)	0	0	1
dist_center_to_ear_right_norm_energy	context (ROI/target)	0	0	1
dist_center_to_ear_right_norm_max	context (ROI/target)	0	0	1
dist_center_to_ear_right_norm_mean	context (ROI/target)	0	0	1
dist_center_to_ear_right_norm_median	context (ROI/target)	0	0	1
dist_center_to_ear_right_norm_p10	context (ROI/target)	0	0	1
dist_center_to_ear_right_norm_p90	context (ROI/target)	0	0	1
dist_center_to_ear_right_norm_periodicity	context (ROI/target)	0	0	1
dist_center_to_ear_right_norm_std	context (ROI/target)	0	0	1
dist_center_to_ear_right_norm_trend	context (ROI/target)	0	0	1
dist_center_to_ear_right_p10	context (ROI/target)	0	0	1
dist_center_to_ear_right_p90	context (ROI/target)	0	0	1
dist_center_to_ear_right_periodicity	context (ROI/target)	0	0	1
dist_center_to_ear_right_std	context (ROI/target)	0	0	1
dist_center_to_ear_right_trend	context (ROI/target)	0	0	1

dist_center_to_lateral_left_delta	context (ROI/target)	0	0	1
dist_center_to_lateral_left_energy	context (ROI/target)	0	0	1
dist_center_to_lateral_left_max	context (ROI/target)	0	0	1
dist_center_to_lateral_left_mean	context (ROI/target)	0	0	1
dist_center_to_lateral_left_median	context (ROI/target)	0	0	1
dist_center_to_lateral_left_norm_delta	context (ROI/target)	0	0	1
dist_center_to_lateral_left_norm_energy	context (ROI/target)	0	0	1
dist_center_to_lateral_left_norm_max	context (ROI/target)	0	0	1
dist_center_to_lateral_left_norm_mean	context (ROI/target)	0	0	1
dist_center_to_lateral_left_norm_median	context (ROI/target)	0	0	1
dist_center_to_lateral_left_norm_p10	context (ROI/target)	0	0	1
dist_center_to_lateral_left_norm_p90	context (ROI/target)	0	0	1
dist_center_to_lateral_left_norm_periodicity	context (ROI/target)	0	0	1
dist_center_to_lateral_left_norm_std	context (ROI/target)	0	0	1
dist_center_to_lateral_left_norm_trend	context (ROI/target)	0	0	1
dist_center_to_lateral_left_p10	context (ROI/target)	0	0	1
dist_center_to_lateral_left_p90	context (ROI/target)	0	0	1
dist_center_to_lateral_left_periodicity	context (ROI/target)	0	0	1
dist_center_to_lateral_left_std	context (ROI/target)	0	0	1
dist_center_to_lateral_left_trend	context (ROI/target)	0	0	1
dist_center_to_lateral_right_delta	context (ROI/target)	0	0	1
dist_center_to_lateral_right_energy	context (ROI/target)	0	0	1
dist_center_to_lateral_right_max	context (ROI/target)	0	0	1
dist_center_to_lateral_right_mean	context (ROI/target)	0	0	1
dist_center_to_lateral_right_median	context (ROI/target)	0	0	1
dist_center_to_lateral_right_norm_delta	context (ROI/target)	0	0	1
dist_center_to_lateral_right_norm_energy	context (ROI/target)	0	0	1
dist_center_to_lateral_right_norm_max	context (ROI/target)	0	0	1
dist_center_to_lateral_right_norm_mean	context (ROI/target)	0	0	1
dist_center_to_lateral_right_norm_median	context (ROI/target)	0	0	1
dist_center_to_lateral_right_norm_p10	context (ROI/target)	0	0	1
dist_center_to_lateral_right_norm_p90	context (ROI/target)	0	0	1
dist_center_to_lateral_right_norm_periodicity	context (ROI/target)	0	0	1
dist_center_to_lateral_right_norm_std	context (ROI/target)	0	0	1
dist_center_to_lateral_right_norm_trend	context (ROI/target)	0	0	1
dist_center_to_lateral_right_p10	context (ROI/target)	0	0	1
dist_center_to_lateral_right_p90	context (ROI/target)	0	0	1
dist_center_to_lateral_right_periodicity	context (ROI/target)	0	0	1
dist_center_to_lateral_right_std	context (ROI/target)	0	0	1
dist_center_to_lateral_right_trend	context (ROI/target)	0	0	1
dist_center_to_nose_delta	context (ROI/target)	0	0	1
dist_center_to_nose_energy	context (ROI/target)	0	0	1
dist_center_to_nose_max	context (ROI/target)	0	0	1
dist_center_to_nose_mean	context (ROI/target)	0	0	1

dist_center_to_nose_median	context (ROI/target)	0	0	1
dist_center_to_nose_norm_delta	context (ROI/target)	0	0	1
dist_center_to_nose_norm_energy	context (ROI/target)	0	0	1
dist_center_to_nose_norm_max	context (ROI/target)	0	0	1
dist_center_to_nose_norm_mean	context (ROI/target)	0	0	1
dist_center_to_nose_norm_median	context (ROI/target)	0	0	1
dist_center_to_nose_norm_p10	context (ROI/target)	0	0	1
dist_center_to_nose_norm_p90	context (ROI/target)	0	0	1
dist_center_to_nose_norm_periodicity	context (ROI/target)	0	0	1
dist_center_to_nose_norm_std	context (ROI/target)	0	0	1
dist_center_to_nose_norm_trend	context (ROI/target)	0	0	1
dist_center_to_nose_p10	context (ROI/target)	0	0	1
dist_center_to_nose_p90	context (ROI/target)	0	0	1
dist_center_to_nose_periodicity	context (ROI/target)	0	0	1
dist_center_to_nose_std	context (ROI/target)	0	0	1
dist_center_to_nose_trend	context (ROI/target)	0	0	1
dist_center_to_tail_base_delta	context (ROI/target)	0	0	1
dist_center_to_tail_base_energy	context (ROI/target)	0	0	1
dist_center_to_tail_base_max	context (ROI/target)	0	0	1
dist_center_to_tail_base_mean	context (ROI/target)	0	0	1
dist_center_to_tail_base_median	context (ROI/target)	0	0	1
dist_center_to_tail_base_norm_delta	context (ROI/target)	0	0	1
dist_center_to_tail_base_norm_energy	context (ROI/target)	0	0	1
dist_center_to_tail_base_norm_max	context (ROI/target)	0	0	1
dist_center_to_tail_base_norm_mean	context (ROI/target)	0	0	1
dist_center_to_tail_base_norm_median	context (ROI/target)	0	0	1
dist_center_to_tail_base_norm_p10	context (ROI/target)	0	0	1
dist_center_to_tail_base_norm_p90	context (ROI/target)	0	0	1
dist_center_to_tail_base_norm_periodicity	context (ROI/target)	0	0	1
dist_center_to_tail_base_norm_std	context (ROI/target)	0	0	1
dist_center_to_tail_base_norm_trend	context (ROI/target)	0	0	1
dist_center_to_tail_base_p10	context (ROI/target)	0	0	1
dist_center_to_tail_base_p90	context (ROI/target)	0	0	1
dist_center_to_tail_base_periodicity	context (ROI/target)	0	0	1
dist_center_to_tail_base_std	context (ROI/target)	0	0	1
dist_center_to_tail_base_trend	context (ROI/target)	0	0	1
dist_ear_left_to_ear_right_delta	kinematics	0	1	1
dist_ear_left_to_ear_right_energy	kinematics	0	1	1
dist_ear_left_to_ear_right_max	pose geometry	0	1	1
dist_ear_left_to_ear_right_mean	pose geometry	0	1	1
dist_ear_left_to_ear_right_median	pose geometry	0	1	1
dist_ear_left_to_ear_right_norm_delta	kinematics	0	1	1
dist_ear_left_to_ear_right_norm_energy	kinematics	0	1	1
dist_ear_left_to_ear_right_norm_max	pose geometry	0	1	1

dist_ear_left_to_ear_right_norm_mean	pose geometry	0	1	1
dist_ear_left_to_ear_right_norm_median	pose geometry	0	1	1
dist_ear_left_to_ear_right_norm_p10	pose geometry	0	1	1
dist_ear_left_to_ear_right_norm_p90	pose geometry	0	1	1
dist_ear_left_to_ear_right_norm_periodicity	pose geometry	0	1	1
dist_ear_left_to_ear_right_norm_std	kinematics	0	1	1
dist_ear_left_to_ear_right_norm_trend	pose geometry	0	1	1
dist_ear_left_to_ear_right_p10	pose geometry	0	1	1
dist_ear_left_to_ear_right_p90	pose geometry	0	1	1
dist_ear_left_to_ear_right_periodicity	pose geometry	0	1	1
dist_ear_left_to_ear_right_std	kinematics	0	1	1
dist_ear_left_to_ear_right_trend	pose geometry	0	1	1
dist_ear_left_to_lateral_left_delta	kinematics	0	0	1
dist_ear_left_to_lateral_left_energy	kinematics	0	0	1
dist_ear_left_to_lateral_left_max	pose geometry	0	0	1
dist_ear_left_to_lateral_left_mean	pose geometry	0	0	1
dist_ear_left_to_lateral_left_median	pose geometry	0	0	1
dist_ear_left_to_lateral_left_norm_delta	kinematics	0	0	1
dist_ear_left_to_lateral_left_norm_energy	kinematics	0	0	1
dist_ear_left_to_lateral_left_norm_max	pose geometry	0	0	1
dist_ear_left_to_lateral_left_norm_mean	pose geometry	0	0	1
dist_ear_left_to_lateral_left_norm_median	pose geometry	0	0	1
dist_ear_left_to_lateral_left_norm_p10	pose geometry	0	0	1
dist_ear_left_to_lateral_left_norm_p90	pose geometry	0	0	1
dist_ear_left_to_lateral_left_norm_periodicity	pose geometry	0	0	1
dist_ear_left_to_lateral_left_norm_std	kinematics	0	0	1
dist_ear_left_to_lateral_left_norm_trend	pose geometry	0	0	1
dist_ear_left_to_lateral_left_p10	pose geometry	0	0	1
dist_ear_left_to_lateral_left_p90	pose geometry	0	0	1
dist_ear_left_to_lateral_left_periodicity	pose geometry	0	0	1
dist_ear_left_to_lateral_left_std	kinematics	0	0	1
dist_ear_left_to_lateral_left_trend	pose geometry	0	0	1
dist_ear_left_to_lateral_right_delta	kinematics	0	0	1
dist_ear_left_to_lateral_right_energy	kinematics	0	0	1
dist_ear_left_to_lateral_right_max	pose geometry	0	0	1
dist_ear_left_to_lateral_right_mean	pose geometry	0	0	1
dist_ear_left_to_lateral_right_median	pose geometry	0	0	1
dist_ear_left_to_lateral_right_norm_delta	kinematics	0	0	1
dist_ear_left_to_lateral_right_norm_energy	kinematics	0	0	1
dist_ear_left_to_lateral_right_norm_max	pose geometry	0	0	1
dist_ear_left_to_lateral_right_norm_mean	pose geometry	0	0	1
dist_ear_left_to_lateral_right_norm_median	pose geometry	0	0	1
dist_ear_left_to_lateral_right_norm_p10	pose geometry	0	0	1
dist_ear_left_to_lateral_right_norm_p90	pose geometry	0	0	1

dist_ear_left_to_lateral_right_norm_periodicity	pose geometry	0	0	1
dist_ear_left_to_lateral_right_norm_std	kinematics	0	0	1
dist_ear_left_to_lateral_right_norm_trend	pose geometry	0	0	1
dist_ear_left_to_lateral_right_p10	pose geometry	0	0	1
dist_ear_left_to_lateral_right_p90	pose geometry	0	0	1
dist_ear_left_to_lateral_right_periodicity	pose geometry	0	0	1
dist_ear_left_to_lateral_right_std	kinematics	0	0	1
dist_ear_left_to_lateral_right_trend	pose geometry	0	0	1
dist_ear_left_to_nose_delta	kinematics	0	1	1
dist_ear_left_to_nose_energy	kinematics	0	1	1
dist_ear_left_to_nose_max	pose geometry	0	1	1
dist_ear_left_to_nose_mean	pose geometry	0	1	1
dist_ear_left_to_nose_median	pose geometry	0	1	1
dist_ear_left_to_nose_norm_delta	kinematics	0	1	1
dist_ear_left_to_nose_norm_energy	kinematics	0	1	1
dist_ear_left_to_nose_norm_max	pose geometry	0	1	1
dist_ear_left_to_nose_norm_mean	pose geometry	0	1	1
dist_ear_left_to_nose_norm_median	pose geometry	0	1	1
dist_ear_left_to_nose_norm_p10	pose geometry	0	1	1
dist_ear_left_to_nose_norm_p90	pose geometry	0	1	1
dist_ear_left_to_nose_norm_periodicity	pose geometry	0	1	1
dist_ear_left_to_nose_norm_std	kinematics	0	1	1
dist_ear_left_to_nose_norm_trend	pose geometry	0	1	1
dist_ear_left_to_nose_p10	pose geometry	0	1	1
dist_ear_left_to_nose_p90	pose geometry	0	1	1
dist_ear_left_to_nose_periodicity	pose geometry	0	1	1
dist_ear_left_to_nose_std	kinematics	0	1	1
dist_ear_left_to_nose_trend	pose geometry	0	1	1
dist_ear_left_to_tail_base_delta	kinematics	0	1	1
dist_ear_left_to_tail_base_energy	kinematics	0	1	1
dist_ear_left_to_tail_base_max	pose geometry	0	1	1
dist_ear_left_to_tail_base_mean	pose geometry	0	1	1
dist_ear_left_to_tail_base_median	pose geometry	0	1	1
dist_ear_left_to_tail_base_norm_delta	kinematics	0	1	1
dist_ear_left_to_tail_base_norm_energy	kinematics	0	1	1
dist_ear_left_to_tail_base_norm_max	pose geometry	0	1	1
dist_ear_left_to_tail_base_norm_mean	pose geometry	0	1	1
dist_ear_left_to_tail_base_norm_median	pose geometry	0	1	1
dist_ear_left_to_tail_base_norm_p10	pose geometry	0	1	1
dist_ear_left_to_tail_base_norm_p90	pose geometry	0	1	1
dist_ear_left_to_tail_base_norm_periodicity	pose geometry	0	1	1
dist_ear_left_to_tail_base_norm_std	kinematics	0	1	1
dist_ear_left_to_tail_base_norm_trend	pose geometry	0	1	1
dist_ear_left_to_tail_base_p10	pose geometry	0	1	1

dist_ear_left_to_tail_base_p90	pose geometry	0	1	1
dist_ear_left_to_tail_base_periodicity	pose geometry	0	1	1
dist_ear_left_to_tail_base_std	kinematics	0	1	1
dist_ear_left_to_tail_base_trend	pose geometry	0	1	1
dist_ear_right_to_lateral_left_delta	kinematics	0	0	1
dist_ear_right_to_lateral_left_energy	kinematics	0	0	1
dist_ear_right_to_lateral_left_max	pose geometry	0	0	1
dist_ear_right_to_lateral_left_mean	pose geometry	0	0	1
dist_ear_right_to_lateral_left_median	pose geometry	0	0	1
dist_ear_right_to_lateral_left_norm_delta	kinematics	0	0	1
dist_ear_right_to_lateral_left_norm_energy	kinematics	0	0	1
dist_ear_right_to_lateral_left_norm_max	pose geometry	0	0	1
dist_ear_right_to_lateral_left_norm_mean	pose geometry	0	0	1
dist_ear_right_to_lateral_left_norm_median	pose geometry	0	0	1
dist_ear_right_to_lateral_left_norm_p10	pose geometry	0	0	1
dist_ear_right_to_lateral_left_norm_p90	pose geometry	0	0	1
dist_ear_right_to_lateral_left_norm_periodicity	pose geometry	0	0	1
dist_ear_right_to_lateral_left_norm_std	kinematics	0	0	1
dist_ear_right_to_lateral_left_norm_trend	pose geometry	0	0	1
dist_ear_right_to_lateral_left_p10	pose geometry	0	0	1
dist_ear_right_to_lateral_left_p90	pose geometry	0	0	1
dist_ear_right_to_lateral_left_periodicity	pose geometry	0	0	1
dist_ear_right_to_lateral_left_std	kinematics	0	0	1
dist_ear_right_to_lateral_left_trend	pose geometry	0	0	1
dist_ear_right_to_lateral_right_delta	kinematics	0	0	1
dist_ear_right_to_lateral_right_energy	kinematics	0	0	1
dist_ear_right_to_lateral_right_max	pose geometry	0	0	1
dist_ear_right_to_lateral_right_mean	pose geometry	0	0	1
dist_ear_right_to_lateral_right_median	pose geometry	0	0	1
dist_ear_right_to_lateral_right_norm_delta	kinematics	0	0	1
dist_ear_right_to_lateral_right_norm_energy	kinematics	0	0	1
dist_ear_right_to_lateral_right_norm_max	pose geometry	0	0	1
dist_ear_right_to_lateral_right_norm_mean	pose geometry	0	0	1
dist_ear_right_to_lateral_right_norm_median	pose geometry	0	0	1
dist_ear_right_to_lateral_right_norm_p10	pose geometry	0	0	1
dist_ear_right_to_lateral_right_norm_p90	pose geometry	0	0	1
dist_ear_right_to_lateral_right_norm_periodicity	pose geometry	0	0	1
dist_ear_right_to_lateral_right_norm_std	kinematics	0	0	1
dist_ear_right_to_lateral_right_norm_trend	pose geometry	0	0	1
dist_ear_right_to_lateral_right_p10	pose geometry	0	0	1
dist_ear_right_to_lateral_right_p90	pose geometry	0	0	1
dist_ear_right_to_lateral_right_periodicity	pose geometry	0	0	1
dist_ear_right_to_lateral_right_std	kinematics	0	0	1
dist_ear_right_to_lateral_right_trend	pose geometry	0	0	1

dist_ear_right_to_nose_delta	kinematics	0	1	1
dist_ear_right_to_nose_energy	kinematics	0	1	1
dist_ear_right_to_nose_max	pose geometry	0	1	1
dist_ear_right_to_nose_mean	pose geometry	0	1	1
dist_ear_right_to_nose_median	pose geometry	0	1	1
dist_ear_right_to_nose_norm_delta	kinematics	0	1	1
dist_ear_right_to_nose_norm_energy	kinematics	0	1	1
dist_ear_right_to_nose_norm_max	pose geometry	0	1	1
dist_ear_right_to_nose_norm_mean	pose geometry	0	1	1
dist_ear_right_to_nose_norm_median	pose geometry	0	1	1
dist_ear_right_to_nose_norm_p10	pose geometry	0	1	1
dist_ear_right_to_nose_norm_p90	pose geometry	0	1	1
dist_ear_right_to_nose_norm_periodicity	pose geometry	0	1	1
dist_ear_right_to_nose_norm_std	kinematics	0	1	1
dist_ear_right_to_nose_norm_trend	pose geometry	0	1	1
dist_ear_right_to_nose_p10	pose geometry	0	1	1
dist_ear_right_to_nose_p90	pose geometry	0	1	1
dist_ear_right_to_nose_periodicity	pose geometry	0	1	1
dist_ear_right_to_nose_std	kinematics	0	1	1
dist_ear_right_to_nose_trend	pose geometry	0	1	1
dist_ear_right_to_tail_base_delta	kinematics	0	1	1
dist_ear_right_to_tail_base_energy	kinematics	0	1	1
dist_ear_right_to_tail_base_max	pose geometry	0	1	1
dist_ear_right_to_tail_base_mean	pose geometry	0	1	1
dist_ear_right_to_tail_base_median	pose geometry	0	1	1
dist_ear_right_to_tail_base_norm_delta	kinematics	0	1	1
dist_ear_right_to_tail_base_norm_energy	kinematics	0	1	1
dist_ear_right_to_tail_base_norm_max	pose geometry	0	1	1
dist_ear_right_to_tail_base_norm_mean	pose geometry	0	1	1
dist_ear_right_to_tail_base_norm_median	pose geometry	0	1	1
dist_ear_right_to_tail_base_norm_p10	pose geometry	0	1	1
dist_ear_right_to_tail_base_norm_p90	pose geometry	0	1	1
dist_ear_right_to_tail_base_norm_periodicity	pose geometry	0	1	1
dist_ear_right_to_tail_base_norm_std	kinematics	0	1	1
dist_ear_right_to_tail_base_norm_trend	pose geometry	0	1	1
dist_ear_right_to_tail_base_p10	pose geometry	0	1	1
dist_ear_right_to_tail_base_p90	pose geometry	0	1	1
dist_ear_right_to_tail_base_periodicity	pose geometry	0	1	1
dist_ear_right_to_tail_base_std	kinematics	0	1	1
dist_ear_right_to_tail_base_trend	pose geometry	0	1	1
dist_front_left_paw_to_front_right_paw_delta	kinematics	0	0	0
dist_front_left_paw_to_front_right_paw_energy	kinematics	0	0	0
dist_front_left_paw_to_front_right_paw_max	pose geometry	0	0	0
dist_front_left_paw_to_front_right_paw_mean	pose geometry	0	0	0

dist_front_left_paw_to_front_right_paw_median	pose geometry	0	0	0
dist_front_left_paw_to_front_right_paw_norm_delta	kinematics	0	0	0
dist_front_left_paw_to_front_right_paw_norm_energy	kinematics	0	0	0
dist_front_left_paw_to_front_right_paw_norm_max	pose geometry	0	0	0
dist_front_left_paw_to_front_right_paw_norm_mean	pose geometry	0	0	0
dist_front_left_paw_to_front_right_paw_norm_median	pose geometry	0	0	0
dist_front_left_paw_to_front_right_paw_norm_p10	pose geometry	0	0	0
dist_front_left_paw_to_front_right_paw_norm_p90	pose geometry	0	0	0
dist_front_left_paw_to_front_right_paw_norm_periodic	pose geometry	0	0	0
dist_front_left_paw_to_front_right_paw_norm_std	kinematics	0	0	0
dist_front_left_paw_to_front_right_paw_norm_trend	pose geometry	0	0	0
dist_front_left_paw_to_front_right_paw_p10	pose geometry	0	0	0
dist_front_left_paw_to_front_right_paw_p90	pose geometry	0	0	0
dist_front_left_paw_to_front_right_paw_periodicity	pose geometry	0	0	0
dist_front_left_paw_to_front_right_paw_std	kinematics	0	0	0
dist_front_left_paw_to_front_right_paw_trend	pose geometry	0	0	0
dist_front_left_paw_to_left_ear_delta	kinematics	0	0	0
dist_front_left_paw_to_left_ear_energy	kinematics	0	0	0
dist_front_left_paw_to_left_ear_max	pose geometry	0	0	0
dist_front_left_paw_to_left_ear_mean	pose geometry	0	0	0
dist_front_left_paw_to_left_ear_median	pose geometry	0	0	0
dist_front_left_paw_to_left_ear_norm_delta	kinematics	0	0	0
dist_front_left_paw_to_left_ear_norm_energy	kinematics	0	0	0
dist_front_left_paw_to_left_ear_norm_max	pose geometry	0	0	0
dist_front_left_paw_to_left_ear_norm_mean	pose geometry	0	0	0
dist_front_left_paw_to_left_ear_norm_median	pose geometry	0	0	0
dist_front_left_paw_to_left_ear_norm_p10	pose geometry	0	0	0
dist_front_left_paw_to_left_ear_norm_p90	pose geometry	0	0	0
dist_front_left_paw_to_left_ear_norm_periodicity	pose geometry	0	0	0
dist_front_left_paw_to_left_ear_norm_std	kinematics	0	0	0
dist_front_left_paw_to_left_ear_norm_trend	pose geometry	0	0	0
dist_front_left_paw_to_left_ear_p10	pose geometry	0	0	0
dist_front_left_paw_to_left_ear_p90	pose geometry	0	0	0
dist_front_left_paw_to_left_ear_periodicity	pose geometry	0	0	0
dist_front_left_paw_to_left_ear_std	kinematics	0	0	0
dist_front_left_paw_to_left_ear_trend	pose geometry	0	0	0
dist_front_left_paw_to_mid_back_delta	kinematics	0	0	0
dist_front_left_paw_to_mid_back_energy	kinematics	0	0	0
dist_front_left_paw_to_mid_back_max	pose geometry	0	0	0
dist_front_left_paw_to_mid_back_mean	pose geometry	0	0	0
dist_front_left_paw_to_mid_back_median	pose geometry	0	0	0
dist_front_left_paw_to_mid_back_norm_delta	kinematics	0	0	0
dist_front_left_paw_to_mid_back_norm_energy	kinematics	0	0	0
dist_front_left_paw_to_mid_back_norm_max	pose geometry	0	0	0

dist_front_left_paw_to_mid_back_norm_mean	pose geometry	0	0	0
dist_front_left_paw_to_mid_back_norm_median	pose geometry	0	0	0
dist_front_left_paw_to_mid_back_norm_p10	pose geometry	0	0	0
dist_front_left_paw_to_mid_back_norm_p90	pose geometry	0	0	0
dist_front_left_paw_to_mid_back_norm_periodicity	pose geometry	0	0	0
dist_front_left_paw_to_mid_back_norm_std	kinematics	0	0	0
dist_front_left_paw_to_mid_back_norm_trend	pose geometry	0	0	0
dist_front_left_paw_to_mid_back_p10	pose geometry	0	0	0
dist_front_left_paw_to_mid_back_p90	pose geometry	0	0	0
dist_front_left_paw_to_mid_back_periodicity	pose geometry	0	0	0
dist_front_left_paw_to_mid_back_std	kinematics	0	0	0
dist_front_left_paw_to_mid_back_trend	pose geometry	0	0	0
dist_front_left_paw_to_nose_delta	kinematics	0	0	0
dist_front_left_paw_to_nose_energy	kinematics	0	0	0
dist_front_left_paw_to_nose_max	pose geometry	0	0	0
dist_front_left_paw_to_nose_mean	pose geometry	0	0	0
dist_front_left_paw_to_nose_median	pose geometry	0	0	0
dist_front_left_paw_to_nose_norm_delta	kinematics	0	0	0
dist_front_left_paw_to_nose_norm_energy	kinematics	0	0	0
dist_front_left_paw_to_nose_norm_max	pose geometry	0	0	0
dist_front_left_paw_to_nose_norm_mean	pose geometry	0	0	0
dist_front_left_paw_to_nose_norm_median	pose geometry	0	0	0
dist_front_left_paw_to_nose_norm_p10	pose geometry	0	0	0
dist_front_left_paw_to_nose_norm_p90	pose geometry	0	0	0
dist_front_left_paw_to_nose_norm_periodicity	pose geometry	0	0	0
dist_front_left_paw_to_nose_norm_std	kinematics	0	0	0
dist_front_left_paw_to_nose_norm_trend	pose geometry	0	0	0
dist_front_left_paw_to_nose_p10	pose geometry	0	0	0
dist_front_left_paw_to_nose_p90	pose geometry	0	0	0
dist_front_left_paw_to_nose_periodicity	pose geometry	0	0	0
dist_front_left_paw_to_nose_std	kinematics	0	0	0
dist_front_left_paw_to_nose_trend	pose geometry	0	0	0
dist_front_left_paw_to_right_ear_delta	kinematics	0	0	0
dist_front_left_paw_to_right_ear_energy	kinematics	0	0	0
dist_front_left_paw_to_right_ear_max	pose geometry	0	0	0
dist_front_left_paw_to_right_ear_mean	pose geometry	0	0	0
dist_front_left_paw_to_right_ear_median	pose geometry	0	0	0
dist_front_left_paw_to_right_ear_norm_delta	kinematics	0	0	0
dist_front_left_paw_to_right_ear_norm_energy	kinematics	0	0	0
dist_front_left_paw_to_right_ear_norm_max	pose geometry	0	0	0
dist_front_left_paw_to_right_ear_norm_mean	pose geometry	0	0	0
dist_front_left_paw_to_right_ear_norm_median	pose geometry	0	0	0
dist_front_left_paw_to_right_ear_norm_p10	pose geometry	0	0	0
dist_front_left_paw_to_right_ear_norm_p90	pose geometry	0	0	0

dist_front_left_paw_to_right_ear_norm_periodicity	pose geometry	0	0	0
dist_front_left_paw_to_right_ear_norm_std	kinematics	0	0	0
dist_front_left_paw_to_right_ear_norm_trend	pose geometry	0	0	0
dist_front_left_paw_to_right_ear_p10	pose geometry	0	0	0
dist_front_left_paw_to_right_ear_p90	pose geometry	0	0	0
dist_front_left_paw_to_right_ear_periodicity	pose geometry	0	0	0
dist_front_left_paw_to_right_ear_std	kinematics	0	0	0
dist_front_left_paw_to_right_ear_trend	pose geometry	0	0	0
dist_front_left_paw_to_tail_base_delta	kinematics	0	0	0
dist_front_left_paw_to_tail_base_energy	kinematics	0	0	0
dist_front_left_paw_to_tail_base_max	pose geometry	0	0	0
dist_front_left_paw_to_tail_base_mean	pose geometry	0	0	0
dist_front_left_paw_to_tail_base_median	pose geometry	0	0	0
dist_front_left_paw_to_tail_base_norm_delta	kinematics	0	0	0
dist_front_left_paw_to_tail_base_norm_energy	kinematics	0	0	0
dist_front_left_paw_to_tail_base_norm_max	pose geometry	0	0	0
dist_front_left_paw_to_tail_base_norm_mean	pose geometry	0	0	0
dist_front_left_paw_to_tail_base_norm_median	pose geometry	0	0	0
dist_front_left_paw_to_tail_base_norm_p10	pose geometry	0	0	0
dist_front_left_paw_to_tail_base_norm_p90	pose geometry	0	0	0
dist_front_left_paw_to_tail_base_norm_periodicity	pose geometry	0	0	0
dist_front_left_paw_to_tail_base_norm_std	kinematics	0	0	0
dist_front_left_paw_to_tail_base_norm_trend	pose geometry	0	0	0
dist_front_left_paw_to_tail_base_p10	pose geometry	0	0	0
dist_front_left_paw_to_tail_base_p90	pose geometry	0	0	0
dist_front_left_paw_to_tail_base_periodicity	pose geometry	0	0	0
dist_front_left_paw_to_tail_base_std	kinematics	0	0	0
dist_front_left_paw_to_tail_base_trend	pose geometry	0	0	0
dist_front_right_paw_to_left_ear_delta	kinematics	0	0	0
dist_front_right_paw_to_left_ear_energy	kinematics	0	0	0
dist_front_right_paw_to_left_ear_max	pose geometry	0	0	0
dist_front_right_paw_to_left_ear_mean	pose geometry	0	0	0
dist_front_right_paw_to_left_ear_median	pose geometry	0	0	0
dist_front_right_paw_to_left_ear_norm_delta	kinematics	0	0	0
dist_front_right_paw_to_left_ear_norm_energy	kinematics	0	0	0
dist_front_right_paw_to_left_ear_norm_max	pose geometry	0	0	0
dist_front_right_paw_to_left_ear_norm_mean	pose geometry	0	0	0
dist_front_right_paw_to_left_ear_norm_median	pose geometry	0	0	0
dist_front_right_paw_to_left_ear_norm_p10	pose geometry	0	0	0
dist_front_right_paw_to_left_ear_norm_p90	pose geometry	0	0	0
dist_front_right_paw_to_left_ear_norm_periodicity	pose geometry	0	0	0
dist_front_right_paw_to_left_ear_norm_std	kinematics	0	0	0
dist_front_right_paw_to_left_ear_norm_trend	pose geometry	0	0	0
dist_front_right_paw_to_left_ear_p10	pose geometry	0	0	0

dist_front_right_paw_to_left_ear_p90	pose geometry	0	0	0
dist_front_right_paw_to_left_ear_periodicity	pose geometry	0	0	0
dist_front_right_paw_to_left_ear_std	kinematics	0	0	0
dist_front_right_paw_to_left_ear_trend	pose geometry	0	0	0
dist_front_right_paw_to_mid_back_delta	kinematics	0	0	0
dist_front_right_paw_to_mid_back_energy	kinematics	0	0	0
dist_front_right_paw_to_mid_back_max	pose geometry	0	0	0
dist_front_right_paw_to_mid_back_mean	pose geometry	0	0	0
dist_front_right_paw_to_mid_back_median	pose geometry	0	0	0
dist_front_right_paw_to_mid_back_norm_delta	kinematics	0	0	0
dist_front_right_paw_to_mid_back_norm_energy	kinematics	0	0	0
dist_front_right_paw_to_mid_back_norm_max	pose geometry	0	0	0
dist_front_right_paw_to_mid_back_norm_mean	pose geometry	0	0	0
dist_front_right_paw_to_mid_back_norm_median	pose geometry	0	0	0
dist_front_right_paw_to_mid_back_norm_p10	pose geometry	0	0	0
dist_front_right_paw_to_mid_back_norm_p90	pose geometry	0	0	0
dist_front_right_paw_to_mid_back_norm_periodicity	pose geometry	0	0	0
dist_front_right_paw_to_mid_back_norm_std	kinematics	0	0	0
dist_front_right_paw_to_mid_back_norm_trend	pose geometry	0	0	0
dist_front_right_paw_to_mid_back_p10	pose geometry	0	0	0
dist_front_right_paw_to_mid_back_p90	pose geometry	0	0	0
dist_front_right_paw_to_mid_back_periodicity	pose geometry	0	0	0
dist_front_right_paw_to_mid_back_std	kinematics	0	0	0
dist_front_right_paw_to_mid_back_trend	pose geometry	0	0	0
dist_front_right_paw_to_nose_delta	kinematics	0	0	0
dist_front_right_paw_to_nose_energy	kinematics	0	0	0
dist_front_right_paw_to_nose_max	pose geometry	0	0	0
dist_front_right_paw_to_nose_mean	pose geometry	0	0	0
dist_front_right_paw_to_nose_median	pose geometry	0	0	0
dist_front_right_paw_to_nose_norm_delta	kinematics	0	0	0
dist_front_right_paw_to_nose_norm_energy	kinematics	0	0	0
dist_front_right_paw_to_nose_norm_max	pose geometry	0	0	0
dist_front_right_paw_to_nose_norm_mean	pose geometry	0	0	0
dist_front_right_paw_to_nose_norm_median	pose geometry	0	0	0
dist_front_right_paw_to_nose_norm_p10	pose geometry	0	0	0
dist_front_right_paw_to_nose_norm_p90	pose geometry	0	0	0
dist_front_right_paw_to_nose_norm_periodicity	pose geometry	0	0	0
dist_front_right_paw_to_nose_norm_std	kinematics	0	0	0
dist_front_right_paw_to_nose_norm_trend	pose geometry	0	0	0
dist_front_right_paw_to_nose_p10	pose geometry	0	0	0
dist_front_right_paw_to_nose_p90	pose geometry	0	0	0
dist_front_right_paw_to_nose_periodicity	pose geometry	0	0	0
dist_front_right_paw_to_nose_std	kinematics	0	0	0
dist_front_right_paw_to_nose_trend	pose geometry	0	0	0

dist_front_right_paw_to_right_ear_delta	kinematics	0	0	0
dist_front_right_paw_to_right_ear_energy	kinematics	0	0	0
dist_front_right_paw_to_right_ear_max	pose geometry	0	0	0
dist_front_right_paw_to_right_ear_mean	pose geometry	0	0	0
dist_front_right_paw_to_right_ear_median	pose geometry	0	0	0
dist_front_right_paw_to_right_ear_norm_delta	kinematics	0	0	0
dist_front_right_paw_to_right_ear_norm_energy	kinematics	0	0	0
dist_front_right_paw_to_right_ear_norm_max	pose geometry	0	0	0
dist_front_right_paw_to_right_ear_norm_mean	pose geometry	0	0	0
dist_front_right_paw_to_right_ear_norm_median	pose geometry	0	0	0
dist_front_right_paw_to_right_ear_norm_p10	pose geometry	0	0	0
dist_front_right_paw_to_right_ear_norm_p90	pose geometry	0	0	0
dist_front_right_paw_to_right_ear_norm_periodicity	pose geometry	0	0	0
dist_front_right_paw_to_right_ear_norm_std	kinematics	0	0	0
dist_front_right_paw_to_right_ear_norm_trend	pose geometry	0	0	0
dist_front_right_paw_to_right_ear_p10	pose geometry	0	0	0
dist_front_right_paw_to_right_ear_p90	pose geometry	0	0	0
dist_front_right_paw_to_right_ear_periodicity	pose geometry	0	0	0
dist_front_right_paw_to_right_ear_std	kinematics	0	0	0
dist_front_right_paw_to_right_ear_trend	pose geometry	0	0	0
dist_front_right_paw_to_tail_base_delta	kinematics	0	0	0
dist_front_right_paw_to_tail_base_energy	kinematics	0	0	0
dist_front_right_paw_to_tail_base_max	pose geometry	0	0	0
dist_front_right_paw_to_tail_base_mean	pose geometry	0	0	0
dist_front_right_paw_to_tail_base_median	pose geometry	0	0	0
dist_front_right_paw_to_tail_base_norm_delta	kinematics	0	0	0
dist_front_right_paw_to_tail_base_norm_energy	kinematics	0	0	0
dist_front_right_paw_to_tail_base_norm_max	pose geometry	0	0	0
dist_front_right_paw_to_tail_base_norm_mean	pose geometry	0	0	0
dist_front_right_paw_to_tail_base_norm_median	pose geometry	0	0	0
dist_front_right_paw_to_tail_base_norm_p10	pose geometry	0	0	0
dist_front_right_paw_to_tail_base_norm_p90	pose geometry	0	0	0
dist_front_right_paw_to_tail_base_norm_periodicity	pose geometry	0	0	0
dist_front_right_paw_to_tail_base_norm_std	kinematics	0	0	0
dist_front_right_paw_to_tail_base_norm_trend	pose geometry	0	0	0
dist_front_right_paw_to_tail_base_p10	pose geometry	0	0	0
dist_front_right_paw_to_tail_base_p90	pose geometry	0	0	0
dist_front_right_paw_to_tail_base_periodicity	pose geometry	0	0	0
dist_front_right_paw_to_tail_base_std	kinematics	0	0	0
dist_front_right_paw_to_tail_base_trend	pose geometry	0	0	0
dist_lateral_left_to_lateral_right_delta	kinematics	0	0	1
dist_lateral_left_to_lateral_right_energy	kinematics	0	0	1
dist_lateral_left_to_lateral_right_max	pose geometry	0	0	1
dist_lateral_left_to_lateral_right_mean	pose geometry	0	0	1

dist_lateral_left_to_lateral_right_median	pose geometry	0	0	1
dist_lateral_left_to_lateral_right_norm_delta	kinematics	0	0	1
dist_lateral_left_to_lateral_right_norm_energy	kinematics	0	0	1
dist_lateral_left_to_lateral_right_norm_max	pose geometry	0	0	1
dist_lateral_left_to_lateral_right_norm_mean	pose geometry	0	0	1
dist_lateral_left_to_lateral_right_norm_median	pose geometry	0	0	1
dist_lateral_left_to_lateral_right_norm_p10	pose geometry	0	0	1
dist_lateral_left_to_lateral_right_norm_p90	pose geometry	0	0	1
dist_lateral_left_to_lateral_right_norm_periodicity	pose geometry	0	0	1
dist_lateral_left_to_lateral_right_norm_std	kinematics	0	0	1
dist_lateral_left_to_lateral_right_norm_trend	pose geometry	0	0	1
dist_lateral_left_to_lateral_right_p10	pose geometry	0	0	1
dist_lateral_left_to_lateral_right_p90	pose geometry	0	0	1
dist_lateral_left_to_lateral_right_periodicity	pose geometry	0	0	1
dist_lateral_left_to_lateral_right_std	kinematics	0	0	1
dist_lateral_left_to_lateral_right_trend	pose geometry	0	0	1
dist_lateral_left_to_nose_delta	kinematics	0	0	1
dist_lateral_left_to_nose_energy	kinematics	0	0	1
dist_lateral_left_to_nose_max	pose geometry	0	0	1
dist_lateral_left_to_nose_mean	pose geometry	0	0	1
dist_lateral_left_to_nose_median	pose geometry	0	0	1
dist_lateral_left_to_nose_norm_delta	kinematics	0	0	1
dist_lateral_left_to_nose_norm_energy	kinematics	0	0	1
dist_lateral_left_to_nose_norm_max	pose geometry	0	0	1
dist_lateral_left_to_nose_norm_mean	pose geometry	0	0	1
dist_lateral_left_to_nose_norm_median	pose geometry	0	0	1
dist_lateral_left_to_nose_norm_p10	pose geometry	0	0	1
dist_lateral_left_to_nose_norm_p90	pose geometry	0	0	1
dist_lateral_left_to_nose_norm_periodicity	pose geometry	0	0	1
dist_lateral_left_to_nose_norm_std	kinematics	0	0	1
dist_lateral_left_to_nose_norm_trend	pose geometry	0	0	1
dist_lateral_left_to_nose_p10	pose geometry	0	0	1
dist_lateral_left_to_nose_p90	pose geometry	0	0	1
dist_lateral_left_to_nose_periodicity	pose geometry	0	0	1
dist_lateral_left_to_nose_std	kinematics	0	0	1
dist_lateral_left_to_nose_trend	pose geometry	0	0	1
dist_lateral_left_to_tail_base_delta	kinematics	0	0	1
dist_lateral_left_to_tail_base_energy	kinematics	0	0	1
dist_lateral_left_to_tail_base_max	pose geometry	0	0	1
dist_lateral_left_to_tail_base_mean	pose geometry	0	0	1
dist_lateral_left_to_tail_base_median	pose geometry	0	0	1
dist_lateral_left_to_tail_base_norm_delta	kinematics	0	0	1
dist_lateral_left_to_tail_base_norm_energy	kinematics	0	0	1
dist_lateral_left_to_tail_base_norm_max	pose geometry	0	0	1

dist_lateral_left_to_tail_base_norm_mean	pose geometry	0	0	1
dist_lateral_left_to_tail_base_norm_median	pose geometry	0	0	1
dist_lateral_left_to_tail_base_norm_p10	pose geometry	0	0	1
dist_lateral_left_to_tail_base_norm_p90	pose geometry	0	0	1
dist_lateral_left_to_tail_base_norm_periodicity	pose geometry	0	0	1
dist_lateral_left_to_tail_base_norm_std	kinematics	0	0	1
dist_lateral_left_to_tail_base_norm_trend	pose geometry	0	0	1
dist_lateral_left_to_tail_base_p10	pose geometry	0	0	1
dist_lateral_left_to_tail_base_p90	pose geometry	0	0	1
dist_lateral_left_to_tail_base_periodicity	pose geometry	0	0	1
dist_lateral_left_to_tail_base_std	kinematics	0	0	1
dist_lateral_left_to_tail_base_trend	pose geometry	0	0	1
dist_lateral_right_to_nose_delta	kinematics	0	0	1
dist_lateral_right_to_nose_energy	kinematics	0	0	1
dist_lateral_right_to_nose_max	pose geometry	0	0	1
dist_lateral_right_to_nose_mean	pose geometry	0	0	1
dist_lateral_right_to_nose_median	pose geometry	0	0	1
dist_lateral_right_to_nose_norm_delta	kinematics	0	0	1
dist_lateral_right_to_nose_norm_energy	kinematics	0	0	1
dist_lateral_right_to_nose_norm_max	pose geometry	0	0	1
dist_lateral_right_to_nose_norm_mean	pose geometry	0	0	1
dist_lateral_right_to_nose_norm_median	pose geometry	0	0	1
dist_lateral_right_to_nose_norm_p10	pose geometry	0	0	1
dist_lateral_right_to_nose_norm_p90	pose geometry	0	0	1
dist_lateral_right_to_nose_norm_periodicity	pose geometry	0	0	1
dist_lateral_right_to_nose_norm_std	kinematics	0	0	1
dist_lateral_right_to_nose_norm_trend	pose geometry	0	0	1
dist_lateral_right_to_nose_p10	pose geometry	0	0	1
dist_lateral_right_to_nose_p90	pose geometry	0	0	1
dist_lateral_right_to_nose_periodicity	pose geometry	0	0	1
dist_lateral_right_to_nose_std	kinematics	0	0	1
dist_lateral_right_to_nose_trend	pose geometry	0	0	1
dist_lateral_right_to_tail_base_delta	kinematics	0	0	1
dist_lateral_right_to_tail_base_energy	kinematics	0	0	1
dist_lateral_right_to_tail_base_max	pose geometry	0	0	1
dist_lateral_right_to_tail_base_mean	pose geometry	0	0	1
dist_lateral_right_to_tail_base_median	pose geometry	0	0	1
dist_lateral_right_to_tail_base_norm_delta	kinematics	0	0	1
dist_lateral_right_to_tail_base_norm_energy	kinematics	0	0	1
dist_lateral_right_to_tail_base_norm_max	pose geometry	0	0	1
dist_lateral_right_to_tail_base_norm_mean	pose geometry	0	0	1
dist_lateral_right_to_tail_base_norm_median	pose geometry	0	0	1
dist_lateral_right_to_tail_base_norm_p10	pose geometry	0	0	1
dist_lateral_right_to_tail_base_norm_p90	pose geometry	0	0	1

dist_lateral_right_to_tail_base_norm_periodicity	pose geometry	0	0	1
dist_lateral_right_to_tail_base_norm_std	kinematics	0	0	1
dist_lateral_right_to_tail_base_norm_trend	pose geometry	0	0	1
dist_lateral_right_to_tail_base_p10	pose geometry	0	0	1
dist_lateral_right_to_tail_base_p90	pose geometry	0	0	1
dist_lateral_right_to_tail_base_periodicity	pose geometry	0	0	1
dist_lateral_right_to_tail_base_std	kinematics	0	0	1
dist_lateral_right_to_tail_base_trend	pose geometry	0	0	1
dist_left_body_to_left_ear_delta	kinematics	1	0	0
dist_left_body_to_left_ear_energy	kinematics	1	0	0
dist_left_body_to_left_ear_max	pose geometry	1	0	0
dist_left_body_to_left_ear_mean	pose geometry	1	0	0
dist_left_body_to_left_ear_median	pose geometry	1	0	0
dist_left_body_to_left_ear_norm_delta	kinematics	1	0	0
dist_left_body_to_left_ear_norm_energy	kinematics	1	0	0
dist_left_body_to_left_ear_norm_max	pose geometry	1	0	0
dist_left_body_to_left_ear_norm_mean	pose geometry	1	0	0
dist_left_body_to_left_ear_norm_median	pose geometry	1	0	0
dist_left_body_to_left_ear_norm_p10	pose geometry	1	0	0
dist_left_body_to_left_ear_norm_p90	pose geometry	1	0	0
dist_left_body_to_left_ear_norm_periodicity	pose geometry	1	0	0
dist_left_body_to_left_ear_norm_std	kinematics	1	0	0
dist_left_body_to_left_ear_norm_trend	pose geometry	1	0	0
dist_left_body_to_left_ear_p10	pose geometry	1	0	0
dist_left_body_to_left_ear_p90	pose geometry	1	0	0
dist_left_body_to_left_ear_periodicity	pose geometry	1	0	0
dist_left_body_to_left_ear_std	kinematics	1	0	0
dist_left_body_to_left_ear_trend	pose geometry	1	0	0
dist_left_body_to_nose_delta	kinematics	1	0	0
dist_left_body_to_nose_energy	kinematics	1	0	0
dist_left_body_to_nose_max	pose geometry	1	0	0
dist_left_body_to_nose_mean	pose geometry	1	0	0
dist_left_body_to_nose_median	pose geometry	1	0	0
dist_left_body_to_nose_norm_delta	kinematics	1	0	0
dist_left_body_to_nose_norm_energy	kinematics	1	0	0
dist_left_body_to_nose_norm_max	pose geometry	1	0	0
dist_left_body_to_nose_norm_mean	pose geometry	1	0	0
dist_left_body_to_nose_norm_median	pose geometry	1	0	0
dist_left_body_to_nose_norm_p10	pose geometry	1	0	0
dist_left_body_to_nose_norm_p90	pose geometry	1	0	0
dist_left_body_to_nose_norm_periodicity	pose geometry	1	0	0
dist_left_body_to_nose_norm_std	kinematics	1	0	0
dist_left_body_to_nose_norm_trend	pose geometry	1	0	0
dist_left_body_to_nose_p10	pose geometry	1	0	0

dist_left_body_to_nose_p90	pose geometry	1	0	0
dist_left_body_to_nose_periodicity	pose geometry	1	0	0
dist_left_body_to_nose_std	kinematics	1	0	0
dist_left_body_to_nose_trend	pose geometry	1	0	0
dist_left_body_to_right_body_delta	kinematics	1	0	0
dist_left_body_to_right_body_energy	kinematics	1	0	0
dist_left_body_to_right_body_max	pose geometry	1	0	0
dist_left_body_to_right_body_mean	pose geometry	1	0	0
dist_left_body_to_right_body_median	pose geometry	1	0	0
dist_left_body_to_right_body_norm_delta	kinematics	1	0	0
dist_left_body_to_right_body_norm_energy	kinematics	1	0	0
dist_left_body_to_right_body_norm_max	pose geometry	1	0	0
dist_left_body_to_right_body_norm_mean	pose geometry	1	0	0
dist_left_body_to_right_body_norm_median	pose geometry	1	0	0
dist_left_body_to_right_body_norm_p10	pose geometry	1	0	0
dist_left_body_to_right_body_norm_p90	pose geometry	1	0	0
dist_left_body_to_right_body_norm_periodicity	pose geometry	1	0	0
dist_left_body_to_right_body_norm_std	kinematics	1	0	0
dist_left_body_to_right_body_norm_trend	pose geometry	1	0	0
dist_left_body_to_right_body_p10	pose geometry	1	0	0
dist_left_body_to_right_body_p90	pose geometry	1	0	0
dist_left_body_to_right_body_periodicity	pose geometry	1	0	0
dist_left_body_to_right_body_std	kinematics	1	0	0
dist_left_body_to_right_body_trend	pose geometry	1	0	0
dist_left_body_to_right_ear_delta	kinematics	1	0	0
dist_left_body_to_right_ear_energy	kinematics	1	0	0
dist_left_body_to_right_ear_max	pose geometry	1	0	0
dist_left_body_to_right_ear_mean	pose geometry	1	0	0
dist_left_body_to_right_ear_median	pose geometry	1	0	0
dist_left_body_to_right_ear_norm_delta	kinematics	1	0	0
dist_left_body_to_right_ear_norm_energy	kinematics	1	0	0
dist_left_body_to_right_ear_norm_max	pose geometry	1	0	0
dist_left_body_to_right_ear_norm_mean	pose geometry	1	0	0
dist_left_body_to_right_ear_norm_median	pose geometry	1	0	0
dist_left_body_to_right_ear_norm_p10	pose geometry	1	0	0
dist_left_body_to_right_ear_norm_p90	pose geometry	1	0	0
dist_left_body_to_right_ear_norm_periodicity	pose geometry	1	0	0
dist_left_body_to_right_ear_norm_std	kinematics	1	0	0
dist_left_body_to_right_ear_norm_trend	pose geometry	1	0	0
dist_left_body_to_right_ear_p10	pose geometry	1	0	0
dist_left_body_to_right_ear_p90	pose geometry	1	0	0
dist_left_body_to_right_ear_periodicity	pose geometry	1	0	0
dist_left_body_to_right_ear_std	kinematics	1	0	0
dist_left_body_to_right_ear_trend	pose geometry	1	0	0

dist_left_body_to_tail_base_delta	kinematics	1	0	0
dist_left_body_to_tail_base_energy	kinematics	1	0	0
dist_left_body_to_tail_base_max	pose geometry	1	0	0
dist_left_body_to_tail_base_mean	pose geometry	1	0	0
dist_left_body_to_tail_base_median	pose geometry	1	0	0
dist_left_body_to_tail_base_norm_delta	kinematics	1	0	0
dist_left_body_to_tail_base_norm_energy	kinematics	1	0	0
dist_left_body_to_tail_base_norm_max	pose geometry	1	0	0
dist_left_body_to_tail_base_norm_mean	pose geometry	1	0	0
dist_left_body_to_tail_base_norm_median	pose geometry	1	0	0
dist_left_body_to_tail_base_norm_p10	pose geometry	1	0	0
dist_left_body_to_tail_base_norm_p90	pose geometry	1	0	0
dist_left_body_to_tail_base_norm_periodicity	pose geometry	1	0	0
dist_left_body_to_tail_base_norm_std	kinematics	1	0	0
dist_left_body_to_tail_base_norm_trend	pose geometry	1	0	0
dist_left_body_to_tail_base_p10	pose geometry	1	0	0
dist_left_body_to_tail_base_p90	pose geometry	1	0	0
dist_left_body_to_tail_base_periodicity	pose geometry	1	0	0
dist_left_body_to_tail_base_std	kinematics	1	0	0
dist_left_body_to_tail_base_trend	pose geometry	1	0	0
dist_left_ear_to_mid_back_delta	kinematics	0	0	0
dist_left_ear_to_mid_back_energy	kinematics	0	0	0
dist_left_ear_to_mid_back_max	pose geometry	0	0	0
dist_left_ear_to_mid_back_mean	pose geometry	0	0	0
dist_left_ear_to_mid_back_median	pose geometry	0	0	0
dist_left_ear_to_mid_back_norm_delta	kinematics	0	0	0
dist_left_ear_to_mid_back_norm_energy	kinematics	0	0	0
dist_left_ear_to_mid_back_norm_max	pose geometry	0	0	0
dist_left_ear_to_mid_back_norm_mean	pose geometry	0	0	0
dist_left_ear_to_mid_back_norm_median	pose geometry	0	0	0
dist_left_ear_to_mid_back_norm_p10	pose geometry	0	0	0
dist_left_ear_to_mid_back_norm_p90	pose geometry	0	0	0
dist_left_ear_to_mid_back_norm_periodicity	pose geometry	0	0	0
dist_left_ear_to_mid_back_norm_std	kinematics	0	0	0
dist_left_ear_to_mid_back_norm_trend	pose geometry	0	0	0
dist_left_ear_to_mid_back_p10	pose geometry	0	0	0
dist_left_ear_to_mid_back_p90	pose geometry	0	0	0
dist_left_ear_to_mid_back_periodicity	pose geometry	0	0	0
dist_left_ear_to_mid_back_std	kinematics	0	0	0
dist_left_ear_to_mid_back_trend	pose geometry	0	0	0
dist_left_ear_to_nose_delta	kinematics	1	0	0
dist_left_ear_to_nose_energy	kinematics	1	0	0
dist_left_ear_to_nose_max	pose geometry	1	0	0
dist_left_ear_to_nose_mean	pose geometry	1	0	0

dist_left_ear_to_nose_median	pose geometry	1	0	0
dist_left_ear_to_nose_norm_delta	kinematics	1	0	0
dist_left_ear_to_nose_norm_energy	kinematics	1	0	0
dist_left_ear_to_nose_norm_max	pose geometry	1	0	0
dist_left_ear_to_nose_norm_mean	pose geometry	1	0	0
dist_left_ear_to_nose_norm_median	pose geometry	1	0	0
dist_left_ear_to_nose_norm_p10	pose geometry	1	0	0
dist_left_ear_to_nose_norm_p90	pose geometry	1	0	0
dist_left_ear_to_nose_norm_periodicity	pose geometry	1	0	0
dist_left_ear_to_nose_norm_std	kinematics	1	0	0
dist_left_ear_to_nose_norm_trend	pose geometry	1	0	0
dist_left_ear_to_nose_p10	pose geometry	1	0	0
dist_left_ear_to_nose_p90	pose geometry	1	0	0
dist_left_ear_to_nose_periodicity	pose geometry	1	0	0
dist_left_ear_to_nose_std	kinematics	1	0	0
dist_left_ear_to_nose_trend	pose geometry	1	0	0
dist_left_ear_to_right_body_delta	kinematics	1	0	0
dist_left_ear_to_right_body_energy	kinematics	1	0	0
dist_left_ear_to_right_body_max	pose geometry	1	0	0
dist_left_ear_to_right_body_mean	pose geometry	1	0	0
dist_left_ear_to_right_body_median	pose geometry	1	0	0
dist_left_ear_to_right_body_norm_delta	kinematics	1	0	0
dist_left_ear_to_right_body_norm_energy	kinematics	1	0	0
dist_left_ear_to_right_body_norm_max	pose geometry	1	0	0
dist_left_ear_to_right_body_norm_mean	pose geometry	1	0	0
dist_left_ear_to_right_body_norm_median	pose geometry	1	0	0
dist_left_ear_to_right_body_norm_p10	pose geometry	1	0	0
dist_left_ear_to_right_body_norm_p90	pose geometry	1	0	0
dist_left_ear_to_right_body_norm_periodicity	pose geometry	1	0	0
dist_left_ear_to_right_body_norm_std	kinematics	1	0	0
dist_left_ear_to_right_body_norm_trend	pose geometry	1	0	0
dist_left_ear_to_right_body_p10	pose geometry	1	0	0
dist_left_ear_to_right_body_p90	pose geometry	1	0	0
dist_left_ear_to_right_body_periodicity	pose geometry	1	0	0
dist_left_ear_to_right_body_std	kinematics	1	0	0
dist_left_ear_to_right_body_trend	pose geometry	1	0	0
dist_left_ear_to_right_ear_delta	kinematics	1	0	0
dist_left_ear_to_right_ear_energy	kinematics	1	0	0
dist_left_ear_to_right_ear_max	pose geometry	1	0	0
dist_left_ear_to_right_ear_mean	pose geometry	1	0	0
dist_left_ear_to_right_ear_median	pose geometry	1	0	0
dist_left_ear_to_right_ear_norm_delta	kinematics	1	0	0
dist_left_ear_to_right_ear_norm_energy	kinematics	1	0	0
dist_left_ear_to_right_ear_norm_max	pose geometry	1	0	0

dist_left_ear_to_right_ear_norm_mean	pose geometry	1	0	0
dist_left_ear_to_right_ear_norm_median	pose geometry	1	0	0
dist_left_ear_to_right_ear_norm_p10	pose geometry	1	0	0
dist_left_ear_to_right_ear_norm_p90	pose geometry	1	0	0
dist_left_ear_to_right_ear_norm_periodicity	pose geometry	1	0	0
dist_left_ear_to_right_ear_norm_std	kinematics	1	0	0
dist_left_ear_to_right_ear_norm_trend	pose geometry	1	0	0
dist_left_ear_to_right_ear_p10	pose geometry	1	0	0
dist_left_ear_to_right_ear_p90	pose geometry	1	0	0
dist_left_ear_to_right_ear_periodicity	pose geometry	1	0	0
dist_left_ear_to_right_ear_std	kinematics	1	0	0
dist_left_ear_to_right_ear_trend	pose geometry	1	0	0
dist_left_ear_to_tail_base_delta	kinematics	1	0	0
dist_left_ear_to_tail_base_energy	kinematics	1	0	0
dist_left_ear_to_tail_base_max	pose geometry	1	0	0
dist_left_ear_to_tail_base_mean	pose geometry	1	0	0
dist_left_ear_to_tail_base_median	pose geometry	1	0	0
dist_left_ear_to_tail_base_norm_delta	kinematics	1	0	0
dist_left_ear_to_tail_base_norm_energy	kinematics	1	0	0
dist_left_ear_to_tail_base_norm_max	pose geometry	1	0	0
dist_left_ear_to_tail_base_norm_mean	pose geometry	1	0	0
dist_left_ear_to_tail_base_norm_median	pose geometry	1	0	0
dist_left_ear_to_tail_base_norm_p10	pose geometry	1	0	0
dist_left_ear_to_tail_base_norm_p90	pose geometry	1	0	0
dist_left_ear_to_tail_base_norm_periodicity	pose geometry	1	0	0
dist_left_ear_to_tail_base_norm_std	kinematics	1	0	0
dist_left_ear_to_tail_base_norm_trend	pose geometry	1	0	0
dist_left_ear_to_tail_base_p10	pose geometry	1	0	0
dist_left_ear_to_tail_base_p90	pose geometry	1	0	0
dist_left_ear_to_tail_base_periodicity	pose geometry	1	0	0
dist_left_ear_to_tail_base_std	kinematics	1	0	0
dist_left_ear_to_tail_base_trend	pose geometry	1	0	0
dist_mid_back_to_nose_delta	kinematics	0	0	0
dist_mid_back_to_nose_energy	kinematics	0	0	0
dist_mid_back_to_nose_max	pose geometry	0	0	0
dist_mid_back_to_nose_mean	pose geometry	0	0	0
dist_mid_back_to_nose_median	pose geometry	0	0	0
dist_mid_back_to_nose_norm_delta	kinematics	0	0	0
dist_mid_back_to_nose_norm_energy	kinematics	0	0	0
dist_mid_back_to_nose_norm_max	pose geometry	0	0	0
dist_mid_back_to_nose_norm_mean	pose geometry	0	0	0
dist_mid_back_to_nose_norm_median	pose geometry	0	0	0
dist_mid_back_to_nose_norm_p10	pose geometry	0	0	0
dist_mid_back_to_nose_norm_p90	pose geometry	0	0	0

dist_mid_back_to_nose_norm_periodicity	pose geometry	0	0	0
dist_mid_back_to_nose_norm_std	kinematics	0	0	0
dist_mid_back_to_nose_norm_trend	pose geometry	0	0	0
dist_mid_back_to_nose_p10	pose geometry	0	0	0
dist_mid_back_to_nose_p90	pose geometry	0	0	0
dist_mid_back_to_nose_periodicity	pose geometry	0	0	0
dist_mid_back_to_nose_std	kinematics	0	0	0
dist_mid_back_to_nose_trend	pose geometry	0	0	0
dist_mid_back_to_right_ear_delta	kinematics	0	0	0
dist_mid_back_to_right_ear_energy	kinematics	0	0	0
dist_mid_back_to_right_ear_max	pose geometry	0	0	0
dist_mid_back_to_right_ear_mean	pose geometry	0	0	0
dist_mid_back_to_right_ear_median	pose geometry	0	0	0
dist_mid_back_to_right_ear_norm_delta	kinematics	0	0	0
dist_mid_back_to_right_ear_norm_energy	kinematics	0	0	0
dist_mid_back_to_right_ear_norm_max	pose geometry	0	0	0
dist_mid_back_to_right_ear_norm_mean	pose geometry	0	0	0
dist_mid_back_to_right_ear_norm_median	pose geometry	0	0	0
dist_mid_back_to_right_ear_norm_p10	pose geometry	0	0	0
dist_mid_back_to_right_ear_norm_p90	pose geometry	0	0	0
dist_mid_back_to_right_ear_norm_periodicity	pose geometry	0	0	0
dist_mid_back_to_right_ear_norm_std	kinematics	0	0	0
dist_mid_back_to_right_ear_norm_trend	pose geometry	0	0	0
dist_mid_back_to_right_ear_p10	pose geometry	0	0	0
dist_mid_back_to_right_ear_p90	pose geometry	0	0	0
dist_mid_back_to_right_ear_periodicity	pose geometry	0	0	0
dist_mid_back_to_right_ear_std	kinematics	0	0	0
dist_mid_back_to_right_ear_trend	pose geometry	0	0	0
dist_mid_back_to_tail_base_delta	kinematics	0	0	0
dist_mid_back_to_tail_base_energy	kinematics	0	0	0
dist_mid_back_to_tail_base_max	pose geometry	0	0	0
dist_mid_back_to_tail_base_mean	pose geometry	0	0	0
dist_mid_back_to_tail_base_median	pose geometry	0	0	0
dist_mid_back_to_tail_base_norm_delta	kinematics	0	0	0
dist_mid_back_to_tail_base_norm_energy	kinematics	0	0	0
dist_mid_back_to_tail_base_norm_max	pose geometry	0	0	0
dist_mid_back_to_tail_base_norm_mean	pose geometry	0	0	0
dist_mid_back_to_tail_base_norm_median	pose geometry	0	0	0
dist_mid_back_to_tail_base_norm_p10	pose geometry	0	0	0
dist_mid_back_to_tail_base_norm_p90	pose geometry	0	0	0
dist_mid_back_to_tail_base_norm_periodicity	pose geometry	0	0	0
dist_mid_back_to_tail_base_norm_std	kinematics	0	0	0
dist_mid_back_to_tail_base_norm_trend	pose geometry	0	0	0
dist_mid_back_to_tail_base_p10	pose geometry	0	0	0

dist_mid_back_to_tail_base_p90	pose geometry	0	0	0
dist_mid_back_to_tail_base_periodicity	pose geometry	0	0	0
dist_mid_back_to_tail_base_std	kinematics	0	0	0
dist_mid_back_to_tail_base_trend	pose geometry	0	0	0
dist_nose_to_right_body_delta	kinematics	1	0	0
dist_nose_to_right_body_energy	kinematics	1	0	0
dist_nose_to_right_body_max	pose geometry	1	0	0
dist_nose_to_right_body_mean	pose geometry	1	0	0
dist_nose_to_right_body_median	pose geometry	1	0	0
dist_nose_to_right_body_norm_delta	kinematics	1	0	0
dist_nose_to_right_body_norm_energy	kinematics	1	0	0
dist_nose_to_right_body_norm_max	pose geometry	1	0	0
dist_nose_to_right_body_norm_mean	pose geometry	1	0	0
dist_nose_to_right_body_norm_median	pose geometry	1	0	0
dist_nose_to_right_body_norm_p10	pose geometry	1	0	0
dist_nose_to_right_body_norm_p90	pose geometry	1	0	0
dist_nose_to_right_body_norm_periodicity	pose geometry	1	0	0
dist_nose_to_right_body_norm_std	kinematics	1	0	0
dist_nose_to_right_body_norm_trend	pose geometry	1	0	0
dist_nose_to_right_body_p10	pose geometry	1	0	0
dist_nose_to_right_body_p90	pose geometry	1	0	0
dist_nose_to_right_body_periodicity	pose geometry	1	0	0
dist_nose_to_right_body_std	kinematics	1	0	0
dist_nose_to_right_body_trend	pose geometry	1	0	0
dist_nose_to_right_ear_delta	kinematics	1	0	0
dist_nose_to_right_ear_energy	kinematics	1	0	0
dist_nose_to_right_ear_max	pose geometry	1	0	0
dist_nose_to_right_ear_mean	pose geometry	1	0	0
dist_nose_to_right_ear_median	pose geometry	1	0	0
dist_nose_to_right_ear_norm_delta	kinematics	1	0	0
dist_nose_to_right_ear_norm_energy	kinematics	1	0	0
dist_nose_to_right_ear_norm_max	pose geometry	1	0	0
dist_nose_to_right_ear_norm_mean	pose geometry	1	0	0
dist_nose_to_right_ear_norm_median	pose geometry	1	0	0
dist_nose_to_right_ear_norm_p10	pose geometry	1	0	0
dist_nose_to_right_ear_norm_p90	pose geometry	1	0	0
dist_nose_to_right_ear_norm_periodicity	pose geometry	1	0	0
dist_nose_to_right_ear_norm_std	kinematics	1	0	0
dist_nose_to_right_ear_norm_trend	pose geometry	1	0	0
dist_nose_to_right_ear_p10	pose geometry	1	0	0
dist_nose_to_right_ear_p90	pose geometry	1	0	0
dist_nose_to_right_ear_periodicity	pose geometry	1	0	0
dist_nose_to_right_ear_std	kinematics	1	0	0
dist_nose_to_right_ear_trend	pose geometry	1	0	0

dist_nose_to_tail_base_delta	kinematics	1	1	1
dist_nose_to_tail_base_energy	kinematics	1	1	1
dist_nose_to_tail_base_max	pose geometry	1	1	1
dist_nose_to_tail_base_mean	pose geometry	1	1	1
dist_nose_to_tail_base_median	pose geometry	1	1	1
dist_nose_to_tail_base_p10	pose geometry	1	1	1
dist_nose_to_tail_base_p90	pose geometry	1	1	1
dist_nose_to_tail_base_periodicity	pose geometry	1	1	1
dist_nose_to_tail_base_std	kinematics	1	1	1
dist_nose_to_tail_base_trend	pose geometry	1	1	1
dist_right_body_to_right_ear_delta	kinematics	1	0	0
dist_right_body_to_right_ear_energy	kinematics	1	0	0
dist_right_body_to_right_ear_max	pose geometry	1	0	0
dist_right_body_to_right_ear_mean	pose geometry	1	0	0
dist_right_body_to_right_ear_median	pose geometry	1	0	0
dist_right_body_to_right_ear_norm_delta	kinematics	1	0	0
dist_right_body_to_right_ear_norm_energy	kinematics	1	0	0
dist_right_body_to_right_ear_norm_max	pose geometry	1	0	0
dist_right_body_to_right_ear_norm_mean	pose geometry	1	0	0
dist_right_body_to_right_ear_norm_median	pose geometry	1	0	0
dist_right_body_to_right_ear_norm_p10	pose geometry	1	0	0
dist_right_body_to_right_ear_norm_p90	pose geometry	1	0	0
dist_right_body_to_right_ear_norm_periodicity	pose geometry	1	0	0
dist_right_body_to_right_ear_norm_std	kinematics	1	0	0
dist_right_body_to_right_ear_norm_trend	pose geometry	1	0	0
dist_right_body_to_right_ear_p10	pose geometry	1	0	0
dist_right_body_to_right_ear_p90	pose geometry	1	0	0
dist_right_body_to_right_ear_periodicity	pose geometry	1	0	0
dist_right_body_to_right_ear_std	kinematics	1	0	0
dist_right_body_to_right_ear_trend	pose geometry	1	0	0
dist_right_body_to_tail_base_delta	kinematics	1	0	0
dist_right_body_to_tail_base_energy	kinematics	1	0	0
dist_right_body_to_tail_base_max	pose geometry	1	0	0
dist_right_body_to_tail_base_mean	pose geometry	1	0	0
dist_right_body_to_tail_base_median	pose geometry	1	0	0
dist_right_body_to_tail_base_norm_delta	kinematics	1	0	0
dist_right_body_to_tail_base_norm_energy	kinematics	1	0	0
dist_right_body_to_tail_base_norm_max	pose geometry	1	0	0
dist_right_body_to_tail_base_norm_mean	pose geometry	1	0	0
dist_right_body_to_tail_base_norm_median	pose geometry	1	0	0
dist_right_body_to_tail_base_norm_p10	pose geometry	1	0	0
dist_right_body_to_tail_base_norm_p90	pose geometry	1	0	0
dist_right_body_to_tail_base_norm_periodicity	pose geometry	1	0	0
dist_right_body_to_tail_base_norm_std	kinematics	1	0	0

dist_right_body_to_tail_base_norm_trend	pose geometry	1	0	0
dist_right_body_to_tail_base_p10	pose geometry	1	0	0
dist_right_body_to_tail_base_p90	pose geometry	1	0	0
dist_right_body_to_tail_base_periodicity	pose geometry	1	0	0
dist_right_body_to_tail_base_std	kinematics	1	0	0
dist_right_body_to_tail_base_trend	pose geometry	1	0	0
dist_right_ear_to_tail_base_delta	kinematics	1	0	0
dist_right_ear_to_tail_base_energy	kinematics	1	0	0
dist_right_ear_to_tail_base_max	pose geometry	1	0	0
dist_right_ear_to_tail_base_mean	pose geometry	1	0	0
dist_right_ear_to_tail_base_median	pose geometry	1	0	0
dist_right_ear_to_tail_base_norm_delta	kinematics	1	0	0
dist_right_ear_to_tail_base_norm_energy	kinematics	1	0	0
dist_right_ear_to_tail_base_norm_max	pose geometry	1	0	0
dist_right_ear_to_tail_base_norm_mean	pose geometry	1	0	0
dist_right_ear_to_tail_base_norm_median	pose geometry	1	0	0
dist_right_ear_to_tail_base_norm_p10	pose geometry	1	0	0
dist_right_ear_to_tail_base_norm_p90	pose geometry	1	0	0
dist_right_ear_to_tail_base_norm_periodicity	pose geometry	1	0	0
dist_right_ear_to_tail_base_norm_std	kinematics	1	0	0
dist_right_ear_to_tail_base_norm_trend	pose geometry	1	0	0
dist_right_ear_to_tail_base_p10	pose geometry	1	0	0
dist_right_ear_to_tail_base_p90	pose geometry	1	0	0
dist_right_ear_to_tail_base_periodicity	pose geometry	1	0	0
dist_right_ear_to_tail_base_std	kinematics	1	0	0
dist_right_ear_to_tail_base_trend	pose geometry	1	0	0
ear_left_acceleration_energy	kinematics	0	1	1
ear_left_acceleration_max	kinematics	0	1	1
ear_left_acceleration_mean	kinematics	0	1	1
ear_left_acceleration_median	kinematics	0	1	1
ear_left_acceleration_p10	kinematics	0	1	1
ear_left_acceleration_p90	kinematics	0	1	1
ear_left_acceleration_periodicity	kinematics	0	1	1
ear_left_acceleration_std	kinematics	0	1	1
ear_left_forward_velocity_energy	kinematics	0	1	1
ear_left_forward_velocity_max	kinematics	0	1	1
ear_left_forward_velocity_mean	kinematics	0	1	1
ear_left_forward_velocity_median	kinematics	0	1	1
ear_left_forward_velocity_p10	kinematics	0	1	1
ear_left_forward_velocity_p90	kinematics	0	1	1
ear_left_forward_velocity_periodicity	kinematics	0	1	1
ear_left_forward_velocity_std	kinematics	0	1	1
ear_left_jerk_energy	kinematics	0	1	1
ear_left_jerk_max	kinematics	0	1	1

ear_left_jerk_mean	kinematics	0	1	1
ear_left_jerk_median	kinematics	0	1	1
ear_left_jerk_p10	kinematics	0	1	1
ear_left_jerk_p90	kinematics	0	1	1
ear_left_jerk_periodicity	kinematics	0	1	1
ear_left_jerk_std	kinematics	0	1	1
ear_left_lateral_velocity_energy	kinematics	0	1	1
ear_left_lateral_velocity_max	kinematics	0	1	1
ear_left_lateral_velocity_mean	kinematics	0	1	1
ear_left_lateral_velocity_median	kinematics	0	1	1
ear_left_lateral_velocity_p10	kinematics	0	1	1
ear_left_lateral_velocity_p90	kinematics	0	1	1
ear_left_lateral_velocity_periodicity	kinematics	0	1	1
ear_left_lateral_velocity_std	kinematics	0	1	1
ear_left_speed_energy	kinematics	0	1	1
ear_left_speed_max	kinematics	0	1	1
ear_left_speed_mean	kinematics	0	1	1
ear_left_speed_median	kinematics	0	1	1
ear_left_speed_p10	kinematics	0	1	1
ear_left_speed_p90	kinematics	0	1	1
ear_left_speed_periodicity	kinematics	0	1	1
ear_left_speed_std	kinematics	0	1	1
ear_left_velocity_x_energy	kinematics	0	1	1
ear_left_velocity_x_max	kinematics	0	1	1
ear_left_velocity_x_mean	kinematics	0	1	1
ear_left_velocity_x_median	kinematics	0	1	1
ear_left_velocity_x_p10	kinematics	0	1	1
ear_left_velocity_x_p90	kinematics	0	1	1
ear_left_velocity_x_periodicity	kinematics	0	1	1
ear_left_velocity_x_std	kinematics	0	1	1
ear_left_velocity_y_energy	kinematics	0	1	1
ear_left_velocity_y_max	kinematics	0	1	1
ear_left_velocity_y_mean	kinematics	0	1	1
ear_left_velocity_y_median	kinematics	0	1	1
ear_left_velocity_y_p10	kinematics	0	1	1
ear_left_velocity_y_p90	kinematics	0	1	1
ear_left_velocity_y_periodicity	kinematics	0	1	1
ear_left_velocity_y_std	kinematics	0	1	1
ear_right_acceleration_energy	kinematics	0	1	1
ear_right_acceleration_max	kinematics	0	1	1
ear_right_acceleration_mean	kinematics	0	1	1
ear_right_acceleration_median	kinematics	0	1	1
ear_right_acceleration_p10	kinematics	0	1	1
ear_right_acceleration_p90	kinematics	0	1	1

ear_right_acceleration_periodicity	kinematics	0	1	1
ear_right_acceleration_std	kinematics	0	1	1
ear_right_forward_velocity_energy	kinematics	0	1	1
ear_right_forward_velocity_max	kinematics	0	1	1
ear_right_forward_velocity_mean	kinematics	0	1	1
ear_right_forward_velocity_median	kinematics	0	1	1
ear_right_forward_velocity_p10	kinematics	0	1	1
ear_right_forward_velocity_p90	kinematics	0	1	1
ear_right_forward_velocity_periodicity	kinematics	0	1	1
ear_right_forward_velocity_std	kinematics	0	1	1
ear_right_jerk_energy	kinematics	0	1	1
ear_right_jerk_max	kinematics	0	1	1
ear_right_jerk_mean	kinematics	0	1	1
ear_right_jerk_median	kinematics	0	1	1
ear_right_jerk_p10	kinematics	0	1	1
ear_right_jerk_p90	kinematics	0	1	1
ear_right_jerk_periodicity	kinematics	0	1	1
ear_right_jerk_std	kinematics	0	1	1
ear_right_lateral_velocity_energy	kinematics	0	1	1
ear_right_lateral_velocity_max	kinematics	0	1	1
ear_right_lateral_velocity_mean	kinematics	0	1	1
ear_right_lateral_velocity_median	kinematics	0	1	1
ear_right_lateral_velocity_p10	kinematics	0	1	1
ear_right_lateral_velocity_p90	kinematics	0	1	1
ear_right_lateral_velocity_periodicity	kinematics	0	1	1
ear_right_lateral_velocity_std	kinematics	0	1	1
ear_right_speed_energy	kinematics	0	1	1
ear_right_speed_max	kinematics	0	1	1
ear_right_speed_mean	kinematics	0	1	1
ear_right_speed_median	kinematics	0	1	1
ear_right_speed_p10	kinematics	0	1	1
ear_right_speed_p90	kinematics	0	1	1
ear_right_speed_periodicity	kinematics	0	1	1
ear_right_speed_std	kinematics	0	1	1
ear_right_velocity_x_energy	kinematics	0	1	1
ear_right_velocity_x_max	kinematics	0	1	1
ear_right_velocity_x_mean	kinematics	0	1	1
ear_right_velocity_x_median	kinematics	0	1	1
ear_right_velocity_x_p10	kinematics	0	1	1
ear_right_velocity_x_p90	kinematics	0	1	1
ear_right_velocity_x_periodicity	kinematics	0	1	1
ear_right_velocity_x_std	kinematics	0	1	1
ear_right_velocity_y_energy	kinematics	0	1	1
ear_right_velocity_y_max	kinematics	0	1	1

ear_right_velocity_y_mean	kinematics	0	1	1
ear_right_velocity_y_median	kinematics	0	1	1
ear_right_velocity_y_p10	kinematics	0	1	1
ear_right_velocity_y_p90	kinematics	0	1	1
ear_right_velocity_y_periodicity	kinematics	0	1	1
ear_right_velocity_y_std	kinematics	0	1	1
ear_spread_energy	kinematics	1	1	1
ear_spread_max	pose geometry	1	1	1
ear_spread_mean	pose geometry	1	1	1
ear_spread_median	pose geometry	1	1	1
ear_spread_p10	pose geometry	1	1	1
ear_spread_p90	pose geometry	1	1	1
ear_spread_periodicity	pose geometry	1	1	1
ear_spread_std	kinematics	1	1	1
flow_directionality_paw_energy	video (motion/flow)	1	1	1
flow_directionality_paw_max	video (motion/flow)	1	1	1
flow_directionality_paw_mean	video (motion/flow)	1	1	1
flow_directionality_paw_median	video (motion/flow)	1	1	1
flow_directionality_paw_p10	video (motion/flow)	1	1	1
flow_directionality_paw_p90	video (motion/flow)	1	1	1
flow_directionality_paw_periodicity	video (motion/flow)	1	1	1
flow_directionality_paw_std	video (motion/flow)	1	1	1
flow_entropy_local_energy	video (motion/flow)	1	1	1
flow_entropy_local_max	video (motion/flow)	1	1	1
flow_entropy_local_mean	video (motion/flow)	1	1	1
flow_entropy_local_median	video (motion/flow)	1	1	1
flow_entropy_local_p10	video (motion/flow)	1	1	1
flow_entropy_local_p90	video (motion/flow)	1	1	1
flow_entropy_local_periodicity	video (motion/flow)	1	1	1
flow_entropy_local_std	video (motion/flow)	1	1	1
flow_mag_near_nose_energy	video (motion/flow)	1	1	1
flow_mag_near_nose_max	video (motion/flow)	1	1	1
flow_mag_near_nose_mean	video (motion/flow)	1	1	1
flow_mag_near_nose_median	video (motion/flow)	1	1	1
flow_mag_near_nose_p10	video (motion/flow)	1	1	1
flow_mag_near_nose_p90	video (motion/flow)	1	1	1
flow_mag_near_nose_periodicity	video (motion/flow)	1	1	1
flow_mag_near_nose_std	video (motion/flow)	1	1	1
flow_mag_near_roi_1_energy	video (motion/flow)	1	1	1
flow_mag_near_roi_1_max	video (motion/flow)	1	1	1
flow_mag_near_roi_1_mean	video (motion/flow)	1	1	1
flow_mag_near_roi_1_median	video (motion/flow)	1	1	1
flow_mag_near_roi_1_p10	video (motion/flow)	1	1	1
flow_mag_near_roi_1_p90	video (motion/flow)	1	1	1

flow_mag_near_roi_1_periodicity	video (motion/flow)	1	1	1
flow_mag_near_roi_1_std	video (motion/flow)	1	1	1
flow_mag_near_roi_2_energy	video (motion/flow)	1	0	0
flow_mag_near_roi_2_max	video (motion/flow)	1	0	0
flow_mag_near_roi_2_mean	video (motion/flow)	1	0	0
flow_mag_near_roi_2_median	video (motion/flow)	1	0	0
flow_mag_near_roi_2_p10	video (motion/flow)	1	0	0
flow_mag_near_roi_2_p90	video (motion/flow)	1	0	0
flow_mag_near_roi_2_periodicity	video (motion/flow)	1	0	0
flow_mag_near_roi_2_std	video (motion/flow)	1	0	0
flow_mag_near_target_energy	video (motion/flow)	1	1	1
flow_mag_near_target_max	video (motion/flow)	1	1	1
flow_mag_near_target_mean	video (motion/flow)	1	1	1
flow_mag_near_target_median	video (motion/flow)	1	1	1
flow_mag_near_target_p10	video (motion/flow)	1	1	1
flow_mag_near_target_p90	video (motion/flow)	1	1	1
flow_mag_near_target_periodicity	video (motion/flow)	1	1	1
flow_mag_near_target_std	video (motion/flow)	1	1	1
flow_mag_paw_L_energy	video (motion/flow)	1	1	1
flow_mag_paw_L_max	video (motion/flow)	1	1	1
flow_mag_paw_L_mean	video (motion/flow)	1	1	1
flow_mag_paw_L_median	video (motion/flow)	1	1	1
flow_mag_paw_L_p10	video (motion/flow)	1	1	1
flow_mag_paw_L_p90	video (motion/flow)	1	1	1
flow_mag_paw_L_periodicity	video (motion/flow)	1	1	1
flow_mag_paw_L_std	video (motion/flow)	1	1	1
flow_mag_paw_R_energy	video (motion/flow)	1	1	1
flow_mag_paw_R_max	video (motion/flow)	1	1	1
flow_mag_paw_R_mean	video (motion/flow)	1	1	1
flow_mag_paw_R_median	video (motion/flow)	1	1	1
flow_mag_paw_R_p10	video (motion/flow)	1	1	1
flow_mag_paw_R_p90	video (motion/flow)	1	1	1
flow_mag_paw_R_periodicity	video (motion/flow)	1	1	1
flow_mag_paw_R_std	video (motion/flow)	1	1	1
forepaw_autocorr_peak_energy	kinematics	1	1	1
forepaw_autocorr_peak_max	pose geometry	1	1	1
forepaw_autocorr_peak_mean	pose geometry	1	1	1
forepaw_autocorr_peak_median	pose geometry	1	1	1
forepaw_autocorr_peak_p10	pose geometry	1	1	1
forepaw_autocorr_peak_p90	pose geometry	1	1	1
forepaw_autocorr_peak_periodicity	pose geometry	1	1	1
forepaw_autocorr_peak_std	kinematics	1	1	1
forepaw_centroid_roi_1_axial_abs_energy	context (ROI/target)	1	1	1
forepaw_centroid_roi_1_axial_abs_max	context (ROI/target)	1	1	1

forepaw_centroid_roi_1_axial_abs_mean	context (ROI/target)	1	1	1
forepaw_centroid_roi_1_axial_abs_median	context (ROI/target)	1	1	1
forepaw_centroid_roi_1_axial_abs_p10	context (ROI/target)	1	1	1
forepaw_centroid_roi_1_axial_abs_p90	context (ROI/target)	1	1	1
forepaw_centroid_roi_1_axial_abs_periodicity	context (ROI/target)	1	1	1
forepaw_centroid_roi_1_axial_abs_std	context (ROI/target)	1	1	1
forepaw_centroid_roi_1_axial_energy	context (ROI/target)	1	1	1
forepaw_centroid_roi_1_axial_max	context (ROI/target)	1	1	1
forepaw_centroid_roi_1_axial_mean	context (ROI/target)	1	1	1
forepaw_centroid_roi_1_axial_median	context (ROI/target)	1	1	1
forepaw_centroid_roi_1_axial_p10	context (ROI/target)	1	1	1
forepaw_centroid_roi_1_axial_p90	context (ROI/target)	1	1	1
forepaw_centroid_roi_1_axial_periodicity	context (ROI/target)	1	1	1
forepaw_centroid_roi_1_axial_std	context (ROI/target)	1	1	1
forepaw_centroid_roi_1_lateral_abs_energy	context (ROI/target)	1	1	1
forepaw_centroid_roi_1_lateral_abs_max	context (ROI/target)	1	1	1
forepaw_centroid_roi_1_lateral_abs_mean	context (ROI/target)	1	1	1
forepaw_centroid_roi_1_lateral_abs_median	context (ROI/target)	1	1	1
forepaw_centroid_roi_1_lateral_abs_p10	context (ROI/target)	1	1	1
forepaw_centroid_roi_1_lateral_abs_p90	context (ROI/target)	1	1	1
forepaw_centroid_roi_1_lateral_abs_periodicity	context (ROI/target)	1	1	1
forepaw_centroid_roi_1_lateral_abs_std	context (ROI/target)	1	1	1
forepaw_centroid_roi_1_lateral_energy	context (ROI/target)	1	1	1
forepaw_centroid_roi_1_lateral_max	context (ROI/target)	1	1	1
forepaw_centroid_roi_1_lateral_mean	context (ROI/target)	1	1	1
forepaw_centroid_roi_1_lateral_median	context (ROI/target)	1	1	1
forepaw_centroid_roi_1_lateral_p10	context (ROI/target)	1	1	1
forepaw_centroid_roi_1_lateral_p90	context (ROI/target)	1	1	1
forepaw_centroid_roi_1_lateral_periodicity	context (ROI/target)	1	1	1
forepaw_centroid_roi_1_lateral_std	context (ROI/target)	1	1	1
forepaw_centroid_roi_2_axial_abs_energy	context (ROI/target)	1	0	0
forepaw_centroid_roi_2_axial_abs_max	context (ROI/target)	1	0	0
forepaw_centroid_roi_2_axial_abs_mean	context (ROI/target)	1	0	0
forepaw_centroid_roi_2_axial_abs_median	context (ROI/target)	1	0	0
forepaw_centroid_roi_2_axial_abs_p10	context (ROI/target)	1	0	0
forepaw_centroid_roi_2_axial_abs_p90	context (ROI/target)	1	0	0
forepaw_centroid_roi_2_axial_abs_periodicity	context (ROI/target)	1	0	0
forepaw_centroid_roi_2_axial_abs_std	context (ROI/target)	1	0	0
forepaw_centroid_roi_2_axial_energy	context (ROI/target)	1	0	0
forepaw_centroid_roi_2_axial_max	context (ROI/target)	1	0	0
forepaw_centroid_roi_2_axial_mean	context (ROI/target)	1	0	0
forepaw_centroid_roi_2_axial_median	context (ROI/target)	1	0	0
forepaw_centroid_roi_2_axial_p10	context (ROI/target)	1	0	0
forepaw_centroid_roi_2_axial_p90	context (ROI/target)	1	0	0

forepaw_centroid_roi_2_axial_periodicity	context (ROI/target)	1	0	0
forepaw_centroid_roi_2_axial_std	context (ROI/target)	1	0	0
forepaw_centroid_roi_2_lateral_abs_energy	context (ROI/target)	1	0	0
forepaw_centroid_roi_2_lateral_abs_max	context (ROI/target)	1	0	0
forepaw_centroid_roi_2_lateral_abs_mean	context (ROI/target)	1	0	0
forepaw_centroid_roi_2_lateral_abs_median	context (ROI/target)	1	0	0
forepaw_centroid_roi_2_lateral_abs_p10	context (ROI/target)	1	0	0
forepaw_centroid_roi_2_lateral_abs_p90	context (ROI/target)	1	0	0
forepaw_centroid_roi_2_lateral_abs_periodicity	context (ROI/target)	1	0	0
forepaw_centroid_roi_2_lateral_abs_std	context (ROI/target)	1	0	0
forepaw_centroid_roi_2_lateral_energy	context (ROI/target)	1	0	0
forepaw_centroid_roi_2_lateral_max	context (ROI/target)	1	0	0
forepaw_centroid_roi_2_lateral_mean	context (ROI/target)	1	0	0
forepaw_centroid_roi_2_lateral_median	context (ROI/target)	1	0	0
forepaw_centroid_roi_2_lateral_p10	context (ROI/target)	1	0	0
forepaw_centroid_roi_2_lateral_p90	context (ROI/target)	1	0	0
forepaw_centroid_roi_2_lateral_periodicity	context (ROI/target)	1	0	0
forepaw_centroid_roi_2_lateral_std	context (ROI/target)	1	0	0
forepaw_centroid_to_roi_1_corner_dist_delta	context (ROI/target)	1	1	1
forepaw_centroid_to_roi_1_corner_dist_energy	context (ROI/target)	1	1	1
forepaw_centroid_to_roi_1_corner_dist_max	context (ROI/target)	1	1	1
forepaw_centroid_to_roi_1_corner_dist_mean	context (ROI/target)	1	1	1
forepaw_centroid_to_roi_1_corner_dist_median	context (ROI/target)	1	1	1
forepaw_centroid_to_roi_1_corner_dist_p10	context (ROI/target)	1	1	1
forepaw_centroid_to_roi_1_corner_dist_p90	context (ROI/target)	1	1	1
forepaw_centroid_to_roi_1_corner_dist_periodicity	context (ROI/target)	1	1	1
forepaw_centroid_to_roi_1_corner_dist_std	context (ROI/target)	1	1	1
forepaw_centroid_to_roi_1_corner_dist_trend	context (ROI/target)	1	1	1
forepaw_centroid_to_roi_1_dist_delta	context (ROI/target)	1	1	1
forepaw_centroid_to_roi_1_dist_energy	context (ROI/target)	1	1	1
forepaw_centroid_to_roi_1_dist_max	context (ROI/target)	1	1	1
forepaw_centroid_to_roi_1_dist_mean	context (ROI/target)	1	1	1
forepaw_centroid_to_roi_1_dist_median	context (ROI/target)	1	1	1
forepaw_centroid_to_roi_1_dist_p10	context (ROI/target)	1	1	1
forepaw_centroid_to_roi_1_dist_p90	context (ROI/target)	1	1	1
forepaw_centroid_to_roi_1_dist_periodicity	context (ROI/target)	1	1	1
forepaw_centroid_to_roi_1_dist_std	context (ROI/target)	1	1	1
forepaw_centroid_to_roi_1_dist_trend	context (ROI/target)	1	1	1
forepaw_centroid_to_roi_1_edge_dist_delta	context (ROI/target)	1	1	1
forepaw_centroid_to_roi_1_edge_dist_energy	context (ROI/target)	1	1	1
forepaw_centroid_to_roi_1_edge_dist_max	context (ROI/target)	1	1	1
forepaw_centroid_to_roi_1_edge_dist_mean	context (ROI/target)	1	1	1
forepaw_centroid_to_roi_1_edge_dist_median	context (ROI/target)	1	1	1
forepaw_centroid_to_roi_1_edge_dist_p10	context (ROI/target)	1	1	1

forepaw_centroid_to_roi_1_edge_dist_p90	context (ROI/target)	1	1	1
forepaw_centroid_to_roi_1_edge_dist_periodicity	context (ROI/target)	1	1	1
forepaw_centroid_to_roi_1_edge_dist_std	context (ROI/target)	1	1	1
forepaw_centroid_to_roi_1_edge_dist_trend	context (ROI/target)	1	1	1
forepaw_centroid_to_roi_1_signed_dist_delta	context (ROI/target)	1	1	1
forepaw_centroid_to_roi_1_signed_dist_energy	context (ROI/target)	1	1	1
forepaw_centroid_to_roi_1_signed_dist_max	context (ROI/target)	1	1	1
forepaw_centroid_to_roi_1_signed_dist_mean	context (ROI/target)	1	1	1
forepaw_centroid_to_roi_1_signed_dist_median	context (ROI/target)	1	1	1
forepaw_centroid_to_roi_1_signed_dist_p10	context (ROI/target)	1	1	1
forepaw_centroid_to_roi_1_signed_dist_p90	context (ROI/target)	1	1	1
forepaw_centroid_to_roi_1_signed_dist_periodicity	context (ROI/target)	1	1	1
forepaw_centroid_to_roi_1_signed_dist_std	context (ROI/target)	1	1	1
forepaw_centroid_to_roi_1_signed_dist_trend	context (ROI/target)	1	1	1
forepaw_centroid_to_roi_2_corner_dist_delta	context (ROI/target)	1	0	0
forepaw_centroid_to_roi_2_corner_dist_energy	context (ROI/target)	1	0	0
forepaw_centroid_to_roi_2_corner_dist_max	context (ROI/target)	1	0	0
forepaw_centroid_to_roi_2_corner_dist_mean	context (ROI/target)	1	0	0
forepaw_centroid_to_roi_2_corner_dist_median	context (ROI/target)	1	0	0
forepaw_centroid_to_roi_2_corner_dist_p10	context (ROI/target)	1	0	0
forepaw_centroid_to_roi_2_corner_dist_p90	context (ROI/target)	1	0	0
forepaw_centroid_to_roi_2_corner_dist_periodicity	context (ROI/target)	1	0	0
forepaw_centroid_to_roi_2_corner_dist_std	context (ROI/target)	1	0	0
forepaw_centroid_to_roi_2_corner_dist_trend	context (ROI/target)	1	0	0
forepaw_centroid_to_roi_2_dist_delta	context (ROI/target)	1	0	0
forepaw_centroid_to_roi_2_dist_energy	context (ROI/target)	1	0	0
forepaw_centroid_to_roi_2_dist_max	context (ROI/target)	1	0	0
forepaw_centroid_to_roi_2_dist_mean	context (ROI/target)	1	0	0
forepaw_centroid_to_roi_2_dist_median	context (ROI/target)	1	0	0
forepaw_centroid_to_roi_2_dist_p10	context (ROI/target)	1	0	0
forepaw_centroid_to_roi_2_dist_p90	context (ROI/target)	1	0	0
forepaw_centroid_to_roi_2_dist_periodicity	context (ROI/target)	1	0	0
forepaw_centroid_to_roi_2_dist_std	context (ROI/target)	1	0	0
forepaw_centroid_to_roi_2_dist_trend	context (ROI/target)	1	0	0
forepaw_centroid_to_roi_2_edge_dist_delta	context (ROI/target)	1	0	0
forepaw_centroid_to_roi_2_edge_dist_energy	context (ROI/target)	1	0	0
forepaw_centroid_to_roi_2_edge_dist_max	context (ROI/target)	1	0	0
forepaw_centroid_to_roi_2_edge_dist_mean	context (ROI/target)	1	0	0
forepaw_centroid_to_roi_2_edge_dist_median	context (ROI/target)	1	0	0
forepaw_centroid_to_roi_2_edge_dist_p10	context (ROI/target)	1	0	0
forepaw_centroid_to_roi_2_edge_dist_p90	context (ROI/target)	1	0	0
forepaw_centroid_to_roi_2_edge_dist_periodicity	context (ROI/target)	1	0	0
forepaw_centroid_to_roi_2_edge_dist_std	context (ROI/target)	1	0	0
forepaw_centroid_to_roi_2_edge_dist_trend	context (ROI/target)	1	0	0

forepaw_centroid_to_roi_2_signed_dist_delta	context (ROI/target)	1	0	0
forepaw_centroid_to_roi_2_signed_dist_energy	context (ROI/target)	1	0	0
forepaw_centroid_to_roi_2_signed_dist_max	context (ROI/target)	1	0	0
forepaw_centroid_to_roi_2_signed_dist_mean	context (ROI/target)	1	0	0
forepaw_centroid_to_roi_2_signed_dist_median	context (ROI/target)	1	0	0
forepaw_centroid_to_roi_2_signed_dist_p10	context (ROI/target)	1	0	0
forepaw_centroid_to_roi_2_signed_dist_p90	context (ROI/target)	1	0	0
forepaw_centroid_to_roi_2_signed_dist_periodicity	context (ROI/target)	1	0	0
forepaw_centroid_to_roi_2_signed_dist_std	context (ROI/target)	1	0	0
forepaw_centroid_to_roi_2_signed_dist_trend	context (ROI/target)	1	0	0
forepaw_centroid_to_target_dist_delta	context (ROI/target)	1	1	1
forepaw_centroid_to_target_dist_energy	context (ROI/target)	1	1	1
forepaw_centroid_to_target_dist_max	context (ROI/target)	1	1	1
forepaw_centroid_to_target_dist_mean	context (ROI/target)	1	1	1
forepaw_centroid_to_target_dist_median	context (ROI/target)	1	1	1
forepaw_centroid_to_target_dist_p10	context (ROI/target)	1	1	1
forepaw_centroid_to_target_dist_p90	context (ROI/target)	1	1	1
forepaw_centroid_to_target_dist_periodicity	context (ROI/target)	1	1	1
forepaw_centroid_to_target_dist_std	context (ROI/target)	1	1	1
forepaw_centroid_to_target_dist_trend	context (ROI/target)	1	1	1
forepaw_movement_frequency_energy	kinematics	1	1	1
forepaw_movement_frequency_max	kinematics	1	1	1
forepaw_movement_frequency_mean	kinematics	1	1	1
forepaw_movement_frequency_median	kinematics	1	1	1
forepaw_movement_frequency_p10	kinematics	1	1	1
forepaw_movement_frequency_p90	kinematics	1	1	1
forepaw_movement_frequency_periodicity	kinematics	1	1	1
forepaw_movement_frequency_std	kinematics	1	1	1
forepaw_oscillation_power_energy	kinematics	1	1	1
forepaw_oscillation_power_max	pose geometry	1	1	1
forepaw_oscillation_power_mean	pose geometry	1	1	1
forepaw_oscillation_power_median	pose geometry	1	1	1
forepaw_oscillation_power_p10	pose geometry	1	1	1
forepaw_oscillation_power_p90	pose geometry	1	1	1
forepaw_oscillation_power_periodicity	pose geometry	1	1	1
forepaw_oscillation_power_std	kinematics	1	1	1
forepaw_speed_energy	kinematics	1	1	1
forepaw_speed_max	kinematics	1	1	1
forepaw_speed_mean	kinematics	1	1	1
forepaw_speed_median	kinematics	1	1	1
forepaw_speed_p10	kinematics	1	1	1
forepaw_speed_p90	kinematics	1	1	1
forepaw_speed_periodicity	kinematics	1	1	1
forepaw_speed_std	kinematics	1	1	1

forepaw_vertical_velocity_energy	kinematics	1	1	1
forepaw_vertical_velocity_max	kinematics	1	1	1
forepaw_vertical_velocity_mean	kinematics	1	1	1
forepaw_vertical_velocity_median	kinematics	1	1	1
forepaw_vertical_velocity_p10	kinematics	1	1	1
forepaw_vertical_velocity_p90	kinematics	1	1	1
forepaw_vertical_velocity_periodicity	kinematics	1	1	1
forepaw_vertical_velocity_std	kinematics	1	1	1
front_left_paw_acceleration_energy	kinematics	0	0	0
front_left_paw_acceleration_max	kinematics	0	0	0
front_left_paw_acceleration_mean	kinematics	0	0	0
front_left_paw_acceleration_median	kinematics	0	0	0
front_left_paw_acceleration_p10	kinematics	0	0	0
front_left_paw_acceleration_p90	kinematics	0	0	0
front_left_paw_acceleration_periodicity	kinematics	0	0	0
front_left_paw_acceleration_std	kinematics	0	0	0
front_left_paw_forward_velocity_energy	kinematics	0	0	0
front_left_paw_forward_velocity_max	kinematics	0	0	0
front_left_paw_forward_velocity_mean	kinematics	0	0	0
front_left_paw_forward_velocity_median	kinematics	0	0	0
front_left_paw_forward_velocity_p10	kinematics	0	0	0
front_left_paw_forward_velocity_p90	kinematics	0	0	0
front_left_paw_forward_velocity_periodicity	kinematics	0	0	0
front_left_paw_forward_velocity_std	kinematics	0	0	0
front_left_paw_jerk_energy	kinematics	0	0	0
front_left_paw_jerk_max	kinematics	0	0	0
front_left_paw_jerk_mean	kinematics	0	0	0
front_left_paw_jerk_median	kinematics	0	0	0
front_left_paw_jerk_p10	kinematics	0	0	0
front_left_paw_jerk_p90	kinematics	0	0	0
front_left_paw_jerk_periodicity	kinematics	0	0	0
front_left_paw_jerk_std	kinematics	0	0	0
front_left_paw_lateral_velocity_energy	kinematics	0	0	0
front_left_paw_lateral_velocity_max	kinematics	0	0	0
front_left_paw_lateral_velocity_mean	kinematics	0	0	0
front_left_paw_lateral_velocity_median	kinematics	0	0	0
front_left_paw_lateral_velocity_p10	kinematics	0	0	0
front_left_paw_lateral_velocity_p90	kinematics	0	0	0
front_left_paw_lateral_velocity_periodicity	kinematics	0	0	0
front_left_paw_lateral_velocity_std	kinematics	0	0	0
front_left_paw_speed_energy	kinematics	0	0	0
front_left_paw_speed_max	kinematics	0	0	0
front_left_paw_speed_mean	kinematics	0	0	0
front_left_paw_speed_median	kinematics	0	0	0

front_left_paw_speed_p10	kinematics	0	0	0
front_left_paw_speed_p90	kinematics	0	0	0
front_left_paw_speed_periodicity	kinematics	0	0	0
front_left_paw_speed_std	kinematics	0	0	0
front_left_paw_velocity_x_energy	kinematics	0	0	0
front_left_paw_velocity_x_max	kinematics	0	0	0
front_left_paw_velocity_x_mean	kinematics	0	0	0
front_left_paw_velocity_x_median	kinematics	0	0	0
front_left_paw_velocity_x_p10	kinematics	0	0	0
front_left_paw_velocity_x_p90	kinematics	0	0	0
front_left_paw_velocity_x_periodicity	kinematics	0	0	0
front_left_paw_velocity_x_std	kinematics	0	0	0
front_left_paw_velocity_y_energy	kinematics	0	0	0
front_left_paw_velocity_y_max	kinematics	0	0	0
front_left_paw_velocity_y_mean	kinematics	0	0	0
front_left_paw_velocity_y_median	kinematics	0	0	0
front_left_paw_velocity_y_p10	kinematics	0	0	0
front_left_paw_velocity_y_p90	kinematics	0	0	0
front_left_paw_velocity_y_periodicity	kinematics	0	0	0
front_left_paw_velocity_y_std	kinematics	0	0	0
front_right_paw_acceleration_energy	kinematics	0	0	0
front_right_paw_acceleration_max	kinematics	0	0	0
front_right_paw_acceleration_mean	kinematics	0	0	0
front_right_paw_acceleration_median	kinematics	0	0	0
front_right_paw_acceleration_p10	kinematics	0	0	0
front_right_paw_acceleration_p90	kinematics	0	0	0
front_right_paw_acceleration_periodicity	kinematics	0	0	0
front_right_paw_acceleration_std	kinematics	0	0	0
front_right_paw_forward_velocity_energy	kinematics	0	0	0
front_right_paw_forward_velocity_max	kinematics	0	0	0
front_right_paw_forward_velocity_mean	kinematics	0	0	0
front_right_paw_forward_velocity_median	kinematics	0	0	0
front_right_paw_forward_velocity_p10	kinematics	0	0	0
front_right_paw_forward_velocity_p90	kinematics	0	0	0
front_right_paw_forward_velocity_periodicity	kinematics	0	0	0
front_right_paw_forward_velocity_std	kinematics	0	0	0
front_right_paw_jerk_energy	kinematics	0	0	0
front_right_paw_jerk_max	kinematics	0	0	0
front_right_paw_jerk_mean	kinematics	0	0	0
front_right_paw_jerk_median	kinematics	0	0	0
front_right_paw_jerk_p10	kinematics	0	0	0
front_right_paw_jerk_p90	kinematics	0	0	0
front_right_paw_jerk_periodicity	kinematics	0	0	0
front_right_paw_jerk_std	kinematics	0	0	0

front_right_paw_lateral_velocity_energy	kinematics	0	0	0
front_right_paw_lateral_velocity_max	kinematics	0	0	0
front_right_paw_lateral_velocity_mean	kinematics	0	0	0
front_right_paw_lateral_velocity_median	kinematics	0	0	0
front_right_paw_lateral_velocity_p10	kinematics	0	0	0
front_right_paw_lateral_velocity_p90	kinematics	0	0	0
front_right_paw_lateral_velocity_periodicity	kinematics	0	0	0
front_right_paw_lateral_velocity_std	kinematics	0	0	0
front_right_paw_speed_energy	kinematics	0	0	0
front_right_paw_speed_max	kinematics	0	0	0
front_right_paw_speed_mean	kinematics	0	0	0
front_right_paw_speed_median	kinematics	0	0	0
front_right_paw_speed_p10	kinematics	0	0	0
front_right_paw_speed_p90	kinematics	0	0	0
front_right_paw_speed_periodicity	kinematics	0	0	0
front_right_paw_speed_std	kinematics	0	0	0
front_right_paw_velocity_x_energy	kinematics	0	0	0
front_right_paw_velocity_x_max	kinematics	0	0	0
front_right_paw_velocity_x_mean	kinematics	0	0	0
front_right_paw_velocity_x_median	kinematics	0	0	0
front_right_paw_velocity_x_p10	kinematics	0	0	0
front_right_paw_velocity_x_p90	kinematics	0	0	0
front_right_paw_velocity_x_periodicity	kinematics	0	0	0
front_right_paw_velocity_x_std	kinematics	0	0	0
front_right_paw_velocity_y_energy	kinematics	0	0	0
front_right_paw_velocity_y_max	kinematics	0	0	0
front_right_paw_velocity_y_mean	kinematics	0	0	0
front_right_paw_velocity_y_median	kinematics	0	0	0
front_right_paw_velocity_y_p10	kinematics	0	0	0
front_right_paw_velocity_y_p90	kinematics	0	0	0
front_right_paw_velocity_y_periodicity	kinematics	0	0	0
front_right_paw_velocity_y_std	kinematics	0	0	0
head_angle_to_roi_1_delta	context (ROI/target)	1	1	1
head_angle_to_roi_1_energy	context (ROI/target)	1	1	1
head_angle_to_roi_1_max	context (ROI/target)	1	1	1
head_angle_to_roi_1_mean	context (ROI/target)	1	1	1
head_angle_to_roi_1_median	context (ROI/target)	1	1	1
head_angle_to_roi_1_p10	context (ROI/target)	1	1	1
head_angle_to_roi_1_p90	context (ROI/target)	1	1	1
head_angle_to_roi_1_periodicity	context (ROI/target)	1	1	1
head_angle_to_roi_1_std	context (ROI/target)	1	1	1
head_angle_to_roi_1_trend	context (ROI/target)	1	1	1
head_angle_to_roi_2_delta	context (ROI/target)	1	0	0
head_angle_to_roi_2_energy	context (ROI/target)	1	0	0

head_angle_to_roi_2_max	context (ROI/target)	1	0	0
head_angle_to_roi_2_mean	context (ROI/target)	1	0	0
head_angle_to_roi_2_median	context (ROI/target)	1	0	0
head_angle_to_roi_2_p10	context (ROI/target)	1	0	0
head_angle_to_roi_2_p90	context (ROI/target)	1	0	0
head_angle_to_roi_2_periodicity	context (ROI/target)	1	0	0
head_angle_to_roi_2_std	context (ROI/target)	1	0	0
head_angle_to_roi_2_trend	context (ROI/target)	1	0	0
head_angle_to_target_delta	context (ROI/target)	1	1	1
head_angle_to_target_energy	context (ROI/target)	1	1	1
head_angle_to_target_max	context (ROI/target)	1	1	1
head_angle_to_target_mean	context (ROI/target)	1	1	1
head_angle_to_target_median	context (ROI/target)	1	1	1
head_angle_to_target_p10	context (ROI/target)	1	1	1
head_angle_to_target_p90	context (ROI/target)	1	1	1
head_angle_to_target_periodicity	context (ROI/target)	1	1	1
head_angle_to_target_std	context (ROI/target)	1	1	1
head_angle_to_target_trend	context (ROI/target)	1	1	1
head_angular_velocity_energy	kinematics	1	1	1
head_angular_velocity_max	kinematics	1	1	1
head_angular_velocity_mean	kinematics	1	1	1
head_angular_velocity_median	kinematics	1	1	1
head_angular_velocity_p10	kinematics	1	1	1
head_angular_velocity_p90	kinematics	1	1	1
head_angular_velocity_periodicity	kinematics	1	1	1
head_angular_velocity_std	kinematics	1	1	1
head_direction_angle_delta	kinematics	1	1	1
head_direction_angle_energy	kinematics	1	1	1
head_direction_angle_max	pose geometry	1	1	1
head_direction_angle_mean	pose geometry	1	1	1
head_direction_angle_median	pose geometry	1	1	1
head_direction_angle_p10	pose geometry	1	1	1
head_direction_angle_p90	pose geometry	1	1	1
head_direction_angle_periodicity	pose geometry	1	1	1
head_direction_angle_std	kinematics	1	1	1
head_direction_angle_trend	pose geometry	1	1	1
head_forward_speed_energy	kinematics	1	1	1
head_forward_speed_max	kinematics	1	1	1
head_forward_speed_mean	kinematics	1	1	1
head_forward_speed_median	kinematics	1	1	1
head_forward_speed_p10	kinematics	1	1	1
head_forward_speed_p90	kinematics	1	1	1
head_forward_speed_periodicity	kinematics	1	1	1
head_forward_speed_std	kinematics	1	1	1

head_lateral_speed_energy	kinematics	1	1	1
head_lateral_speed_max	kinematics	1	1	1
head_lateral_speed_mean	kinematics	1	1	1
head_lateral_speed_median	kinematics	1	1	1
head_lateral_speed_p10	kinematics	1	1	1
head_lateral_speed_p90	kinematics	1	1	1
head_lateral_speed_periodicity	kinematics	1	1	1
head_lateral_speed_std	kinematics	1	1	1
head_pitch_delta	kinematics	1	1	1
head_pitch_energy	kinematics	1	1	1
head_pitch_max	pose geometry	1	1	1
head_pitch_mean	pose geometry	1	1	1
head_pitch_median	pose geometry	1	1	1
head_pitch_p10	pose geometry	1	1	1
head_pitch_p90	pose geometry	1	1	1
head_pitch_periodicity	pose geometry	1	1	1
head_pitch_std	kinematics	1	1	1
head_pitch_trend	pose geometry	1	1	1
in_roi_1_body_centroid_energy	context (ROI/target)	1	1	1
in_roi_1_body_centroid_max	context (ROI/target)	1	1	1
in_roi_1_body_centroid_mean	context (ROI/target)	1	1	1
in_roi_1_body_centroid_median	context (ROI/target)	1	1	1
in_roi_1_body_centroid_p10	context (ROI/target)	1	1	1
in_roi_1_body_centroid_p90	context (ROI/target)	1	1	1
in_roi_1_body_centroid_periodicity	context (ROI/target)	1	1	1
in_roi_1_body_centroid_std	context (ROI/target)	1	1	1
in_roi_1_forepaw_centroid_energy	context (ROI/target)	1	1	1
in_roi_1_forepaw_centroid_max	context (ROI/target)	1	1	1
in_roi_1_forepaw_centroid_mean	context (ROI/target)	1	1	1
in_roi_1_forepaw_centroid_median	context (ROI/target)	1	1	1
in_roi_1_forepaw_centroid_p10	context (ROI/target)	1	1	1
in_roi_1_forepaw_centroid_p90	context (ROI/target)	1	1	1
in_roi_1_forepaw_centroid_periodicity	context (ROI/target)	1	1	1
in_roi_1_forepaw_centroid_std	context (ROI/target)	1	1	1
in_roi_1_nose_energy	context (ROI/target)	1	1	1
in_roi_1_nose_max	context (ROI/target)	1	1	1
in_roi_1_nose_mean	context (ROI/target)	1	1	1
in_roi_1_nose_median	context (ROI/target)	1	1	1
in_roi_1_nose_p10	context (ROI/target)	1	1	1
in_roi_1_nose_p90	context (ROI/target)	1	1	1
in_roi_1_nose_periodicity	context (ROI/target)	1	1	1
in_roi_1_nose_std	context (ROI/target)	1	1	1
in_roi_2_body_centroid_energy	context (ROI/target)	1	0	0
in_roi_2_body_centroid_max	context (ROI/target)	1	0	0

in_roi_2_body_centroid_mean	context (ROI/target)	1	0	0
in_roi_2_body_centroid_median	context (ROI/target)	1	0	0
in_roi_2_body_centroid_p10	context (ROI/target)	1	0	0
in_roi_2_body_centroid_p90	context (ROI/target)	1	0	0
in_roi_2_body_centroid_periodicity	context (ROI/target)	1	0	0
in_roi_2_body_centroid_std	context (ROI/target)	1	0	0
in_roi_2_forepaw_centroid_energy	context (ROI/target)	1	0	0
in_roi_2_forepaw_centroid_max	context (ROI/target)	1	0	0
in_roi_2_forepaw_centroid_mean	context (ROI/target)	1	0	0
in_roi_2_forepaw_centroid_median	context (ROI/target)	1	0	0
in_roi_2_forepaw_centroid_p10	context (ROI/target)	1	0	0
in_roi_2_forepaw_centroid_p90	context (ROI/target)	1	0	0
in_roi_2_forepaw_centroid_periodicity	context (ROI/target)	1	0	0
in_roi_2_forepaw_centroid_std	context (ROI/target)	1	0	0
in_roi_2_nose_energy	context (ROI/target)	1	0	0
in_roi_2_nose_max	context (ROI/target)	1	0	0
in_roi_2_nose_mean	context (ROI/target)	1	0	0
in_roi_2_nose_median	context (ROI/target)	1	0	0
in_roi_2_nose_p10	context (ROI/target)	1	0	0
in_roi_2_nose_p90	context (ROI/target)	1	0	0
in_roi_2_nose_periodicity	context (ROI/target)	1	0	0
in_roi_2_nose_std	context (ROI/target)	1	0	0
joint_angle_forelimb_spread_delta	kinematics	0	0	0
joint_angle_forelimb_spread_energy	kinematics	0	0	0
joint_angle_forelimb_spread_max	pose geometry	0	0	0
joint_angle_forelimb_spread_mean	pose geometry	0	0	0
joint_angle_forelimb_spread_median	pose geometry	0	0	0
joint_angle_forelimb_spread_p10	pose geometry	0	0	0
joint_angle_forelimb_spread_p90	pose geometry	0	0	0
joint_angle_forelimb_spread_periodicity	pose geometry	0	0	0
joint_angle_forelimb_spread_std	kinematics	0	0	0
joint_angle_forelimb_spread_trend	pose geometry	0	0	0
joint_angle_head_neck_angle_delta	kinematics	1	1	1
joint_angle_head_neck_angle_energy	kinematics	1	1	1
joint_angle_head_neck_angle_max	pose geometry	1	1	1
joint_angle_head_neck_angle_mean	pose geometry	1	1	1
joint_angle_head_neck_angle_median	pose geometry	1	1	1
joint_angle_head_neck_angle_p10	pose geometry	1	1	1
joint_angle_head_neck_angle_p90	pose geometry	1	1	1
joint_angle_head_neck_angle_periodicity	pose geometry	1	1	1
joint_angle_head_neck_angle_std	kinematics	1	1	1
joint_angle_head_neck_angle_trend	pose geometry	1	1	1
joint_angle_lateral_torso_delta	kinematics	1	1	0
joint_angle_lateral_torso_energy	kinematics	1	1	0

joint_angle_lateral_torso_max	pose geometry	1	1	0
joint_angle_lateral_torso_mean	pose geometry	1	1	0
joint_angle_lateral_torso_median	pose geometry	1	1	0
joint_angle_lateral_torso_p10	pose geometry	1	1	0
joint_angle_lateral_torso_p90	pose geometry	1	1	0
joint_angle_lateral_torso_periodicity	pose geometry	1	1	0
joint_angle_lateral_torso_std	kinematics	1	1	0
joint_angle_lateral_torso_trend	pose geometry	1	1	0
joint_angle_spine_flexion_delta	kinematics	1	1	1
joint_angle_spine_flexion_energy	kinematics	1	1	1
joint_angle_spine_flexion_max	pose geometry	1	1	1
joint_angle_spine_flexion_mean	pose geometry	1	1	1
joint_angle_spine_flexion_median	pose geometry	1	1	1
joint_angle_spine_flexion_p10	pose geometry	1	1	1
joint_angle_spine_flexion_p90	pose geometry	1	1	1
joint_angle_spine_flexion_periodicity	pose geometry	1	1	1
joint_angle_spine_flexion_std	kinematics	1	1	1
joint_angle_spine_flexion_trend	pose geometry	1	1	1
lateral_left_acceleration_energy	kinematics	0	0	1
lateral_left_acceleration_max	kinematics	0	0	1
lateral_left_acceleration_mean	kinematics	0	0	1
lateral_left_acceleration_median	kinematics	0	0	1
lateral_left_acceleration_p10	kinematics	0	0	1
lateral_left_acceleration_p90	kinematics	0	0	1
lateral_left_acceleration_periodicity	kinematics	0	0	1
lateral_left_acceleration_std	kinematics	0	0	1
lateral_left_forward_velocity_energy	kinematics	0	0	1
lateral_left_forward_velocity_max	kinematics	0	0	1
lateral_left_forward_velocity_mean	kinematics	0	0	1
lateral_left_forward_velocity_median	kinematics	0	0	1
lateral_left_forward_velocity_p10	kinematics	0	0	1
lateral_left_forward_velocity_p90	kinematics	0	0	1
lateral_left_forward_velocity_periodicity	kinematics	0	0	1
lateral_left_forward_velocity_std	kinematics	0	0	1
lateral_left_jerk_energy	kinematics	0	0	1
lateral_left_jerk_max	kinematics	0	0	1
lateral_left_jerk_mean	kinematics	0	0	1
lateral_left_jerk_median	kinematics	0	0	1
lateral_left_jerk_p10	kinematics	0	0	1
lateral_left_jerk_p90	kinematics	0	0	1
lateral_left_jerk_periodicity	kinematics	0	0	1
lateral_left_jerk_std	kinematics	0	0	1
lateral_left_lateral_velocity_energy	kinematics	0	0	1
lateral_left_lateral_velocity_max	kinematics	0	0	1

lateral_left_lateral_velocity_mean	kinematics	0	0	1
lateral_left_lateral_velocity_median	kinematics	0	0	1
lateral_left_lateral_velocity_p10	kinematics	0	0	1
lateral_left_lateral_velocity_p90	kinematics	0	0	1
lateral_left_lateral_velocity_periodicity	kinematics	0	0	1
lateral_left_lateral_velocity_std	kinematics	0	0	1
lateral_left_speed_energy	kinematics	0	0	1
lateral_left_speed_max	kinematics	0	0	1
lateral_left_speed_mean	kinematics	0	0	1
lateral_left_speed_median	kinematics	0	0	1
lateral_left_speed_p10	kinematics	0	0	1
lateral_left_speed_p90	kinematics	0	0	1
lateral_left_speed_periodicity	kinematics	0	0	1
lateral_left_speed_std	kinematics	0	0	1
lateral_left_velocity_x_energy	kinematics	0	0	1
lateral_left_velocity_x_max	kinematics	0	0	1
lateral_left_velocity_x_mean	kinematics	0	0	1
lateral_left_velocity_x_median	kinematics	0	0	1
lateral_left_velocity_x_p10	kinematics	0	0	1
lateral_left_velocity_x_p90	kinematics	0	0	1
lateral_left_velocity_x_periodicity	kinematics	0	0	1
lateral_left_velocity_x_std	kinematics	0	0	1
lateral_left_velocity_y_energy	kinematics	0	0	1
lateral_left_velocity_y_max	kinematics	0	0	1
lateral_left_velocity_y_mean	kinematics	0	0	1
lateral_left_velocity_y_median	kinematics	0	0	1
lateral_left_velocity_y_p10	kinematics	0	0	1
lateral_left_velocity_y_p90	kinematics	0	0	1
lateral_left_velocity_y_periodicity	kinematics	0	0	1
lateral_left_velocity_y_std	kinematics	0	0	1
lateral_right_acceleration_energy	kinematics	0	0	1
lateral_right_acceleration_max	kinematics	0	0	1
lateral_right_acceleration_mean	kinematics	0	0	1
lateral_right_acceleration_median	kinematics	0	0	1
lateral_right_acceleration_p10	kinematics	0	0	1
lateral_right_acceleration_p90	kinematics	0	0	1
lateral_right_acceleration_periodicity	kinematics	0	0	1
lateral_right_acceleration_std	kinematics	0	0	1
lateral_right_forward_velocity_energy	kinematics	0	0	1
lateral_right_forward_velocity_max	kinematics	0	0	1
lateral_right_forward_velocity_mean	kinematics	0	0	1
lateral_right_forward_velocity_median	kinematics	0	0	1
lateral_right_forward_velocity_p10	kinematics	0	0	1
lateral_right_forward_velocity_p90	kinematics	0	0	1

lateral_right_forward_velocity_periodicity	kinematics	0	0	1
lateral_right_forward_velocity_std	kinematics	0	0	1
lateral_right_jerk_energy	kinematics	0	0	1
lateral_right_jerk_max	kinematics	0	0	1
lateral_right_jerk_mean	kinematics	0	0	1
lateral_right_jerk_median	kinematics	0	0	1
lateral_right_jerk_p10	kinematics	0	0	1
lateral_right_jerk_p90	kinematics	0	0	1
lateral_right_jerk_periodicity	kinematics	0	0	1
lateral_right_jerk_std	kinematics	0	0	1
lateral_right_lateral_velocity_energy	kinematics	0	0	1
lateral_right_lateral_velocity_max	kinematics	0	0	1
lateral_right_lateral_velocity_mean	kinematics	0	0	1
lateral_right_lateral_velocity_median	kinematics	0	0	1
lateral_right_lateral_velocity_p10	kinematics	0	0	1
lateral_right_lateral_velocity_p90	kinematics	0	0	1
lateral_right_lateral_velocity_periodicity	kinematics	0	0	1
lateral_right_lateral_velocity_std	kinematics	0	0	1
lateral_right_speed_energy	kinematics	0	0	1
lateral_right_speed_max	kinematics	0	0	1
lateral_right_speed_mean	kinematics	0	0	1
lateral_right_speed_median	kinematics	0	0	1
lateral_right_speed_p10	kinematics	0	0	1
lateral_right_speed_p90	kinematics	0	0	1
lateral_right_speed_periodicity	kinematics	0	0	1
lateral_right_speed_std	kinematics	0	0	1
lateral_right_velocity_x_energy	kinematics	0	0	1
lateral_right_velocity_x_max	kinematics	0	0	1
lateral_right_velocity_x_mean	kinematics	0	0	1
lateral_right_velocity_x_median	kinematics	0	0	1
lateral_right_velocity_x_p10	kinematics	0	0	1
lateral_right_velocity_x_p90	kinematics	0	0	1
lateral_right_velocity_x_periodicity	kinematics	0	0	1
lateral_right_velocity_x_std	kinematics	0	0	1
lateral_right_velocity_y_energy	kinematics	0	0	1
lateral_right_velocity_y_max	kinematics	0	0	1
lateral_right_velocity_y_mean	kinematics	0	0	1
lateral_right_velocity_y_median	kinematics	0	0	1
lateral_right_velocity_y_p10	kinematics	0	0	1
lateral_right_velocity_y_p90	kinematics	0	0	1
lateral_right_velocity_y_periodicity	kinematics	0	0	1
lateral_right_velocity_y_std	kinematics	0	0	1
left_body_acceleration_energy	kinematics	1	0	0
left_body_acceleration_max	kinematics	1	0	0

left_body_acceleration_mean	kinematics	1	0	0
left_body_acceleration_median	kinematics	1	0	0
left_body_acceleration_p10	kinematics	1	0	0
left_body_acceleration_p90	kinematics	1	0	0
left_body_acceleration_periodicity	kinematics	1	0	0
left_body_acceleration_std	kinematics	1	0	0
left_body_forward_velocity_energy	kinematics	1	0	0
left_body_forward_velocity_max	kinematics	1	0	0
left_body_forward_velocity_mean	kinematics	1	0	0
left_body_forward_velocity_median	kinematics	1	0	0
left_body_forward_velocity_p10	kinematics	1	0	0
left_body_forward_velocity_p90	kinematics	1	0	0
left_body_forward_velocity_periodicity	kinematics	1	0	0
left_body_forward_velocity_std	kinematics	1	0	0
left_body_jerk_energy	kinematics	1	0	0
left_body_jerk_max	kinematics	1	0	0
left_body_jerk_mean	kinematics	1	0	0
left_body_jerk_median	kinematics	1	0	0
left_body_jerk_p10	kinematics	1	0	0
left_body_jerk_p90	kinematics	1	0	0
left_body_jerk_periodicity	kinematics	1	0	0
left_body_jerk_std	kinematics	1	0	0
left_body_lateral_velocity_energy	kinematics	1	0	0
left_body_lateral_velocity_max	kinematics	1	0	0
left_body_lateral_velocity_mean	kinematics	1	0	0
left_body_lateral_velocity_median	kinematics	1	0	0
left_body_lateral_velocity_p10	kinematics	1	0	0
left_body_lateral_velocity_p90	kinematics	1	0	0
left_body_lateral_velocity_periodicity	kinematics	1	0	0
left_body_lateral_velocity_std	kinematics	1	0	0
left_body_speed_energy	kinematics	1	0	0
left_body_speed_max	kinematics	1	0	0
left_body_speed_mean	kinematics	1	0	0
left_body_speed_median	kinematics	1	0	0
left_body_speed_p10	kinematics	1	0	0
left_body_speed_p90	kinematics	1	0	0
left_body_speed_periodicity	kinematics	1	0	0
left_body_speed_std	kinematics	1	0	0
left_body_velocity_x_energy	kinematics	1	0	0
left_body_velocity_x_max	kinematics	1	0	0
left_body_velocity_x_mean	kinematics	1	0	0
left_body_velocity_x_median	kinematics	1	0	0
left_body_velocity_x_p10	kinematics	1	0	0
left_body_velocity_x_p90	kinematics	1	0	0

left_body_velocity_x_periodicity	kinematics	1	0	0
left_body_velocity_x_std	kinematics	1	0	0
left_body_velocity_y_energy	kinematics	1	0	0
left_body_velocity_y_max	kinematics	1	0	0
left_body_velocity_y_mean	kinematics	1	0	0
left_body_velocity_y_median	kinematics	1	0	0
left_body_velocity_y_p10	kinematics	1	0	0
left_body_velocity_y_p90	kinematics	1	0	0
left_body_velocity_y_periodicity	kinematics	1	0	0
left_body_velocity_y_std	kinematics	1	0	0
left_ear_acceleration_energy	kinematics	1	0	0
left_ear_acceleration_max	kinematics	1	0	0
left_ear_acceleration_mean	kinematics	1	0	0
left_ear_acceleration_median	kinematics	1	0	0
left_ear_acceleration_p10	kinematics	1	0	0
left_ear_acceleration_p90	kinematics	1	0	0
left_ear_acceleration_periodicity	kinematics	1	0	0
left_ear_acceleration_std	kinematics	1	0	0
left_ear_forward_velocity_energy	kinematics	1	0	0
left_ear_forward_velocity_max	kinematics	1	0	0
left_ear_forward_velocity_mean	kinematics	1	0	0
left_ear_forward_velocity_median	kinematics	1	0	0
left_ear_forward_velocity_p10	kinematics	1	0	0
left_ear_forward_velocity_p90	kinematics	1	0	0
left_ear_forward_velocity_periodicity	kinematics	1	0	0
left_ear_forward_velocity_std	kinematics	1	0	0
left_ear_jerk_energy	kinematics	1	0	0
left_ear_jerk_max	kinematics	1	0	0
left_ear_jerk_mean	kinematics	1	0	0
left_ear_jerk_median	kinematics	1	0	0
left_ear_jerk_p10	kinematics	1	0	0
left_ear_jerk_p90	kinematics	1	0	0
left_ear_jerk_periodicity	kinematics	1	0	0
left_ear_jerk_std	kinematics	1	0	0
left_ear_lateral_velocity_energy	kinematics	1	0	0
left_ear_lateral_velocity_max	kinematics	1	0	0
left_ear_lateral_velocity_mean	kinematics	1	0	0
left_ear_lateral_velocity_median	kinematics	1	0	0
left_ear_lateral_velocity_p10	kinematics	1	0	0
left_ear_lateral_velocity_p90	kinematics	1	0	0
left_ear_lateral_velocity_periodicity	kinematics	1	0	0
left_ear_lateral_velocity_std	kinematics	1	0	0
left_ear_speed_energy	kinematics	1	0	0
left_ear_speed_max	kinematics	1	0	0

left_ear_speed_mean	kinematics	1	0	0
left_ear_speed_median	kinematics	1	0	0
left_ear_speed_p10	kinematics	1	0	0
left_ear_speed_p90	kinematics	1	0	0
left_ear_speed_periodicity	kinematics	1	0	0
left_ear_speed_std	kinematics	1	0	0
left_ear_velocity_x_energy	kinematics	1	0	0
left_ear_velocity_x_max	kinematics	1	0	0
left_ear_velocity_x_mean	kinematics	1	0	0
left_ear_velocity_x_median	kinematics	1	0	0
left_ear_velocity_x_p10	kinematics	1	0	0
left_ear_velocity_x_p90	kinematics	1	0	0
left_ear_velocity_x_periodicity	kinematics	1	0	0
left_ear_velocity_x_std	kinematics	1	0	0
left_ear_velocity_y_energy	kinematics	1	0	0
left_ear_velocity_y_max	kinematics	1	0	0
left_ear_velocity_y_mean	kinematics	1	0	0
left_ear_velocity_y_median	kinematics	1	0	0
left_ear_velocity_y_p10	kinematics	1	0	0
left_ear_velocity_y_p90	kinematics	1	0	0
left_ear_velocity_y_periodicity	kinematics	1	0	0
left_ear_velocity_y_std	kinematics	1	0	0
local_surface_change_rate_energy	kinematics	1	1	1
local_surface_change_rate_max	pose geometry	1	1	1
local_surface_change_rate_mean	pose geometry	1	1	1
local_surface_change_rate_median	pose geometry	1	1	1
local_surface_change_rate_p10	pose geometry	1	1	1
local_surface_change_rate_p90	pose geometry	1	1	1
local_surface_change_rate_periodicity	pose geometry	1	1	1
local_surface_change_rate_std	kinematics	1	1	1
local_surface_motion_energy_energy	video (motion/flow)	1	1	1
local_surface_motion_energy_max	video (motion/flow)	1	1	1
local_surface_motion_energy_mean	video (motion/flow)	1	1	1
local_surface_motion_energy_median	video (motion/flow)	1	1	1
local_surface_motion_energy_p10	video (motion/flow)	1	1	1
local_surface_motion_energy_p90	video (motion/flow)	1	1	1
local_surface_motion_energy_periodicity	video (motion/flow)	1	1	1
local_surface_motion_energy_std	video (motion/flow)	1	1	1
local_surface_motion_variance_energy	kinematics	1	1	1
local_surface_motion_variance_max	kinematics	1	1	1
local_surface_motion_variance_mean	kinematics	1	1	1
local_surface_motion_variance_median	kinematics	1	1	1
local_surface_motion_variance_p10	kinematics	1	1	1
local_surface_motion_variance_p90	kinematics	1	1	1

local_surface_motion_variance_periodicity	kinematics	1	1	1
local_surface_motion_variance_std	kinematics	1	1	1
mid_back_acceleration_energy	kinematics	0	0	0
mid_back_acceleration_max	kinematics	0	0	0
mid_back_acceleration_mean	kinematics	0	0	0
mid_back_acceleration_median	kinematics	0	0	0
mid_back_acceleration_p10	kinematics	0	0	0
mid_back_acceleration_p90	kinematics	0	0	0
mid_back_acceleration_periodicity	kinematics	0	0	0
mid_back_acceleration_std	kinematics	0	0	0
mid_back_forward_velocity_energy	kinematics	0	0	0
mid_back_forward_velocity_max	kinematics	0	0	0
mid_back_forward_velocity_mean	kinematics	0	0	0
mid_back_forward_velocity_median	kinematics	0	0	0
mid_back_forward_velocity_p10	kinematics	0	0	0
mid_back_forward_velocity_p90	kinematics	0	0	0
mid_back_forward_velocity_periodicity	kinematics	0	0	0
mid_back_forward_velocity_std	kinematics	0	0	0
mid_back_jerk_energy	kinematics	0	0	0
mid_back_jerk_max	kinematics	0	0	0
mid_back_jerk_mean	kinematics	0	0	0
mid_back_jerk_median	kinematics	0	0	0
mid_back_jerk_p10	kinematics	0	0	0
mid_back_jerk_p90	kinematics	0	0	0
mid_back_jerk_periodicity	kinematics	0	0	0
mid_back_jerk_std	kinematics	0	0	0
mid_back_lateral_velocity_energy	kinematics	0	0	0
mid_back_lateral_velocity_max	kinematics	0	0	0
mid_back_lateral_velocity_mean	kinematics	0	0	0
mid_back_lateral_velocity_median	kinematics	0	0	0
mid_back_lateral_velocity_p10	kinematics	0	0	0
mid_back_lateral_velocity_p90	kinematics	0	0	0
mid_back_lateral_velocity_periodicity	kinematics	0	0	0
mid_back_lateral_velocity_std	kinematics	0	0	0
mid_back_speed_energy	kinematics	0	0	0
mid_back_speed_max	kinematics	0	0	0
mid_back_speed_mean	kinematics	0	0	0
mid_back_speed_median	kinematics	0	0	0
mid_back_speed_p10	kinematics	0	0	0
mid_back_speed_p90	kinematics	0	0	0
mid_back_speed_periodicity	kinematics	0	0	0
mid_back_speed_std	kinematics	0	0	0
mid_back_velocity_x_energy	kinematics	0	0	0
mid_back_velocity_x_max	kinematics	0	0	0

mid_back_velocity_x_mean	kinematics	0	0	0
mid_back_velocity_x_median	kinematics	0	0	0
mid_back_velocity_x_p10	kinematics	0	0	0
mid_back_velocity_x_p90	kinematics	0	0	0
mid_back_velocity_x_periodicity	kinematics	0	0	0
mid_back_velocity_x_std	kinematics	0	0	0
mid_back_velocity_y_energy	kinematics	0	0	0
mid_back_velocity_y_max	kinematics	0	0	0
mid_back_velocity_y_mean	kinematics	0	0	0
mid_back_velocity_y_median	kinematics	0	0	0
mid_back_velocity_y_p10	kinematics	0	0	0
mid_back_velocity_y_p90	kinematics	0	0	0
mid_back_velocity_y_periodicity	kinematics	0	0	0
mid_back_velocity_y_std	kinematics	0	0	0
nose_acceleration_energy	kinematics	1	1	1
nose_acceleration_max	kinematics	1	1	1
nose_acceleration_mean	kinematics	1	1	1
nose_acceleration_median	kinematics	1	1	1
nose_acceleration_p10	kinematics	1	1	1
nose_acceleration_p90	kinematics	1	1	1
nose_acceleration_periodicity	kinematics	1	1	1
nose_acceleration_std	kinematics	1	1	1
nose_autocorr_peak_energy	kinematics	1	1	1
nose_autocorr_peak_max	pose geometry	1	1	1
nose_autocorr_peak_mean	pose geometry	1	1	1
nose_autocorr_peak_median	pose geometry	1	1	1
nose_autocorr_peak_p10	pose geometry	1	1	1
nose_autocorr_peak_p90	pose geometry	1	1	1
nose_autocorr_peak_periodicity	pose geometry	1	1	1
nose_autocorr_peak_std	kinematics	1	1	1
nose_forward_velocity_energy	kinematics	1	1	1
nose_forward_velocity_max	kinematics	1	1	1
nose_forward_velocity_mean	kinematics	1	1	1
nose_forward_velocity_median	kinematics	1	1	1
nose_forward_velocity_p10	kinematics	1	1	1
nose_forward_velocity_p90	kinematics	1	1	1
nose_forward_velocity_periodicity	kinematics	1	1	1
nose_forward_velocity_std	kinematics	1	1	1
nose_jerk_energy	kinematics	1	1	1
nose_jerk_max	kinematics	1	1	1
nose_jerk_mean	kinematics	1	1	1
nose_jerk_median	kinematics	1	1	1
nose_jerk_p10	kinematics	1	1	1
nose_jerk_p90	kinematics	1	1	1

nose_jerk_periodicity	kinematics	1	1	1
nose_jerk_std	kinematics	1	1	1
nose_lateral_velocity_energy	kinematics	1	1	1
nose_lateral_velocity_max	kinematics	1	1	1
nose_lateral_velocity_mean	kinematics	1	1	1
nose_lateral_velocity_median	kinematics	1	1	1
nose_lateral_velocity_p10	kinematics	1	1	1
nose_lateral_velocity_p90	kinematics	1	1	1
nose_lateral_velocity_periodicity	kinematics	1	1	1
nose_lateral_velocity_std	kinematics	1	1	1
nose_local_change_rate_energy	kinematics	1	1	1
nose_local_change_rate_max	pose geometry	1	1	1
nose_local_change_rate_mean	pose geometry	1	1	1
nose_local_change_rate_median	pose geometry	1	1	1
nose_local_change_rate_p10	pose geometry	1	1	1
nose_local_change_rate_p90	pose geometry	1	1	1
nose_local_change_rate_periodicity	pose geometry	1	1	1
nose_local_change_rate_std	kinematics	1	1	1
nose_local_variance_energy	kinematics	1	1	1
nose_local_variance_max	kinematics	1	1	1
nose_local_variance_mean	kinematics	1	1	1
nose_local_variance_median	kinematics	1	1	1
nose_local_variance_p10	kinematics	1	1	1
nose_local_variance_p90	kinematics	1	1	1
nose_local_variance_periodicity	kinematics	1	1	1
nose_local_variance_std	kinematics	1	1	1
nose_movement_frequency_energy	kinematics	1	1	1
nose_movement_frequency_max	kinematics	1	1	1
nose_movement_frequency_mean	kinematics	1	1	1
nose_movement_frequency_median	kinematics	1	1	1
nose_movement_frequency_p10	kinematics	1	1	1
nose_movement_frequency_p90	kinematics	1	1	1
nose_movement_frequency_periodicity	kinematics	1	1	1
nose_movement_frequency_std	kinematics	1	1	1
nose_oscillation_energy_energy	kinematics	1	1	1
nose_oscillation_energy_max	kinematics	1	1	1
nose_oscillation_energy_mean	kinematics	1	1	1
nose_oscillation_energy_median	kinematics	1	1	1
nose_oscillation_energy_p10	kinematics	1	1	1
nose_oscillation_energy_p90	kinematics	1	1	1
nose_oscillation_energy_periodicity	kinematics	1	1	1
nose_oscillation_energy_std	kinematics	1	1	1
nose_oscillation_power_energy	kinematics	1	1	1
nose_oscillation_power_max	pose geometry	1	1	1

nose_oscillation_power_mean	pose geometry	1	1	1
nose_oscillation_power_median	pose geometry	1	1	1
nose_oscillation_power_p10	pose geometry	1	1	1
nose_oscillation_power_p90	pose geometry	1	1	1
nose_oscillation_power_periodicity	pose geometry	1	1	1
nose_oscillation_power_std	kinematics	1	1	1
nose_roi_1_axial_abs_energy	context (ROI/target)	1	1	1
nose_roi_1_axial_abs_max	context (ROI/target)	1	1	1
nose_roi_1_axial_abs_mean	context (ROI/target)	1	1	1
nose_roi_1_axial_abs_median	context (ROI/target)	1	1	1
nose_roi_1_axial_abs_p10	context (ROI/target)	1	1	1
nose_roi_1_axial_abs_p90	context (ROI/target)	1	1	1
nose_roi_1_axial_abs_periodicity	context (ROI/target)	1	1	1
nose_roi_1_axial_abs_std	context (ROI/target)	1	1	1
nose_roi_1_axial_energy	context (ROI/target)	1	1	1
nose_roi_1_axial_max	context (ROI/target)	1	1	1
nose_roi_1_axial_mean	context (ROI/target)	1	1	1
nose_roi_1_axial_median	context (ROI/target)	1	1	1
nose_roi_1_axial_p10	context (ROI/target)	1	1	1
nose_roi_1_axial_p90	context (ROI/target)	1	1	1
nose_roi_1_axial_periodicity	context (ROI/target)	1	1	1
nose_roi_1_axial_std	context (ROI/target)	1	1	1
nose_roi_1_lateral_abs_energy	context (ROI/target)	1	1	1
nose_roi_1_lateral_abs_max	context (ROI/target)	1	1	1
nose_roi_1_lateral_abs_mean	context (ROI/target)	1	1	1
nose_roi_1_lateral_abs_median	context (ROI/target)	1	1	1
nose_roi_1_lateral_abs_p10	context (ROI/target)	1	1	1
nose_roi_1_lateral_abs_p90	context (ROI/target)	1	1	1
nose_roi_1_lateral_abs_periodicity	context (ROI/target)	1	1	1
nose_roi_1_lateral_abs_std	context (ROI/target)	1	1	1
nose_roi_1_lateral_energy	context (ROI/target)	1	1	1
nose_roi_1_lateral_max	context (ROI/target)	1	1	1
nose_roi_1_lateral_mean	context (ROI/target)	1	1	1
nose_roi_1_lateral_median	context (ROI/target)	1	1	1
nose_roi_1_lateral_p10	context (ROI/target)	1	1	1
nose_roi_1_lateral_p90	context (ROI/target)	1	1	1
nose_roi_1_lateral_periodicity	context (ROI/target)	1	1	1
nose_roi_1_lateral_std	context (ROI/target)	1	1	1
nose_roi_2_axial_abs_energy	context (ROI/target)	1	0	0
nose_roi_2_axial_abs_max	context (ROI/target)	1	0	0
nose_roi_2_axial_abs_mean	context (ROI/target)	1	0	0
nose_roi_2_axial_abs_median	context (ROI/target)	1	0	0
nose_roi_2_axial_abs_p10	context (ROI/target)	1	0	0
nose_roi_2_axial_abs_p90	context (ROI/target)	1	0	0

nose_roi_2_axial_abs_periodicity	context (ROI/target)	1	0	0
nose_roi_2_axial_abs_std	context (ROI/target)	1	0	0
nose_roi_2_axial_energy	context (ROI/target)	1	0	0
nose_roi_2_axial_max	context (ROI/target)	1	0	0
nose_roi_2_axial_mean	context (ROI/target)	1	0	0
nose_roi_2_axial_median	context (ROI/target)	1	0	0
nose_roi_2_axial_p10	context (ROI/target)	1	0	0
nose_roi_2_axial_p90	context (ROI/target)	1	0	0
nose_roi_2_axial_periodicity	context (ROI/target)	1	0	0
nose_roi_2_axial_std	context (ROI/target)	1	0	0
nose_roi_2_lateral_abs_energy	context (ROI/target)	1	0	0
nose_roi_2_lateral_abs_max	context (ROI/target)	1	0	0
nose_roi_2_lateral_abs_mean	context (ROI/target)	1	0	0
nose_roi_2_lateral_abs_median	context (ROI/target)	1	0	0
nose_roi_2_lateral_abs_p10	context (ROI/target)	1	0	0
nose_roi_2_lateral_abs_p90	context (ROI/target)	1	0	0
nose_roi_2_lateral_abs_periodicity	context (ROI/target)	1	0	0
nose_roi_2_lateral_abs_std	context (ROI/target)	1	0	0
nose_roi_2_lateral_energy	context (ROI/target)	1	0	0
nose_roi_2_lateral_max	context (ROI/target)	1	0	0
nose_roi_2_lateral_mean	context (ROI/target)	1	0	0
nose_roi_2_lateral_median	context (ROI/target)	1	0	0
nose_roi_2_lateral_p10	context (ROI/target)	1	0	0
nose_roi_2_lateral_p90	context (ROI/target)	1	0	0
nose_roi_2_lateral_periodicity	context (ROI/target)	1	0	0
nose_roi_2_lateral_std	context (ROI/target)	1	0	0
nose_speed_energy	kinematics	1	1	1
nose_speed_max	kinematics	1	1	1
nose_speed_mean	kinematics	1	1	1
nose_speed_median	kinematics	1	1	1
nose_speed_p10	kinematics	1	1	1
nose_speed_p90	kinematics	1	1	1
nose_speed_periodicity	kinematics	1	1	1
nose_speed_std	kinematics	1	1	1
nose_surface_change_rate_energy	kinematics	1	1	1
nose_surface_change_rate_max	pose geometry	1	1	1
nose_surface_change_rate_mean	pose geometry	1	1	1
nose_surface_change_rate_median	pose geometry	1	1	1
nose_surface_change_rate_p10	pose geometry	1	1	1
nose_surface_change_rate_p90	pose geometry	1	1	1
nose_surface_change_rate_periodicity	pose geometry	1	1	1
nose_surface_change_rate_std	kinematics	1	1	1
nose_surface_motion_energy_energy	video (motion/flow)	1	1	1
nose_surface_motion_energy_max	video (motion/flow)	1	1	1

nose_surface_motion_energy_mean	video (motion/flow)	1	1	1
nose_surface_motion_energy_median	video (motion/flow)	1	1	1
nose_surface_motion_energy_p10	video (motion/flow)	1	1	1
nose_surface_motion_energy_p90	video (motion/flow)	1	1	1
nose_surface_motion_energy_periodicity	video (motion/flow)	1	1	1
nose_surface_motion_energy_std	video (motion/flow)	1	1	1
nose_surface_motion_variance_energy	kinematics	1	1	1
nose_surface_motion_variance_max	kinematics	1	1	1
nose_surface_motion_variance_mean	kinematics	1	1	1
nose_surface_motion_variance_median	kinematics	1	1	1
nose_surface_motion_variance_p10	kinematics	1	1	1
nose_surface_motion_variance_p90	kinematics	1	1	1
nose_surface_motion_variance_periodicity	kinematics	1	1	1
nose_surface_motion_variance_std	kinematics	1	1	1
nose_to_roi_1_corner_dist_delta	context (ROI/target)	1	1	1
nose_to_roi_1_corner_dist_energy	context (ROI/target)	1	1	1
nose_to_roi_1_corner_dist_max	context (ROI/target)	1	1	1
nose_to_roi_1_corner_dist_mean	context (ROI/target)	1	1	1
nose_to_roi_1_corner_dist_median	context (ROI/target)	1	1	1
nose_to_roi_1_corner_dist_p10	context (ROI/target)	1	1	1
nose_to_roi_1_corner_dist_p90	context (ROI/target)	1	1	1
nose_to_roi_1_corner_dist_periodicity	context (ROI/target)	1	1	1
nose_to_roi_1_corner_dist_std	context (ROI/target)	1	1	1
nose_to_roi_1_corner_dist_trend	context (ROI/target)	1	1	1
nose_to_roi_1_dist_delta	context (ROI/target)	1	1	1
nose_to_roi_1_dist_energy	context (ROI/target)	1	1	1
nose_to_roi_1_dist_max	context (ROI/target)	1	1	1
nose_to_roi_1_dist_mean	context (ROI/target)	1	1	1
nose_to_roi_1_dist_median	context (ROI/target)	1	1	1
nose_to_roi_1_dist_p10	context (ROI/target)	1	1	1
nose_to_roi_1_dist_p90	context (ROI/target)	1	1	1
nose_to_roi_1_dist_periodicity	context (ROI/target)	1	1	1
nose_to_roi_1_dist_std	context (ROI/target)	1	1	1
nose_to_roi_1_dist_trend	context (ROI/target)	1	1	1
nose_to_roi_1_edge_dist_delta	context (ROI/target)	1	1	1
nose_to_roi_1_edge_dist_energy	context (ROI/target)	1	1	1
nose_to_roi_1_edge_dist_max	context (ROI/target)	1	1	1
nose_to_roi_1_edge_dist_mean	context (ROI/target)	1	1	1
nose_to_roi_1_edge_dist_median	context (ROI/target)	1	1	1
nose_to_roi_1_edge_dist_p10	context (ROI/target)	1	1	1
nose_to_roi_1_edge_dist_p90	context (ROI/target)	1	1	1
nose_to_roi_1_edge_dist_periodicity	context (ROI/target)	1	1	1
nose_to_roi_1_edge_dist_std	context (ROI/target)	1	1	1
nose_to_roi_1_edge_dist_trend	context (ROI/target)	1	1	1

nose_to_roi_1_signed_dist_delta	context (ROI/target)	1	1	1
nose_to_roi_1_signed_dist_energy	context (ROI/target)	1	1	1
nose_to_roi_1_signed_dist_max	context (ROI/target)	1	1	1
nose_to_roi_1_signed_dist_mean	context (ROI/target)	1	1	1
nose_to_roi_1_signed_dist_median	context (ROI/target)	1	1	1
nose_to_roi_1_signed_dist_p10	context (ROI/target)	1	1	1
nose_to_roi_1_signed_dist_p90	context (ROI/target)	1	1	1
nose_to_roi_1_signed_dist_periodicity	context (ROI/target)	1	1	1
nose_to_roi_1_signed_dist_std	context (ROI/target)	1	1	1
nose_to_roi_1_signed_dist_trend	context (ROI/target)	1	1	1
nose_to_roi_2_corner_dist_delta	context (ROI/target)	1	0	0
nose_to_roi_2_corner_dist_energy	context (ROI/target)	1	0	0
nose_to_roi_2_corner_dist_max	context (ROI/target)	1	0	0
nose_to_roi_2_corner_dist_mean	context (ROI/target)	1	0	0
nose_to_roi_2_corner_dist_median	context (ROI/target)	1	0	0
nose_to_roi_2_corner_dist_p10	context (ROI/target)	1	0	0
nose_to_roi_2_corner_dist_p90	context (ROI/target)	1	0	0
nose_to_roi_2_corner_dist_periodicity	context (ROI/target)	1	0	0
nose_to_roi_2_corner_dist_std	context (ROI/target)	1	0	0
nose_to_roi_2_corner_dist_trend	context (ROI/target)	1	0	0
nose_to_roi_2_dist_delta	context (ROI/target)	1	0	0
nose_to_roi_2_dist_energy	context (ROI/target)	1	0	0
nose_to_roi_2_dist_max	context (ROI/target)	1	0	0
nose_to_roi_2_dist_mean	context (ROI/target)	1	0	0
nose_to_roi_2_dist_median	context (ROI/target)	1	0	0
nose_to_roi_2_dist_p10	context (ROI/target)	1	0	0
nose_to_roi_2_dist_p90	context (ROI/target)	1	0	0
nose_to_roi_2_dist_periodicity	context (ROI/target)	1	0	0
nose_to_roi_2_dist_std	context (ROI/target)	1	0	0
nose_to_roi_2_dist_trend	context (ROI/target)	1	0	0
nose_to_roi_2_edge_dist_delta	context (ROI/target)	1	0	0
nose_to_roi_2_edge_dist_energy	context (ROI/target)	1	0	0
nose_to_roi_2_edge_dist_max	context (ROI/target)	1	0	0
nose_to_roi_2_edge_dist_mean	context (ROI/target)	1	0	0
nose_to_roi_2_edge_dist_median	context (ROI/target)	1	0	0
nose_to_roi_2_edge_dist_p10	context (ROI/target)	1	0	0
nose_to_roi_2_edge_dist_p90	context (ROI/target)	1	0	0
nose_to_roi_2_edge_dist_periodicity	context (ROI/target)	1	0	0
nose_to_roi_2_edge_dist_std	context (ROI/target)	1	0	0
nose_to_roi_2_edge_dist_trend	context (ROI/target)	1	0	0
nose_to_roi_2_signed_dist_delta	context (ROI/target)	1	0	0
nose_to_roi_2_signed_dist_energy	context (ROI/target)	1	0	0
nose_to_roi_2_signed_dist_max	context (ROI/target)	1	0	0
nose_to_roi_2_signed_dist_mean	context (ROI/target)	1	0	0

nose_to_roi_2_signed_dist_median	context (ROI/target)	1	0	0
nose_to_roi_2_signed_dist_p10	context (ROI/target)	1	0	0
nose_to_roi_2_signed_dist_p90	context (ROI/target)	1	0	0
nose_to_roi_2_signed_dist_periodicity	context (ROI/target)	1	0	0
nose_to_roi_2_signed_dist_std	context (ROI/target)	1	0	0
nose_to_roi_2_signed_dist_trend	context (ROI/target)	1	0	0
nose_to_target_dist_delta	context (ROI/target)	1	1	1
nose_to_target_dist_energy	context (ROI/target)	1	1	1
nose_to_target_dist_max	context (ROI/target)	1	1	1
nose_to_target_dist_mean	context (ROI/target)	1	1	1
nose_to_target_dist_median	context (ROI/target)	1	1	1
nose_to_target_dist_p10	context (ROI/target)	1	1	1
nose_to_target_dist_p90	context (ROI/target)	1	1	1
nose_to_target_dist_periodicity	context (ROI/target)	1	1	1
nose_to_target_dist_std	context (ROI/target)	1	1	1
nose_to_target_dist_trend	context (ROI/target)	1	1	1
nose_velocity_energy	kinematics	1	1	1
nose_velocity_max	kinematics	1	1	1
nose_velocity_mean	kinematics	1	1	1
nose_velocity_median	kinematics	1	1	1
nose_velocity_p10	kinematics	1	1	1
nose_velocity_p90	kinematics	1	1	1
nose_velocity_periodicity	kinematics	1	1	1
nose_velocity_std	kinematics	1	1	1
nose_velocity_x_energy	kinematics	1	1	1
nose_velocity_x_max	kinematics	1	1	1
nose_velocity_x_mean	kinematics	1	1	1
nose_velocity_x_median	kinematics	1	1	1
nose_velocity_x_p10	kinematics	1	1	1
nose_velocity_x_p90	kinematics	1	1	1
nose_velocity_x_periodicity	kinematics	1	1	1
nose_velocity_x_std	kinematics	1	1	1
nose_velocity_y_energy	kinematics	1	1	1
nose_velocity_y_max	kinematics	1	1	1
nose_velocity_y_mean	kinematics	1	1	1
nose_velocity_y_median	kinematics	1	1	1
nose_velocity_y_p10	kinematics	1	1	1
nose_velocity_y_p90	kinematics	1	1	1
nose_velocity_y_periodicity	kinematics	1	1	1
nose_velocity_y_std	kinematics	1	1	1
nose_vertical_velocity_energy	kinematics	1	1	1
nose_vertical_velocity_max	kinematics	1	1	1
nose_vertical_velocity_mean	kinematics	1	1	1
nose_vertical_velocity_median	kinematics	1	1	1

nose_vertical_velocity_p10	kinematics	1	1	1
nose_vertical_velocity_p90	kinematics	1	1	1
nose_vertical_velocity_periodicity	kinematics	1	1	1
nose_vertical_velocity_std	kinematics	1	1	1
oscillation_energy_energy	kinematics	1	1	1
oscillation_energy_max	kinematics	1	1	1
oscillation_energy_mean	kinematics	1	1	1
oscillation_energy_median	kinematics	1	1	1
oscillation_energy_p10	kinematics	1	1	1
oscillation_energy_p90	kinematics	1	1	1
oscillation_energy_periodicity	kinematics	1	1	1
oscillation_energy_std	kinematics	1	1	1
prediction_prob	pose geometry	1	1	1
prediction_variance	kinematics	1	1	1
r3d_000	video (R3D appearance	1	1	1
r3d_001	video (R3D appearance	1	1	1
r3d_002	video (R3D appearance	1	1	1
r3d_003	video (R3D appearance	1	1	1
r3d_004	video (R3D appearance	1	1	1
r3d_005	video (R3D appearance	1	1	1
r3d_006	video (R3D appearance	1	1	1
r3d_007	video (R3D appearance	1	1	1
r3d_008	video (R3D appearance	1	1	1
r3d_009	video (R3D appearance	1	1	1
r3d_010	video (R3D appearance	1	1	1
r3d_011	video (R3D appearance	1	1	1
r3d_012	video (R3D appearance	1	1	1
r3d_013	video (R3D appearance	1	1	1
r3d_014	video (R3D appearance	1	1	1
r3d_015	video (R3D appearance	1	1	1
r3d_016	video (R3D appearance	1	1	1
r3d_017	video (R3D appearance	1	1	1
r3d_018	video (R3D appearance	1	1	1
r3d_019	video (R3D appearance	1	1	1
r3d_020	video (R3D appearance	1	1	1
r3d_021	video (R3D appearance	1	1	1
r3d_022	video (R3D appearance	1	1	1
r3d_023	video (R3D appearance	1	1	1
r3d_024	video (R3D appearance	1	1	1
r3d_025	video (R3D appearance	1	1	1
r3d_026	video (R3D appearance	1	1	1
r3d_027	video (R3D appearance	1	1	1
r3d_028	video (R3D appearance	1	1	1
r3d_029	video (R3D appearance	1	1	1

r3d_030	video (R3D appearance	1	1	1
r3d_031	video (R3D appearance	1	1	1
r3d_032	video (R3D appearance	1	1	1
r3d_033	video (R3D appearance	1	1	1
r3d_034	video (R3D appearance	1	1	1
r3d_035	video (R3D appearance	1	1	1
r3d_036	video (R3D appearance	1	1	1
r3d_037	video (R3D appearance	1	1	1
r3d_038	video (R3D appearance	1	1	1
r3d_039	video (R3D appearance	1	1	1
r3d_040	video (R3D appearance	1	1	1
r3d_041	video (R3D appearance	1	1	1
r3d_042	video (R3D appearance	1	1	1
r3d_043	video (R3D appearance	1	1	1
r3d_044	video (R3D appearance	1	1	1
r3d_045	video (R3D appearance	1	1	1
r3d_046	video (R3D appearance	1	1	1
r3d_047	video (R3D appearance	1	1	1
r3d_048	video (R3D appearance	1	1	1
r3d_049	video (R3D appearance	1	1	1
r3d_050	video (R3D appearance	1	1	1
r3d_051	video (R3D appearance	1	1	1
r3d_052	video (R3D appearance	1	1	1
r3d_053	video (R3D appearance	1	1	1
r3d_054	video (R3D appearance	1	1	1
r3d_055	video (R3D appearance	1	1	1
r3d_056	video (R3D appearance	1	1	1
r3d_057	video (R3D appearance	1	1	1
r3d_058	video (R3D appearance	1	1	1
r3d_059	video (R3D appearance	1	1	1
r3d_060	video (R3D appearance	1	1	1
r3d_061	video (R3D appearance	1	1	1
r3d_062	video (R3D appearance	1	1	1
r3d_063	video (R3D appearance	1	1	1
r3d_064	video (R3D appearance	1	1	1
r3d_065	video (R3D appearance	1	1	1
r3d_066	video (R3D appearance	1	1	1
r3d_067	video (R3D appearance	1	1	1
r3d_068	video (R3D appearance	1	1	1
r3d_069	video (R3D appearance	1	1	1
r3d_070	video (R3D appearance	1	1	1
r3d_071	video (R3D appearance	1	1	1
r3d_072	video (R3D appearance	1	1	1
r3d_073	video (R3D appearance	1	1	1

r3d_074	video (R3D appearance	1	1	1
r3d_075	video (R3D appearance	1	1	1
r3d_076	video (R3D appearance	1	1	1
r3d_077	video (R3D appearance	1	1	1
r3d_078	video (R3D appearance	1	1	1
r3d_079	video (R3D appearance	1	1	1
r3d_080	video (R3D appearance	1	1	1
r3d_081	video (R3D appearance	1	1	1
r3d_082	video (R3D appearance	1	1	1
r3d_083	video (R3D appearance	1	1	1
r3d_084	video (R3D appearance	1	1	1
r3d_085	video (R3D appearance	1	1	1
r3d_086	video (R3D appearance	1	1	1
r3d_087	video (R3D appearance	1	1	1
r3d_088	video (R3D appearance	1	1	1
r3d_089	video (R3D appearance	1	1	1
r3d_090	video (R3D appearance	1	1	1
r3d_091	video (R3D appearance	1	1	1
r3d_092	video (R3D appearance	1	1	1
r3d_093	video (R3D appearance	1	1	1
r3d_094	video (R3D appearance	1	1	1
r3d_095	video (R3D appearance	1	1	1
r3d_096	video (R3D appearance	1	1	1
r3d_097	video (R3D appearance	1	1	1
r3d_098	video (R3D appearance	1	1	1
r3d_099	video (R3D appearance	1	1	1
r3d_100	video (R3D appearance	1	1	1
r3d_101	video (R3D appearance	1	1	1
r3d_102	video (R3D appearance	1	1	1
r3d_103	video (R3D appearance	1	1	1
r3d_104	video (R3D appearance	1	1	1
r3d_105	video (R3D appearance	1	1	1
r3d_106	video (R3D appearance	1	1	1
r3d_107	video (R3D appearance	1	1	1
r3d_108	video (R3D appearance	1	1	1
r3d_109	video (R3D appearance	1	1	1
r3d_110	video (R3D appearance	1	1	1
r3d_111	video (R3D appearance	1	1	1
r3d_112	video (R3D appearance	1	1	1
r3d_113	video (R3D appearance	1	1	1
r3d_114	video (R3D appearance	1	1	1
r3d_115	video (R3D appearance	1	1	1
r3d_116	video (R3D appearance	1	1	1
r3d_117	video (R3D appearance	1	1	1

r3d_118	video (R3D appearance	1	1	1
r3d_119	video (R3D appearance	1	1	1
r3d_120	video (R3D appearance	1	1	1
r3d_121	video (R3D appearance	1	1	1
r3d_122	video (R3D appearance	1	1	1
r3d_123	video (R3D appearance	1	1	1
r3d_124	video (R3D appearance	1	1	1
r3d_125	video (R3D appearance	1	1	1
r3d_126	video (R3D appearance	1	1	1
r3d_127	video (R3D appearance	1	1	1
r3d_128	video (R3D appearance	1	1	1
r3d_129	video (R3D appearance	1	1	1
r3d_130	video (R3D appearance	1	1	1
r3d_131	video (R3D appearance	1	1	1
r3d_132	video (R3D appearance	1	1	1
r3d_133	video (R3D appearance	1	1	1
r3d_134	video (R3D appearance	1	1	1
r3d_135	video (R3D appearance	1	1	1
r3d_136	video (R3D appearance	1	1	1
r3d_137	video (R3D appearance	1	1	1
r3d_138	video (R3D appearance	1	1	1
r3d_139	video (R3D appearance	1	1	1
r3d_140	video (R3D appearance	1	1	1
r3d_141	video (R3D appearance	1	1	1
r3d_142	video (R3D appearance	1	1	1
r3d_143	video (R3D appearance	1	1	1
r3d_144	video (R3D appearance	1	1	1
r3d_145	video (R3D appearance	1	1	1
r3d_146	video (R3D appearance	1	1	1
r3d_147	video (R3D appearance	1	1	1
r3d_148	video (R3D appearance	1	1	1
r3d_149	video (R3D appearance	1	1	1
r3d_150	video (R3D appearance	1	1	1
r3d_151	video (R3D appearance	1	1	1
r3d_152	video (R3D appearance	1	1	1
r3d_153	video (R3D appearance	1	1	1
r3d_154	video (R3D appearance	1	1	1
r3d_155	video (R3D appearance	1	1	1
r3d_156	video (R3D appearance	1	1	1
r3d_157	video (R3D appearance	1	1	1
r3d_158	video (R3D appearance	1	1	1
r3d_159	video (R3D appearance	1	1	1
r3d_160	video (R3D appearance	1	1	1
r3d_161	video (R3D appearance	1	1	1

r3d_162	video (R3D appearance	1	1	1
r3d_163	video (R3D appearance	1	1	1
r3d_164	video (R3D appearance	1	1	1
r3d_165	video (R3D appearance	1	1	1
r3d_166	video (R3D appearance	1	1	1
r3d_167	video (R3D appearance	1	1	1
r3d_168	video (R3D appearance	1	1	1
r3d_169	video (R3D appearance	1	1	1
r3d_170	video (R3D appearance	1	1	1
r3d_171	video (R3D appearance	1	1	1
r3d_172	video (R3D appearance	1	1	1
r3d_173	video (R3D appearance	1	1	1
r3d_174	video (R3D appearance	1	1	1
r3d_175	video (R3D appearance	1	1	1
r3d_176	video (R3D appearance	1	1	1
r3d_177	video (R3D appearance	1	1	1
r3d_178	video (R3D appearance	1	1	1
r3d_179	video (R3D appearance	1	1	1
r3d_180	video (R3D appearance	1	1	1
r3d_181	video (R3D appearance	1	1	1
r3d_182	video (R3D appearance	1	1	1
r3d_183	video (R3D appearance	1	1	1
r3d_184	video (R3D appearance	1	1	1
r3d_185	video (R3D appearance	1	1	1
r3d_186	video (R3D appearance	1	1	1
r3d_187	video (R3D appearance	1	1	1
r3d_188	video (R3D appearance	1	1	1
r3d_189	video (R3D appearance	1	1	1
r3d_190	video (R3D appearance	1	1	1
r3d_191	video (R3D appearance	1	1	1
r3d_192	video (R3D appearance	1	1	1
r3d_193	video (R3D appearance	1	1	1
r3d_194	video (R3D appearance	1	1	1
r3d_195	video (R3D appearance	1	1	1
r3d_196	video (R3D appearance	1	1	1
r3d_197	video (R3D appearance	1	1	1
r3d_198	video (R3D appearance	1	1	1
r3d_199	video (R3D appearance	1	1	1
r3d_200	video (R3D appearance	1	1	1
r3d_201	video (R3D appearance	1	1	1
r3d_202	video (R3D appearance	1	1	1
r3d_203	video (R3D appearance	1	1	1
r3d_204	video (R3D appearance	1	1	1
r3d_205	video (R3D appearance	1	1	1

r3d_206	video (R3D appearance	1	1	1
r3d_207	video (R3D appearance	1	1	1
r3d_208	video (R3D appearance	1	1	1
r3d_209	video (R3D appearance	1	1	1
r3d_210	video (R3D appearance	1	1	1
r3d_211	video (R3D appearance	1	1	1
r3d_212	video (R3D appearance	1	1	1
r3d_213	video (R3D appearance	1	1	1
r3d_214	video (R3D appearance	1	1	1
r3d_215	video (R3D appearance	1	1	1
r3d_216	video (R3D appearance	1	1	1
r3d_217	video (R3D appearance	1	1	1
r3d_218	video (R3D appearance	1	1	1
r3d_219	video (R3D appearance	1	1	1
r3d_220	video (R3D appearance	1	1	1
r3d_221	video (R3D appearance	1	1	1
r3d_222	video (R3D appearance	1	1	1
r3d_223	video (R3D appearance	1	1	1
r3d_224	video (R3D appearance	1	1	1
r3d_225	video (R3D appearance	1	1	1
r3d_226	video (R3D appearance	1	1	1
r3d_227	video (R3D appearance	1	1	1
r3d_228	video (R3D appearance	1	1	1
r3d_229	video (R3D appearance	1	1	1
r3d_230	video (R3D appearance	1	1	1
r3d_231	video (R3D appearance	1	1	1
r3d_232	video (R3D appearance	1	1	1
r3d_233	video (R3D appearance	1	1	1
r3d_234	video (R3D appearance	1	1	1
r3d_235	video (R3D appearance	1	1	1
r3d_236	video (R3D appearance	1	1	1
r3d_237	video (R3D appearance	1	1	1
r3d_238	video (R3D appearance	1	1	1
r3d_239	video (R3D appearance	1	1	1
r3d_240	video (R3D appearance	1	1	1
r3d_241	video (R3D appearance	1	1	1
r3d_242	video (R3D appearance	1	1	1
r3d_243	video (R3D appearance	1	1	1
r3d_244	video (R3D appearance	1	1	1
r3d_245	video (R3D appearance	1	1	1
r3d_246	video (R3D appearance	1	1	1
r3d_247	video (R3D appearance	1	1	1
r3d_248	video (R3D appearance	1	1	1
r3d_249	video (R3D appearance	1	1	1

r3d_250	video (R3D appearance	1	1	1
r3d_251	video (R3D appearance	1	1	1
r3d_252	video (R3D appearance	1	1	1
r3d_253	video (R3D appearance	1	1	1
r3d_254	video (R3D appearance	1	1	1
r3d_255	video (R3D appearance	1	1	1
r3d_256	video (R3D appearance	1	1	1
r3d_257	video (R3D appearance	1	1	1
r3d_258	video (R3D appearance	1	1	1
r3d_259	video (R3D appearance	1	1	1
r3d_260	video (R3D appearance	1	1	1
r3d_261	video (R3D appearance	1	1	1
r3d_262	video (R3D appearance	1	1	1
r3d_263	video (R3D appearance	1	1	1
r3d_264	video (R3D appearance	1	1	1
r3d_265	video (R3D appearance	1	1	1
r3d_266	video (R3D appearance	1	1	1
r3d_267	video (R3D appearance	1	1	1
r3d_268	video (R3D appearance	1	1	1
r3d_269	video (R3D appearance	1	1	1
r3d_270	video (R3D appearance	1	1	1
r3d_271	video (R3D appearance	1	1	1
r3d_272	video (R3D appearance	1	1	1
r3d_273	video (R3D appearance	1	1	1
r3d_274	video (R3D appearance	1	1	1
r3d_275	video (R3D appearance	1	1	1
r3d_276	video (R3D appearance	1	1	1
r3d_277	video (R3D appearance	1	1	1
r3d_278	video (R3D appearance	1	1	1
r3d_279	video (R3D appearance	1	1	1
r3d_280	video (R3D appearance	1	1	1
r3d_281	video (R3D appearance	1	1	1
r3d_282	video (R3D appearance	1	1	1
r3d_283	video (R3D appearance	1	1	1
r3d_284	video (R3D appearance	1	1	1
r3d_285	video (R3D appearance	1	1	1
r3d_286	video (R3D appearance	1	1	1
r3d_287	video (R3D appearance	1	1	1
r3d_288	video (R3D appearance	1	1	1
r3d_289	video (R3D appearance	1	1	1
r3d_290	video (R3D appearance	1	1	1
r3d_291	video (R3D appearance	1	1	1
r3d_292	video (R3D appearance	1	1	1
r3d_293	video (R3D appearance	1	1	1

r3d_294	video (R3D appearance	1	1	1
r3d_295	video (R3D appearance	1	1	1
r3d_296	video (R3D appearance	1	1	1
r3d_297	video (R3D appearance	1	1	1
r3d_298	video (R3D appearance	1	1	1
r3d_299	video (R3D appearance	1	1	1
r3d_300	video (R3D appearance	1	1	1
r3d_301	video (R3D appearance	1	1	1
r3d_302	video (R3D appearance	1	1	1
r3d_303	video (R3D appearance	1	1	1
r3d_304	video (R3D appearance	1	1	1
r3d_305	video (R3D appearance	1	1	1
r3d_306	video (R3D appearance	1	1	1
r3d_307	video (R3D appearance	1	1	1
r3d_308	video (R3D appearance	1	1	1
r3d_309	video (R3D appearance	1	1	1
r3d_310	video (R3D appearance	1	1	1
r3d_311	video (R3D appearance	1	1	1
r3d_312	video (R3D appearance	1	1	1
r3d_313	video (R3D appearance	1	1	1
r3d_314	video (R3D appearance	1	1	1
r3d_315	video (R3D appearance	1	1	1
r3d_316	video (R3D appearance	1	1	1
r3d_317	video (R3D appearance	1	1	1
r3d_318	video (R3D appearance	1	1	1
r3d_319	video (R3D appearance	1	1	1
r3d_320	video (R3D appearance	1	1	1
r3d_321	video (R3D appearance	1	1	1
r3d_322	video (R3D appearance	1	1	1
r3d_323	video (R3D appearance	1	1	1
r3d_324	video (R3D appearance	1	1	1
r3d_325	video (R3D appearance	1	1	1
r3d_326	video (R3D appearance	1	1	1
r3d_327	video (R3D appearance	1	1	1
r3d_328	video (R3D appearance	1	1	1
r3d_329	video (R3D appearance	1	1	1
r3d_330	video (R3D appearance	1	1	1
r3d_331	video (R3D appearance	1	1	1
r3d_332	video (R3D appearance	1	1	1
r3d_333	video (R3D appearance	1	1	1
r3d_334	video (R3D appearance	1	1	1
r3d_335	video (R3D appearance	1	1	1
r3d_336	video (R3D appearance	1	1	1
r3d_337	video (R3D appearance	1	1	1

r3d_338	video (R3D appearance	1	1	1
r3d_339	video (R3D appearance	1	1	1
r3d_340	video (R3D appearance	1	1	1
r3d_341	video (R3D appearance	1	1	1
r3d_342	video (R3D appearance	1	1	1
r3d_343	video (R3D appearance	1	1	1
r3d_344	video (R3D appearance	1	1	1
r3d_345	video (R3D appearance	1	1	1
r3d_346	video (R3D appearance	1	1	1
r3d_347	video (R3D appearance	1	1	1
r3d_348	video (R3D appearance	1	1	1
r3d_349	video (R3D appearance	1	1	1
r3d_350	video (R3D appearance	1	1	1
r3d_351	video (R3D appearance	1	1	1
r3d_352	video (R3D appearance	1	1	1
r3d_353	video (R3D appearance	1	1	1
r3d_354	video (R3D appearance	1	1	1
r3d_355	video (R3D appearance	1	1	1
r3d_356	video (R3D appearance	1	1	1
r3d_357	video (R3D appearance	1	1	1
r3d_358	video (R3D appearance	1	1	1
r3d_359	video (R3D appearance	1	1	1
r3d_360	video (R3D appearance	1	1	1
r3d_361	video (R3D appearance	1	1	1
r3d_362	video (R3D appearance	1	1	1
r3d_363	video (R3D appearance	1	1	1
r3d_364	video (R3D appearance	1	1	1
r3d_365	video (R3D appearance	1	1	1
r3d_366	video (R3D appearance	1	1	1
r3d_367	video (R3D appearance	1	1	1
r3d_368	video (R3D appearance	1	1	1
r3d_369	video (R3D appearance	1	1	1
r3d_370	video (R3D appearance	1	1	1
r3d_371	video (R3D appearance	1	1	1
r3d_372	video (R3D appearance	1	1	1
r3d_373	video (R3D appearance	1	1	1
r3d_374	video (R3D appearance	1	1	1
r3d_375	video (R3D appearance	1	1	1
r3d_376	video (R3D appearance	1	1	1
r3d_377	video (R3D appearance	1	1	1
r3d_378	video (R3D appearance	1	1	1
r3d_379	video (R3D appearance	1	1	1
r3d_380	video (R3D appearance	1	1	1
r3d_381	video (R3D appearance	1	1	1

r3d_382	video (R3D appearance	1	1	1
r3d_383	video (R3D appearance	1	1	1
r3d_384	video (R3D appearance	1	1	1
r3d_385	video (R3D appearance	1	1	1
r3d_386	video (R3D appearance	1	1	1
r3d_387	video (R3D appearance	1	1	1
r3d_388	video (R3D appearance	1	1	1
r3d_389	video (R3D appearance	1	1	1
r3d_390	video (R3D appearance	1	1	1
r3d_391	video (R3D appearance	1	1	1
r3d_392	video (R3D appearance	1	1	1
r3d_393	video (R3D appearance	1	1	1
r3d_394	video (R3D appearance	1	1	1
r3d_395	video (R3D appearance	1	1	1
r3d_396	video (R3D appearance	1	1	1
r3d_397	video (R3D appearance	1	1	1
r3d_398	video (R3D appearance	1	1	1
r3d_399	video (R3D appearance	1	1	1
r3d_400	video (R3D appearance	1	1	1
r3d_401	video (R3D appearance	1	1	1
r3d_402	video (R3D appearance	1	1	1
r3d_403	video (R3D appearance	1	1	1
r3d_404	video (R3D appearance	1	1	1
r3d_405	video (R3D appearance	1	1	1
r3d_406	video (R3D appearance	1	1	1
r3d_407	video (R3D appearance	1	1	1
r3d_408	video (R3D appearance	1	1	1
r3d_409	video (R3D appearance	1	1	1
r3d_410	video (R3D appearance	1	1	1
r3d_411	video (R3D appearance	1	1	1
r3d_412	video (R3D appearance	1	1	1
r3d_413	video (R3D appearance	1	1	1
r3d_414	video (R3D appearance	1	1	1
r3d_415	video (R3D appearance	1	1	1
r3d_416	video (R3D appearance	1	1	1
r3d_417	video (R3D appearance	1	1	1
r3d_418	video (R3D appearance	1	1	1
r3d_419	video (R3D appearance	1	1	1
r3d_420	video (R3D appearance	1	1	1
r3d_421	video (R3D appearance	1	1	1
r3d_422	video (R3D appearance	1	1	1
r3d_423	video (R3D appearance	1	1	1
r3d_424	video (R3D appearance	1	1	1
r3d_425	video (R3D appearance	1	1	1

r3d_426	video (R3D appearance	1	1	1
r3d_427	video (R3D appearance	1	1	1
r3d_428	video (R3D appearance	1	1	1
r3d_429	video (R3D appearance	1	1	1
r3d_430	video (R3D appearance	1	1	1
r3d_431	video (R3D appearance	1	1	1
r3d_432	video (R3D appearance	1	1	1
r3d_433	video (R3D appearance	1	1	1
r3d_434	video (R3D appearance	1	1	1
r3d_435	video (R3D appearance	1	1	1
r3d_436	video (R3D appearance	1	1	1
r3d_437	video (R3D appearance	1	1	1
r3d_438	video (R3D appearance	1	1	1
r3d_439	video (R3D appearance	1	1	1
r3d_440	video (R3D appearance	1	1	1
r3d_441	video (R3D appearance	1	1	1
r3d_442	video (R3D appearance	1	1	1
r3d_443	video (R3D appearance	1	1	1
r3d_444	video (R3D appearance	1	1	1
r3d_445	video (R3D appearance	1	1	1
r3d_446	video (R3D appearance	1	1	1
r3d_447	video (R3D appearance	1	1	1
r3d_448	video (R3D appearance	1	1	1
r3d_449	video (R3D appearance	1	1	1
r3d_450	video (R3D appearance	1	1	1
r3d_451	video (R3D appearance	1	1	1
r3d_452	video (R3D appearance	1	1	1
r3d_453	video (R3D appearance	1	1	1
r3d_454	video (R3D appearance	1	1	1
r3d_455	video (R3D appearance	1	1	1
r3d_456	video (R3D appearance	1	1	1
r3d_457	video (R3D appearance	1	1	1
r3d_458	video (R3D appearance	1	1	1
r3d_459	video (R3D appearance	1	1	1
r3d_460	video (R3D appearance	1	1	1
r3d_461	video (R3D appearance	1	1	1
r3d_462	video (R3D appearance	1	1	1
r3d_463	video (R3D appearance	1	1	1
r3d_464	video (R3D appearance	1	1	1
r3d_465	video (R3D appearance	1	1	1
r3d_466	video (R3D appearance	1	1	1
r3d_467	video (R3D appearance	1	1	1
r3d_468	video (R3D appearance	1	1	1
r3d_469	video (R3D appearance	1	1	1

r3d_470	video (R3D appearance	1	1	1
r3d_471	video (R3D appearance	1	1	1
r3d_472	video (R3D appearance	1	1	1
r3d_473	video (R3D appearance	1	1	1
r3d_474	video (R3D appearance	1	1	1
r3d_475	video (R3D appearance	1	1	1
r3d_476	video (R3D appearance	1	1	1
r3d_477	video (R3D appearance	1	1	1
r3d_478	video (R3D appearance	1	1	1
r3d_479	video (R3D appearance	1	1	1
r3d_480	video (R3D appearance	1	1	1
r3d_481	video (R3D appearance	1	1	1
r3d_482	video (R3D appearance	1	1	1
r3d_483	video (R3D appearance	1	1	1
r3d_484	video (R3D appearance	1	1	1
r3d_485	video (R3D appearance	1	1	1
r3d_486	video (R3D appearance	1	1	1
r3d_487	video (R3D appearance	1	1	1
r3d_488	video (R3D appearance	1	1	1
r3d_489	video (R3D appearance	1	1	1
r3d_490	video (R3D appearance	1	1	1
r3d_491	video (R3D appearance	1	1	1
r3d_492	video (R3D appearance	1	1	1
r3d_493	video (R3D appearance	1	1	1
r3d_494	video (R3D appearance	1	1	1
r3d_495	video (R3D appearance	1	1	1
r3d_496	video (R3D appearance	1	1	1
r3d_497	video (R3D appearance	1	1	1
r3d_498	video (R3D appearance	1	1	1
r3d_499	video (R3D appearance	1	1	1
r3d_500	video (R3D appearance	1	1	1
r3d_501	video (R3D appearance	1	1	1
r3d_502	video (R3D appearance	1	1	1
r3d_503	video (R3D appearance	1	1	1
r3d_504	video (R3D appearance	1	1	1
r3d_505	video (R3D appearance	1	1	1
r3d_506	video (R3D appearance	1	1	1
r3d_507	video (R3D appearance	1	1	1
r3d_508	video (R3D appearance	1	1	1
r3d_509	video (R3D appearance	1	1	1
r3d_510	video (R3D appearance	1	1	1
r3d_511	video (R3D appearance	1	1	1
right_body_acceleration_energy	kinematics	1	0	0
right_body_acceleration_max	kinematics	1	0	0

right_body_acceleration_mean	kinematics	1	0	0
right_body_acceleration_median	kinematics	1	0	0
right_body_acceleration_p10	kinematics	1	0	0
right_body_acceleration_p90	kinematics	1	0	0
right_body_acceleration_periodicity	kinematics	1	0	0
right_body_acceleration_std	kinematics	1	0	0
right_body_forward_velocity_energy	kinematics	1	0	0
right_body_forward_velocity_max	kinematics	1	0	0
right_body_forward_velocity_mean	kinematics	1	0	0
right_body_forward_velocity_median	kinematics	1	0	0
right_body_forward_velocity_p10	kinematics	1	0	0
right_body_forward_velocity_p90	kinematics	1	0	0
right_body_forward_velocity_periodicity	kinematics	1	0	0
right_body_forward_velocity_std	kinematics	1	0	0
right_body_jerk_energy	kinematics	1	0	0
right_body_jerk_max	kinematics	1	0	0
right_body_jerk_mean	kinematics	1	0	0
right_body_jerk_median	kinematics	1	0	0
right_body_jerk_p10	kinematics	1	0	0
right_body_jerk_p90	kinematics	1	0	0
right_body_jerk_periodicity	kinematics	1	0	0
right_body_jerk_std	kinematics	1	0	0
right_body_lateral_velocity_energy	kinematics	1	0	0
right_body_lateral_velocity_max	kinematics	1	0	0
right_body_lateral_velocity_mean	kinematics	1	0	0
right_body_lateral_velocity_median	kinematics	1	0	0
right_body_lateral_velocity_p10	kinematics	1	0	0
right_body_lateral_velocity_p90	kinematics	1	0	0
right_body_lateral_velocity_periodicity	kinematics	1	0	0
right_body_lateral_velocity_std	kinematics	1	0	0
right_body_speed_energy	kinematics	1	0	0
right_body_speed_max	kinematics	1	0	0
right_body_speed_mean	kinematics	1	0	0
right_body_speed_median	kinematics	1	0	0
right_body_speed_p10	kinematics	1	0	0
right_body_speed_p90	kinematics	1	0	0
right_body_speed_periodicity	kinematics	1	0	0
right_body_speed_std	kinematics	1	0	0
right_body_velocity_x_energy	kinematics	1	0	0
right_body_velocity_x_max	kinematics	1	0	0
right_body_velocity_x_mean	kinematics	1	0	0
right_body_velocity_x_median	kinematics	1	0	0
right_body_velocity_x_p10	kinematics	1	0	0
right_body_velocity_x_p90	kinematics	1	0	0

right_body_velocity_x_periodicity	kinematics	1	0	0
right_body_velocity_x_std	kinematics	1	0	0
right_body_velocity_y_energy	kinematics	1	0	0
right_body_velocity_y_max	kinematics	1	0	0
right_body_velocity_y_mean	kinematics	1	0	0
right_body_velocity_y_median	kinematics	1	0	0
right_body_velocity_y_p10	kinematics	1	0	0
right_body_velocity_y_p90	kinematics	1	0	0
right_body_velocity_y_periodicity	kinematics	1	0	0
right_body_velocity_y_std	kinematics	1	0	0
right_ear_acceleration_energy	kinematics	1	0	0
right_ear_acceleration_max	kinematics	1	0	0
right_ear_acceleration_mean	kinematics	1	0	0
right_ear_acceleration_median	kinematics	1	0	0
right_ear_acceleration_p10	kinematics	1	0	0
right_ear_acceleration_p90	kinematics	1	0	0
right_ear_acceleration_periodicity	kinematics	1	0	0
right_ear_acceleration_std	kinematics	1	0	0
right_ear_forward_velocity_energy	kinematics	1	0	0
right_ear_forward_velocity_max	kinematics	1	0	0
right_ear_forward_velocity_mean	kinematics	1	0	0
right_ear_forward_velocity_median	kinematics	1	0	0
right_ear_forward_velocity_p10	kinematics	1	0	0
right_ear_forward_velocity_p90	kinematics	1	0	0
right_ear_forward_velocity_periodicity	kinematics	1	0	0
right_ear_forward_velocity_std	kinematics	1	0	0
right_ear_jerk_energy	kinematics	1	0	0
right_ear_jerk_max	kinematics	1	0	0
right_ear_jerk_mean	kinematics	1	0	0
right_ear_jerk_median	kinematics	1	0	0
right_ear_jerk_p10	kinematics	1	0	0
right_ear_jerk_p90	kinematics	1	0	0
right_ear_jerk_periodicity	kinematics	1	0	0
right_ear_jerk_std	kinematics	1	0	0
right_ear_lateral_velocity_energy	kinematics	1	0	0
right_ear_lateral_velocity_max	kinematics	1	0	0
right_ear_lateral_velocity_mean	kinematics	1	0	0
right_ear_lateral_velocity_median	kinematics	1	0	0
right_ear_lateral_velocity_p10	kinematics	1	0	0
right_ear_lateral_velocity_p90	kinematics	1	0	0
right_ear_lateral_velocity_periodicity	kinematics	1	0	0
right_ear_lateral_velocity_std	kinematics	1	0	0
right_ear_speed_energy	kinematics	1	0	0
right_ear_speed_max	kinematics	1	0	0

right_ear_speed_mean	kinematics	1	0	0
right_ear_speed_median	kinematics	1	0	0
right_ear_speed_p10	kinematics	1	0	0
right_ear_speed_p90	kinematics	1	0	0
right_ear_speed_periodicity	kinematics	1	0	0
right_ear_speed_std	kinematics	1	0	0
right_ear_velocity_x_energy	kinematics	1	0	0
right_ear_velocity_x_max	kinematics	1	0	0
right_ear_velocity_x_mean	kinematics	1	0	0
right_ear_velocity_x_median	kinematics	1	0	0
right_ear_velocity_x_p10	kinematics	1	0	0
right_ear_velocity_x_p90	kinematics	1	0	0
right_ear_velocity_x_periodicity	kinematics	1	0	0
right_ear_velocity_x_std	kinematics	1	0	0
right_ear_velocity_y_energy	kinematics	1	0	0
right_ear_velocity_y_max	kinematics	1	0	0
right_ear_velocity_y_mean	kinematics	1	0	0
right_ear_velocity_y_median	kinematics	1	0	0
right_ear_velocity_y_p10	kinematics	1	0	0
right_ear_velocity_y_p90	kinematics	1	0	0
right_ear_velocity_y_periodicity	kinematics	1	0	0
right_ear_velocity_y_std	kinematics	1	0	0
roi_1_present_energy	context (ROI/target)	1	1	1
roi_1_present_max	context (ROI/target)	1	1	1
roi_1_present_mean	context (ROI/target)	1	1	1
roi_1_present_median	context (ROI/target)	1	1	1
roi_1_present_p10	context (ROI/target)	1	1	1
roi_1_present_p90	context (ROI/target)	1	1	1
roi_1_present_periodicity	context (ROI/target)	1	1	1
roi_1_present_std	context (ROI/target)	1	1	1
roi_2_present_energy	context (ROI/target)	1	0	0
roi_2_present_max	context (ROI/target)	1	0	0
roi_2_present_mean	context (ROI/target)	1	0	0
roi_2_present_median	context (ROI/target)	1	0	0
roi_2_present_p10	context (ROI/target)	1	0	0
roi_2_present_p90	context (ROI/target)	1	0	0
roi_2_present_periodicity	context (ROI/target)	1	0	0
roi_2_present_std	context (ROI/target)	1	0	0
tail_base_acceleration_energy	kinematics	1	1	1
tail_base_acceleration_max	kinematics	1	1	1
tail_base_acceleration_mean	kinematics	1	1	1
tail_base_acceleration_median	kinematics	1	1	1
tail_base_acceleration_p10	kinematics	1	1	1
tail_base_acceleration_p90	kinematics	1	1	1

tail_base_acceleration_periodicity	kinematics	1	1	1
tail_base_acceleration_std	kinematics	1	1	1
tail_base_forward_velocity_energy	kinematics	1	1	1
tail_base_forward_velocity_max	kinematics	1	1	1
tail_base_forward_velocity_mean	kinematics	1	1	1
tail_base_forward_velocity_median	kinematics	1	1	1
tail_base_forward_velocity_p10	kinematics	1	1	1
tail_base_forward_velocity_p90	kinematics	1	1	1
tail_base_forward_velocity_periodicity	kinematics	1	1	1
tail_base_forward_velocity_std	kinematics	1	1	1
tail_base_jerk_energy	kinematics	1	1	1
tail_base_jerk_max	kinematics	1	1	1
tail_base_jerk_mean	kinematics	1	1	1
tail_base_jerk_median	kinematics	1	1	1
tail_base_jerk_p10	kinematics	1	1	1
tail_base_jerk_p90	kinematics	1	1	1
tail_base_jerk_periodicity	kinematics	1	1	1
tail_base_jerk_std	kinematics	1	1	1
tail_base_lateral_velocity_energy	kinematics	1	1	1
tail_base_lateral_velocity_max	kinematics	1	1	1
tail_base_lateral_velocity_mean	kinematics	1	1	1
tail_base_lateral_velocity_median	kinematics	1	1	1
tail_base_lateral_velocity_p10	kinematics	1	1	1
tail_base_lateral_velocity_p90	kinematics	1	1	1
tail_base_lateral_velocity_periodicity	kinematics	1	1	1
tail_base_lateral_velocity_std	kinematics	1	1	1
tail_base_speed_energy	kinematics	1	1	1
tail_base_speed_max	kinematics	1	1	1
tail_base_speed_mean	kinematics	1	1	1
tail_base_speed_median	kinematics	1	1	1
tail_base_speed_p10	kinematics	1	1	1
tail_base_speed_p90	kinematics	1	1	1
tail_base_speed_periodicity	kinematics	1	1	1
tail_base_speed_std	kinematics	1	1	1
tail_base_velocity_x_energy	kinematics	1	1	1
tail_base_velocity_x_max	kinematics	1	1	1
tail_base_velocity_x_mean	kinematics	1	1	1
tail_base_velocity_x_median	kinematics	1	1	1
tail_base_velocity_x_p10	kinematics	1	1	1
tail_base_velocity_x_p90	kinematics	1	1	1
tail_base_velocity_x_periodicity	kinematics	1	1	1
tail_base_velocity_x_std	kinematics	1	1	1
tail_base_velocity_y_energy	kinematics	1	1	1
tail_base_velocity_y_max	kinematics	1	1	1

tail_base_velocity_y_mean	kinematics	1	1	1
tail_base_velocity_y_median	kinematics	1	1	1
tail_base_velocity_y_p10	kinematics	1	1	1
tail_base_velocity_y_p90	kinematics	1	1	1
tail_base_velocity_y_periodicity	kinematics	1	1	1
tail_base_velocity_y_std	kinematics	1	1	1
uncertainty_entropy	pose geometry	1	1	1
uncertainty_margin	pose geometry	1	1	1
uncertainty_score	pose geometry	1	1	1

HC	NOR	NSF	OF	TMT	n_assays
0	0	0	0	1	1
0	0	0	0	1	1
0	0	0	0	1	1
0	0	0	0	1	1
0	0	0	0	1	1
0	0	0	0	1	1
0	0	0	0	1	1
0	0	0	0	1	1
0	0	0	0	1	1
0	0	0	0	1	1
0	0	0	0	1	1
0	0	0	0	1	1
0	0	0	0	1	1
0	0	0	0	1	1
0	0	0	0	1	1
0	0	0	0	1	1
0	0	0	0	1	1
0	0	0	0	1	1
0	0	0	0	1	1
0	0	0	0	1	1
0	0	0	0	1	1
0	0	0	0	1	1
0	0	0	0	1	1
0	0	0	0	1	1
0	0	0	0	1	1
0	0	0	0	1	1
0	0	0	0	1	1
0	0	0	0	1	1
0	0	0	0	1	1
0	0	0	0	1	1
0	0	0	0	1	1
0	0	0	0	1	1
0	0	0	0	1	1
0	0	0	0	1	1
0	0	0	0	1	1
0	0	0	0	1	1
0	0	0	0	1	1
0	0	0	0	1	1
0	0	0	0	1	1
0	0	0	0	1	1
0	0	0	0	1	1
0	0	0	0	1	1
0	0	0	0	1	1

0	0	0	0	1	1
0	0	0	0	1	1
0	0	0	0	1	1
0	0	0	0	1	1
0	0	0	0	1	1
0	0	0	0	1	1
0	0	0	0	1	1
0	0	0	0	1	1
0	0	0	0	1	1
0	0	0	0	1	1
0	0	0	0	1	1
0	0	0	0	1	1
0	0	0	0	1	1
0	0	0	0	1	1
0	0	0	0	1	1
0	0	0	0	1	1
0	0	0	0	1	1
0	0	0	0	1	1
0	0	0	0	1	1
0	0	0	0	1	1
0	0	0	0	1	1
0	0	0	0	1	1
0	0	0	0	1	1
0	0	0	0	1	1
0	0	0	0	1	1
0	0	0	0	1	1
0	0	0	0	1	1
0	0	0	0	1	1
0	0	0	0	1	1
0	0	0	0	1	1
0	0	0	0	1	1
0	0	0	0	1	1
0	0	0	0	1	1
0	0	0	0	1	1
0	0	0	0	1	1
0	0	0	0	1	1
0	0	0	0	1	1
0	0	0	0	1	1
0	0	0	0	1	1
0	0	0	0	1	1
0	0	0	0	1	1
0	0	0	0	1	1
0	0	0	0	1	1
0	0	0	0	1	1

0	0	0	0	1	1
0	0	0	0	1	1
0	0	0	0	1	1
0	0	0	0	1	1
0	0	0	0	1	1
0	0	0	0	1	1
0	0	0	0	1	1
0	0	0	0	1	1
0	0	0	0	1	1
0	0	0	0	1	1
0	0	0	0	1	1
0	0	0	0	1	1
0	0	0	0	1	1
0	0	0	0	1	1
0	0	0	0	1	1
0	0	0	0	1	1
0	0	0	0	1	1
0	0	0	0	1	1
0	0	0	0	1	1
0	0	0	0	1	1
0	0	0	0	1	1
0	0	0	0	1	1
0	0	0	0	1	1
0	0	0	0	1	1
0	0	0	0	1	1
1	1	1	1	1	8
1	1	1	1	1	8
1	1	1	1	1	8
1	1	1	1	1	8
1	1	1	1	1	8
1	1	1	1	1	8
1	1	1	1	1	8
1	1	1	1	1	8
1	1	1	1	1	8
1	1	1	1	1	8
0	1	0	0	0	2
0	1	0	0	0	2
0	1	0	0	0	2
0	1	0	0	0	2
0	1	0	0	0	2
0	1	0	0	0	2
0	1	0	0	0	2
0	1	0	0	0	2
0	1	0	0	0	2

0	1	0	0	0	2
1	1	1	1	1	8
1	1	1	1	1	8
1	1	1	1	1	8
1	1	1	1	1	8
1	1	1	1	1	8
1	1	1	1	1	8
1	1	1	1	1	8
1	1	1	1	1	8
1	1	1	1	1	8
1	1	1	1	1	8
1	1	1	1	1	8
1	1	1	1	1	8
1	1	1	1	1	8
1	1	1	1	1	8
1	1	1	1	1	8
1	1	1	1	1	8
1	1	1	1	1	8
1	1	1	1	1	8
1	1	1	1	1	8
1	1	1	1	1	8
1	1	1	1	1	8
1	1	1	1	1	8
1	1	1	1	1	8
1	1	1	1	1	8
1	1	1	1	1	8
1	1	1	1	1	8
1	1	1	1	1	8
1	1	1	1	1	8
1	1	1	1	1	8
1	1	1	1	1	8
1	1	1	1	1	8
1	1	1	1	1	8
1	1	1	1	1	8
1	1	1	1	1	8
1	1	1	1	1	8
1	1	1	1	1	8
1	1	1	1	1	8
1	1	1	1	1	8
1	1	1	1	1	8
1	1	1	1	1	8
1	1	1	1	1	8
1	1	1	1	1	8
0	1	0	0	0	2

0	1	0	0	0	2
0	1	0	0	0	2
0	1	0	0	0	2
0	1	0	0	0	2
0	1	0	0	0	2
0	1	0	0	0	2
0	1	0	0	0	2
0	1	0	0	0	2
0	1	0	0	0	2
0	1	0	0	0	2
0	1	0	0	0	2
0	1	0	0	0	2
0	1	0	0	0	2
0	1	0	0	0	2
0	1	0	0	0	2
0	1	0	0	0	2
0	1	0	0	0	2
0	1	0	0	0	2
0	1	0	0	0	2
0	1	0	0	0	2
0	1	0	0	0	2
0	1	0	0	0	2
0	1	0	0	0	2
0	1	0	0	0	2
0	1	0	0	0	2
0	1	0	0	0	2
0	1	0	0	0	2
0	1	0	0	0	2
0	1	0	0	0	2
0	1	0	0	0	2
0	1	0	0	0	2
1	1	1	1	1	8
1	1	1	1	1	8
1	1	1	1	1	8
1	1	1	1	1	8
1	1	1	1	1	8
1	1	1	1	1	8
1	1	1	1	1	8
1	1	1	1	1	8
1	1	1	1	1	8
1	1	1	1	1	8
1	1	1	1	1	8
1	1	1	1	1	8
1	1	1	1	1	8

1	1	1	1	1	8
1	1	1	1	1	8
1	1	1	1	1	8
1	1	1	1	1	8
1	1	1	1	1	8
1	1	1	1	1	8
1	1	1	1	1	8
1	1	1	1	1	8
1	1	1	1	1	8
1	1	1	1	1	8
1	1	1	1	1	8
1	1	1	1	1	8
1	1	1	1	1	8
1	1	1	1	1	8
1	1	1	1	1	8
1	1	1	1	1	8
1	1	1	1	1	8
1	1	1	1	1	8
1	1	1	1	1	8
1	1	1	1	1	8
1	1	1	1	1	8
1	1	1	1	1	8
1	1	1	1	1	8
1	1	1	1	1	8
1	1	1	1	1	8
1	1	1	1	1	8
1	1	1	1	1	8
0	1	0	0	0	2
0	1	0	0	0	2
0	1	0	0	0	2
0	1	0	0	0	2
0	1	0	0	0	2
0	1	0	0	0	2
0	1	0	0	0	2
0	1	0	0	0	2
0	1	0	0	0	2
0	1	0	0	0	2
0	1	0	0	0	2
0	1	0	0	0	2
0	1	0	0	0	2
0	1	0	0	0	2
0	1	0	0	0	2
0	1	0	0	0	2
0	1	0	0	0	2

0	1	0	0	0	2
0	1	0	0	0	2
0	1	0	0	0	2
0	1	0	0	0	2
0	1	0	0	0	2
0	1	0	0	0	2
0	1	0	0	0	2
0	1	0	0	0	2
0	1	0	0	0	2
0	1	0	0	0	2
0	1	0	0	0	2
0	1	0	0	0	2
0	1	0	0	0	2
0	1	0	0	0	2
0	1	0	0	0	2
0	1	0	0	0	2
0	1	0	0	0	2
0	1	0	0	0	2
0	1	0	0	0	2
0	1	0	0	0	2
0	1	0	0	0	2
0	1	0	0	0	2
0	1	0	0	0	2
1	1	1	1	1	8
1	1	1	1	1	8
1	1	1	1	1	8
1	1	1	1	1	8
1	1	1	1	1	8
1	1	1	1	1	8
1	1	1	1	1	8
1	1	1	1	1	8
1	1	1	1	1	8
1	1	1	1	1	8
0	0	0	0	0	1
0	0	0	0	0	1
0	0	0	0	0	1
0	0	0	0	0	1
0	0	0	0	0	1
0	0	0	0	0	1
0	0	0	0	0	1
0	0	0	0	0	1
0	0	0	0	0	1
0	0	0	0	0	1
0	0	0	0	0	1

0	0	0	0	0	1
0	0	0	0	0	1
0	0	0	0	0	1
0	0	0	0	0	1
0	0	0	0	0	1
0	0	0	0	0	1
0	0	0	0	0	1
0	0	0	0	0	1
0	0	0	0	0	1
0	0	0	0	0	1
0	0	0	0	0	1
0	0	0	0	0	1
0	0	0	0	0	1
0	0	0	0	0	1
0	0	0	0	0	1
0	0	0	0	0	1
0	0	0	0	0	1
0	0	0	0	0	1
0	0	0	0	0	1
0	0	0	0	0	1
0	0	0	0	0	1
0	0	0	0	0	1
0	0	0	0	0	1
0	0	0	0	0	1
0	0	0	0	0	1
0	0	0	0	0	1
0	0	0	0	0	1
0	0	0	0	0	1
0	0	0	0	0	1
0	0	0	0	0	1
0	0	0	0	0	1
0	0	0	0	0	1
0	0	0	0	0	1
0	0	0	0	0	1
0	0	0	0	0	1
0	0	0	0	0	1
0	0	0	0	0	1
0	0	0	0	0	1
0	0	0	0	0	1
0	0	0	0	0	1
0	0	0	0	0	1
0	0	0	0	0	1
0	0	0	0	0	1
0	0	0	0	0	1

0	0	0	0	0	1
1	1	1	1	1	8
1	1	1	1	1	8
1	1	1	1	1	8
1	1	1	1	1	8
1	1	1	1	1	8
1	1	1	1	1	8
1	1	1	1	1	8
1	1	1	1	1	8
0	0	0	0	0	1
0	0	0	0	0	1
0	0	0	0	0	1
0	0	0	0	0	1
0	0	0	0	0	1
0	0	0	0	0	1
0	0	0	0	0	1
0	0	0	0	0	1
0	0	0	0	0	1
0	0	0	0	0	1
0	0	0	0	0	1
0	0	0	0	0	1
0	0	0	0	0	1
0	0	0	0	0	1
0	0	0	0	0	1
0	0	0	0	0	1
0	0	0	0	0	1
0	0	0	0	0	1
0	0	0	0	0	1
0	0	0	0	0	1
0	0	0	0	0	1
0	0	0	0	0	1
0	0	0	0	0	1
0	0	0	0	0	1
0	0	0	0	0	1
0	0	0	0	0	1
0	0	0	0	0	1
0	0	0	0	0	1
0	0	0	0	0	1
0	0	0	0	0	1
0	0	0	0	0	1
0	0	0	0	0	1
0	0	0	0	0	1
0	0	0	0	0	1
0	0	0	0	0	1

0	0	0	0	0	1
0	0	0	0	0	1
0	0	0	0	0	1
0	0	0	0	0	1
0	0	0	0	0	1
0	0	0	0	0	1
0	0	0	0	0	1
0	0	0	0	0	1
0	0	0	0	0	1
0	0	0	0	0	1
0	0	0	0	0	1
0	0	0	0	0	1
0	0	0	0	0	1
0	0	0	0	0	1
0	0	0	0	0	1
0	0	0	0	0	1
0	0	0	0	0	1
0	0	0	0	0	1
0	0	0	0	0	1
0	0	0	0	0	1
0	0	0	0	0	1
1	1	1	1	1	8
1	1	1	1	1	8
1	1	1	1	1	8
1	1	1	1	1	8
1	1	1	1	1	8
1	1	1	1	1	8
1	1	1	1	1	8
1	1	1	1	1	8
1	1	1	1	1	8
1	1	1	1	1	8
0	0	0	0	0	1
0	0	0	0	0	1
0	0	0	0	0	1
0	0	0	0	0	1
0	0	0	0	0	1
0	0	0	0	0	1
0	0	0	0	0	1
0	0	0	0	0	1
0	0	0	0	0	1
0	0	0	0	0	1
0	0	0	0	0	1
0	0	0	0	0	1
0	0	0	0	0	1

0	0	0	0	0	1
0	0	0	0	0	1
0	0	0	0	0	1
0	0	0	0	0	1
0	0	0	0	0	1
0	0	0	0	0	1
0	0	0	0	0	1
0	0	0	0	0	1
0	0	0	0	0	1
0	0	0	0	0	1
0	0	0	0	0	1
0	0	0	0	0	1
0	0	0	0	0	1
0	0	0	0	0	1
0	0	0	0	0	1
0	0	0	0	0	1
0	0	0	0	0	1
0	0	0	0	0	1
0	0	0	0	0	1
0	0	0	0	0	1
0	0	0	0	0	1
0	0	0	0	0	1
0	0	0	0	0	1
0	0	0	0	0	1
0	0	0	0	0	1
0	0	0	0	0	1
0	0	0	0	0	1
0	0	0	0	0	1
0	0	0	0	0	1
0	0	0	0	0	1
0	0	0	0	0	1
0	0	0	0	0	1
0	0	0	0	0	1
0	0	0	0	0	1
0	0	0	0	0	1
0	0	0	0	0	1
0	0	0	0	0	1
0	0	0	0	0	1
0	0	0	0	0	1
0	0	0	0	0	1
0	0	0	0	0	1
0	0	0	0	0	1
0	0	0	0	0	1
1	0	0	0	0	2

1	0	0	0	0	2
1	0	0	0	0	2
1	0	0	0	0	2
1	0	0	0	0	2
1	0	0	0	0	2
1	0	0	0	0	2
1	0	0	0	0	2
0	1	1	1	0	4
0	1	1	1	0	4
0	1	1	1	0	4
0	1	1	1	0	4
0	1	1	1	0	4
0	1	1	1	0	4
0	1	1	1	0	4
0	1	1	1	0	4
0	1	1	1	0	4
0	1	1	1	0	4
0	1	1	1	0	4
0	1	1	1	0	4
0	1	1	1	0	4
0	1	1	1	0	4
0	1	1	1	0	4
0	1	1	1	0	4
0	1	1	1	0	4
0	1	1	1	0	4
0	1	1	1	0	4
0	1	1	1	0	4
0	1	1	1	0	4
0	1	1	1	0	4
0	1	1	1	0	4
0	1	1	1	0	4
0	1	1	1	0	4
0	1	1	1	0	4
0	1	1	1	0	4
0	1	1	1	0	4
0	1	1	1	0	4
0	1	1	1	0	4
0	1	1	1	0	4
0	1	1	1	0	4
0	1	1	1	0	4
0	1	1	1	0	4
0	1	1	1	0	4
0	1	1	1	0	4
0	1	1	1	0	4

0	1	1	1	0	4
0	1	1	1	0	4
0	1	1	1	0	4
0	1	1	1	0	4
0	1	1	1	0	4
0	1	1	1	0	4
0	1	1	1	0	4
0	1	1	1	0	4
0	1	1	1	0	4
0	1	1	1	0	4
0	1	1	1	0	4
0	1	1	1	0	4
0	1	1	1	0	4
0	1	1	1	0	4
0	1	1	1	0	4
0	1	1	1	0	4
0	1	1	1	0	4
0	1	1	1	0	4
0	1	1	1	0	4
1	0	0	0	0	2
1	0	0	0	0	2
1	0	0	0	0	2
1	0	0	0	0	2
1	0	0	0	0	2
1	0	0	0	0	2
1	0	0	0	0	2
1	0	0	0	0	2
1	0	0	0	0	2
1	0	0	0	0	2
1	0	0	0	0	2
1	0	0	0	0	2
1	0	0	0	0	2
1	0	0	0	0	2
1	0	0	0	0	2
1	0	0	0	0	2
1	0	0	0	0	2
1	0	0	0	0	2
1	0	0	0	0	2
1	0	0	0	0	2
1	0	0	0	0	2
1	0	0	0	0	2
1	0	0	0	0	2
1	0	0	0	0	2
1	0	0	0	0	2

1	0	0	0	0	2
1	0	0	0	0	2
1	0	0	0	0	2
1	0	0	0	0	2
1	0	0	0	0	2
1	0	0	0	0	2
1	0	0	0	0	2
1	0	0	0	0	2
1	0	0	0	0	2
1	0	0	0	0	2
1	0	0	0	0	2
1	0	0	0	0	2
1	0	0	0	0	2
1	0	0	0	0	2
1	0	0	0	0	2
1	0	0	0	0	2
1	0	0	0	0	2
1	0	0	0	0	2
1	0	0	0	0	2
1	0	0	0	0	2
1	0	0	0	0	2
1	0	0	0	0	2
1	0	0	0	0	2
1	1	1	1	1	8
1	1	1	1	1	8
1	1	1	1	1	8
1	1	1	1	1	8
1	1	1	1	1	8
1	1	1	1	1	8
1	1	1	1	1	8
1	1	1	1	1	8
1	1	1	1	1	8
0	0	0	0	1	1
0	0	0	0	1	1
0	0	0	0	1	1
0	0	0	0	1	1
0	0	0	0	1	1
0	0	0	0	1	1
0	0	0	0	1	1
0	0	0	0	1	1
0	0	0	0	1	1
0	0	0	0	1	1
0	0	0	0	1	1
0	0	0	0	1	1

0	0	0	0	1	1
0	0	0	0	1	1
0	0	0	0	1	1
0	0	0	0	1	1
0	0	0	0	1	1
0	0	0	0	1	1
0	0	0	0	1	1
0	0	0	0	1	1
0	0	0	0	1	1
0	0	0	0	1	1
0	0	0	0	1	1
0	0	0	0	1	1
0	0	0	0	1	1
0	0	0	0	1	1
0	0	0	0	1	1
0	0	0	0	1	1
0	0	0	0	1	1
0	0	0	0	1	1
0	0	0	0	1	1
0	0	0	0	1	1
0	0	0	0	1	1
0	0	0	0	1	1
0	0	0	0	1	1
0	0	0	0	1	1
0	0	0	0	1	1
0	0	0	0	1	1
0	0	0	0	1	1
0	0	0	0	1	1
0	0	0	0	1	1
0	0	0	0	1	1
0	0	0	0	1	1
0	0	0	0	1	1
0	0	0	0	1	1
0	0	0	0	1	1
0	0	0	0	1	1
0	0	0	0	1	1
0	0	0	0	1	1
0	0	0	0	1	1
0	0	0	0	1	1
0	0	0	0	1	1
0	0	0	0	1	1
0	0	0	0	1	1
0	0	0	0	1	1
0	0	0	0	1	1

0	0	0	0	1	1
0	0	0	0	1	1
0	0	0	0	1	1
0	0	0	0	1	1
0	0	0	0	1	1
0	0	0	0	1	1
0	0	0	0	1	1
0	0	0	0	1	1
0	0	0	0	1	1
0	0	0	0	1	1
0	0	0	0	1	1
0	0	0	0	1	1
0	0	0	0	1	1
0	0	0	0	1	1
0	0	0	0	1	1
0	0	0	0	1	1
0	0	0	0	1	1
0	0	0	0	1	1
0	0	0	0	1	1
0	0	0	0	1	1
0	0	0	0	1	1
0	0	0	0	1	1
0	0	0	0	1	1
0	0	0	0	1	1
0	0	0	0	1	1
0	0	0	0	1	1
0	0	0	0	1	1
0	0	0	0	1	1
0	0	0	0	1	1
0	0	0	0	1	1
0	0	0	0	1	1
0	0	0	0	1	1
0	0	0	0	1	1
0	0	0	0	1	1
0	0	0	0	1	1
0	0	0	0	1	1
0	0	0	0	1	1
0	0	0	0	1	1
0	0	0	0	1	1
0	0	0	0	1	1
0	0	0	0	1	1
0	0	0	0	1	1
0	0	0	0	1	1
0	0	0	0	1	1

0	0	0	0	1	1
0	0	0	0	1	1
0	0	0	0	1	1
0	0	0	0	1	1
0	0	0	0	1	1
0	0	0	0	1	1
0	0	0	0	1	1
0	0	0	0	1	1
0	0	0	0	1	1
0	0	0	0	1	1
0	0	0	0	1	1
0	0	0	0	1	1
0	0	0	0	1	1
0	0	0	0	1	1
0	0	0	0	1	1
0	0	0	0	1	1
0	0	0	0	1	1
0	0	0	0	1	1
0	0	0	0	1	1
0	0	0	0	1	1
0	0	0	0	1	1
0	0	0	0	1	1
0	0	0	0	1	1
0	0	0	0	1	1
0	0	0	0	1	1
0	0	0	0	1	1
0	0	0	0	1	1
0	0	0	0	1	1
0	0	0	0	1	1
0	0	0	0	1	1
0	0	0	0	1	1
0	0	0	0	1	1
0	0	0	0	1	1
0	0	0	0	1	1
0	0	0	0	1	1
0	0	0	0	1	1
0	0	0	0	1	1
0	0	0	0	1	1
0	0	0	0	1	1
0	0	0	0	1	1
0	0	0	0	1	1
0	0	0	0	1	1
0	0	0	0	1	1
0	0	0	0	1	1

0	0	0	0	1	1
0	0	0	0	1	1
0	0	0	0	1	1
0	0	0	0	1	1
0	0	0	0	1	1
0	0	0	0	1	1
0	0	0	0	1	1
0	0	0	0	1	1
0	0	0	0	1	1
0	0	0	0	1	1
0	0	0	0	1	1
0	0	0	0	1	1
0	0	0	0	1	1
0	0	0	0	1	1
0	0	0	0	1	1
0	0	0	0	1	1
0	0	0	0	1	1
0	0	0	0	1	1
0	0	0	0	1	1
0	0	0	0	1	1
0	0	0	0	1	1
0	0	0	0	1	1
0	0	0	0	1	1
0	0	0	0	1	1
0	0	0	0	1	1
0	0	0	0	1	1
0	0	0	0	1	1
0	0	0	0	1	1
0	0	0	0	1	1
0	0	0	0	1	1
0	0	0	0	1	1
0	0	0	0	1	1
0	0	0	0	1	1
0	0	0	0	1	1
0	0	0	0	1	1
0	0	0	0	1	1
0	0	0	0	1	1
0	0	0	0	1	1
0	0	0	0	1	1
0	0	0	0	1	1
0	0	0	0	1	1
0	0	0	0	1	1
0	0	0	0	1	1
0	0	0	0	1	1

0	0	0	0	1	1
0	0	0	0	1	1
0	0	0	0	1	1
0	0	0	0	1	1
0	0	0	0	1	1
0	0	0	0	1	1
0	0	0	0	1	1
0	0	0	0	1	1
0	0	0	0	1	1
0	0	0	0	1	1
0	0	0	0	1	1
0	0	0	0	1	1
0	0	0	0	1	1
0	0	0	0	1	1
0	0	0	0	1	1
0	0	0	0	1	1
0	0	0	0	1	1
0	0	0	0	1	1
0	0	0	0	1	1
0	0	0	0	1	1
0	0	0	0	1	1
0	0	0	0	1	1
0	0	0	0	1	1
0	0	0	0	1	1
0	0	0	0	1	1
0	0	0	0	1	1
0	0	0	0	1	1
0	0	0	0	1	1
0	0	0	0	1	1
0	0	0	0	1	1
0	0	0	0	1	1
0	0	0	0	1	1
0	0	0	0	1	1
0	0	0	0	1	1
0	0	0	0	1	1
0	0	0	0	1	1
0	0	0	0	1	1
0	0	0	0	1	1
0	0	0	0	1	1
0	0	0	0	1	1
0	0	0	0	1	1
0	0	0	0	1	1
0	0	0	0	1	1
0	0	0	0	1	1

0	0	0	0	1	1
0	0	0	0	1	1
0	0	0	0	1	1
0	0	0	0	1	1
0	0	0	0	1	1
0	0	0	0	1	1
0	0	0	0	1	1
0	0	0	0	1	1
0	0	0	0	1	1
0	0	0	0	1	1
0	0	0	0	1	1
0	0	0	0	1	1
0	0	0	0	1	1
0	0	0	0	1	1
0	0	0	0	1	1
0	0	0	0	1	1
0	0	0	0	1	1
0	0	0	0	1	1
0	0	0	0	1	1
0	0	0	0	1	1
0	0	0	0	1	1
0	0	0	0	1	1
0	0	0	0	1	1
0	0	0	0	1	1
0	0	0	0	1	1
0	0	0	0	1	1
0	0	0	0	1	1
0	0	0	0	1	1
0	0	0	0	1	1
0	0	0	0	1	1
0	0	0	0	1	1
0	0	0	0	1	1
0	0	0	0	1	1
0	0	0	0	1	1
0	0	0	0	1	1
0	0	0	0	1	1
0	0	0	0	1	1
0	0	0	0	1	1
0	0	0	0	1	1
0	0	0	0	1	1
0	0	0	0	1	1
0	0	0	0	1	1
0	0	0	0	1	1
0	0	0	0	1	1
0	0	0	0	1	1

0	0	0	0	1	1
0	0	0	0	1	1
0	0	0	0	1	1
0	0	0	0	1	1
0	0	0	0	1	1
0	0	0	0	1	1
0	0	0	0	1	1
0	0	0	0	1	1
0	0	0	0	1	1
0	0	0	0	1	1
0	0	0	0	1	1
0	0	0	0	1	1
0	0	0	0	1	1
0	0	0	0	1	1
0	0	0	0	1	1
0	0	0	0	1	1
0	0	0	0	1	1
0	0	0	0	1	1
0	0	0	0	1	1
0	0	0	0	1	1
0	0	0	0	1	1
0	0	0	0	1	1
0	0	0	0	1	1
0	0	0	0	1	1
0	0	0	0	0	1
0	0	0	0	0	1
0	0	0	0	0	1
0	0	0	0	0	1
0	0	0	0	0	1
0	0	0	0	0	1
0	0	0	0	0	1
0	0	0	0	0	1
0	0	0	0	0	1
0	0	0	0	0	1
0	0	0	0	0	1
0	0	0	0	0	1
0	0	0	0	0	1
0	0	0	0	0	1
0	0	0	0	0	1
0	0	0	0	0	1
0	0	0	0	0	1
0	0	0	0	0	1
0	0	0	0	0	1
0	0	0	0	0	1

0	0	0	0	0	1
0	0	0	0	0	1
0	0	0	0	0	1
0	0	0	0	0	1
0	0	0	0	0	1
0	0	0	0	0	1
0	0	0	0	0	1
0	0	0	0	0	1
0	0	0	0	0	1
0	0	0	0	0	1
0	0	0	0	0	1
0	0	0	0	0	1
0	0	0	0	0	1
0	0	0	0	0	1
0	0	0	0	0	1
0	0	0	0	0	1
0	0	0	0	0	1
0	0	0	0	0	1
0	0	0	0	0	1
0	0	0	0	0	1
0	0	0	0	0	1
0	0	0	0	0	1
0	0	0	0	0	1
0	0	0	0	0	1
0	0	0	0	0	1
0	0	0	0	0	1
0	0	0	0	0	1
0	0	0	0	0	1
0	0	0	0	0	1
0	0	0	0	0	1
0	0	0	0	0	1
0	0	0	0	0	1
0	0	0	0	0	1
0	0	0	0	0	1
0	0	0	0	0	1
0	0	0	0	0	1
0	0	0	0	0	1
0	0	0	0	0	1
0	0	0	0	0	1
0	0	0	0	0	1
0	0	0	0	0	1
0	0	0	0	0	1
0	0	0	0	0	1
0	0	0	0	0	1

0	0	0	0	0	1
0	0	0	0	0	1
0	0	0	0	0	1
0	0	0	0	0	1
0	0	0	0	0	1
0	0	0	0	0	1
0	0	0	0	0	1
0	0	0	0	0	1
0	0	0	0	0	1
0	0	0	0	0	1
0	0	0	0	0	1
0	0	0	0	0	1
0	0	0	0	0	1
0	0	0	0	0	1
0	0	0	0	0	1
0	0	0	0	0	1
0	0	0	0	0	1
0	0	0	0	0	1
0	0	0	0	0	1
0	0	0	0	0	1
0	0	0	0	0	1
0	0	0	0	0	1
0	0	0	0	0	1
0	0	0	0	0	1
0	0	0	0	0	1
0	0	0	0	0	1
0	0	0	0	0	1
0	0	0	0	0	1
0	0	0	0	0	1
0	0	0	0	0	1
0	0	0	0	0	1
0	0	0	0	0	1
0	0	0	0	0	1
0	0	0	0	0	1
0	0	0	0	0	1
0	0	0	0	0	1
0	0	0	0	0	1
0	0	0	0	0	1
0	0	0	0	0	1
0	0	0	0	0	1
0	0	0	0	0	1
0	0	0	0	0	1
0	0	0	0	0	1
0	0	0	0	0	1

0	0	0	0	0	1
0	0	0	0	0	1
0	0	0	0	0	1
0	0	0	0	0	1
0	0	0	0	0	1
0	0	0	0	0	1
0	0	0	0	0	1
0	0	0	0	0	1
0	0	0	0	0	1
0	0	0	0	0	1
0	0	0	0	0	1
0	0	0	0	0	1
0	0	0	0	0	1
0	0	0	0	0	1
0	0	0	0	0	1
0	0	0	0	0	1
0	0	0	0	0	1
0	0	0	0	0	1
0	0	0	0	0	1
0	0	0	0	0	1
0	0	0	0	0	1
0	0	0	0	0	1
0	0	0	0	0	1
0	0	0	0	0	1
0	0	0	0	0	1
0	0	0	0	0	1
0	0	0	0	0	1
0	0	0	0	0	1
0	0	0	0	0	1
0	0	0	0	0	1
0	0	0	0	0	1
0	0	0	0	0	1
0	0	0	0	0	1
0	0	0	0	0	1
0	0	0	0	0	1
0	0	0	0	0	1
0	0	0	0	0	1
0	0	0	0	0	1
0	0	0	0	0	1
0	0	0	0	0	1
0	0	0	0	0	1
0	0	0	0	0	1
0	0	0	0	0	1
0	0	0	0	0	1
0	0	0	0	0	1

0	0	0	0	0	1
0	0	0	0	0	1
0	0	0	0	0	1
0	0	0	0	0	1
0	0	0	0	0	1
0	0	0	0	0	1
0	0	0	0	0	1
0	0	0	0	0	1
0	0	0	0	0	1
0	0	0	0	0	1
0	0	0	0	0	1
0	0	0	0	0	1
0	0	0	0	0	1
0	0	0	0	0	1
0	0	0	0	0	1
0	0	0	0	0	1
0	0	0	0	0	1
0	0	0	0	0	1
0	0	0	0	0	1
0	0	0	0	0	1
0	0	0	0	0	1
0	0	0	0	0	1
0	0	0	0	0	1
0	0	0	0	0	1
0	0	0	0	0	1
0	0	0	0	0	1
0	0	0	0	0	1
0	0	0	0	0	1
0	0	0	0	0	1
0	0	0	0	0	1
0	0	0	0	0	1
0	0	0	0	0	1
0	0	0	0	0	1
0	0	0	0	0	1
0	0	0	0	0	1
0	0	0	0	0	1
0	0	0	0	0	1
0	0	0	0	0	1
0	0	0	0	0	1
0	0	0	0	0	1
0	0	0	0	0	1
0	0	0	0	0	1
0	0	0	0	0	1
0	0	0	0	0	1

0	0	0	0	0	1
0	0	0	0	0	1
0	0	0	0	0	1
0	0	0	0	0	1
0	0	0	0	0	1
0	0	0	0	0	1
0	0	0	0	0	1
0	0	0	0	0	1
0	0	0	0	0	1
0	0	0	0	0	1
0	0	0	0	0	1
0	0	0	0	0	1
0	0	0	0	0	1
0	0	0	0	0	1
0	0	0	0	0	1
0	0	0	0	0	1
0	0	0	0	0	1
0	0	0	0	0	1
0	0	0	0	0	1
0	0	0	0	0	1
0	0	0	0	0	1
0	0	0	0	0	1
0	0	0	0	0	1
0	0	0	0	0	1
0	0	0	0	0	1
0	0	0	0	0	1
0	0	0	0	0	1
0	0	0	0	0	1
0	0	0	0	0	1
0	0	0	0	0	1
0	0	0	0	0	1
0	0	0	0	0	1
0	0	0	0	0	1
0	0	0	0	0	1
0	0	0	0	0	1
0	0	0	0	0	1
0	0	0	0	0	1
0	0	0	0	0	1
0	0	0	0	0	1
0	0	0	0	0	1
0	0	0	0	0	1
0	0	0	0	0	1
0	0	0	0	0	1
0	0	0	0	0	1

0	0	0	0	0	1
0	0	0	0	0	1
0	0	0	0	0	1
0	0	0	0	0	1
0	0	0	0	0	1
0	0	0	0	0	1
0	0	0	0	0	1
0	0	0	0	0	1
0	0	0	0	0	1
0	0	0	0	0	1
0	0	0	0	0	1
0	0	0	0	0	1
0	0	0	0	0	1
0	0	0	0	0	1
0	0	0	0	0	1
0	0	0	0	0	1
0	0	0	0	0	1
0	0	0	0	0	1
0	0	0	0	0	1
0	0	0	0	0	1
0	0	0	0	0	1
0	0	0	0	0	1
0	0	0	0	0	1
0	0	0	0	0	1
0	0	0	0	0	1
0	0	0	0	0	1
0	0	0	0	0	1
0	0	0	0	0	1
0	0	0	0	0	1
0	0	0	0	0	1
0	0	0	0	0	1
0	0	0	0	0	1
0	0	0	0	0	1
0	0	0	0	0	1
0	0	0	0	0	1
0	0	0	0	0	1
0	0	0	0	0	1
0	0	0	0	0	1
0	0	0	0	0	1
0	0	0	0	0	1
0	0	0	0	0	1
0	0	0	0	0	1
0	0	0	0	0	1
0	0	0	0	0	1

0	0	0	0	0	1
0	0	0	0	0	1
0	0	0	0	0	1
0	0	0	0	0	1
0	0	0	0	0	1
0	0	0	0	0	1
0	0	0	0	0	1
0	0	0	0	0	1
0	0	0	0	0	1
0	0	0	0	0	1
0	0	0	0	0	1
0	0	0	0	0	1
0	0	0	0	0	1
0	0	0	0	0	1
0	0	0	0	0	1
0	0	0	0	0	1
0	1	1	1	0	4
0	1	1	1	0	4
0	1	1	1	0	4
0	1	1	1	0	4
0	1	1	1	0	4
0	1	1	1	0	4
0	1	1	1	0	4
0	1	1	1	0	4
0	1	1	1	0	4
0	1	1	1	0	4
0	1	1	1	0	4
0	1	1	1	0	4
0	1	1	1	0	4
0	1	1	1	0	4
0	1	1	1	0	4
0	1	1	1	0	4
0	1	1	1	0	4
0	1	1	1	0	4
0	1	1	1	0	4
0	1	1	1	0	4
0	1	1	1	0	4
0	1	1	1	0	4
0	1	1	1	0	4
0	1	1	1	0	4
0	1	1	1	0	4
0	1	1	1	0	4
0	1	1	1	0	4
0	1	1	1	0	4

0	1	1	1	0	4
0	1	1	1	0	4
0	1	1	1	0	4
0	1	1	1	0	4
0	1	1	1	0	4
0	1	1	1	0	4
0	1	1	1	0	4
0	1	1	1	0	4
0	1	1	1	0	4
0	1	1	1	0	4
0	1	1	1	0	4
0	1	1	1	0	4
0	1	1	1	0	4
0	1	1	1	0	4
0	1	1	1	0	4
0	1	1	1	0	4
0	1	1	1	0	4
0	1	1	1	0	4
0	1	1	1	0	4
0	1	1	1	0	4
0	1	1	1	0	4
0	1	1	1	0	4
0	1	1	1	0	4
0	1	1	1	0	4
0	1	1	1	0	4
0	1	1	1	0	4
0	1	1	1	0	4
0	1	1	1	0	4
0	1	1	1	0	4
0	1	1	1	0	4
0	1	1	1	0	4
0	1	1	1	0	4
0	1	1	1	0	4
0	1	1	1	0	4
0	1	1	1	0	4
0	1	1	1	0	4
0	1	1	1	0	4
0	1	1	1	0	4
0	1	1	1	0	4
0	1	1	1	0	4
0	1	1	1	0	4
0	1	1	1	0	4
0	1	1	1	0	4
0	1	1	1	0	4

0	1	1	1	0	4
0	1	1	1	0	4
0	1	1	1	0	4
0	1	1	1	0	4
0	1	1	1	0	4
0	1	1	1	0	4
0	1	1	1	0	4
0	1	1	1	0	4
0	1	1	1	0	4
0	1	1	1	0	4
0	1	1	1	0	4
0	1	1	1	0	4
0	1	1	1	0	4
0	1	1	1	0	4
0	1	1	1	0	4
0	1	1	1	0	4
0	1	1	1	0	4
0	1	1	1	0	4
0	1	1	1	0	4
0	1	1	1	0	4
0	1	1	1	0	4
0	1	1	1	0	4
0	1	1	1	0	4
0	1	1	1	0	4
0	1	1	1	0	4
0	1	1	1	0	4
0	1	1	1	0	4
0	1	1	1	0	4
0	1	1	1	0	4
0	1	1	1	0	4
0	1	1	1	0	4
0	1	1	1	0	4
0	1	1	1	0	4
0	1	1	1	0	4
0	1	1	1	0	4
0	1	1	1	0	4
0	1	1	1	0	4
0	1	1	1	0	4
0	1	1	1	0	4
0	1	1	1	0	4
0	1	1	1	0	4
0	1	1	1	0	4
0	1	1	1	0	4
0	1	1	1	0	4

0	1	1	1	0	4
0	1	1	1	0	4
0	1	1	1	0	4
0	1	1	1	0	4
1	0	0	0	0	2
1	0	0	0	0	2
1	0	0	0	0	2
1	0	0	0	0	2
1	0	0	0	0	2
1	0	0	0	0	2
1	0	0	0	0	2
1	0	0	0	0	2
1	0	0	0	0	2
1	0	0	0	0	2
1	0	0	0	0	2
1	0	0	0	0	2
1	0	0	0	0	2
1	0	0	0	0	2
1	0	0	0	0	2
1	0	0	0	0	2
1	0	0	0	0	2
1	0	0	0	0	2
1	0	0	0	0	2
1	0	0	0	0	2
1	0	0	0	0	2
1	0	0	0	0	2
1	0	0	0	0	2
1	0	0	0	0	2
1	0	0	0	0	2
1	0	0	0	0	2
1	0	0	0	0	2
1	0	0	0	0	2
1	0	0	0	0	2
1	0	0	0	0	2
1	0	0	0	0	2
1	0	0	0	0	2
1	0	0	0	0	2
1	0	0	0	0	2
1	0	0	0	0	2
1	0	0	0	0	2
1	0	0	0	0	2
1	0	0	0	0	2
1	0	0	0	0	2
1	0	0	0	0	2

1	0	0	0	0	2
1	0	0	0	0	2
1	0	0	0	0	2
1	0	0	0	0	2
1	0	0	0	0	2
1	0	0	0	0	2
1	0	0	0	0	2
1	0	0	0	0	2
1	0	0	0	0	2
1	0	0	0	0	2
1	0	0	0	0	2
1	0	0	0	0	2
1	0	0	0	0	2
1	0	0	0	0	2
1	0	0	0	0	2
1	0	0	0	0	2
1	0	0	0	0	2
1	0	0	0	0	2
1	0	0	0	0	2
1	0	0	0	0	2
1	0	0	0	0	2
1	0	0	0	0	2
1	0	0	0	0	2
1	0	0	0	0	2
1	0	0	0	0	2
1	0	0	0	0	2
1	0	0	0	0	2
1	0	0	0	0	2
1	0	0	0	0	2
1	0	0	0	0	2
1	0	0	0	0	2
1	0	0	0	0	2
1	0	0	0	0	2
1	0	0	0	0	2
1	0	0	0	0	2
1	0	0	0	0	2
1	0	0	0	0	2
1	0	0	0	0	2
1	0	0	0	0	2
1	0	0	0	0	2
1	0	0	0	0	2
1	0	0	0	0	2
1	0	0	0	0	2
1	0	0	0	0	2

1	0	0	0	0	2
1	0	0	0	0	2
1	0	0	0	0	2
1	0	0	0	0	2
1	0	0	0	0	2
1	0	0	0	0	2
1	0	0	0	0	2
1	0	0	0	0	2
1	0	0	0	0	2
1	0	0	0	0	2
1	0	0	0	0	2
1	0	0	0	0	2
1	0	0	0	0	2
1	0	0	0	0	2
1	0	0	0	0	2
1	0	0	0	0	2
1	0	0	0	0	2
1	0	0	0	0	2
1	0	0	0	0	2
1	0	0	0	0	2
1	0	0	0	0	2
1	0	0	0	0	2
1	0	0	0	0	2
1	0	0	0	0	2
1	0	0	0	0	2
1	0	0	0	0	2
1	0	0	0	0	2
1	0	0	0	0	2
1	0	0	0	0	2
1	0	0	0	0	2
1	0	0	0	0	2
1	0	0	0	0	2
1	0	0	0	0	2
1	0	0	0	0	2
1	0	0	0	0	2
1	0	0	0	0	2
1	0	0	0	0	3
1	0	0	0	0	3
1	0	0	0	0	3
1	0	0	0	0	3
1	0	0	0	0	3
1	0	0	0	0	3
1	0	0	0	0	3
1	0	0	0	0	3

1	0	0	0	0	3
1	0	0	0	0	3
1	0	0	0	0	3
1	0	0	0	0	3
1	0	0	0	0	3
1	0	0	0	0	3
1	0	0	0	0	3
1	0	0	0	0	3
1	0	0	0	0	3
1	0	0	0	0	3
1	0	0	0	0	3
1	0	0	0	0	3
1	0	0	0	0	2
1	0	0	0	0	2
1	0	0	0	0	2
1	0	0	0	0	2
1	0	0	0	0	2
1	0	0	0	0	2
1	0	0	0	0	2
1	0	0	0	0	2
1	0	0	0	0	2
1	0	0	0	0	2
1	0	0	0	0	2
1	0	0	0	0	2
1	0	0	0	0	2
1	0	0	0	0	2
1	0	0	0	0	2
1	0	0	0	0	2
1	0	0	0	0	2
1	0	0	0	0	2
1	0	0	0	0	2
1	0	0	0	0	2
1	0	0	0	0	2
1	0	0	0	0	2
1	0	0	0	0	2
1	0	0	0	0	2
1	0	0	0	0	2
1	0	0	0	0	2
1	0	0	0	0	2
1	0	0	0	0	2
1	0	0	0	0	2
1	0	0	0	0	2
1	0	0	0	0	2
1	0	0	0	0	2

1	0	0	0	0	2
1	0	0	0	0	2
1	0	0	0	0	2
1	0	0	0	0	2
1	0	0	0	0	2
1	0	0	0	0	2
1	0	0	0	0	2
1	0	0	0	0	2
1	0	0	0	0	3
1	0	0	0	0	3
1	0	0	0	0	3
1	0	0	0	0	3
1	0	0	0	0	3
1	0	0	0	0	3
1	0	0	0	0	3
1	0	0	0	0	3
1	0	0	0	0	3
1	0	0	0	0	3
1	0	0	0	0	3
1	0	0	0	0	3
1	0	0	0	0	3
1	0	0	0	0	3
1	0	0	0	0	3
1	0	0	0	0	3
1	0	0	0	0	3
1	0	0	0	0	3
1	0	0	0	0	3
1	0	0	0	0	3
1	0	0	0	0	3
1	0	0	0	0	3
1	0	0	0	0	3
1	0	0	0	0	3
1	0	0	0	0	3
1	0	0	0	0	3
1	0	0	0	0	3
1	0	0	0	0	3
1	0	0	0	0	3
1	0	0	0	0	3
1	0	0	0	0	3
1	0	0	0	0	3
1	0	0	0	0	3
1	0	0	0	0	3
1	0	0	0	0	3
1	0	0	0	0	3

1	0	0	0	0	3
1	0	0	0	0	3
1	0	0	0	0	3
1	0	0	0	0	3
1	0	0	0	0	2
1	0	0	0	0	2
1	0	0	0	0	2
1	0	0	0	0	2
1	0	0	0	0	2
1	0	0	0	0	2
1	0	0	0	0	2
1	0	0	0	0	2
1	0	0	0	0	2
1	0	0	0	0	2
1	0	0	0	0	2
1	0	0	0	0	2
1	0	0	0	0	2
1	0	0	0	0	2
1	0	0	0	0	2
1	0	0	0	0	2
1	0	0	0	0	2
1	0	0	0	0	2
1	0	0	0	0	2
1	0	0	0	0	2
1	0	0	0	0	2
1	0	0	0	0	2
1	0	0	0	0	2
1	0	0	0	0	2
1	0	0	0	0	2
1	0	0	0	0	2
1	0	0	0	0	2
1	0	0	0	0	2
1	0	0	0	0	2
1	0	0	0	0	2
1	0	0	0	0	2
1	0	0	0	0	2
1	0	0	0	0	2
1	0	0	0	0	2
1	0	0	0	0	2
1	0	0	0	0	2
1	0	0	0	0	2
1	0	0	0	0	2
1	0	0	0	0	2
1	0	0	0	0	2

1	0	0	0	0	3
1	0	0	0	0	3
1	0	0	0	0	3
1	0	0	0	0	3
1	0	0	0	0	3
1	0	0	0	0	3
1	0	0	0	0	3
1	0	0	0	0	3
1	0	0	0	0	3
1	0	0	0	0	3
1	0	0	0	0	3
1	0	0	0	0	3
1	0	0	0	0	3
1	0	0	0	0	3
1	0	0	0	0	3
1	0	0	0	0	3
1	0	0	0	0	3
1	0	0	0	0	3
1	0	0	0	0	3
1	0	0	0	0	3
1	0	0	0	0	3
1	0	0	0	0	3
1	0	0	0	0	3
1	0	0	0	0	3
1	0	0	0	0	3
1	0	0	0	0	3
1	0	0	0	0	3
1	0	0	0	0	3
1	0	0	0	0	3
1	0	0	0	0	3
1	0	0	0	0	3
1	0	0	0	0	3
1	0	0	0	0	3
1	0	0	0	0	3
1	0	0	0	0	3
1	0	0	0	0	3
1	0	0	0	0	3
1	0	0	0	0	3
1	0	0	0	0	3
1	0	0	0	0	3
0	0	0	0	1	1
0	0	0	0	1	1
0	0	0	0	1	1
0	0	0	0	1	1

0	0	0	0	1	1
0	0	0	0	1	1
0	0	0	0	1	1
0	0	0	0	1	1
0	0	0	0	1	1
0	0	0	0	1	1
0	0	0	0	1	1
0	0	0	0	1	1
0	0	0	0	1	1
0	0	0	0	1	1
0	0	0	0	1	1
0	0	0	0	1	1
0	0	0	0	1	1
0	0	0	0	1	1
0	0	0	0	1	1
0	0	0	0	1	1
0	0	0	0	1	1
0	0	0	0	1	1
0	0	0	0	1	1
0	0	0	0	1	1
0	0	0	0	1	1
0	0	0	0	1	1
0	0	0	0	1	1
0	0	0	0	1	1
0	0	0	0	1	1
0	0	0	0	1	1
0	0	0	0	1	1
0	0	0	0	1	1
0	0	0	0	1	1
0	0	0	0	1	1
0	0	0	0	1	1
0	0	0	0	1	1
0	0	0	0	1	1
0	0	0	0	1	1
0	0	0	0	1	1
0	0	0	0	1	1
0	0	0	0	1	1
0	0	0	0	1	1
0	0	0	0	1	1
0	0	0	0	1	1
0	0	0	0	1	1
0	0	0	0	1	1
0	0	0	0	1	1
0	0	0	0	1	1

0	0	0	0	1	1
0	0	0	0	1	1
0	0	0	0	1	1
0	0	0	0	1	1
0	0	0	0	1	1
0	0	0	0	1	1
0	0	0	0	1	1
0	0	0	0	1	1
0	0	0	0	1	1
0	0	0	0	1	1
0	0	0	0	1	1
0	0	0	0	1	1
0	0	0	0	1	1
0	0	0	0	1	1
0	0	0	0	1	1
0	0	0	0	1	1
0	0	0	0	1	1
0	0	0	0	1	1
0	0	0	0	1	1
0	0	0	0	1	1
0	0	0	0	1	1
0	0	0	0	1	1
0	0	0	0	1	1
0	0	0	0	1	1
0	0	0	0	1	1
0	0	0	0	1	1
0	0	0	0	1	1
0	0	0	0	1	1
0	0	0	0	1	1
0	0	0	0	1	1
0	0	0	0	1	1
0	0	0	0	1	1
0	0	0	0	1	1
0	0	0	0	1	1
0	0	0	0	1	1
0	0	0	0	1	1
0	0	0	0	1	1
0	0	0	0	1	1
0	0	0	0	1	1
0	0	0	0	1	1
0	0	0	0	1	1
0	0	0	0	1	1
0	0	0	0	1	1
0	0	0	0	1	1

0	0	0	0	1	1
0	0	0	0	1	1
0	0	0	0	1	1
0	0	0	0	1	1
0	0	0	0	1	1
0	0	0	0	1	1
0	0	0	0	1	1
0	0	0	0	1	1
0	0	0	0	1	1
0	0	0	0	1	1
0	0	0	0	1	1
0	0	0	0	1	1
0	0	0	0	1	1
0	0	0	0	1	1
0	0	0	0	1	1
0	0	0	0	1	1
0	0	0	0	1	1
0	0	0	0	1	1
0	0	0	0	1	1
0	0	0	0	1	1
0	0	0	0	1	1
0	0	0	0	1	1
0	0	0	0	1	1
0	0	0	0	1	1
0	0	0	0	1	1
0	0	0	0	1	1
0	0	0	0	1	1
0	0	0	0	1	1
0	0	0	0	1	1
0	0	0	0	1	1
0	0	0	0	1	1
0	0	0	0	1	1
0	0	0	0	1	1
0	0	0	0	1	1
0	0	0	0	1	1
0	0	0	0	1	1
0	0	0	0	1	1
0	0	0	0	1	1
0	0	0	0	1	1
0	0	0	0	1	1
0	0	0	0	1	1
0	0	0	0	1	1
0	0	0	0	1	1
0	0	0	0	1	1

0	0	0	0	1	1
0	0	0	0	1	1
0	0	0	0	1	1
0	0	0	0	1	1
0	0	0	0	1	1
0	0	0	0	1	1
0	0	0	0	1	1
0	0	0	0	1	1
0	0	0	0	1	1
0	0	0	0	1	1
0	0	0	0	1	1
0	0	0	0	1	1
0	0	0	0	1	1
0	0	0	0	1	1
0	0	0	0	1	1
0	0	0	0	1	1
0	0	0	0	1	1
0	0	0	0	1	1
0	0	0	0	1	1
0	0	0	0	1	1
0	0	0	0	1	1
0	0	0	0	1	1
0	0	0	0	1	1
0	0	0	0	1	1
0	0	0	0	1	1
0	0	0	0	1	1
0	0	0	0	1	1
0	0	0	0	1	1
0	0	0	0	1	1
0	0	0	0	1	1
0	0	0	0	1	1
0	0	0	0	1	1
0	0	0	0	1	1
0	0	0	0	1	1
0	0	0	0	1	1
0	0	0	0	1	1
0	0	0	0	1	1
0	0	0	0	1	1
0	0	0	0	1	1
0	0	0	0	1	1
0	0	0	0	1	1
0	0	0	0	1	1
0	0	0	0	1	1
0	0	0	0	1	1
0	0	0	0	1	1

0	0	0	0	1	1
0	0	0	0	1	1
0	0	0	0	1	1
0	0	0	0	1	1
0	0	0	0	1	1
0	0	0	0	1	1
0	0	0	0	1	1
0	0	0	0	1	1
0	0	0	0	1	1
0	0	0	0	1	1
0	0	0	0	1	1
0	0	0	0	1	1
0	0	0	0	1	1
0	0	0	0	1	1
0	0	0	0	1	1
0	0	0	0	1	1
0	0	0	0	1	1
0	0	0	0	1	1
0	0	0	0	1	1
0	0	0	0	1	1
0	0	0	0	1	1
0	0	0	0	1	1
0	0	0	0	1	1
0	0	0	0	1	1
0	0	0	0	1	1
0	0	0	0	1	1
0	0	0	0	1	1
0	0	0	0	1	1
0	0	0	0	1	1
0	0	0	0	1	1
0	0	0	0	1	1
0	0	0	0	1	1
0	0	0	0	1	1
0	0	0	0	1	1
0	0	0	0	1	1
0	0	0	0	1	1
0	0	0	0	1	1
0	0	0	0	1	1
0	0	0	0	1	1
0	0	0	0	1	1
1	0	0	0	0	2
1	0	0	0	0	2
1	0	0	0	0	2
1	0	0	0	0	2

1	0	0	0	0	2
1	0	0	0	0	2
1	0	0	0	0	2
1	0	0	0	0	2
1	0	0	0	0	2
1	0	0	0	0	2
1	0	0	0	0	2
1	0	0	0	0	2
1	0	0	0	0	2
1	0	0	0	0	2
1	0	0	0	0	2
1	0	0	0	0	2
1	0	0	0	0	2
1	0	0	0	0	2
1	0	0	0	0	2
1	0	0	0	0	2
1	0	0	0	0	2
1	0	0	0	0	2
1	0	0	0	0	2
1	0	0	0	0	2
1	0	0	0	0	2
1	0	0	0	0	2
1	0	0	0	0	2
1	0	0	0	0	2
1	0	0	0	0	2
1	0	0	0	0	2
1	0	0	0	0	2
1	0	0	0	0	2
1	0	0	0	0	2
1	0	0	0	0	2
1	0	0	0	0	2
1	0	0	0	0	2
1	0	0	0	0	2
1	0	0	0	0	2
1	0	0	0	0	2
1	0	0	0	0	2
1	0	0	0	0	2
1	0	0	0	0	2
1	0	0	0	0	2
1	0	0	0	0	2
1	0	0	0	0	2
1	0	0	0	0	2
1	0	0	0	0	2
1	0	0	0	0	2

1	0	0	0	0	2
1	0	0	0	0	2
1	0	0	0	0	2
1	0	0	0	0	2
1	0	0	0	0	2
1	0	0	0	0	2
1	0	0	0	0	2
1	0	0	0	0	2
1	0	0	0	0	2
1	0	0	0	0	2
1	0	0	0	0	2
1	0	0	0	0	2
1	0	0	0	0	2
1	0	0	0	0	2
1	0	0	0	0	2
1	0	0	0	0	2
1	0	0	0	0	2
1	0	0	0	0	2
1	0	0	0	0	2
1	0	0	0	0	2
1	0	0	0	0	2
1	0	0	0	0	2
1	0	0	0	0	2
1	0	0	0	0	2
1	0	0	0	0	2
1	0	0	0	0	2
1	0	0	0	0	2
1	0	0	0	0	2
1	0	0	0	0	2
1	0	0	0	0	2
1	0	0	0	0	2
1	0	0	0	0	2
1	0	0	0	0	2
1	0	0	0	0	2
1	0	0	0	0	2
1	0	0	0	0	2
1	0	0	0	0	2
1	0	0	0	0	2
1	0	0	0	0	2
1	0	0	0	0	2
1	0	0	0	0	2
1	0	0	0	0	2
1	0	0	0	0	2
1	0	0	0	0	2

1	0	0	0	0	2
1	0	0	0	0	2
1	0	0	0	0	2
1	0	0	0	0	2
1	0	0	0	0	2
1	0	0	0	0	2
1	0	0	0	0	2
1	0	0	0	0	2
0	1	1	1	0	4
0	1	1	1	0	4
0	1	1	1	0	4
0	1	1	1	0	4
0	1	1	1	0	4
0	1	1	1	0	4
0	1	1	1	0	4
0	1	1	1	0	4
0	1	1	1	0	4
0	1	1	1	0	4
0	1	1	1	0	4
0	1	1	1	0	4
0	1	1	1	0	4
0	1	1	1	0	4
0	1	1	1	0	4
0	1	1	1	0	4
0	1	1	1	0	4
0	1	1	1	0	4
0	1	1	1	0	4
0	1	1	1	0	4
0	1	1	1	0	4
0	1	1	1	0	4
0	1	1	1	0	4
0	1	1	1	0	4
0	1	1	1	0	4
0	1	1	1	0	4
0	1	1	1	0	4
0	1	1	1	0	4
0	1	1	1	0	4
0	1	1	1	0	4
0	1	1	1	0	4
0	1	1	1	0	4
0	1	1	1	0	4
0	1	1	1	0	4
0	1	1	1	0	4
0	1	1	1	0	4

0	1	1	1	0	4
0	1	1	1	0	4
0	1	1	1	0	4
0	1	1	1	0	4
0	1	1	1	0	4
0	1	1	1	0	4
0	1	1	1	0	4
0	1	1	1	0	4
0	1	1	1	0	4
0	1	1	1	0	4
0	1	1	1	0	4
0	1	1	1	0	4
0	1	1	1	0	4
0	1	1	1	0	4
0	1	1	1	0	4
0	1	1	1	0	4
0	1	1	1	0	4
0	1	1	1	0	4
0	1	1	1	0	4
0	1	1	1	0	4
0	1	1	1	0	4
0	1	1	1	0	4
0	1	1	1	0	4
0	1	1	1	0	4
0	1	1	1	0	4
0	1	1	1	0	4
0	1	1	1	0	4
0	1	1	1	0	4
0	1	1	1	0	4
0	1	1	1	0	4
0	1	1	1	0	4
0	1	1	1	0	4
0	1	1	1	0	4
0	1	1	1	0	4
0	1	1	1	0	4
0	1	1	1	0	4
0	1	1	1	0	4
0	1	1	1	0	4
0	1	1	1	0	4
0	1	1	1	0	4
0	1	1	1	0	4
0	1	1	1	0	4
0	1	1	1	0	4
0	1	1	1	0	4

0	1	1	1	0	4
0	1	1	1	0	4
0	1	1	1	0	4
0	1	1	1	0	4
0	1	1	1	0	4
0	1	1	1	0	4
0	1	1	1	0	4
0	1	1	1	0	4
0	1	1	1	0	4
0	1	1	1	0	4
0	1	1	1	0	4
0	1	1	1	0	4
0	1	1	1	0	4
0	1	1	1	0	4
0	1	1	1	0	4
0	1	1	1	0	4
0	1	1	1	0	4
0	1	1	1	0	4
0	1	1	1	0	4
0	1	1	1	0	4
0	0	0	0	1	1
0	0	0	0	1	1
0	0	0	0	1	1
0	0	0	0	1	1
0	0	0	0	1	1
0	0	0	0	1	1
0	0	0	0	1	1
0	0	0	0	1	1
0	0	0	0	1	1
0	0	0	0	1	1
0	0	0	0	1	1
0	0	0	0	1	1
0	0	0	0	1	1
0	0	0	0	1	1
0	0	0	0	1	1
0	0	0	0	1	1
0	0	0	0	1	1
0	0	0	0	1	1
0	0	0	0	1	1
0	0	0	0	1	1
0	1	1	1	1	5
0	1	1	1	1	5
0	1	1	1	1	5
0	1	1	1	1	5

0	1	1	1	1	5
0	1	1	1	1	5
0	1	1	1	1	5
0	1	1	1	1	5
0	1	1	1	1	5
0	1	1	1	1	5
0	1	1	1	1	5
0	1	1	1	1	5
0	1	1	1	1	5
0	1	1	1	1	5
0	1	1	1	1	5
0	1	1	1	1	5
0	1	1	1	1	5
0	1	1	1	1	5
0	1	1	1	1	5
0	1	1	1	1	5
0	1	1	1	0	4
0	1	1	1	0	4
0	1	1	1	0	4
0	1	1	1	0	4
0	1	1	1	0	4
0	1	1	1	0	4
0	1	1	1	0	4
0	1	1	1	0	4
0	1	1	1	0	4
0	1	1	1	0	4
0	1	1	1	0	4
0	1	1	1	0	4
0	1	1	1	0	4
0	1	1	1	0	4
0	1	1	1	0	4
0	1	1	1	0	4
0	1	1	1	0	4
0	1	1	1	0	4
0	1	1	1	0	4
0	1	1	1	0	4
0	1	1	1	1	5
0	1	1	1	1	5
0	1	1	1	1	5
0	1	1	1	1	5
0	1	1	1	1	5
0	1	1	1	1	5
0	1	1	1	1	5
0	1	1	1	1	5

0	1	1	1	1	5
0	1	1	1	1	5
0	1	1	1	1	5
0	1	1	1	1	5
0	1	1	1	1	5
0	1	1	1	1	5
0	1	1	1	1	5
0	1	1	1	1	5
0	1	1	1	1	5
0	1	1	1	1	5
0	1	1	1	1	5
0	1	1	1	1	5
0	1	1	1	1	5
0	1	1	1	1	5
0	1	1	1	1	5
0	1	1	1	1	5
0	1	1	1	1	5
0	1	1	1	1	5
0	1	1	1	1	5
0	1	1	1	1	5
0	1	1	1	1	5
0	1	1	1	1	5
0	1	1	1	1	5
0	1	1	1	1	5
0	1	1	1	1	5
0	1	1	1	1	5
0	1	1	1	1	5
0	1	1	1	1	5
0	1	1	1	1	5
0	1	1	1	1	5
0	1	1	1	1	5
0	1	1	1	1	5
0	0	0	0	1	1
0	0	0	0	1	1
0	0	0	0	1	1
0	0	0	0	1	1
0	0	0	0	1	1
0	0	0	0	1	1
0	0	0	0	1	1
0	0	0	0	1	1
0	0	0	0	1	1
0	0	0	0	1	1
0	0	0	0	1	1
0	0	0	0	1	1

0	0	0	0	1	1
0	0	0	0	1	1
0	0	0	0	1	1
0	0	0	0	1	1
0	0	0	0	1	1
0	0	0	0	1	1
0	0	0	0	1	1
0	0	0	0	1	1
0	0	0	0	1	1
0	0	0	0	1	1
0	0	0	0	1	1
0	0	0	0	1	1
0	0	0	0	1	1
0	0	0	0	1	1
0	0	0	0	1	1
0	0	0	0	1	1
0	0	0	0	1	1
0	0	0	0	1	1
0	0	0	0	1	1
0	0	0	0	1	1
0	0	0	0	1	1
0	0	0	0	1	1
0	0	0	0	1	1
0	0	0	0	1	1
0	0	0	0	1	1
0	0	0	0	1	1
0	0	0	0	1	1
0	0	0	0	1	1
0	0	0	0	1	1
0	0	0	0	1	1
0	0	0	0	1	1
0	0	0	0	1	1
0	0	0	0	1	1
0	0	0	0	1	1
0	0	0	0	1	1
0	0	0	0	1	1
0	0	0	0	1	1
0	0	0	0	1	1
0	0	0	0	1	1
0	0	0	0	1	1
0	0	0	0	1	1
0	0	0	0	1	1
0	0	0	0	1	1
0	0	0	0	1	1
0	0	0	0	1	1

0	0	0	0	1	1
0	0	0	0	1	1
0	0	0	0	1	1
0	0	0	0	1	1
0	1	1	1	0	4
0	1	1	1	0	4
0	1	1	1	0	4
0	1	1	1	0	4
0	1	1	1	0	4
0	1	1	1	0	4
0	1	1	1	0	4
0	1	1	1	0	4
0	1	1	1	0	4
0	1	1	1	0	4
0	1	1	1	0	4
0	1	1	1	0	4
0	1	1	1	0	4
0	1	1	1	0	4
0	1	1	1	0	4
0	1	1	1	0	4
0	1	1	1	0	4
0	1	1	1	0	4
0	1	1	1	0	4
0	1	1	1	0	4
0	1	1	1	1	5
0	1	1	1	1	5
0	1	1	1	1	5
0	1	1	1	1	5
0	1	1	1	1	5
0	1	1	1	1	5
0	1	1	1	1	5
0	1	1	1	1	5
0	1	1	1	1	5
0	1	1	1	1	5
0	1	1	1	1	5
0	1	1	1	1	5
0	1	1	1	1	5
0	1	1	1	1	5
0	1	1	1	1	5
0	1	1	1	1	5
0	1	1	1	1	5
0	1	1	1	1	5
0	1	1	1	1	5
0	1	1	1	1	5

1	1	1	1	1	8
1	1	1	1	1	8
1	1	1	1	1	8
1	1	1	1	1	8
1	1	1	1	1	8
1	1	1	1	1	8
1	1	1	1	1	8
1	1	1	1	1	8
1	1	1	1	1	8
1	1	1	1	1	8
0	1	1	1	0	4
0	1	1	1	0	4
0	1	1	1	0	4
0	1	1	1	0	4
0	1	1	1	0	4
0	1	1	1	0	4
0	1	1	1	0	4
0	1	1	1	0	4
0	1	1	1	0	4
0	1	1	1	0	4
0	1	1	1	0	4
0	1	1	1	0	4
0	1	1	1	0	4
0	1	1	1	0	4
0	1	1	1	0	4
0	1	1	1	0	4
0	1	1	1	0	4
0	1	1	1	0	4
0	1	1	1	0	4
0	1	1	1	0	4
0	1	1	1	0	4
0	1	1	1	0	4
0	1	1	1	0	4
0	1	1	1	0	4
0	1	1	1	0	4
0	1	1	1	0	4
0	1	1	1	0	4
0	1	1	1	0	4
0	1	1	1	0	4
0	1	1	1	0	4
0	1	1	1	0	4
0	1	1	1	0	4
0	1	1	1	0	4
0	1	1	1	0	4

0	1	1	1	0	4
0	1	1	1	0	4
0	1	1	1	0	4
0	1	1	1	0	4
0	1	1	1	0	4
0	1	1	1	0	4
0	1	1	1	1	5
0	1	1	1	1	5
0	1	1	1	1	5
0	1	1	1	1	5
0	1	1	1	1	5
0	1	1	1	1	5
0	1	1	1	1	5
0	1	1	1	1	5
0	1	1	1	1	5
0	1	1	1	1	5
0	1	1	1	1	5
0	1	1	1	1	5
0	1	1	1	1	5
0	1	1	1	1	5
0	1	1	1	1	5
0	1	1	1	1	5
0	1	1	1	1	5
0	1	1	1	1	5
0	1	1	1	1	5
0	1	1	1	1	5
1	0	0	0	0	3
1	0	0	0	0	3
1	0	0	0	0	3
1	0	0	0	0	3
1	0	0	0	0	3
1	0	0	0	0	3
1	0	0	0	0	3
1	0	0	0	0	3
1	0	0	0	0	3
1	0	0	0	0	3
1	0	0	0	0	3
1	0	0	0	0	3
1	0	0	0	0	3
1	0	0	0	0	3
1	0	0	0	0	3
1	0	0	0	0	3
1	0	0	0	0	3
1	0	0	0	0	3

1	0	0	0	0	3
1	0	0	0	0	3
1	0	0	0	0	3
1	0	0	0	0	3
1	0	0	0	0	3
1	0	0	0	0	3
1	0	0	0	0	3
1	0	0	0	0	3
1	0	0	0	0	3
1	0	0	0	0	3
1	0	0	0	0	3
1	0	0	0	0	3
1	0	0	0	0	3
1	0	0	0	0	3
1	0	0	0	0	3
1	0	0	0	0	3
1	0	0	0	0	3
1	0	0	0	0	3
1	0	0	0	0	3
1	0	0	0	0	3
1	0	0	0	0	3
1	0	0	0	0	3
1	0	0	0	0	3
1	0	0	0	0	3
1	0	0	0	0	3
1	0	0	0	0	3
1	0	0	0	0	3
1	0	0	0	0	3
1	0	0	0	0	3
1	0	0	0	0	3
1	0	0	0	0	3
1	0	0	0	0	3
1	0	0	0	0	3
1	0	0	0	0	3
1	0	0	0	0	3
1	0	0	0	0	3
1	0	0	0	0	3
1	0	0	0	0	3
1	0	0	0	0	3
1	0	0	0	0	3
1	0	0	0	0	3
1	0	0	0	0	3
1	0	0	0	0	3
1	0	0	0	0	3

1	0	0	0	0	3
1	0	0	0	0	3
1	0	0	0	0	3
1	0	0	0	0	3
1	0	0	0	0	3
1	0	0	0	0	3
1	0	0	0	0	3
1	0	0	0	0	3
1	0	0	0	0	3
1	0	0	0	0	3
1	0	0	0	0	3
1	0	0	0	0	3
1	0	0	0	0	3
1	0	0	0	0	3
1	0	0	0	0	3
1	0	0	0	0	3
1	0	0	0	0	3
1	0	0	0	0	3
1	0	0	0	0	3
1	0	0	0	0	3
1	0	0	0	0	3
1	0	0	0	0	3
1	0	0	0	0	3
1	0	0	0	0	3
1	0	0	0	0	3
1	0	0	0	0	3
1	0	0	0	0	3
1	0	0	0	0	3
1	0	0	0	0	3
1	0	0	0	0	3
1	0	0	0	0	3
1	0	0	0	0	3
1	0	0	0	0	3
1	0	0	0	0	3
1	0	0	0	0	3
1	0	0	0	0	3
1	0	0	0	0	3
1	0	0	0	0	3
1	0	0	0	0	3
1	0	0	0	0	3
1	0	0	0	0	3
1	0	0	0	0	3
1	0	0	0	0	3
1	0	0	0	0	3

1	0	0	0	0	3
1	0	0	0	0	3
1	0	0	0	0	3
1	0	0	0	0	3
1	0	0	0	0	3
1	0	0	0	0	3
1	1	1	1	1	8
1	1	1	1	1	8
1	1	1	1	1	8
1	1	1	1	1	8
1	1	1	1	1	8
1	1	1	1	1	8
1	1	1	1	1	8
1	1	1	1	1	8
1	1	1	1	1	8
1	1	1	1	1	8
1	1	1	1	1	8
1	1	1	1	1	8
1	1	1	1	1	8
1	1	1	1	1	8
1	1	1	1	1	8
1	1	1	1	1	8
1	1	1	1	1	8
1	1	1	1	1	8
1	1	1	1	1	8
1	1	1	1	1	8
1	1	1	1	1	8
1	1	1	1	1	8
1	1	1	1	1	8
1	1	1	1	1	8
1	1	1	1	1	8
1	1	1	1	1	8
1	1	1	1	1	8
1	1	1	1	1	8
1	1	1	1	1	8
1	1	1	1	1	8
1	1	1	1	1	8
1	1	1	1	1	8
1	1	1	1	1	8
1	1	1	1	1	8
1	1	1	1	1	8
1	1	1	1	1	8
1	1	1	1	1	8
1	1	1	1	1	8

1	1	1	1	1	8
1	1	1	1	1	8
0	1	0	0	0	2
0	1	0	0	0	2
0	1	0	0	0	2
0	1	0	0	0	2
0	1	0	0	0	2
0	1	0	0	0	2
0	1	0	0	0	2
0	1	0	0	0	2
1	1	1	1	1	8
1	1	1	1	1	8
1	1	1	1	1	8
1	1	1	1	1	8
1	1	1	1	1	8
1	1	1	1	1	8
1	1	1	1	1	8
1	1	1	1	1	8
1	1	1	1	1	8
1	1	1	1	1	8
1	1	1	1	1	8
1	1	1	1	1	8
1	1	1	1	1	8
1	1	1	1	1	8
1	1	1	1	1	8
1	1	1	1	1	8
1	1	1	1	1	8
1	1	1	1	1	8
1	1	1	1	1	8
1	1	1	1	1	8
1	1	1	1	1	8
1	1	1	1	1	8
1	1	1	1	1	8
1	1	1	1	1	8
1	1	1	1	1	8
1	1	1	1	1	8
1	1	1	1	1	8
1	1	1	1	1	8
1	1	1	1	1	8
1	1	1	1	1	8
1	1	1	1	1	8
1	1	1	1	1	8
1	1	1	1	1	8
1	1	1	1	1	8

1	1	1	1	1	8
1	1	1	1	1	8
1	1	1	1	1	8
1	1	1	1	1	8
1	1	1	1	1	8
1	1	1	1	1	8
1	1	1	1	1	8
1	1	1	1	1	8
1	1	1	1	1	8
1	1	1	1	1	8
1	1	1	1	1	8
1	1	1	1	1	8
1	1	1	1	1	8
1	1	1	1	1	8
1	1	1	1	1	8
1	1	1	1	1	8
1	1	1	1	1	8
1	1	1	1	1	8
1	1	1	1	1	8
1	1	1	1	1	8
1	1	1	1	1	8
1	1	1	1	1	8
1	1	1	1	1	8
1	1	1	1	1	8
1	1	1	1	1	8
1	1	1	1	1	8
1	1	1	1	1	8
1	1	1	1	1	8
1	1	1	1	1	8
1	1	1	1	1	8
0	1	0	0	0	2
0	1	0	0	0	2
0	1	0	0	0	2
0	1	0	0	0	2
0	1	0	0	0	2
0	1	0	0	0	2
0	1	0	0	0	2
0	1	0	0	0	2
0	1	0	0	0	2
0	1	0	0	0	2
0	1	0	0	0	2
0	1	0	0	0	2
0	1	0	0	0	2
0	1	0	0	0	2

0	1	0	0	0	2
0	1	0	0	0	2
0	1	0	0	0	2
0	1	0	0	0	2
0	1	0	0	0	2
0	1	0	0	0	2
0	1	0	0	0	2
0	1	0	0	0	2
0	1	0	0	0	2
0	1	0	0	0	2
0	1	0	0	0	2
0	1	0	0	0	2
0	1	0	0	0	2
0	1	0	0	0	2
0	1	0	0	0	2
0	1	0	0	0	2
0	1	0	0	0	2
0	1	0	0	0	2
1	1	1	1	1	8
1	1	1	1	1	8
1	1	1	1	1	8
1	1	1	1	1	8
1	1	1	1	1	8
1	1	1	1	1	8
1	1	1	1	1	8
1	1	1	1	1	8
1	1	1	1	1	8
1	1	1	1	1	8
1	1	1	1	1	8
1	1	1	1	1	8
1	1	1	1	1	8
1	1	1	1	1	8
1	1	1	1	1	8
1	1	1	1	1	8
1	1	1	1	1	8
1	1	1	1	1	8
1	1	1	1	1	8
1	1	1	1	1	8
1	1	1	1	1	8
1	1	1	1	1	8
1	1	1	1	1	8
1	1	1	1	1	8
1	1	1	1	1	8
1	1	1	1	1	8

1	1	1	1	1	8
1	1	1	1	1	8
1	1	1	1	1	8
1	1	1	1	1	8
1	1	1	1	1	8
1	1	1	1	1	8
1	1	1	1	1	8
1	1	1	1	1	8
1	1	1	1	1	8
1	1	1	1	1	8
1	1	1	1	1	8
1	1	1	1	1	8
1	1	1	1	1	8
1	1	1	1	1	8
0	1	0	0	0	2
0	1	0	0	0	2
0	1	0	0	0	2
0	1	0	0	0	2
0	1	0	0	0	2
0	1	0	0	0	2
0	1	0	0	0	2
0	1	0	0	0	2
0	1	0	0	0	2
0	1	0	0	0	2
0	1	0	0	0	2
0	1	0	0	0	2
0	1	0	0	0	2
0	1	0	0	0	2
0	1	0	0	0	2
0	1	0	0	0	2
0	1	0	0	0	2
0	1	0	0	0	2
0	1	0	0	0	2
0	1	0	0	0	2
0	1	0	0	0	2
0	1	0	0	0	2
0	1	0	0	0	2
0	1	0	0	0	2
0	1	0	0	0	2
0	1	0	0	0	2
0	1	0	0	0	2
0	1	0	0	0	2
0	1	0	0	0	2
0	1	0	0	0	2

0	1	0	0	0	2
0	1	0	0	0	2
0	1	0	0	0	2
0	1	0	0	0	2
0	1	0	0	0	2
0	1	0	0	0	2
0	1	0	0	0	2
0	1	0	0	0	2
0	1	0	0	0	2
0	1	0	0	0	2
1	1	1	1	1	8
1	1	1	1	1	8
1	1	1	1	1	8
1	1	1	1	1	8
1	1	1	1	1	8
1	1	1	1	1	8
1	1	1	1	1	8
1	1	1	1	1	8
1	1	1	1	1	8
1	1	1	1	1	8
1	1	1	1	1	8
1	1	1	1	1	8
1	1	1	1	1	8
1	1	1	1	1	8
1	1	1	1	1	8
1	1	1	1	1	8
1	1	1	1	1	8
1	1	1	1	1	8
1	1	1	1	1	8
1	1	1	1	1	8
1	1	1	1	1	8
1	1	1	1	1	8
1	1	1	1	1	8
1	1	1	1	1	8
1	1	1	1	1	8
1	1	1	1	1	8
1	1	1	1	1	8
1	1	1	1	1	8
1	1	1	1	1	8
1	1	1	1	1	8
1	1	1	1	1	8
1	1	1	1	1	8
1	1	1	1	1	8
1	1	1	1	1	8

1	1	1	1	1	8
1	1	1	1	1	8
1	1	1	1	1	8
1	1	1	1	1	8
1	1	1	1	1	8
1	1	1	1	1	8
1	1	1	1	1	8
1	1	1	1	1	8
0	0	0	0	1	1
0	0	0	0	1	1
0	0	0	0	1	1
0	0	0	0	1	1
0	0	0	0	1	1
0	0	0	0	1	1
0	0	0	0	1	1
0	0	0	0	1	1
0	0	0	0	1	1
0	0	0	0	1	1
0	0	0	0	1	1
0	0	0	0	1	1
0	0	0	0	1	1
0	0	0	0	1	1
0	0	0	0	1	1
0	0	0	0	1	1
0	0	0	0	1	1
0	0	0	0	1	1
0	0	0	0	1	1
0	0	0	0	1	1
0	0	0	0	1	1
0	0	0	0	1	1
0	0	0	0	1	1
0	0	0	0	1	1
0	0	0	0	1	1
0	0	0	0	1	1
0	0	0	0	1	1
0	0	0	0	1	1
0	0	0	0	1	1
0	0	0	0	1	1
0	0	0	0	1	1
0	0	0	0	1	1
0	0	0	0	1	1
0	0	0	0	1	1
0	0	0	0	1	1
0	0	0	0	1	1

0	0	0	0	1	1
0	0	0	0	1	1
0	0	0	0	1	1
0	0	0	0	1	1
0	0	0	0	1	1
0	0	0	0	1	1
0	0	0	0	1	1
0	0	0	0	1	1
0	0	0	0	1	1
0	0	0	0	1	1
0	0	0	0	1	1
0	0	0	0	1	1
0	0	0	0	1	1
0	0	0	0	1	1
0	0	0	0	1	1
0	0	0	0	1	1
0	0	0	0	1	1
0	0	0	0	1	1
0	0	0	0	1	1
0	0	0	0	1	1
0	0	0	0	1	1
0	0	0	0	1	1
0	0	0	0	1	1
0	0	0	0	1	1
0	0	0	0	1	1
0	0	0	0	1	1
0	0	0	0	1	1
0	0	0	0	1	1
0	0	0	0	1	1
0	0	0	0	1	1
0	0	0	0	1	1
0	0	0	0	1	1
0	0	0	0	1	1
0	0	0	0	1	1
0	0	0	0	1	1
0	0	0	0	1	1
0	0	0	0	1	1
0	0	0	0	1	1
0	0	0	0	1	1
0	0	0	0	1	1
0	0	0	0	1	1
0	0	0	0	1	1
0	0	0	0	1	1
0	0	0	0	1	1
0	0	0	0	1	1

0	0	0	0	1	1
0	0	0	0	1	1
0	0	0	0	1	1
0	0	0	0	1	1
0	0	0	0	1	1
0	0	0	0	1	1
0	0	0	0	1	1
0	0	0	0	1	1
0	0	0	0	1	1
0	0	0	0	1	1
0	0	0	0	1	1
0	0	0	0	1	1
0	0	0	0	1	1
0	0	0	0	1	1
0	0	0	0	1	1
0	0	0	0	1	1
0	0	0	0	1	1
0	0	0	0	1	1
0	0	0	0	1	1
0	0	0	0	1	1
0	0	0	0	1	1
0	0	0	0	1	1
0	0	0	0	1	1
0	0	0	0	1	1
0	0	0	0	1	1
0	0	0	0	1	1
0	0	0	0	1	1
0	0	0	0	1	1
0	0	0	0	1	1
0	0	0	0	1	1
0	0	0	0	1	1
0	0	0	0	1	1
1	1	1	1	1	8
1	1	1	1	1	8
1	1	1	1	1	8
1	1	1	1	1	8
1	1	1	1	1	8
1	1	1	1	1	8
1	1	1	1	1	8
1	1	1	1	1	8
1	1	1	1	1	8
1	1	1	1	1	8
0	1	0	0	0	2
0	1	0	0	0	2

0	1	0	0	0	2
0	1	0	0	0	2
0	1	0	0	0	2
0	1	0	0	0	2
0	1	0	0	0	2
0	1	0	0	0	2
0	1	0	0	0	2
0	1	0	0	0	2
1	1	1	1	1	8
1	1	1	1	1	8
1	1	1	1	1	8
1	1	1	1	1	8
1	1	1	1	1	8
1	1	1	1	1	8
1	1	1	1	1	8
1	1	1	1	1	8
1	1	1	1	1	8
1	1	1	1	1	8
1	1	1	1	1	8
1	1	1	1	1	8
1	1	1	1	1	8
1	1	1	1	1	8
1	1	1	1	1	8
1	1	1	1	1	8
1	1	1	1	1	8
1	1	1	1	1	8
1	1	1	1	1	8
1	1	1	1	1	8
1	1	1	1	1	8
1	1	1	1	1	8
1	1	1	1	1	8
1	1	1	1	1	8
1	1	1	1	1	8
1	1	1	1	1	8
1	1	1	1	1	8
1	1	1	1	1	8
1	1	1	1	1	8
1	1	1	1	1	8
1	1	1	1	1	8
1	1	1	1	1	8
1	1	1	1	1	8
1	1	1	1	1	8
1	1	1	1	1	8
1	1	1	1	1	8

1	1	1	1	1	8
1	1	1	1	1	8
1	1	1	1	1	8
1	1	1	1	1	8
1	1	1	1	1	8
1	1	1	1	1	8
1	1	1	1	1	8
1	1	1	1	1	8
1	1	1	1	1	8
1	1	1	1	1	8
1	1	1	1	1	8
1	1	1	1	1	8
1	1	1	1	1	8
1	1	1	1	1	8
1	1	1	1	1	8
1	1	1	1	1	8
1	1	1	1	1	8
1	1	1	1	1	8
1	1	1	1	1	8
1	1	1	1	1	8
1	1	1	1	1	8
1	1	1	1	1	8
1	1	1	1	1	8
1	1	1	1	1	8
1	1	1	1	1	8
1	1	1	1	1	8
1	1	1	1	1	8
1	1	1	1	1	8
1	1	1	1	1	8
1	1	1	1	1	8
1	1	1	1	1	8
1	1	1	1	1	8
1	1	1	1	1	8
1	1	1	1	1	8
1	1	1	1	1	8
1	1	1	1	1	8
1	1	1	1	1	8
1	1	1	1	1	8
1	1	1	1	1	8
1	1	1	1	1	8
1	1	1	1	1	8
1	1	1	1	1	8
0	1	0	0	0	2
0	1	0	0	0	2

0	1	0	0	0	2
0	1	0	0	0	2
0	1	0	0	0	2
0	1	0	0	0	2
0	1	0	0	0	2
0	1	0	0	0	2
0	1	0	0	0	2
0	1	0	0	0	2
0	1	0	0	0	2
0	1	0	0	0	2
0	1	0	0	0	2
0	1	0	0	0	2
0	1	0	0	0	2
0	1	0	0	0	2
0	1	0	0	0	2
0	1	0	0	0	2
0	1	0	0	0	2
0	1	0	0	0	2
0	1	0	0	0	2
0	1	0	0	0	2
0	1	0	0	0	2
0	1	0	0	0	2
0	0	0	0	1	1
0	0	0	0	1	1
0	0	0	0	1	1
0	0	0	0	1	1
0	0	0	0	1	1
0	0	0	0	1	1
0	0	0	0	1	1
0	0	0	0	1	1
0	0	0	0	1	1
0	0	0	0	1	1
1	1	1	1	1	8
1	1	1	1	1	8
1	1	1	1	1	8
1	1	1	1	1	8
1	1	1	1	1	8
1	1	1	1	1	8
1	1	1	1	1	8
1	1	1	1	1	8
1	1	1	1	1	8
1	1	1	1	1	8
0	1	1	1	0	5
0	1	1	1	0	5

0	1	1	1	0	5
0	1	1	1	0	5
0	1	1	1	0	5
0	1	1	1	0	5
0	1	1	1	0	5
0	1	1	1	0	5
0	1	1	1	0	5
0	1	1	1	0	5
1	1	1	1	1	8
1	1	1	1	1	8
1	1	1	1	1	8
1	1	1	1	1	8
1	1	1	1	1	8
1	1	1	1	1	8
1	1	1	1	1	8
1	1	1	1	1	8
1	1	1	1	1	8
1	1	1	1	1	8
1	0	0	0	0	2
1	0	0	0	0	2
1	0	0	0	0	2
1	0	0	0	0	2
1	0	0	0	0	2
1	0	0	0	0	2
1	0	0	0	0	2
1	0	0	0	0	2
1	0	0	0	0	2
1	0	0	0	0	2
1	0	0	0	0	2
1	0	0	0	0	2
1	0	0	0	0	2
1	0	0	0	0	2
1	0	0	0	0	2
1	0	0	0	0	2
1	0	0	0	0	2
1	0	0	0	0	2
1	0	0	0	0	2
1	0	0	0	0	2
1	0	0	0	0	2
1	0	0	0	0	2
1	0	0	0	0	2
1	0	0	0	0	2
1	0	0	0	0	2
1	0	0	0	0	2

1	0	0	0	0	2
1	0	0	0	0	2
1	0	0	0	0	2
1	0	0	0	0	2
1	0	0	0	0	2
1	0	0	0	0	2
1	0	0	0	0	2
1	0	0	0	0	2
1	0	0	0	0	2
1	0	0	0	0	2
1	0	0	0	0	2
1	0	0	0	0	2
1	0	0	0	0	2
1	0	0	0	0	2
1	0	0	0	0	2
1	0	0	0	0	2
1	0	0	0	0	2
1	0	0	0	0	2
1	0	0	0	0	2
1	0	0	0	0	2
1	0	0	0	0	2
1	0	0	0	0	2
1	0	0	0	0	2
1	0	0	0	0	2
1	0	0	0	0	2
1	0	0	0	0	2
1	0	0	0	0	2
1	0	0	0	0	2
1	0	0	0	0	2
1	0	0	0	0	2
1	0	0	0	0	2
1	0	0	0	0	2
1	0	0	0	0	2
1	0	0	0	0	2
1	0	0	0	0	2
1	0	0	0	0	2
1	0	0	0	0	2
1	0	0	0	0	2
1	0	0	0	0	2
1	0	0	0	0	2
1	0	0	0	0	2
1	0	0	0	0	2
1	0	0	0	0	2
1	0	0	0	0	2

1	0	0	0	0	2
1	0	0	0	0	2
1	0	0	0	0	2
1	0	0	0	0	2
1	0	0	0	0	2
1	0	0	0	0	2
1	0	0	0	0	2
1	0	0	0	0	2
1	0	0	0	0	2
1	0	0	0	0	2
1	0	0	0	0	2
1	0	0	0	0	2
1	0	0	0	0	2
1	0	0	0	0	2
1	0	0	0	0	2
1	0	0	0	0	2
1	0	0	0	0	2
1	0	0	0	0	2
1	0	0	0	0	2
1	0	0	0	0	2
1	0	0	0	0	2
1	0	0	0	0	2
1	0	0	0	0	2
1	0	0	0	0	2
1	0	0	0	0	2
1	0	0	0	0	2
1	0	0	0	0	2
1	0	0	0	0	2
1	0	0	0	0	2
1	0	0	0	0	2
1	0	0	0	0	2
1	0	0	0	0	2
1	0	0	0	0	2
1	0	0	0	0	2
1	0	0	0	0	2
1	0	0	0	0	2
1	0	0	0	0	2
1	0	0	0	0	2
1	0	0	0	0	2
1	0	0	0	0	2
1	0	0	0	0	2
1	0	0	0	0	2
0	1	1	1	0	4
0	1	1	1	0	4

0	1	1	1	0	4
0	1	1	1	0	4
0	1	1	1	0	4
0	1	1	1	0	4
0	1	1	1	0	4
0	1	1	1	0	4
0	1	1	1	0	4
0	1	1	1	0	4
0	1	1	1	0	4
0	1	1	1	0	4
0	1	1	1	0	4
0	1	1	1	0	4
0	1	1	1	0	4
0	1	1	1	0	4
0	1	1	1	0	4
0	1	1	1	0	4
0	1	1	1	0	4
0	1	1	1	0	4
0	1	1	1	0	4
0	1	1	1	0	4
0	1	1	1	0	4
0	1	1	1	0	4
0	1	1	1	0	4
0	1	1	1	0	4
0	1	1	1	0	4
0	1	1	1	0	4
0	1	1	1	0	4
0	1	1	1	0	4
0	1	1	1	0	4
0	1	1	1	0	4
0	1	1	1	0	4
0	1	1	1	0	4
0	1	1	1	0	4
0	1	1	1	0	4
0	1	1	1	0	4
0	1	1	1	0	4
0	1	1	1	0	4
0	1	1	1	0	4
0	1	1	1	0	4
0	1	1	1	0	4
0	1	1	1	0	4
0	1	1	1	0	4
0	1	1	1	0	4
0	1	1	1	0	4
0	1	1	1	0	4

0	1	1	1	0	4
0	1	1	1	0	4
0	1	1	1	0	4
0	1	1	1	0	4
0	1	1	1	0	4
0	1	1	1	0	4
0	1	1	1	0	4
0	1	1	1	0	4
0	1	1	1	0	4
0	1	1	1	0	4
0	1	1	1	1	5
0	1	1	1	1	5
0	1	1	1	1	5
0	1	1	1	1	5
0	1	1	1	1	5
0	1	1	1	1	5
0	1	1	1	1	5
0	1	1	1	1	5
0	1	1	1	1	5
0	1	1	1	1	5
0	1	1	1	1	5
0	1	1	1	1	5
0	1	1	1	1	5
0	1	1	1	1	5
0	1	1	1	1	5
0	1	1	1	1	5
0	1	1	1	1	5
0	1	1	1	1	5
0	1	1	1	1	5
0	1	1	1	1	5
0	1	1	1	1	5
0	1	1	1	1	5
0	1	1	1	1	5
0	1	1	1	1	5
0	1	1	1	1	5
0	1	1	1	1	5
0	1	1	1	1	5
0	1	1	1	1	5
0	1	1	1	1	5
0	1	1	1	1	5
0	1	1	1	1	5
0	1	1	1	1	5
0	1	1	1	1	5
0	1	1	1	1	5

0	1	1	1	1	5
0	1	1	1	1	5
0	1	1	1	1	5
0	1	1	1	1	5
0	1	1	1	1	5
0	1	1	1	1	5
0	1	1	1	1	5
0	1	1	1	1	5
0	1	1	1	1	5
0	1	1	1	1	5
0	1	1	1	1	5
0	1	1	1	1	5
0	1	1	1	1	5
0	1	1	1	1	5
0	1	1	1	1	5
0	1	1	1	1	5
0	1	1	1	1	5
0	1	1	1	1	5
0	1	1	1	1	5
0	1	1	1	1	5
0	1	1	1	1	5
0	1	1	1	1	5
1	1	1	1	1	8
1	1	1	1	1	8
1	1	1	1	1	8
1	1	1	1	1	8
1	1	1	1	1	8
1	1	1	1	1	8
1	1	1	1	1	8
1	1	1	1	1	8
1	1	1	1	1	8
1	1	1	1	1	8
1	1	1	1	1	8
1	1	1	1	1	8
1	1	1	1	1	8
1	1	1	1	1	8
1	1	1	1	1	8
1	1	1	1	1	8
1	1	1	1	1	8
1	1	1	1	1	8
1	1	1	1	1	8
1	1	1	1	1	8
1	1	1	1	1	8
1	1	1	1	1	8

1	1	1	1	1	8
1	1	1	1	1	8
0	0	0	0	1	1
0	0	0	0	1	1
0	0	0	0	1	1
0	0	0	0	1	1
0	0	0	0	1	1
0	0	0	0	1	1
0	0	0	0	1	1
0	0	0	0	1	1
0	0	0	0	1	1
0	0	0	0	1	1
0	0	0	0	1	1
0	0	0	0	1	1
0	0	0	0	1	1
0	0	0	0	1	1
0	0	0	0	1	1
0	0	0	0	1	1
0	0	0	0	1	1
0	0	0	0	1	1
0	0	0	0	1	1
0	0	0	0	1	1
0	0	0	0	1	1
0	0	0	0	1	1
0	0	0	0	1	1
0	0	0	0	1	1
0	0	0	0	1	1
0	0	0	0	1	1
0	0	0	0	1	1
0	0	0	0	1	1
0	0	0	0	1	1
0	0	0	0	1	1
0	0	0	0	1	1
0	0	0	0	1	1
0	0	0	0	1	1
0	0	0	0	1	1
0	0	0	0	1	1
0	0	0	0	1	1
0	0	0	0	1	1
0	0	0	0	1	1
0	0	0	0	1	1
0	0	0	0	1	1
0	0	0	0	1	1
0	0	0	0	1	1

0	0	0	0	1	1
0	0	0	0	1	1
0	0	0	0	1	1
0	0	0	0	1	1
0	0	0	0	1	1
0	0	0	0	1	1
0	0	0	0	1	1
0	0	0	0	1	1
0	0	0	0	1	1
0	0	0	0	1	1
0	0	0	0	1	1
0	0	0	0	1	1
0	0	0	0	1	1
0	0	0	0	1	1
1	1	1	1	1	8
1	1	1	1	1	8
1	1	1	1	1	8
1	1	1	1	1	8
1	1	1	1	1	8
1	1	1	1	1	8
1	1	1	1	1	8
1	1	1	1	1	8
1	1	1	1	1	8
1	1	1	1	1	8
1	1	1	1	1	8
1	1	1	1	1	8
1	1	1	1	1	8
1	1	1	1	1	8
1	1	1	1	1	8
1	1	1	1	1	8
1	1	1	1	1	8
1	1	1	1	1	8
1	1	1	1	1	8
1	1	1	1	1	8
1	1	1	1	1	8
1	1	1	1	1	8
1	1	1	1	1	8
1	1	1	1	1	8
1	1	1	1	1	8
1	1	1	1	1	8
1	1	1	1	1	8
1	1	1	1	1	8
1	1	1	1	1	8
1	1	1	1	1	8

1	1	1	1	1	8
1	1	1	1	1	8
1	1	1	1	1	8
1	1	1	1	1	8
1	1	1	1	1	8
1	1	1	1	1	8
1	1	1	1	1	8
1	1	1	1	1	8
1	1	1	1	1	8
1	1	1	1	1	8
1	1	1	1	1	8
1	1	1	1	1	8
1	1	1	1	1	8
1	1	1	1	1	8
1	1	1	1	1	8
1	1	1	1	1	8
1	1	1	1	1	8
1	1	1	1	1	8
1	1	1	1	1	8
1	1	1	1	1	8
1	1	1	1	1	8
1	1	1	1	1	8
1	1	1	1	1	8
1	1	1	1	1	8
1	1	1	1	1	8
1	1	1	1	1	8
1	1	1	1	1	8
1	1	1	1	1	8
1	1	1	1	1	8
1	1	1	1	1	8
1	1	1	1	1	8
1	1	1	1	1	8
1	1	1	1	1	8
1	1	1	1	1	8
1	1	1	1	1	8
1	1	1	1	1	8
1	1	1	1	1	8
1	1	1	1	1	8
1	1	1	1	1	8
1	1	1	1	1	8
1	1	1	1	1	8
1	1	1	1	1	8
1	1	1	1	1	8
1	1	1	1	1	8

1	1	1	1	1	8
1	1	1	1	1	8
1	1	1	1	1	8
1	1	1	1	1	8
1	1	1	1	1	8
1	1	1	1	1	8
1	1	1	1	1	8
1	1	1	1	1	8
1	1	1	1	1	8
1	1	1	1	1	8
1	1	1	1	1	8
1	1	1	1	1	8
1	1	1	1	1	8
1	1	1	1	1	8
1	1	1	1	1	8
1	1	1	1	1	8
1	1	1	1	1	8
1	1	1	1	1	8
1	1	1	1	1	8
1	1	1	1	1	8
1	1	1	1	1	8
1	1	1	1	1	8
1	1	1	1	1	8
1	1	1	1	1	8
1	1	1	1	1	8
1	1	1	1	1	8
1	1	1	1	1	8
1	1	1	1	1	8
1	1	1	1	1	8
1	1	1	1	1	8
1	1	1	1	1	8
1	1	1	1	1	8
1	1	1	1	1	8
1	1	1	1	1	8
1	1	1	1	1	8
1	1	1	1	1	8
1	1	1	1	1	8
1	1	1	1	1	8
0	1	0	0	0	2
0	1	0	0	0	2
0	1	0	0	0	2
0	1	0	0	0	2
0	1	0	0	0	2
0	1	0	0	0	2

0	1	0	0	0	2
0	1	0	0	0	2
0	1	0	0	0	2
0	1	0	0	0	2
0	1	0	0	0	2
0	1	0	0	0	2
0	1	0	0	0	2
0	1	0	0	0	2
0	1	0	0	0	2
0	1	0	0	0	2
0	1	0	0	0	2
0	1	0	0	0	2
0	1	0	0	0	2
0	1	0	0	0	2
0	1	0	0	0	2
0	1	0	0	0	2
0	1	0	0	0	2
0	1	0	0	0	2
0	1	0	0	0	2
0	1	0	0	0	2
0	1	0	0	0	2
0	1	0	0	0	2
0	1	0	0	0	2
0	1	0	0	0	2
0	1	0	0	0	2
0	1	0	0	0	2
1	1	1	1	1	8
1	1	1	1	1	8
1	1	1	1	1	8
1	1	1	1	1	8
1	1	1	1	1	8
1	1	1	1	1	8
1	1	1	1	1	8
1	1	1	1	1	8
1	1	1	1	1	8
1	1	1	1	1	8
1	1	1	1	1	8
1	1	1	1	1	8
1	1	1	1	1	8
1	1	1	1	1	8
1	1	1	1	1	8
1	1	1	1	1	8
1	1	1	1	1	8
1	1	1	1	1	8

1	1	1	1	1	8
1	1	1	1	1	8
1	1	1	1	1	8
1	1	1	1	1	8
1	1	1	1	1	8
1	1	1	1	1	8
1	1	1	1	1	8
1	1	1	1	1	8
1	1	1	1	1	8
1	1	1	1	1	8
1	1	1	1	1	8
1	1	1	1	1	8
1	1	1	1	1	8
1	1	1	1	1	8
1	1	1	1	1	8
1	1	1	1	1	8
1	1	1	1	1	8
1	1	1	1	1	8
1	1	1	1	1	8
1	1	1	1	1	8
1	1	1	1	1	8
1	1	1	1	1	8
1	1	1	1	1	8
1	1	1	1	1	8
1	1	1	1	1	8
1	1	1	1	1	8
1	1	1	1	1	8
1	1	1	1	1	8
1	1	1	1	1	8
1	1	1	1	1	8
1	1	1	1	1	8
1	1	1	1	1	8
1	1	1	1	1	8
1	1	1	1	1	8
1	1	1	1	1	8
1	1	1	1	1	8
1	1	1	1	1	8
1	1	1	1	1	8
1	1	1	1	1	8
1	1	1	1	1	8
1	1	1	1	1	8
1	1	1	1	1	8
1	1	1	1	1	8
1	1	1	1	1	8

1	1	1	1	1	8
1	1	1	1	1	8
1	1	1	1	1	8
1	1	1	1	1	8
1	1	1	1	1	8
1	1	1	1	1	8
1	1	1	1	1	8
1	1	1	1	1	8
1	1	1	1	1	8
1	1	1	1	1	8
0	1	0	0	0	2
0	1	0	0	0	2
0	1	0	0	0	2
0	1	0	0	0	2
0	1	0	0	0	2
0	1	0	0	0	2
0	1	0	0	0	2
0	1	0	0	0	2
0	1	0	0	0	2
0	1	0	0	0	2
0	1	0	0	0	2
0	1	0	0	0	2
0	1	0	0	0	2
0	1	0	0	0	2
0	1	0	0	0	2
0	1	0	0	0	2
0	1	0	0	0	2
0	1	0	0	0	2
0	1	0	0	0	2
0	1	0	0	0	2
0	1	0	0	0	2
0	1	0	0	0	2
0	1	0	0	0	2
0	1	0	0	0	2
0	1	0	0	0	2
0	1	0	0	0	2
0	1	0	0	0	2
0	1	0	0	0	2
0	1	0	0	0	2
0	1	0	0	0	2
0	1	0	0	0	2
0	1	0	0	0	2
0	1	0	0	0	2
0	1	0	0	0	2

0	1	0	0	0	2
0	1	0	0	0	2
0	1	0	0	0	2
0	1	0	0	0	2
0	1	0	0	0	2
0	1	0	0	0	2
1	1	1	1	1	8
1	1	1	1	1	8
1	1	1	1	1	8
1	1	1	1	1	8
1	1	1	1	1	8
1	1	1	1	1	8
1	1	1	1	1	8
1	1	1	1	1	8
1	1	1	1	1	8
1	1	1	1	1	8
1	1	1	1	1	8
1	1	1	1	1	8
1	1	1	1	1	8
1	1	1	1	1	8
1	1	1	1	1	8
1	1	1	1	1	8
1	1	1	1	1	8
1	1	1	1	1	8
1	1	1	1	1	8
1	1	1	1	1	8
1	1	1	1	1	8
1	1	1	1	1	8
1	1	1	1	1	8
1	1	1	1	1	8
1	1	1	1	1	8
1	1	1	1	1	8
1	1	1	1	1	8
1	1	1	1	1	8
1	1	1	1	1	8
1	1	1	1	1	8
1	1	1	1	1	8
1	1	1	1	1	8
1	1	1	1	1	8
1	1	1	1	1	8
1	1	1	1	1	8
1	1	1	1	1	8
1	1	1	1	1	8
1	1	1	1	1	8

1	1	1	1	1	8
1	1	1	1	1	8
1	1	1	1	1	8
1	1	1	1	1	8
1	1	1	1	1	8
1	1	1	1	1	8
1	1	1	1	1	8
1	1	1	1	1	8
1	1	1	1	1	8
1	1	1	1	1	8
1	1	1	1	1	8
1	1	1	1	1	8
1	1	1	1	1	8
1	1	1	1	1	8
1	1	1	1	1	8
1	1	1	1	1	8
1	1	1	1	1	8
1	1	1	1	1	8
1	1	1	1	1	8
1	1	1	1	1	8
1	1	1	1	1	8
1	1	1	1	1	8
1	1	1	1	1	8
1	1	1	1	1	8
1	1	1	1	1	8
1	1	1	1	1	8
1	1	1	1	1	8
1	1	1	1	1	8
1	1	1	1	1	8
1	1	1	1	1	8
1	1	1	1	1	8
1	1	1	1	1	8
1	1	1	1	1	8
1	1	1	1	1	8
1	1	1	1	1	8
1	1	1	1	1	8
1	1	1	1	1	8
1	1	1	1	1	8
1	1	1	1	1	8
1	1	1	1	1	8
1	1	1	1	1	8
1	1	1	1	1	8
1	1	1	1	1	8
1	1	1	1	1	8

1	1	1	1	1	8
1	1	1	1	1	8
1	1	1	1	1	8
1	1	1	1	1	8
1	1	1	1	1	8
1	1	1	1	1	8
1	1	1	1	1	8
1	1	1	1	1	8
1	1	1	1	1	8
1	1	1	1	1	8
1	1	1	1	1	8
1	1	1	1	1	8
1	1	1	1	1	8
1	1	1	1	1	8
1	1	1	1	1	8
1	1	1	1	1	8
1	1	1	1	1	8
1	1	1	1	1	8
1	1	1	1	1	8
1	1	1	1	1	8
1	1	1	1	1	8
1	1	1	1	1	8
1	1	1	1	1	8
1	1	1	1	1	8
1	1	1	1	1	8
1	1	1	1	1	8
1	1	1	1	1	8
1	1	1	1	1	8
1	1	1	1	1	8
1	1	1	1	1	8
1	1	1	1	1	8
1	1	1	1	1	8
1	1	1	1	1	8
1	1	1	1	1	8
1	1	1	1	1	8
1	1	1	1	1	8
1	1	1	1	1	8
1	1	1	1	1	8
1	1	1	1	1	8
1	1	1	1	1	8
1	1	1	1	1	8
1	1	1	1	1	8
1	1	1	1	1	8
1	1	1	1	1	8

1	1	1	1	1	8
1	1	1	1	1	8
1	1	1	1	1	8
1	1	1	1	1	8
1	1	1	1	1	8
1	1	1	1	1	8
1	1	1	1	1	8
1	1	1	1	1	8
1	1	1	1	1	8
1	1	1	1	1	8
1	1	1	1	1	8
1	1	1	1	1	8
1	1	1	1	1	8
1	1	1	1	1	8
1	1	1	1	1	8
1	1	1	1	1	8
1	1	1	1	1	8
1	1	1	1	1	8
1	1	1	1	1	8
1	1	1	1	1	8
1	1	1	1	1	8
1	1	1	1	1	8
1	1	1	1	1	8
1	1	1	1	1	8
1	1	1	1	1	8
1	1	1	1	1	8
1	1	1	1	1	8
1	1	1	1	1	8
1	1	1	1	1	8
1	1	1	1	1	8
1	1	1	1	1	8
1	1	1	1	1	8
1	1	1	1	1	8
1	1	1	1	1	8
1	1	1	1	1	8
1	1	1	1	1	8
1	1	1	1	1	8
1	1	1	1	1	8
1	1	1	1	1	8
1	1	1	1	1	8
1	1	1	1	1	8
1	1	1	1	1	8
1	1	1	1	1	8
1	1	1	1	1	8
1	1	1	1	1	8

1	1	1	1	1	8
1	1	1	1	1	8
1	1	1	1	1	8
1	1	1	1	1	8
1	1	1	1	1	8
1	1	1	1	1	8
1	1	1	1	1	8
1	1	1	1	1	8
1	1	1	1	1	8
1	1	1	1	1	8
1	1	1	1	1	8
1	1	1	1	1	8
1	1	1	1	1	8
1	1	1	1	1	8
1	1	1	1	1	8
1	1	1	1	1	8
1	1	1	1	1	8
1	1	1	1	1	8
1	1	1	1	1	8
1	1	1	1	1	8
1	1	1	1	1	8
1	1	1	1	1	8
1	1	1	1	1	8
1	1	1	1	1	8
1	1	1	1	1	8
1	1	1	1	1	8
1	1	1	1	1	8
1	1	1	1	1	8
1	1	1	1	1	8
1	1	1	1	1	8
1	1	1	1	1	8
1	1	1	1	1	8
1	1	1	1	1	8
1	1	1	1	1	8
1	1	1	1	1	8
1	1	1	1	1	8
1	1	1	1	1	8
1	1	1	1	1	8
1	1	1	1	1	8
1	1	1	1	1	8
1	1	1	1	1	8
1	1	1	1	1	8
1	1	1	1	1	8
1	1	1	1	1	8

1	1	1	1	1	8
1	1	1	1	1	8
1	1	1	1	1	8
1	1	1	1	1	8
1	1	1	1	1	8
1	1	1	1	1	8
1	1	1	1	1	8
1	1	1	1	1	8
1	1	1	1	1	8
1	1	1	1	1	8
1	1	1	1	1	8
1	1	1	1	1	8
1	1	1	1	1	8
1	1	1	1	1	8
1	1	1	1	1	8
1	1	1	1	1	8
1	1	1	1	1	8
1	1	1	1	1	8
1	1	1	1	1	8
1	1	1	1	1	8
1	1	1	1	1	8
1	1	1	1	1	8
1	1	1	1	1	8
1	1	1	1	1	8
1	1	1	1	1	8
1	1	1	1	1	8
1	1	1	1	1	8
1	1	1	1	1	8
1	1	1	1	1	8
1	1	1	1	1	8
1	1	1	1	1	8
1	1	1	1	1	8
1	1	1	1	1	8
1	1	1	1	1	8
1	1	1	1	1	8
1	1	1	1	1	8
1	1	1	1	1	8
1	1	1	1	1	8
1	1	1	1	1	8
1	1	1	1	1	8
1	1	1	1	1	8
1	1	1	1	1	8
1	1	1	1	1	8
1	1	1	1	1	8

1	1	1	1	1	8
1	1	1	1	1	8
1	1	1	1	1	8
1	1	1	1	1	8
1	1	1	1	1	8
1	1	1	1	1	8
1	1	1	1	1	8
1	1	1	1	1	8
1	1	1	1	1	8
1	1	1	1	1	8
1	1	1	1	1	8
1	1	1	1	1	8
1	1	1	1	1	8
1	1	1	1	1	8
1	1	1	1	1	8
1	1	1	1	1	8
1	1	1	1	1	8
1	1	1	1	1	8
1	1	1	1	1	8
1	1	1	1	1	8
1	1	1	1	1	8
1	1	1	1	1	8
1	1	1	1	1	8
1	1	1	1	1	8
1	1	1	1	1	8
1	1	1	1	1	8
1	1	1	1	1	8
1	1	1	1	1	8
1	1	1	1	1	8
1	1	1	1	1	8
1	1	1	1	1	8
1	1	1	1	1	8
1	1	1	1	1	8
1	1	1	1	1	8
1	1	1	1	1	8
1	1	1	1	1	8
1	1	1	1	1	8
1	1	1	1	1	8
1	1	1	1	1	8
1	1	1	1	1	8
1	1	1	1	1	8
1	1	1	1	1	8
1	1	1	1	1	8
1	1	1	1	1	8

1	1	1	1	1	8
1	1	1	1	1	8
1	1	1	1	1	8
1	1	1	1	1	8
1	1	1	1	1	8
1	1	1	1	1	8
1	1	1	1	1	8
1	1	1	1	1	8
1	1	1	1	1	8
1	1	1	1	1	8
1	1	1	1	1	8
1	1	1	1	1	8
1	1	1	1	1	8
1	1	1	1	1	8
1	1	1	1	1	8
1	1	1	1	1	8
1	1	1	1	1	8
1	1	1	1	1	8
1	1	1	1	1	8
1	1	1	1	1	8
1	1	1	1	1	8
1	1	1	1	1	8
1	1	1	1	1	8
1	1	1	1	1	8
1	1	1	1	1	8
1	1	1	1	1	8
1	1	1	1	1	8
1	1	1	1	1	8
1	1	1	1	1	8
1	1	1	1	1	8
1	1	1	1	1	8
1	1	1	1	1	8
1	1	1	1	1	8
1	1	1	1	1	8
1	1	1	1	1	8
1	1	1	1	1	8
1	1	1	1	1	8
1	1	1	1	1	8
1	1	1	1	1	8
1	1	1	1	1	8
1	1	1	1	1	8
1	1	1	1	1	8
1	1	1	1	1	8
1	1	1	1	1	8

1	1	1	1	1	8
1	1	1	1	1	8
1	1	1	1	1	8
1	1	1	1	1	8
1	1	1	1	1	8
1	1	1	1	1	8
1	1	1	1	1	8
1	1	1	1	1	8
1	1	1	1	1	8
1	1	1	1	1	8
1	1	1	1	1	8
1	1	1	1	1	8
1	1	1	1	1	8
1	1	1	1	1	8
1	1	1	1	1	8
1	1	1	1	1	8
1	1	1	1	1	8
1	1	1	1	1	8
1	1	1	1	1	8
1	1	1	1	1	8
1	1	1	1	1	8
1	1	1	1	1	8
1	1	1	1	1	8
1	1	1	1	1	8
1	1	1	1	1	8
1	1	1	1	1	8
1	1	1	1	1	8
1	1	1	1	1	8
1	1	1	1	1	8
1	1	1	1	1	8
1	1	1	1	1	8
1	1	1	1	1	8
1	1	1	1	1	8
1	1	1	1	1	8
1	1	1	1	1	8
1	1	1	1	1	8
1	1	1	1	1	8
1	1	1	1	1	8
1	1	1	1	1	8
1	1	1	1	1	8
1	1	1	1	1	8
1	1	1	1	1	8
1	1	1	1	1	8
1	1	1	1	1	8

1	1	1	1	1	8
1	1	1	1	1	8
1	1	1	1	1	8
1	1	1	1	1	8
1	1	1	1	1	8
1	1	1	1	1	8
1	1	1	1	1	8
1	1	1	1	1	8
1	1	1	1	1	8
1	1	1	1	1	8
1	1	1	1	1	8
1	1	1	1	1	8
1	1	1	1	1	8
1	1	1	1	1	8
1	1	1	1	1	8
1	1	1	1	1	8
1	1	1	1	1	8
1	1	1	1	1	8
1	1	1	1	1	8
1	1	1	1	1	8
1	1	1	1	1	8
1	1	1	1	1	8
1	1	1	1	1	8
1	1	1	1	1	8
1	1	1	1	1	8
1	1	1	1	1	8
1	1	1	1	1	8
1	1	1	1	1	8
1	1	1	1	1	8
1	1	1	1	1	8
1	1	1	1	1	8
1	1	1	1	1	8
1	1	1	1	1	8
1	1	1	1	1	8
1	1	1	1	1	8
1	1	1	1	1	8
1	1	1	1	1	8
1	1	1	1	1	8
1	1	1	1	1	8
1	1	1	1	1	8
1	1	1	1	1	8
1	1	1	1	1	8
1	1	1	1	1	8
1	1	1	1	1	8

1	1	1	1	1	8
1	1	1	1	1	8
1	1	1	1	1	8
1	1	1	1	1	8
1	1	1	1	1	8
1	1	1	1	1	8
1	1	1	1	1	8
1	1	1	1	1	8
1	1	1	1	1	8
1	1	1	1	1	8
1	1	1	1	1	8
1	1	1	1	1	8
1	1	1	1	1	8
1	1	1	1	1	8
1	1	1	1	1	8
1	1	1	1	1	8
1	1	1	1	1	8
1	1	1	1	1	8
1	1	1	1	1	8
1	1	1	1	1	8
1	1	1	1	1	8
1	1	1	1	1	8
1	1	1	1	1	8
1	1	1	1	1	8
1	1	1	1	1	8
1	1	1	1	1	8
1	1	1	1	1	8
1	1	1	1	1	8
1	1	1	1	1	8
1	1	1	1	1	8
1	1	1	1	1	8
1	1	1	1	1	8
1	1	1	1	1	8
1	1	1	1	1	8
1	1	1	1	1	8
1	1	1	1	1	8
1	1	1	1	1	8
1	1	1	1	1	8
1	1	1	1	1	8
1	1	1	1	1	8
1	1	1	1	1	8
1	1	1	1	1	8
1	1	1	1	1	8
1	1	1	1	1	8

1	1	1	1	1	8
1	1	1	1	1	8
1	1	1	1	1	8
1	1	1	1	1	8
1	1	1	1	1	8
1	1	1	1	1	8
1	1	1	1	1	8
1	1	1	1	1	8
1	1	1	1	1	8
1	1	1	1	1	8
1	1	1	1	1	8
1	1	1	1	1	8
1	1	1	1	1	8
1	1	1	1	1	8
1	1	1	1	1	8
1	1	1	1	1	8
1	1	1	1	1	8
1	1	1	1	1	8
1	1	1	1	1	8
1	1	1	1	1	8
1	1	1	1	1	8
1	1	1	1	1	8
1	1	1	1	1	8
1	1	1	1	1	8
1	1	1	1	1	8
1	1	1	1	1	8
1	1	1	1	1	8
1	1	1	1	1	8
1	1	1	1	1	8
1	1	1	1	1	8
1	1	1	1	1	8
1	1	1	1	1	8
1	1	1	1	1	8
1	1	1	1	1	8
1	1	1	1	1	8
1	1	1	1	1	8
1	1	1	1	1	8
1	1	1	1	1	8
1	1	1	1	1	8
1	1	1	1	1	8
1	1	1	1	1	8
1	1	1	1	1	8
1	1	1	1	1	8
1	1	1	1	1	8

1	1	1	1	1	8
1	1	1	1	1	8
1	1	1	1	1	8
1	1	1	1	1	8
1	1	1	1	1	8
1	1	1	1	1	8
1	1	1	1	1	8
1	1	1	1	1	8
1	1	1	1	1	8
1	1	1	1	1	8
1	1	1	1	1	8
1	1	1	1	1	8
1	1	1	1	1	8
1	1	1	1	1	8
1	1	1	1	1	8
1	1	1	1	1	8
1	1	1	1	1	8
1	1	1	1	1	8
1	1	1	1	1	8
1	1	1	1	1	8
1	1	1	1	1	8
1	1	1	1	1	8
1	1	1	1	1	8
1	1	1	1	1	8
1	1	1	1	1	8
1	1	1	1	1	8
1	1	1	1	1	8
1	1	1	1	1	8
1	1	1	1	1	8
1	1	1	1	1	8
1	1	1	1	1	8
1	1	1	1	1	8
1	1	1	1	1	8
1	1	1	1	1	8
1	1	1	1	1	8
1	1	1	1	1	8
1	1	1	1	1	8
1	1	1	1	1	8
1	1	1	1	1	8
1	1	1	1	1	8
1	1	1	1	1	8
1	1	1	1	1	8
0	1	1	1	0	4
0	1	1	1	0	4

0	1	1	1	0	4
0	1	1	1	0	4
0	1	1	1	0	4
0	1	1	1	0	4
0	1	1	1	0	4
0	1	1	1	0	4
0	1	1	1	0	4
0	1	1	1	0	4
0	1	1	1	0	4
0	1	1	1	0	4
0	1	1	1	0	4
0	1	1	1	0	4
0	1	1	1	0	4
0	1	1	1	0	4
0	1	1	1	0	4
0	1	1	1	0	4
0	1	1	1	0	4
0	1	1	1	0	4
0	1	1	1	0	4
0	1	1	1	0	4
0	1	1	1	0	4
0	1	1	1	0	4
0	1	1	1	0	4
0	1	1	1	0	4
0	1	1	1	0	4
0	1	1	1	0	4
0	1	1	1	0	4
0	1	1	1	0	4
0	1	1	1	0	4
0	1	1	1	0	4
0	1	1	1	0	4
0	1	1	1	0	4
0	1	1	1	0	4
0	1	1	1	0	4
0	1	1	1	0	4
0	1	1	1	0	4
0	1	1	1	0	4
0	1	1	1	0	4
0	1	1	1	0	4
0	1	1	1	0	4
0	1	1	1	0	4
0	1	1	1	0	4
0	1	1	1	0	4
0	1	1	1	0	4

0	1	1	1	0	4
0	1	1	1	0	4
0	1	1	1	0	4
0	1	1	1	0	4
0	1	1	1	0	4
0	1	1	1	0	4
0	1	1	1	0	4
0	1	1	1	0	4
0	1	1	1	0	4
0	1	1	1	0	4
0	1	1	1	1	5
0	1	1	1	1	5
0	1	1	1	1	5
0	1	1	1	1	5
0	1	1	1	1	5
0	1	1	1	1	5
0	1	1	1	1	5
0	1	1	1	1	5
0	1	1	1	1	5
0	1	1	1	1	5
0	1	1	1	1	5
0	1	1	1	1	5
0	1	1	1	1	5
0	1	1	1	1	5
0	1	1	1	1	5
0	1	1	1	1	5
0	1	1	1	1	5
0	1	1	1	1	5
0	1	1	1	1	5
0	1	1	1	1	5
0	1	1	1	1	5
0	1	1	1	1	5
0	1	1	1	1	5
0	1	1	1	1	5
0	1	1	1	1	5
0	1	1	1	1	5
0	1	1	1	1	5
0	1	1	1	1	5
0	1	1	1	1	5
0	1	1	1	1	5
0	1	1	1	1	5
0	1	1	1	1	5
0	1	1	1	1	5
0	1	1	1	1	5

0	1	1	1	1	5
0	1	1	1	1	5
0	1	1	1	1	5
0	1	1	1	1	5
0	1	1	1	1	5
0	1	1	1	1	5
0	1	1	1	1	5
0	1	1	1	1	5
0	1	1	1	1	5
0	1	1	1	1	5
0	1	1	1	1	5
0	1	1	1	1	5
0	1	1	1	1	5
0	1	1	1	1	5
0	1	1	1	1	5
0	1	1	1	1	5
0	1	1	1	1	5
0	1	1	1	1	5
0	1	1	1	1	5
0	1	1	1	1	5
0	1	1	1	1	5
0	1	1	1	1	5
1	1	1	1	1	8
1	1	1	1	1	8
1	1	1	1	1	8
1	1	1	1	1	8
1	1	1	1	1	8
1	1	1	1	1	8
1	1	1	1	1	8
1	1	1	1	1	8
0	1	0	0	0	2
0	1	0	0	0	2
0	1	0	0	0	2
0	1	0	0	0	2
0	1	0	0	0	2
0	1	0	0	0	2
0	1	0	0	0	2
0	1	0	0	0	2
1	1	1	1	1	8
1	1	1	1	1	8
1	1	1	1	1	8
1	1	1	1	1	8
1	1	1	1	1	8
1	1	1	1	1	8

1	1	1	1	1	8
1	1	1	1	1	8
1	1	1	1	1	8
1	1	1	1	1	8
1	1	1	1	1	8
1	1	1	1	1	8
1	1	1	1	1	8
1	1	1	1	1	8
1	1	1	1	1	8
1	1	1	1	1	8
1	1	1	1	1	8
1	1	1	1	1	8
1	1	1	1	1	8
1	1	1	1	1	8
1	1	1	1	1	8
1	1	1	1	1	8
1	1	1	1	1	8
1	1	1	1	1	8
1	1	1	1	1	8
1	1	1	1	1	8
1	1	1	1	1	8
1	1	1	1	1	8
1	1	1	1	1	8
1	1	1	1	1	8
1	1	1	1	1	8
1	1	1	1	1	8
1	1	1	1	1	8
1	1	1	1	1	8
1	1	1	1	1	8
1	1	1	1	1	8
1	1	1	1	1	8
1	1	1	1	1	8
1	1	1	1	1	8
1	1	1	1	1	8
1	1	1	1	1	8
1	1	1	1	1	8
1	1	1	1	1	8
1	1	1	1	1	8
1	1	1	1	1	8
1	1	1	1	1	8
1	1	1	1	1	8
1	1	1	1	1	8
1	1	1	1	1	8
1	1	1	1	1	8

1	1	1	1	1	8
1	1	1	1	1	8
1	1	1	1	1	8
1	1	1	1	1	8
1	1	1	1	1	8
1	1	1	1	1	8
1	1	1	1	1	8
1	1	1	1	1	8
1	1	1	1	1	8
